# Using bioinformatics to investigate functional diversity: a case study of MHC diversity in koalas

**DOI:** 10.1007/s00251-024-01356-6

**Published:** 2024-10-05

**Authors:** Luke W. Silver, Elspeth A. McLennan, Julian Beaman, Karen Burke da Silva, Peter Timms, Carolyn J. Hogg, Katherine Belov

**Affiliations:** 1 School of Life and Environmental Sciences, The University of Sydney, Camperdown, NSW 2006 Australia; 2 Australian Research Council Centre of Excellence for Innovations in Peptide and Protein Science, University of Sydney, Camperdown, NSW 2006 Australia; 3 College of Science and Engineering, Flinders University, Bedford Park, South Australia 5001 Australia; 4 Genecology Research Centre, University of the Sunshine Coast, Sippy Downs, QLD 4556 Australia

**Keywords:** Whole genome resequencing, TLR, Functional genomics, Bioinformatics, MHC, Koalas

## Abstract

Conservation genomics can greatly improve conservation outcomes of threatened populations, including those impacted by disease. Understanding diversity within immune gene families, including the major histocompatibility complex (MHC) and toll-like receptors (TLR), is important due to the role they play in disease resilience and susceptibility. With recent advancements in sequencing technologies and bioinformatic tools, the cost of generating high-quality sequence data has significantly decreased and made it possible to investigate diversity across entire gene families in large numbers of individuals compared to investigating only a few genes or a few populations previously. Here, we use the koala as a case study for investigating functional diversity across populations. We utilised previous target enrichment data and 438 whole genomes to firstly, determine the level of sequencing depth required to investigate MHC diversity and, secondly, determine the current level of diversity in MHC genes in koala populations. We determined for low complexity, conserved genes such as TLR genes 10 × sequencing depth is sufficient to reliably genotype more than 90% of variants, whereas for complex genes such as the MHC greater than 20 × and preferably 30 × sequencing depth is required. We used whole genome data to identify 270 biallelic SNPs across 24 MHC genes as well as copy number variation (CNV) within class I and class II genes and conduct supertype analysis. Overall, we have provided a bioinformatic workflow for investigating variation in a complex immune gene family from whole genome sequencing data and determined current levels of diversity within koala MHC genes.

**Supplementary Information:**

The online version contains supplementary material available at 10.1007/s00251-024-01356-6.

## Introduction

Our world is more connected than ever, and the threat of zoonotic and emerging infectious diseases continues to increase as humans encroach more on the natural world. This can be seen by the rapid spread of chytridiomycosis around the globe that has severely impacted global amphibian populations (Martel et al. 2014; Scheele et al. 2019). With increasing disease pressures on already under-threat populations, identification of genetic variants that improve an individual’s chance of survival are key. The major histocompatibility complex (MHC) and toll-like receptor (TLR) gene families are of great interest to immunogeneticists as they are involved in two vital aspects of host immunity. The MHC is a highly polymorphic gene family part of the adaptive immune system with the number of loci differing between species as a result of a rapid birth and death evolutionary model (Hughes and Nei 1990; Nei et al. 1997). Genes of the MHC encode molecules which bind and present peptides to T-cells in order to determine self from non-self and triggering an immune reaction if a peptide is determined as non-self. There are two classes of MHC genes involved in peptide binding and presentation, class I genes are present on all nucleated cell types and typically bind to peptides from intracellular pathogens (Bjorkman and Parham 1990; Cresswell et al. 2005), whereas class II genes are only present on antigen presenting cells and bind peptides from exogenous pathogens (Kelley et al. 2005; Neefjes et al. 2011). In contrast, TLR genes are highly conserved across most multicellular organisms and are an ancient part of the innate immune system (Singh et al. 2003). TLR genes encode membrane spanning molecules that are present on the surface of numerous cells and organelles (Akira 2001). TLRs bind to conserved molecules expressed on the surface of pathogens which activates aspects of both the innate and adaptive immune systems (Akira et al. 2001).

Sequencing technologies, assembly algorithms and bioinformatic pipelines are rapidly advancing, enabling researchers to answer biological questions that were unattainable only decades previously. Traditional genomic investigations on non-coding regions on the genome have focused on how populations of species are connected to one another and provided a general overview of the genetic health of a population (Gaughran et al. 2018; Kardos et al. 2021; Waples et al. 2020). It is, however, variation within coding regions of the genome that provides a species with adaptive potential (Allendorf et al. 2010; Eizaguirre and Baltazar-Soares 2014); this variation has typically been assessed with locus specific primers which are effective for investigating diversity within conserved genes. This approach has limitations for complex multicopy gene families such as the MHC (Babik 2010). Most methods to investigate MHC diversity amplify the peptide binding region (PBR), encoded by exon 2 and 3 of the gene (Babik 2010; Cheng et al. 2022, 2018; Hermsen et al. 2017). This methodology may not amplify all loci or alleles intending to be targeted and it is not possible to truly determine the total number of MHC loci within a species (Babik 2010; Lane et al. 2012). The availability of a high-quality (long-read sequencing) reference genome for a species of interest makes it possible to bioinformatically characterise the true number of MHC loci within a species and either design primers to amplify specific loci or use resequencing data to investigate diversity across the genome (Heimeier et al. 2024; Peel et al. 2022). As the use of WGS increases in the field of conservation genomics, it is important to determine whether genotypes in highly polymorphic regions of the genomes can be reliably assessed.

We use koalas as our case study species because there is a high-quality reference genome and accurate immune gene annotation (Johnson et al. 2018; Peel et al. 2022), which made it possible to bioinformatically characterise the entire MHC region. Previous work has identified 11 class I (putatively three classical and eight non-classical) and 14 class II genes (Cheng et al. 2018; Johnson et al. 2018; Lau et al. 2013; Silver et al. 2022). Koalas are impacted by *Chlamydia* and koala retrovirus (KoRV) (Beyer et al. 2018; Cockram and Jackson 1974; Hanger et al. 2000; Polkinghorne et al. 2013). With some populations showing up to 100% of adult koalas are infected with *Chlamydia* (Jackson et al. 1999; Quigley and Timms 2020). In koalas, chlamydiosis is associated with clinical signs in the ocular and urogenital region, including conjunctivitis leading to corneal scarring and blindness, as well as “wet-bottom” and inflammation in the reproductive tract resulting in infertility (Cockram and Jackson 1974; McColl et al. 1984; Polkinghorne et al. 2013). However, not all instances of *Chlamydia* infection result in disease with subclinical infections occurring up to 28% of the time in a south-east QLD population of koalas (Quigley et al. 2018b, 2019). Previous studies on koala MHC and TLR diversity have primarily involved amplicon sequencing of the PBR for MHC and the leucine repeat region, transmembrane and cytoplasmic domains for TLR genes (Cheng et al. 2018; Cui et al. 2015; Lau et al. 2013; Quigley et al. 2018a; Robbins et al. 2020). The study on TLR genes identified a total of 40 SNPs across ten genes (Cui et al. 2015). The primary aim of the MHC studies has been to identify alleles associated with *Chlamydia* susceptibility and identified seven alleles from *β* class II genes and three class I alleles as either increasing susceptibility or resilience to infection (Lau et al. 2014a; Quigley et al. 2018a; Robbins et al. 2020; Silver et al. 2022).

**Fig. 1:**
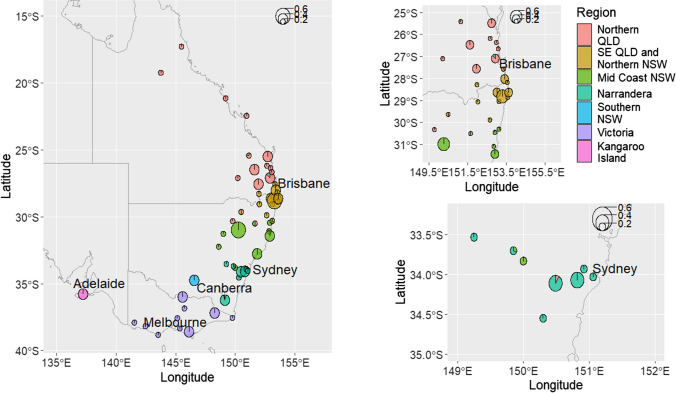
Map showing the locations koala samples were collected from, colours of each pie chart represent the genetic clusters found in each location and the size of the pie chart is representative of the number of samples from a given site with pies of radius 0.2 representing 1–10 samples, 0.4 being 11–20 samples and 0.6 being 21–30 samples

The aims of this study were twofold; firstly, to determine the ideal sequencing depth for genotyping complex gene regions (represented by 25 MHC genes) compared to known single copy, highly conserved genes (represented by ten TLR genes) using available koala target enrichment data (Silver et al. 2022) sequenced to an average of 264 × (59.3–501.2 ×) depth across the targeted regions and ten matched WGS samples (Hogg et al. 2023), sequenced to 47.31 × (42.1–59.3 ×). We then used these results to inform our sampling design for the Koala Genome Survey (Hogg et al. 2023), where we sequenced whole genomes of 438 koalas across the entire geographic range (Fig. 1). Our second aim was to characterise the level genetic variation (both SNPs and copy number) within koala MHC genes across the entire gene length.

## Methods

### Sequencing depth comparison

Sample collection, DNA extraction and sequencing approaches for target enrichment and WGS comparison used in this study have been described previously (Hogg et al. 2023; Robbins et al. 2019, 2020; Silver et al. 2022). Specifically, we used ten samples from a single population in the Moreton Bay Region (MBR; 27.0946° S, 152.9206° E) with DNA extracted from 200 µL of whole blood using the standard MagAttract HMW DNA kit (QIAGEN) protocol. For target enrichment, DNA libraries were prepared using the Kapa Hyperprep Kit (Roche) and DNA was hybridised to RNA baits and sequenced on an Illumina NovaSeq SP 2 × 150 bp flowcell (Illumina) at The Ramaciotti Centre for Genomics (Kensington). For WGS, DNA was sequenced on a Novaseq 6000 S4 flowcell (Illumina) after a TruSeq DNA PCR Free library preparation (Illumina) at The Ramaciotti Centre for Genomics (The University of New South Wales, Kensington, Australia).

For both types of sequence data, raw fastq files were quality checked and aligned to the specific regions of MHC or TLR genes covered by RNA baits in the target capture protocol (Table S1) of the koala reference genome (Johnson et al. 2018) (GCA_002099425.1_phaCin_unsw_v4.1) using Illumina’s Dragen Germline pipeline (v3.8.4) with default parameters. For the WGS data, aligned bam files were then down-sampled using samtools (v1.6) to average sequencing depth of 0.5 × , 1 × , 2 × , 5 × , 10 × , 15 × , 20 × and 30 × (Li and Durbin 2009; Li et al. 2009). For each level of sequencing depth single sample, gvcf files were used as input to Illuminas’ Dragen Joint Genotyping pipeline (v3.9.5) to produce multi-sample vcf files. For TLR genes, vcftools (v0.1.14) (Danecek et al. 2011) was used to retain only biallelic SNPs within TLR exons. GATK (v4.2.0.0) (McKenna et al. 2010) VariantFiltration and SelectVariants were used to filter sites with quality less than 80 and mapping quality less than 40. Bcftools (v1.3.1) (Danecek et al. 2021; Li 2011) and vcftools (v0.1.14) (Danecek et al. 2011) were used to calculate depth for each genotype and remove sites with an average depth less than 1/3 the target sequencing depth up to 10 × (i.e. for a target sequencing depth of 15 × , sites with average depth less than 5 were removed). For MHC genes, vcftools (v0.1.14) was used to retain only biallelic SNPs within MHC exons. GATK (v4.2.0.0) (McKenna et al. 2010) VariantFiltration and SelectVariants were used to filter sites with quality less than 80, mapping quality less than 40 MQRankSum greater than 12.5 or less than − 12.5 and ReadPosRankSum greater than 8 or less than − 8. Bcftools (v1.3.1) (Danecek et al. 2021; Li 2011) and vcftools (v0.1.14) were used to calculate depth for each genotype and remove sites with an average depth less than 1/3 the target sequencing depth up to 10 × (i.e. for a target sequencing depth of 15 × , sites with average depth less than 5 were removed). Bcftools (v1.3.1) and vcftools (v0.1.14) (Danecek et al. 2021; Li 2011) were also used to calculate allelic balance and remove sites with a difference of greater than 90%.

To assess the concordance of variants called between target enrichment and whole genome data, we first identified which variant sites were present in both data types for both MHC and TLR gene families. Secondly, for each genotype call in each individual a letter of the alphabet was assigned (e.g. ‘A’ was assigned when both the target enrichment and WGS variant for an individual was a homozygote for the reference allele; see Table S2 for alphabet letter allocations). Concordance was determined by the number of matching genotype calls between target enrichment and WGS compared to non-matching variant calls. A value for concordance (when the genotype in an individual was the same with both target enrichment and WGS), missing WGS calls (when a genotype was called in an individual with target enrichment but not called in the same individual with WGS), missing target enrichment call (when a genotype was called in an individual with WGS but not called in the same individual with target enrichment) and non-concordant genotype calls (when the genotype in an individual was different between target enrichment and WGS) was calculated as below.

$$\text{Concordance}=\frac{\sum A+\sum F+\sum K+ \sum P}{\sum A+\sum B+\sum C+\sum D+\sum E+\sum F+\sum G+\sum H+\sum I+\sum J+\sum K+\sum L+\sum M+\sum N+\sum O+\sum P}$$

$$\text{Missing whole genome calls}=\frac{\sum M+\sum N+\sum O}{\sum A+\sum B+\sum C+\sum D+\sum E+\sum F+\sum G+\sum H+\sum I+\sum J+\sum K+\sum L+\sum M+\sum N+\sum O+\sum P}$$

$$\text{Missing Target enrichment calls}=\frac{\sum D+\sum H+L}{\sum A+\sum B+\sum C+\sum D+\sum E+\sum F+\sum G+\sum H+\sum I+\sum J+\sum K+\sum L+\sum M+\sum N+\sum O+\sum P}$$

$$\text{Non}-\text{concordance}=\frac{\sum B+\sum C+\sum E+ \sum G + \sum I+ \sum J}{\sum A+\sum B+\sum C+\sum D+\sum E+\sum F+\sum G+\sum H+\sum I+\sum J+\sum K+\sum L+\sum M+\sum N+\sum O+\sum P}$$

Additionally, we wanted to determine whether we would be able to detect structural variants in the form of MHC copy number variation (CNV) in our WGS dataset. We used differences in sequencing depth to identify putative CNV in MHC genes. For each of the ten samples at each of the eight down-sampled sequencing depths and the full dataset, we used the samtools coverage (v1.14) (Li et al. 2009) to determine the number of reads spanning each MHC and TLR gene as well as the number of bases covered by reads in each gene. The number of reads was then divided by the gene length in kb to determine the numbers of reads per kilobase (RPK). To identify potential copy number variations, the RPK value for each MHC gene was divided by the average RPK value for the eight TLR genes and multiplied by two to give an allelic copy number (with a value of two indicating a single copy of a particular gene). The allelic copy numbers were plotted using the ggplot2 package (Wickham 2016) in R (v4.2.1) (R Core Team 2024) to allow for comparison and estimation of copy number differences between individuals. To identify putative copy number variants, any individual with an allelic copy number value between 1.5 and 2.5 were said to have a single copy of the gene (Bidon et al. 2015).

### WGS range wide diversity

From our sequencing depth comparison (see “Results”), we determined that a target of 30 × sequencing depth provided the best quality of data for the most value for money. For our range wide diversity investigation we used 418 koala WGS from Hogg et al. (2023) (we excluded 12 captive samples as we were solely interested in wild diversity) and an additional 20 samples from Kangaroo Island. We performed either a MagAttract HMW DNA kit (Qiagen, Germany; cat: 67,563), or a high salt method (following a modified protocol from Aljanabi and Martinez (1997)) extraction with samples sequenced on Novaseq 6000 S4 flowcell (Illumina) after a TruSeq DNA PCR Free library preparation (Illumina) at The Ramaciotti Centre for Genomics. An initial sequencing run of 24 samples were pooled across one lane of a S4 200 cycle flowcell and sequencing depth assessed. The initial sequencing yielded uneven sequencing depth across sampling due to varying DNA quality and library pooling. As a result, we undertook an additional DNA repair step using a FFPE DNA repair protocol (New England Biosciences) (see Hogg et al. (2023) for details). Further sequencing runs took place by pooling 48 samples across one lane of a S4 200 cycle flowcell with sequencing depth assessed following the completion of the sequencing and pooling adjusted accordingly to meet the 30 × sequencing depth goal.

Raw fastq files were quality checked and aligned to the koala reference genome (GCA_002099425.1_phaCin_unsw_v4.1) (Johnson et al. 2018) using the Dragen Platform (v.3.8.4, Illumina San Diego) on Illumina’s Basespace portal. Following each sequencing run, raw and aligned data files (fastq and BAM) were publicly released on the Amazon Web Services (AWS) Open Data program (https://awgg-lab.github.io/australasiangenomes/species/Phascolarctos_cinereus.html).

Once sequencing of all samples had been completed, we used the Dragen gVCF genotyper in the non-iterative mode on a Dragen V4 server to generate a multi sample vcf file. Following this, we used the Dragen Joint Genotyping Pipeline (v3.9.5) on Illumina’s Basespace portal to run joint genotype calling on the multi sample vcf file. To investigate MHC diversity within the koala population, we first filtered the whole genome joint genotyped multi-sample vcf file to include only biallelic SNPs found within MHC exons (Table S1) using vcftools (v0.1.14) (Danecek et al. 2011) and gatk SelectVariants (v4.2.0.0) (McKenna et al. 2010). Further filtering of variants then occurred as described above.

For all, analysis samples were divided into seven regions (five reflective of the genetic clusters determined by McLennan et al. (2024*)*, Kangaroo Island and Narrandera [Narrandera was included as a separate region as there are reports that these koalas originated from mixing individuals from Victoria and another unknown location (Sullivan 1990)], Fig. 1)*.* To assess genetic differentiation between individuals, we generated PCoA plots using our filtered MHC variants using adegenet (Jombart 2008) in R (v4.1.1) (R Core Team 2024). We performed PCoA analysis separately, one with genes present in single copy and one with genes with CNV (see results below). To determine the correlation between genetic distance and geographical distance, we conducted a Mantel test using the dartR package for R (Gruber et al. 2018). We then phased SNPs by first using gatk FastaAlternateReferenceMaker (v4.2.0.0) (McKenna et al. 2010) to convert the multi-sample vcf file to a single sample consensus fasta sequences for each gene investigated. SeqPHASE (Flot 2010) was used to convert the fasta sequences into phase format then PHASE (v.2.1.1) (Stephens and Scheet 2005; Stephens et al. 2001) and was run to generate alleles to investigate diversity across the koala range. SeqPHASE (Flot 2010) was used to convert PHASE output into phases fasta sequences to give 900 sequences for each gene (two sequences per individual).

Any sequence with unresolved alleles was then removed and data imported into DNA Sequence Polymorphism v6.12.03 (Rozas et al. 2017) to label alleles and calculate diversity statistics including nucleotide diversity and allele diversity (Nei 1987). Prior to diversity metrics being calculated, any alleles that occurred at a frequency less than 0.005 (four occurrences or less) were removed. Allelic frequency for genes present in single copy was visualised using ggplot2 (Wickham 2016) in R (v.4.1.1). PCoA plots based on allele assignment was conducted as described above. Weir and Cockerham’s *F*_ST_ (Weir and Cockerham 1984) was calculated using vcftools v0.1.14 (Danecek et al. 2011) between each cluster.

Non-synonymous variations have functional consequences and these amino acid substitutions may impact an MHC molecule’s antigen binding ability. It is predicted that MHC supertypes can bind pathogens with similar antigenic profiles. We attempted to cluster class I alleles into supertypes by first retaining only amino acids predicted to be involved in peptide binding as determined by Cheng et al. (2018). Then, substituting each amino acid for five *z* scores represent a range of physiochemical properties as per Sandberg et al. (1998). We then used DAPC clustering as part of the adegenet (Jombart 2008) package in R by first using 1000 repeats, through the *replicate()* function, of *find.clusters()* with the following parameters; n.pca = 30, max.n.clust = 30, n.iter = 5e5, n.start = 500. We then identified the best *k* based on the most common occurrence of minimum Bayesian information criterion (BIC). We then performed discriminant analysis of principal components (DAPC) retaining 15 principal components (PC) and all discriminant functions. We then ran *optim.a.score()* to determine the most appropriate number of PCs to retain and then a second iterations of *dapc()* with the number of PCs as chosen by *optim.a.score()*. We then extracted the clusters each amino acid sequences were assigned to and assigned supertypes to each individual. PCoA plots based on supertype assignment was conducted as described above.

Copy number variation (CNV) detection was performed by investigating sequencing depth differences between MHC genes and TLR genes as described above with CNVs visualised using a violin and scatter plot using ggplot2 (Wickham 2016) in R (v4.1.1) (R Core Team 2024) to identify regions and genes with CNV. In order to determine if using a read depth approach to identifying CNV in koala MHC is appropriate, we constructed neighbour joining trees with 1000 bootstrapping replicated using the best model predicted in MEGA11 for MHCI (Tamura 3-parameter + gamma distribution), MHCII A (Kumara 2-parameter + gamma distribution) and MHCII B (Kumara 2-parameter + gamma distribution) alleles identified in this study. These results showed that alleles of the same gene are more similar to each other than to alleles of different MHC genes (Figure S1-S3), additionally the percentage of similarity between MHC genes is sufficiently low that reads map uniquely to the correct locus (Cheng et al. 2018).

## Results

### Sequencing depth comparison

For MHC genes, target enrichment identified 269 variants and due to the high average sequencing depth of the target enrichment samples we designate these as “true” calls. The full (> 40 × sequencing depth) whole genome data identified 262 variant sites and the down-sampled whole genomes found between 49 (0.5 × sequencing depth) and 272 variant sites (20 × sequencing depth). For TLR genes, we identified 26 variants in the target enrichment data, and due to the high average sequencing depth of the target enrichment samples, we designate these as “true” calls. Down-sampled WGS identified between 10 (0.5 × sequencing depth) and 26 (≥ 15 × sequencing depth) variants. For both gene families, at a low-sequencing depth (0.5 × –2 ×) far more variants were not identified in the whole genome data set (Figure S4). As sequencing depth increased, more variants present in the “true” dataset were identified in the whole genome dataset (Figure S4). In MHC genes, the percentage of concordance plateaus slightly at a ≥ 15 × sequencing depth; although, the reliability continued to increase with increasing sequencing depth whilst in TLR genes it plateaus at a ≥ 10 × sequencing depth (Figure S5). Interestingly, there were additional MHC variants identified in the whole genome data that were not present in the target capture (28 MHC variants present only in the full whole genome dataset) (Figure S4A). This trend was not observed in the TLR genes, and at a 15 × sequencing depth and above, all 26 variants were present in both datasets (Figure S4B). As expected, as the sequencing depth of the whole genome data increased there was higher overlap between the variants identified through WGS and target enrichment (Figure S4). Additionally, as the sequencing depth increased there was higher concordance of overlapping variant calls (Figure S5). At a low-sequencing depth (0.5–2 ×), the greatest source of non-concordant calls was due to missing data, with 66% and 63% of overlapping variant sites missing genotype calls in MHC and TLR genes, respectively, at a 0.5 × sequencing depth (Figure S5). Overall, concordance was similar between target enrichment and WGS in the TLR genes compared to MHC genes (Figure S5).

From our copy number analysis, we able to identify CNV at sequencing depth of 1 × (a deletion in DBA2); however, as sequencing depth increases (> 10 ×), it becomes possible to identify hemizygous variations and more reliably identify duplication of genes (Figure S6, S7), with the best results occurring with sequencing depth 30 × or above.

### WGS range wide diversity

**Fig. 2:**
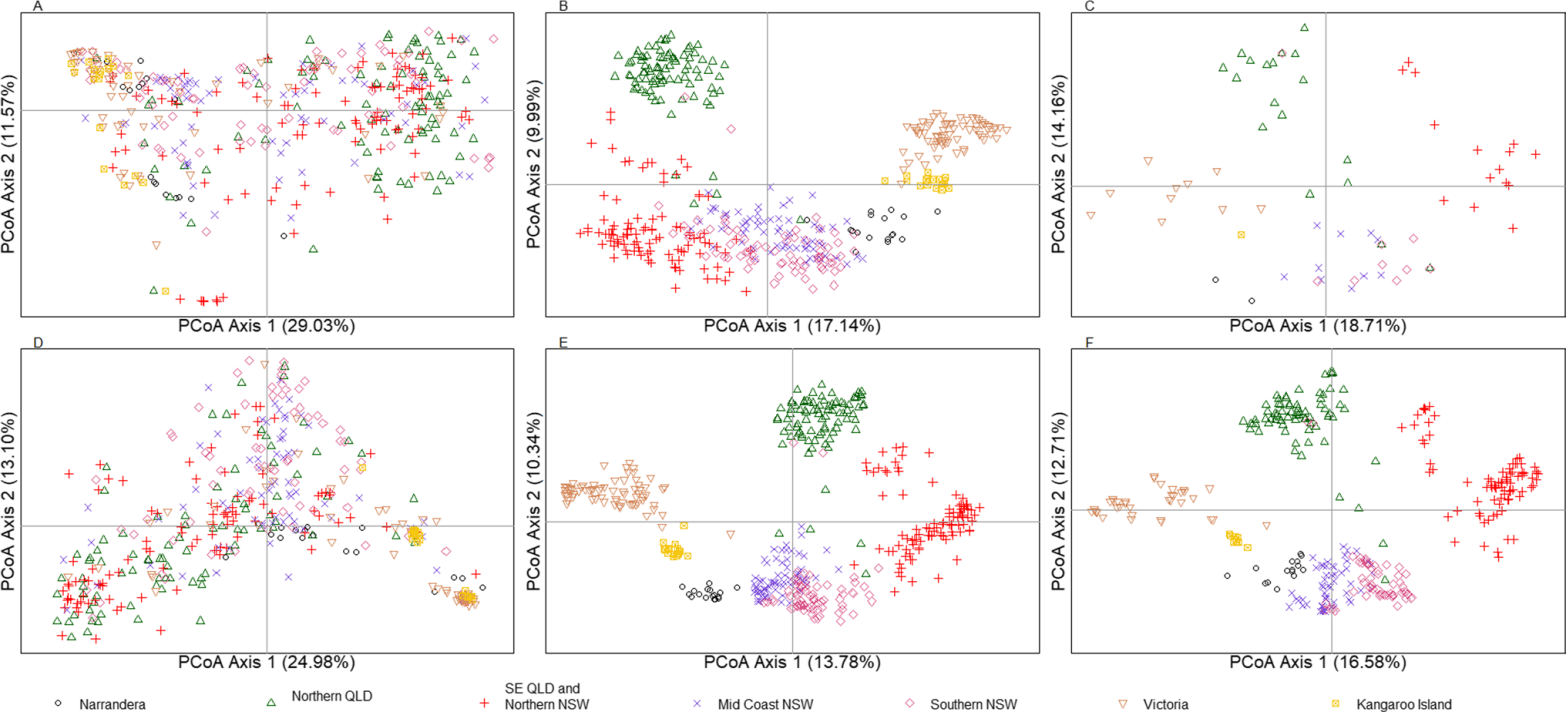
Principal component analysis plots of 438 wild koalas based on SNPs (**A**, **D**), alleles (**B**, **E**) and supertypes (**C**, **F**) with individuals coloured and given a separate symbol according to region from which they originate. Different plots were produced for genes present in single copy (**A**, **B**, **C**) and genes which are duplicated (**D**, **E**, **F**) in koala populations

A total of 438 samples were sequenced across 53 wild locations with an average sequencing depth 32.25 × (range, 11.3–66.8 ×) and the multi-sample vcf file containing all sequenced koalas consisted of 48,695,015 variants. Following identification and filtering of MHC variants, we located 270 biallelic SNPs in 438 koalas, including 144 in class I and 126 in class II genes with only one gene (DAB1) being monomorphic. Out of the 270 variants, 164 are predicted to be amino acid altering, with non-synonymous variation present in 21 genes. UA, UH and DAB3 all had more than 20 non-synonymous variants, and UA also had the most alleles with 27. Across the 24 MHC genes with variants detected, we identified 180 alleles including 75 class I and 105 class II (Table 1, Table S3, Additional Data 1). Overall, the 24 genes with variants had an average of 11.25 exonic SNPs (range, 1–53), 7.5 alleles (range, 2–27) and 6.8 non-synonymous SNPs (range, 0–36) (Table 1). On average, class I genes had higher numbers of exonic SNPs, non-synonymous SNPs and fewer alleles compared to class II genes. The higher numbers of alleles identified in class II genes compared to class I genes may be due to five class II genes having CNV, compared to four class I genes having CNV and also there are more class II genes (14) compared to class I genes (11). The highest nucleotide diversity was seen in UH and UA had the highest allelic diversity (Table 1). Diversity results are consistent with genes that have CNV across the koala range having more alleles and higher levels of genetic diversity. Exploratory population analysis using SNPs in MHC genes indicate homogenous genetic structuring with minimal structuring between populations found in NSW and QLD (Fig. 2). There is tight clustering of individuals sequenced from Victoria and Kangaroo Island that is also supported by extremely low population differentiation (as measured by *F*_ST_; 0.029: Table 2). Similarly, a low differentiation is seen between northern QLD and south-east QLD and northern NSW (*F*_ST_ = 0.051), as well as mid-coast NSW and southern NSW koalas (*F*_ST_ = 0.063). Between wild populations, the highest level of differentiation is seen between northern Queensland and Victorian koalas (*F*_ST_ = 0.318; Table 2). When looking at population structuring through alleles, we can see overlapping clusters according to regions defined from the genome-wide variation (McLennan et al., 2024), with individuals from northern QLD forming a distinct group, koalas from NSW and south-east QLD forming another cluster and Victoria and Kangaroo Island koalas forming two additional clusters (Fig. 2). In our PCoAs using allele and supertype data, koalas from Narrandera sit between Victorian individuals and NSW individuals, supportive of the anecdotal evidence of this population being formed by mixing Victorian koalas and koalas from other regions (Fig. 2) (Sullivan 1990). As anticipated, the Mantel test showed significant correlation between geographical distance and genetic distance (*r* = 0.8461, *p* = 0.002).

**Table 1:** Diversity statistics for each MHC gene investigated including number of non-synonymous SNPs, number of alleles and nucleotide and allelic diversity. Nucleotide and allelic diversity were calculated in DNAsp v.6.12.03 (Nei 1987; Rozas et al. 2017)

Gene	*N*	SNPs	SNPs (ns)	*π*	No. alleles	Allelic diversity
MHCI1 (UI)	876	1	1	0.00045	2	0.483
MHCI2 (UD)	876	1	0	0.00036	2	0.37
MHCI4 (UA)*	611	53	36	0.01914	27	0.888
MHCI5 (UK)	874	4	4	0.00121	2	0.33
MHCI8 (UC)	822	2	1	0.00068	4	0.603
MHCI9 (UE)	814	4	2	0.00128	6	0.703
MHCI10 (UF)	876	2	1	0.00054	3	0.524
MHCI12 (UH)*	363	50	21	0.01948	13	0.787
MHCI13 (UG)*	825	15	12	0.00267	7	0.602
MHCI15 (UJ)	875	1	0	0.00052	2	0.499
MHCI19 (UB)*	785	10	4	0.00333	7	0.802
DAA	876	5	0	0.00313	4	0.569
DAB2*	611	12	7	0.00682	18	0.869
DAB3*	599	27	20	0.01521	25	0.821
DAB4	875	3	1	0.00149	3	0.407
DAB5	876	2	2	0.00081	3	0.589
DBA1	812	18	12	0.00793	11	0.738
DBA2*	835	23	17	0.01052	8	0.569
DBB2*	869	5	4	0.00148	2	0.245
DBB3*	840	12	7	0.00462	12	0.732
DCA	873	8	4	0.00347	6	0.757
DCB	876	5	4	0.00173	5	0.712
DMA	876	3	1	0.00134	3	0.598
DMB	768	3	3	0.00141	5	0.764

*N*, number of sequences; π, nucleotide diversity

^*^Indicates gene has copy number variation

**Table 2:** Mean weighted Weir and Cockerham’s *F*_ST_ values at 270 MHC SNPs between 7 regions as determined by McLennan et al. (2024)

	Northern QLD	South-east QLD and northern NSW	Mid-coast NSW	Narrandera	Southern NSW	Victoria
South-east QLD and northern NSW	0.051161					
Mid-coast NSW	0.12434	0.062581				
Narrandera	0.18975	0.11861	0.070785			
Southern NSW	0.15066	0.10217	0.029405	0.10271		
Victoria	0.31849	0.26508	0.19068	0.1217	0.18289	
Kangaroo Island	0.27092	0.21625	0.15857	0.10518	0.14573	0.029027

**Fig. 3:**
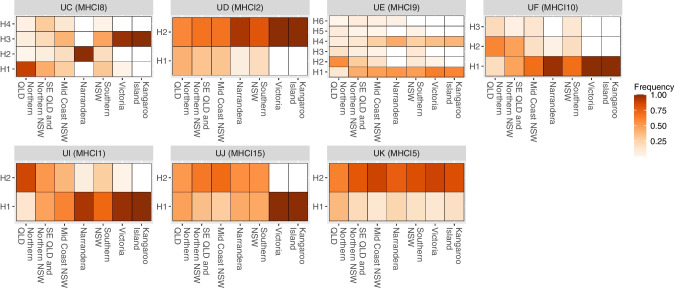
Heatmap of MHC class I allele frequencies. The *x*-axis designates each region koalas were sampled, and the *y*-axis represents each allele as assigned during phasing. Lighter colours represent lower frequency of a given allele and dark colours represent high frequency, if an allele was not seen in a population it is represented by white

**Fig. 4:**
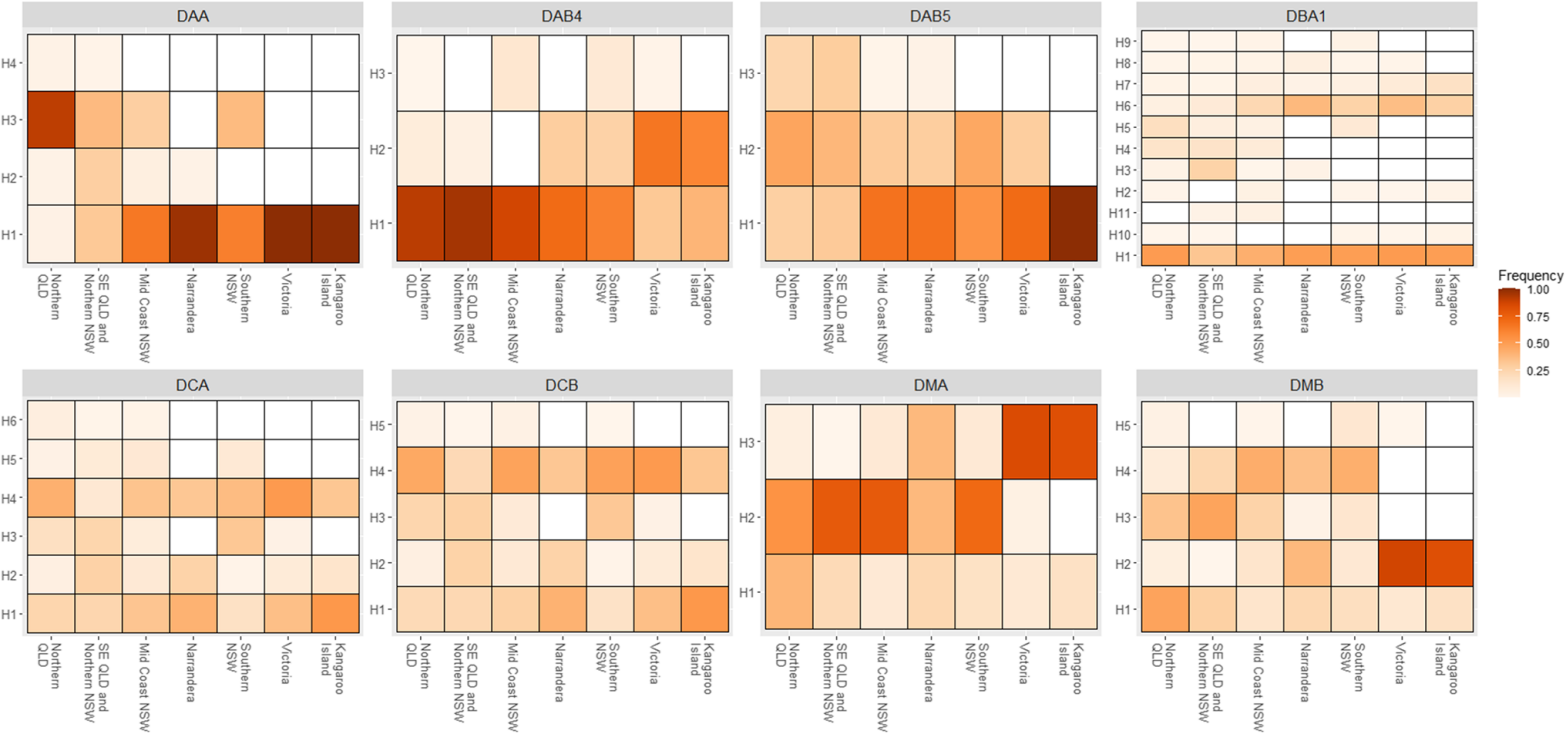
Heatmap of allele frequencies of class II MHC genes. The *x*-axis designates each region koalas were sampled, and the *y*-axis represents each allele as assigned during phasing. Lighter colours represent lower frequency of a given allele and dark colours represent high frequency, if an allele was not seen in a population it is represented by white

For many genes, allele frequencies are similar across regions, often with Victorian and South Australian koalas having higher frequency of the most common allele (Fig. 3, Fig. 4). The lower diversity in Kangaroo Island and Victoria is shown by these populations being dominated by a single allele for four out of eight single copy class I and five out of seven single copy class II genes. There are some genes where the most common allele differs between regions, for example DMA where H3 is the most common in Victoria and Kangaroo Island with far higher occurrence of this allele compared to the most common allele in other regions (Fig. 3). At DAB5 and DMB, regions in NSW and QLD show high allelic diversity with each allele seen at low frequency in the population, whereas Victoria and Kangaroo Island have one allele present in high frequency (Fig. 4). A slightly contrasting pattern is seen at DAB4 where regions in NSW and QLD have a high frequency of H1, whereas Kangaroo Island and Victoria show higher diversity (Fig. 4).

Using a cluster analysis to identify supertypes in class I genes, we determined an optimal number of clusters to be of 28 based on minimum BIC. We identified a total of 28 supertypes in koala class I MHC genes with each gene being represented by a single supertype apart from UA (13 supertypes), UB (3 supertypes) and UG (4 supertypes) (Table S4). Interestingly, all genes represented with multiple supertypes are genes which show CNV. When investigating population structuring based on supertypes, a far more regional differentiation is seen compared to PCoAs constructed with SNPs, but similar differentiation as seen with alleles (Fig. 2). Most likely due to functional redundancy built into the genetic code, there are 64 combinations of codon sequences which translate to 20 amino acids. Additionally, each amino acids shares physiochemical properties with other amino acids, thus further increasing redundancy in the genetic code. We also see similar patterns when conducing PCoA on genes present in single copy and multicopy, suggesting similar pressures are acting on both single copy and multicopy genes. Each regions forms a distinct cluster with mixing only observed between mid-coast NSW and southern NSW koalas (Fig. 2).

**Fig. 5:**
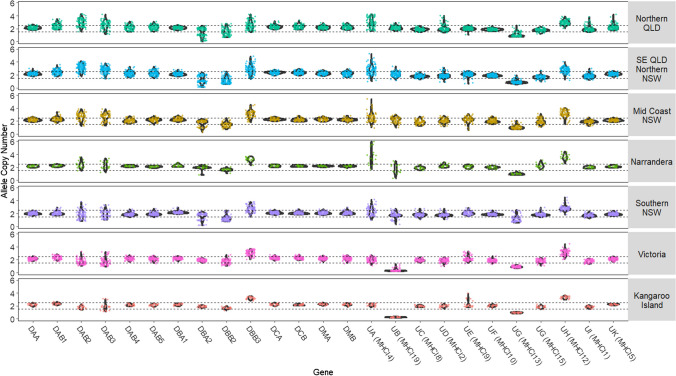
Violin plot of predicted allele copy number for MHC genes. Each dot is representative of one individual with colours representing populations. Dotted horizontal lines at 1.5 and 2.5 indicate bounds of single copy genes. Plots are divided into seven wild regions

Our phylogenetic analysis of alleles showed good support for single clusters of each gene, particularly for MHCI where all alleles a present in a single cluster for each gene (Figure S1). For both alpha and beta loci of class II genes, there is some interspersing of alleles between genes, in particular the DBA and DAB loci (Figure S2, S3). Investigation of sequencing depth across MHC genes revealed differences in sequencing depth between genes and individuals. We identified sequencing depth difference in four class I genes (UA, UB, UG and UH) (Fig. 5) and five class II genes (DAB2, DAB3, DBA2, DBB2 and DBB3) (Fig. 5). We found potential duplications in UA with the sequencing depth difference suggesting some individuals contain up to six allele copies, particularly in SE QLD and northern NSW populations (Fig. 5). We find complete gene deletions in UB in individuals from Kangaroo Island and Victoria and single copy hemizygous deletions of UG in all regions (Fig. 5). Most interestingly, we identify within the same populations a copy number variation in DBA2 with complete deletion, single copy deletion and two alleles seen in Queensland and New South Wales koalas (Fig. 5).

## Discussion

With species under increasing threat from the combined threats of disease and declining genetic diversity, genomic and computational technologies provide a powerful tool to improve the conservation of vulnerable species and populations (Hogg et al. 2022). The increased utility of WGS in conversation programs warrants investigation into the accuracy of genotype calls across a range of sequencing depths in functional regions of the genome. Here, we have undertaken a quantitative assessment that will assist researchers to develop sound sampling design and select methods appropriate for their study questions and their funding resources available.

This study investigated whether high-sequencing depth WGS and a bioinformatic workflow could be used to accurately genotype SNPs and CNV within a complex immune gene family, the MHC. We were able to accurately genotype SNPs and identify alleles within MHC genes using a range of bioinformatic software and a custom script, the results from which we then used to inform our study design for the Koala Genome Survey, ensuring our sequencing depth was high enough to be able to investigate MHC diversity.

Our results indicate that both high coverage WGS and target enrichment methods are appropriate for studying variation in polymorphic, duplicated gene families of non-model populations. The nucleotide sequence similarity between regions of interest may also impact the ability to reliably call genotypes. In koalas, where class I genes show similarities between 63 and 95% (Cheng et al. 2018), there were some regions where reads did not align. This is important as some genetically depauperate species such as Tasmanian devils (*Sarcophilus harrisii*) show sequence similarity as high as 97% between MHC genes (Cheng et al. 2012). However, if researchers are solely interested in the MHC region, a long-read sequencing method more specific to the MHC may be preferred, especially to more thoroughly investigate class I genes (Cheng et al. 2022). For example, in Tasmanian devils, a custom approach utilising PacBio amplicon sequencing to identify previously unknown diversity and identified 50 haplotypes spanning all MHC class I genes, assigned with > 99% confidence (Cheng et al. 2022). Approaches using Oxford Nanopore amplicon sequencing have also been used to assemble the entire DRB locus in a population of Alpine chamois (*Rupicapra rupicapra*) (Fuselli et al. 2018). These approaches offer exciting opportunities to address some of the shortcomings of this paper, such as being able to separate out the different alleles within genes which show CNV and to more reliably assign alleles to all individuals. As in this study, even with a 30 × coverage WGS, it is difficult to reliably phase variants and identify alleles, with ~ 10% of individuals unable to have alleles assigned in our koala WGS dataset (Table 2). Excitingly, these technologies also allow for haplotype identification across multiple MHC genes (Ammar et al. 2015; Cheng et al. 2022; Hu et al. 2022). However, due to the high cost of long-read sequencing and laboratory intensive methods, this MHC typing method may be limited to small numbers of genes and individuals. Therefore, as new genomic technologies become accessible for researchers working on species of conservation concern, it remains imperative that there is consideration for which technology is the most appropriate and suited for answering the key conservation and research questions for that species (Fuentes-Pardo and Ruzzante 2017; McLennan et al. 2019).

We used a bioinformatic approach to characterise the levels of diversity of koala MHC genes from WGS data. To date, this is the widest ranging study on koala immune genes, with previous studies focusing on either a small number of MHC genes or koalas from few populations (Lau et al. 2014a, 2014b, 2013; Quigley et al. 2018a; Robbins et al. 2020; Silver et al. 2022). We characterised diversity in 24 MHC genes from 438 koalas from 54 wild locations across four states representing the entire koala range and identified 270 SNPs, of which 164 resulted in non-synonymous variation. Representing a large body of knowledge to provide a baseline level of MHC diversity in koalas, future studies can compare back to. By using the koala reference genome (Johnson et al. 2018) and WGS, we have been able to identify the complete MHC repertoire of koalas and through a comparison of sequencing depth identified nine genes with CNV. This is the first time CNV has been detected in koalas with most previous MHC studies occurring without alignment to a reference genome and only having estimates of the potential number of MHC loci in koalas (Lau et al. 2014b; Quigley et al. 2018a; Robbins et al. 2020). Whilst these previous studies have investigated some of the same genes (UA, UC, DAB, DBB, DCB and DMB), they utilised the technology current at the time, which only investigated exon 2 of each gene, compared to this study which has looked across the complete length of the gene. Additionally, as these methods occurred without a reference genome, it was not possible to assign alleles to specific loci. Typical methods of investigating MHC diversity through species, specific primers and amplicon sequencing allow identification of SNPs but are limited in their ability to detect CNV due to the lack of locus specific information. The number of MHC loci is often estimated by dividing the number of unique alleles identified in an individual by two (this assumes each locus is heterozygous and therefore contains two unique alleles) (Minias et al. 2019, 2021). Our results are supportive of the conclusions of Lau et al. (2014b) in that there are fewer copy numbers of MHC genes in Victorian populations as shown in this study by the deletion of the UB gene and hemizygosity of UG (Fig. 5). It appears that severe population bottlenecking (Menkhorst 2008) has resulted in a reduction in diversity at both coding and non-coding regions of the genome (Cristescu et al. 2009; Kjeldsen et al. 2015).

We identified two types of genetic variation within MHC genes, SNPs and CNVs (Stervander et al. 2020). Victorian koalas have a lower level of diversity with an average of 1.19 alleles in an individual at each class I gene compared to an average of 1.47 in NSW and QLD populations. Although in contrast to what is generally accepted, where it is assumed that higher diversity results in higher fitness, it appears Victorian and Kangaroo Island koala populations are relatively healthy with increasing population growth and very few Victorian individuals suffering clinical signs of *C. pecorum* infection and Kangaroo Island is currently *C. pecorum* free (Fabijan et al. 2019; Speight et al. 2016). Another potential major driver of genetic differentiation across the koala range is the impact of KoRV. This is because it is thought that KoRV has become endogenous (incorporated into the genome sequence) in koalas in northern NSW and QLD, whereas is still transmitted through infection in koalas in Victoria and SA (Quigley and Timms 2020). To date, no studies have been able to identify a link between specific MHC alleles or expression levels and KoRv; however, there is some research suggest an impact on cytokine expression with KoRV infection which should be investigated further (Maher and Higgins 2016; Maher et al. 2019).

Another way to classify MHC diversity is through supertype clustering. Supertypes are alleles that share similar biochemical properties (Sette and Sidney 1999; Sidney et al. 1996). We clustered 75 class I alleles into 28 supertypes, which is the first supertype identification carried out in koalas. We find distinct supertype clustering of koalas from each region suggesting pathogen induced selective pressures on MHC genes vary widely across the range of koalas. Future work should identify the potential pathogens that can be bound by these identified supertypes.

Results from Narrandera are interesting, as anecdotally this population is a mixture of translocated koalas from French Island, in Victoria and another unknown location (Sullivan 1990). Recent neutral genomic data supports this hypothesis (McLennan et al. 2024). From our PCoA plots, we see that individuals from Narrandera form an intermediate group between Victoria and NSW/QLD populations, suggesting that genetic mixing has occurred between the founder individuals and diversity has been retained both across the genome and in functional regions. Additional evidence that mixing genetically differentiated populations increases diversity can be seen in our CNV analysis. Victorian koalas have a deletion of UB, but koalas from Narrandera have two alleles per individual. In marsupials, CNVs have been detected in class I genes in Tasmanian devils, and it has been proposed that devils rely primarily on CNV for MHC diversity (Cheng et al. 2012; Siddle et al. 2010). Genetic mixing has also been seen in Tasmanian devils at neutral regions of the genome (McLennan et al. 2020), and also in a critically small population of adders (*Vipera berus*) diversity in class I MHC genes increased as a result of mixing with a genetically distinct population (Roca et al. 2018). Further support for mixing genetically distinct populations to increase diversity has been shown in two populations of inbred New Zealand South Island robins (*Petroica australis*) (Grueber et al. 2017). To better ascertain the outcome of mixing genetically distinct koala populations, functional diversity at more genomic regions and overall fitness of individuals should be assessed.

Another exciting avenue of research is the diversity and evolutionary importance of structural variants (Wold et al. 2021). There are numerous challenges that are currently limiting the ability to investigate structural variation within non-model organisms including the difficulties of short-read data identifying genomic insertions (Delage et al. 2020; Pokrovac and Pezer 2022; Wold et al. 2023). One promising approach which could be feasible for koalas is the generation of a pangenome. By selecting a small number of individuals from across the entire koala range to perform a long-read sequencing on it would be possible to identify a comprehensive reference set of structural variants for koalas which could be used to improve performance of structural variant detection tools from short-read data (Ebler et al. 2022; Nguyen et al. 2023).

In conclusion, by providing a baseline measure of standing functional diversity across the entire geographic range of koalas, we have provided a resource that can be referred to in the future as populations of koalas continue to be impacted by anthropogenic climate change and disease threats. More generally, we have provided a bioinformatic workflow for investigating genetic diversity in any gene family.

## Supplementary information

Below is the link to the electronic supplementary material.

Supplementary file1 (DOCX 9683 KB)

Supplementary file2 (XLSX 97 KB)

Supplementary file3 (XLSX 57 KB)

## Data Availability

All raw fastq sequences, aligned bam files and metadata for koala genomes from the Koala Genome Survey and target enrichment sequencing are available at https://awgg-lab.github.io/australasiangenomes/species/Phascolarctos_cinereus.html and data from the target enrichment study in the folder ‘Investigating_immune_genes_of_the_iconic_koala’ and whole genome sequence for individuals in this study in the folder ‘QLD_Moreton_Bay_Region’. By providing these data online as the data was generated, researchers from around the world can access and work on koala genomics in real time. Raw data files for koala genomes are available on NCBI under BioProject PRJNA940526. Genomic coordinates for MHC and TLR genes used in this study are available in Supplementary Table 1 and all fasta sequences of MHC alleles identified in this study are available in Additional Data 1 in the supplementary material.
