# Supplementary material for: Using bioinformatics to investigate functional diversity: a case study of MHC diversity in koalas
Source: Immunogenetics. 2024 Oct 5;76(5-6):381–95. doi: 10.1007/s00251-024-01356-6 (PMC11496358; doi:10.1007/s00251-024-01356-6)
Supplement: Supplementary file 1 — Supplementary file1 (DOCX 9683 KB) [file 251_2024_1356_MOESM1_ESM.docx]

# Supplementary for: Using bioinformatics to investigate functional diversity: A case study of MHC diversity in koalas

Luke W. Silver^1,2^, Elspeth A. McLennan^1^, Julian Beaman^3^, Karen Burke da Silva^3^, Peter Timms^4^, Carolyn J. Hogg^1,2^, Katherine Belov^1,2^

^1^School of Life and Environmental Sciences, The University of Sydney, Camperdown, NSW, 2006, Australia.

^2^Australian Research Council Centre of Excellence for Innovations in Peptide and Protein Science, University of Sydney, Camperdown, NSW 2006, Australia

^3^College of Science and Engineering, Flinders University, Bedford Park, South Australia, 5001, Australia

^4^Genecology Research Centre, University of the Sunshine Coast, Sippy Downs, QLD, 4556, Australia

Corresponding Author: Carolyn Hogg, School of Life and Environmental Sciences, The University of Sydney, New South Wales, 2006, Australia.

Email: [Carolyn.hogg@sydney.edu.au](mailto:Carolyn.hogg@sydney.edu.au)

## Supplementary Tables

Supplementary Table 1: Start and End coordinates of each exon and gene for 11 class I and 14 class II MHC genes and 9 TLR genes

| Scaffold | Start | End | Name | Covered by RNA bait (Y/N) | Gene Feature | Strand |
| --- | --- | --- | --- | --- | --- | --- |
| MSTS01000255 | 1210001 | 1214547 | phci_DAA_1 |  | Gene | - |
| MSTS01000255 | 1214465 | 1214547 | phci_DAA_1_exon1 | Y | Exon | - |
| MSTS01000255 | 1211210 | 1211456 | phci_DAA_1_exon2 | Y | Exon | - |
| MSTS01000255 | 1210439 | 1210721 | phci_DAA_1_exon3 | Y | Exon | - |
| MSTS01000255 | 1210001 | 1210162 | phci_DAA_1_exon4 | Y | Exon | - |
| MSTS01000401 | 78815 | 85339 | phci_DAB_1 |  | Gene | + |
| MSTS01000401 | 78815 | 78897 | phci_DAB_1_exon1 | Y | Exon | + |
| MSTS01000401 | 83889 | 84153 | phci_DAB_1_exon2 | Y | Exon | + |
| MSTS01000401 | 84544 | 84826 | phci_DAB_1_exon3 | Y | Exon | + |
| MSTS01000401 | 85202 | 85339 | phci_DAB_1_exon4 | Y | Exon | + |
| MSTS01000401 | 145117 | 153948 | phci_DAB_2 |  | Gene | - |
| MSTS01000401 | 153848 | 153948 | phci_DAB_2_exon1 | Y | Exon | - |
| MSTS01000401 | 147997 | 148014 | phci_DAB_2_exon2 | Y | Exon | - |
| MSTS01000401 | 147806 | 147995 | phci_DAB_2_exon3 | Y | Exon | - |
| MSTS01000401 | 147747 | 147805 | phci_DAB_2_exon4 | Y | Exon | - |
| MSTS01000401 | 145730 | 146012 | phci_DAB_2_exon5 | Y | Exon | - |
| MSTS01000401 | 145117 | 145254 | phci_DAB_2_exon6 | Y | Exon | - |
| MSTS01000544 | 63131 | 71671 | phci_DAB_3 |  | Gene | + |
| MSTS01000544 | 63131 | 63231 | phci_DAB_3_exon1 | Y | Exon | + |
| MSTS01000544 | 68767 | 69037 | phci_DAB_3_exon2 | Y | Exon | + |
| MSTS01000544 | 70776 | 71058 | phci_DAB_3_exon3 | Y | Exon | + |
| MSTS01000544 | 71534 | 71671 | phci_DAB_3_exon4 | Y | Exon | + |
| MSTS01000840 | 12395 | 23791 | phci_DAB_4 |  | Gene | - |
| MSTS01000840 | 23691 | 23791 | phci_DAB_4_exon1 | Y | Exon | - |
| MSTS01000840 | 15004 | 15274 | phci_DAB_4_exon2 | Y | Exon | - |
| MSTS01000840 | 13126 | 13231 | phci_DAB_4_exon3 | Y | Exon | - |
| MSTS01000840 | 12950 | 13079 | phci_DAB_4_exon4 | Y | Exon | - |
| MSTS01000840 | 12395 | 12511 | phci_DAB_4_exon5 | Y | Exon | - |
| MSTS01000302 | 47357 | 50746 | phci_DAB_5 |  | Gene | + |
| MSTS01000302 | 47357 | 47682 | phci_DAB_5_exon1 | Y | Exon | + |
| MSTS01000302 | 49275 | 49557 | phci_DAB_5_exon2 | Y | Exon | + |
| MSTS01000302 | 50047 | 50158 | phci_DAB_5_exon3 | Y | Exon | + |
| MSTS01000302 | 50627 | 50746 | phci_DAB_5_exon4 | Y | Exon | + |
| MSTS01000255 | 1175692 | 1178484 | phci_DBA_1 |  | Gene | - |
| MSTS01000255 | 1178399 | 1178484 | phci_DBA_1_exon1 | Y | Exon | - |
| MSTS01000255 | 1176989 | 1177238 | phci_DBA_1_exon2 | Y | Exon | - |
| MSTS01000255 | 1176287 | 1176569 | phci_DBA_1_exon3 | Y | Exon | - |
| MSTS01000255 | 1175692 | 1175847 | phci_DBA_1_exon4 | Y | Exon | - |
| MSTS01000255 | 1141123 | 1143891 | phci_DBA_2 |  | Gene | - |
| MSTS01000255 | 1143806 | 1143891 | phci_DBA_2_exon1 | Y | Exon | - |
| MSTS01000255 | 1142410 | 1142656 | phci_DBA_2_exon2 | Y | Exon | - |
| MSTS01000255 | 1141697 | 1141979 | phci_DBA_2_exon3 | Y | Exon | - |
| MSTS01000255 | 1141123 | 1141278 | phci_DBA_2_exon4 | Y | Exon | - |
| MSTS01000255 | 1118112 | 1122643 | phci_DBB_2 |  | Gene | + |
| MSTS01000255 | 1118112 | 1118212 | phci_DBB_2_exon1 | Y | Exon | + |
| MSTS01000255 | 1120898 | 1121168 | phci_DBB_2_exon2 | Y | Exon | + |
| MSTS01000255 | 1121592 | 1121868 | phci_DBB_2_exon3 | Y | Exon | + |
| MSTS01000255 | 1122464 | 1122643 | phci_DBB_2_exon4 | Y | Exon | + |
| MSTS01000255 | 1153152 | 1157508 | phci_DBB_3 |  | Gene | + |
| MSTS01000255 | 1153152 | 1153252 | phci_DBB_3_exon1 | Y | Exon | + |
| MSTS01000255 | 1155832 | 1156102 | phci_DBB_3_exon2 | Y | Exon | + |
| MSTS01000255 | 1156470 | 1156746 | phci_DBB_3_exon3 | Y | Exon | + |
| MSTS01000255 | 1157329 | 1157508 | phci_DBB_3_exon4 | Y | Exon | + |
| MSTS01000255 | 1414232 | 1417702 | phci_DCA_1 |  | Gene | - |
| MSTS01000255 | 1417620 | 1417702 | phci_DCA_1_exon1 | Y | Exon | - |
| MSTS01000255 | 1415764 | 1416007 | phci_DCA_1_exon2 | Y | Exon | - |
| MSTS01000255 | 1414573 | 1414855 | phci_DCA_1_exon3 | Y | Exon | - |
| MSTS01000255 | 1414232 | 1414363 | phci_DCA_1_exon4 | Y | Exon | - |
| MSTS01000255 | 1420780 | 1430221 | phci_DCB_1 |  | Gene | + |
| MSTS01000255 | 1420780 | 1420940 | phci_DCB_1_exon1 | Y | Exon | + |
| MSTS01000255 | 1425401 | 1425668 | phci_DCB_1_exon2 | Y | Exon | + |
| MSTS01000255 | 1428166 | 1428448 | phci_DCB_1_exon3 | Y | Exon | + |
| MSTS01000255 | 1429124 | 1429235 | phci_DCB_1_exon4 | Y | Exon | + |
| MSTS01000255 | 1430123 | 1430221 | phci_DCB_1_exon5 | Y | Exon | + |
| MSTS01000255 | 316690 | 319913 | phci_DMA_1 |  | Gene | + |
| MSTS01000255 | 316690 | 316781 | phci_DMA_1_exon1 | Y | Exon | + |
| MSTS01000255 | 317434 | 317719 | phci_DMA_1_exon2 | Y | Exon | + |
| MSTS01000255 | 318437 | 318716 | phci_DMA_1_exon3 | Y | Exon | + |
| MSTS01000255 | 318946 | 319081 | phci_DMA_1_exon4 | Y | Exon | + |
| MSTS01000255 | 319908 | 319913 | phci_DMA_1_exon5 | N | Exon | + |
| MSTS01000255 | 340432 | 344441 | phci_DMB_1 |  | Gene | + |
| MSTS01000255 | 340432 | 340502 | phci_DMB_1_exon1 | Y | Exon | + |
| MSTS01000255 | 340891 | 341176 | phci_DMB_1_exon2 | Y | Exon | + |
| MSTS01000255 | 342357 | 342639 | phci_DMB_1_exon3 | Y | Exon | + |
| MSTS01000255 | 343580 | 343697 | phci_DMB_1_exon4 | Y | Exon | + |
| MSTS01000255 | 343822 | 343858 | phci_DMB_1_exon5 | N | Exon | + |
| MSTS01000255 | 344424 | 344441 | phci_DMB_1_exon6 | N | Exon | + |
| MSTS01000347 | 1561564 | 1566230 | phci_MHCI_1_UI |  | Gene | + |
| MSTS01000347 | 1561564 | 1561628 | phci_MHCI_1_UI_exon1 | Y | Exon | + |
| MSTS01000347 | 1562458 | 1562728 | phci_MHCI_1_UI_exon2 | Y | Exon | + |
| MSTS01000347 | 1562922 | 1563201 | phci_MHCI_1_UI_exon3 | Y | Exon | + |
| MSTS01000347 | 1563388 | 1563664 | phci_MHCI_1_UI_exon4 | Y | Exon | + |
| MSTS01000347 | 1563813 | 1563915 | phci_MHCI_1_UI_exon5 | Y | Exon | + |
| MSTS01000347 | 1564426 | 1564459 | phci_MHCI_1_UI_exon6 | Y | Exon | + |
| MSTS01000347 | 1564645 | 1564690 | phci_MHCI_1_UI_exon7 | Y | Exon | + |
| MSTS01000347 | 1566225 | 1566230 | phci_MHCI_1_UI_exon8 | N | Exon | + |
| MSTS01000255 | 986710 | 989575 | phci_MHCI_10_UF |  | Gene | + |
| MSTS01000255 | 986710 | 986768 | phci_MHCI_10_UF_exon1 | Y | Exon | + |
| MSTS01000255 | 986963 | 987233 | phci_MHCI_10_UF_exon2 | Y | Exon | + |
| MSTS01000255 | 987484 | 987763 | phci_MHCI_10_UF_exon3 | Y | Exon | + |
| MSTS01000255 | 988091 | 988367 | phci_MHCI_10_UF_exon4 | Y | Exon | + |
| MSTS01000255 | 988530 | 988639 | phci_MHCI_10_UF_exon5 | Y | Exon | + |
| MSTS01000255 | 989120 | 989152 | phci_MHCI_10_UF_exon6 | Y | Exon | + |
| MSTS01000255 | 989324 | 989369 | phci_MHCI_10_UF_exon7 | Y | Exon | + |
| MSTS01000255 | 989570 | 989575 | phci_MHCI_10_UF_exon8 | N | Exon | + |
| MSTS01000129 | 3854764 | 3858138 | phci_MHCI_12_UH |  | Gene | - |
| MSTS01000129 | 3858074 | 3858138 | phci_MHCI_12_UH_exon1 | Y | Exon | - |
| MSTS01000129 | 3856894 | 3857173 | phci_MHCI_12_UH_exon2 | Y | Exon | - |
| MSTS01000129 | 3856431 | 3856710 | phci_MHCI_12_UH_exon3 | Y | Exon | - |
| MSTS01000129 | 3855951 | 3856227 | phci_MHCI_12_UH_exon4 | Y | Exon | - |
| MSTS01000129 | 3855693 | 3855798 | phci_MHCI_12_UH_exon5 | Y | Exon | - |
| MSTS01000129 | 3855181 | 3855214 | phci_MHCI_12_UH_exon6 | Y | Exon | - |
| MSTS01000129 | 3854951 | 3854996 | phci_MHCI_12_UH_exon7 | Y | Exon | - |
| MSTS01000129 | 3854764 | 3854769 | phci_MHCI_12_UH_exon8 | N | Exon | - |
| MSTS01000129 | 3816075 | 3819564 | phci_MHCI_13_UG |  | Gene | - |
| MSTS01000129 | 3819500 | 3819564 | phci_MHCI_13_UG_exon1 | Y | Exon | - |
| MSTS01000129 | 3818272 | 3818551 | phci_MHCI_13_UG_exon2 | Y | Exon | - |
| MSTS01000129 | 3817817 | 3818096 | phci_MHCI_13_UG_exon3 | Y | Exon | - |
| MSTS01000129 | 3817328 | 3817604 | phci_MHCI_13_UG_exon4 | Y | Exon | - |
| MSTS01000129 | 3817070 | 3817175 | phci_MHCI_13_UG_exon5 | Y | Exon | - |
| MSTS01000129 | 3816520 | 3816553 | phci_MHCI_13_UG_exon6 | Y | Exon | - |
| MSTS01000129 | 3816288 | 3816333 | phci_MHCI_13_UG_exon7 | Y | Exon | - |
| MSTS01000129 | 3816075 | 3816104 | phci_MHCI_13_UG_exon8 | N | Exon | - |
| MSTS01000454 | 94286 | 97231 | phci_MHCI_15_UJ |  | Gene | - |
| MSTS01000454 | 97167 | 97231 | phci_MHCI_15_UJ_exon1 | Y | Exon | - |
| MSTS01000454 | 96666 | 96936 | phci_MHCI_15_UJ_exon2 | Y | Exon | - |
| MSTS01000454 | 96178 | 96457 | phci_MHCI_15_UJ_exon3 | Y | Exon | - |
| MSTS01000454 | 95706 | 95982 | phci_MHCI_15_UJ_exon4 | Y | Exon | - |
| MSTS01000454 | 95448 | 95553 | phci_MHCI_15_UJ_exon5 | Y | Exon | - |
| MSTS01000454 | 94707 | 94740 | phci_MHCI_15_UJ_exon6 | Y | Exon | - |
| MSTS01000454 | 94475 | 94520 | phci_MHCI_15_UJ_exon7 | Y | Exon | - |
| MSTS01000454 | 94286 | 94291 | phci_MHCI_15_UJ_exon8 | N | Exon | - |
| MSTS01000381 | 11507 | 15571 | phci_MHCI_19_UB |  | Gene | - |
| MSTS01000381 | 15507 | 15571 | phci_MHCI_19_UB_exon1 | Y | Exon | - |
| MSTS01000381 | 13597 | 13876 | phci_MHCI_19_UB_exon2 | Y | Exon | - |
| MSTS01000381 | 13151 | 13430 | phci_MHCI_19_UB_exon3 | Y | Exon | - |
| MSTS01000381 | 12679 | 12955 | phci_MHCI_19_UB_exon4 | Y | Exon | - |
| MSTS01000381 | 12438 | 12531 | phci_MHCI_19_UB_exon5 | Y | Exon | - |
| MSTS01000381 | 11926 | 11959 | phci_MHCI_19_UB_exon6 | Y | Exon | - |
| MSTS01000381 | 11694 | 11739 | phci_MHCI_19_UB_exon7 | N | Exon | - |
| MSTS01000381 | 11507 | 11512 | phci_MHCI_19_UB_exon8 | N | Exon | - |
| MSTS01000347 | 1588258 | 1591560 | phci_MHCI_2_UD |  | Gene | + |
| MSTS01000347 | 1588258 | 1588322 | phci_MHCI_2_UD_exon1 | Y | Exon | + |
| MSTS01000347 | 1589149 | 1589428 | phci_MHCI_2_UD_exon2 | Y | Exon | + |
| MSTS01000347 | 1589616 | 1589895 | phci_MHCI_2_UD_exon3 | Y | Exon | + |
| MSTS01000347 | 1590109 | 1590385 | phci_MHCI_2_UD_exon4 | Y | Exon | + |
| MSTS01000347 | 1590510 | 1590606 | phci_MHCI_2_UD_exon5 | Y | Exon | + |
| MSTS01000347 | 1591125 | 1591158 | phci_MHCI_2_UD_exon6 | N | Exon | + |
| MSTS01000347 | 1591555 | 1591560 | phci_MHCI_2_UD_exon7 | N | Exon | + |
| MSTS01000263 | 3139640 | 3143043 | phci_MHCI_4_UA |  | Gene | - |
| MSTS01000263 | 3142979 | 3143043 | phci_MHCI_4_UA_exon1 | Y | Exon | - |
| MSTS01000263 | 3141770 | 3142049 | phci_MHCI_4_UA_exon2 | Y | Exon | - |
| MSTS01000263 | 3141322 | 3141601 | phci_MHCI_4_UA_exon3 | Y | Exon | - |
| MSTS01000263 | 3140847 | 3141123 | phci_MHCI_4_UA_exon4 | Y | Exon | - |
| MSTS01000263 | 3140592 | 3140697 | phci_MHCI_4_UA_exon5 | Y | Exon | - |
| MSTS01000263 | 3140058 | 3140091 | phci_MHCI_4_UA_exon6 | Y | Exon | - |
| MSTS01000263 | 3139829 | 3139874 | phci_MHCI_4_UA_exon7 | Y | Exon | - |
| MSTS01000263 | 3139640 | 3139645 | phci_MHCI_4_UA_exon8 | N | Exon | - |
| MSTS01000255 | 400493 | 407248 | phci_MHCI_5_UK |  | Gene | + |
| MSTS01000255 | 400493 | 400557 | phci_MHCI_5_UK_exon1 | Y | Exon | + |
| MSTS01000255 | 404277 | 404547 | phci_MHCI_5_UK_exon2 | Y | Exon | + |
| MSTS01000255 | 404780 | 405059 | phci_MHCI_5_UK_exon3 | Y | Exon | + |
| MSTS01000255 | 405931 | 406207 | phci_MHCI_5_UK_exon4 | Y | Exon | + |
| MSTS01000255 | 406356 | 406452 | phci_MHCI_5_UK_exon5 | Y | Exon | + |
| MSTS01000255 | 406957 | 406989 | phci_MHCI_5_UK_exon6 | Y | Exon | + |
| MSTS01000255 | 407176 | 407248 | phci_MHCI_5_UK_exon7 | Y | Exon | + |
| MSTS01000255 | 764362 | 767250 | phci_MHCI_8_UC |  | Gene | + |
| MSTS01000255 | 764362 | 764426 | phci_MHCI_8_UC_exon1 | Y | Exon | + |
| MSTS01000255 | 764666 | 764927 | phci_MHCI_8_UC_exon2 | Y | Exon | + |
| MSTS01000255 | 765146 | 765425 | phci_MHCI_8_UC_exon3 | Y | Exon | + |
| MSTS01000255 | 765773 | 766049 | phci_MHCI_8_UC_exon4 | Y | Exon | + |
| MSTS01000255 | 766206 | 766314 | phci_MHCI_8_UC_exon5 | Y | Exon | + |
| MSTS01000255 | 766791 | 766824 | phci_MHCI_8_UC_exon6 | Y | Exon | + |
| MSTS01000255 | 766999 | 767044 | phci_MHCI_8_UC_exon7 | Y | Exon | + |
| MSTS01000255 | 767245 | 767250 | phci_MHCI_8_UC_exon8 | N | Exon | + |
| MSTS01000255 | 839055 | 841935 | phci_MHCI_9_UE |  | Gene | + |
| MSTS01000255 | 839055 | 839119 | phci_MHCI_9_UE_exon1 | Y | Exon | + |
| MSTS01000255 | 839359 | 839619 | phci_MHCI_9_UE_exon2 | Y | Exon | + |
| MSTS01000255 | 839850 | 840129 | phci_MHCI_9_UE_exon3 | Y | Exon | + |
| MSTS01000255 | 840478 | 840756 | phci_MHCI_9_UE_exon4 | Y | Exon | + |
| MSTS01000255 | 840913 | 841021 | phci_MHCI_9_UE_exon5 | Y | Exon | + |
| MSTS01000255 | 841476 | 841509 | phci_MHCI_9_UE_exon6 | Y | Exon | + |
| MSTS01000255 | 841684 | 841729 | phci_MHCI_9_UE_exon7 | Y | Exon | + |
| MSTS01000255 | 841930 | 841935 | phci_MHCI_9_UE_exon8 | N | Exon | + |
| MSTS01000003 | 11877704 | 11880122 | phci_TLR1/6-like |  | Gene | + |
| MSTS01000003 | 11877704 | 11880122 | phci_TLR1/6-like_exon1 | Y | Exon | + |
| MSTS01000003 | 11925125 | 11927487 | phci_TLR10 |  | Gene | + |
| MSTS01000003 | 11925125 | 11927487 | phci_TLR10_exon1 | Y | Exon | + |
| MSTS01000056 | 7981884 | 7984236 | phci_TLR2 |  | Gene | + |
| MSTS01000056 | 7981884 | 7984236 | phci_TLR2_exon1 | Y | Exon | + |
| MSTS01000124 | 160216 | 175373 | phci_TLR3 |  | Gene | - |
| MSTS01000124 | 174929 | 175373 | phci_TLR3_exon1 | Y | Exon | - |
| MSTS01000124 | 170745 | 170937 | phci_TLR3_exon2 | Y | Exon | - |
| MSTS01000124 | 162868 | 164721 | phci_TLR3_exon3 | Y | Exon | - |
| MSTS01000124 | 160216 | 160445 | phci_TLR3_exon4 | Y | Exon | - |
| MSTS01000040 | 4959426 | 4961655 | phci_TLR4 |  | Gene | - |
| MSTS01000040 | 4959426 | 4961655 | phci_TLR4_exon1 | Y | Exon | - |
| MSTS01000013 | 14858499 | 14861079 | phci_TLR5 |  | Gene | + |
| MSTS01000013 | 14858499 | 14861079 | phci_TLR5_exon1 | Y | Exon | + |
| MSTS01000122 | 1447018 | 1450159 | phci_TLR7 |  | Gene | + |
| MSTS01000122 | 1447018 | 1450159 | phci_TLR7_exon1 | Y | Exon | + |
| MSTS01000122 | 1496962 | 1500084 | phci_TLR8 |  | Gene | + |
| MSTS01000122 | 1496962 | 1500084 | phci_TLR8_exon1 | Y | Exon | + |
| MSTS01000216 | 372490 | 375570 | phci_TLR9 |  | Gene | - |

Supplementary Table 2: Coding used to assess concordance between “true” target enrichment variants and whole genome sequencing variant calls at nine different coverages (0.5x, 1x, 2x, 5x, 10x, 15x, 20x, 30x and full coverage- see Table S1 for full coverage depths)

|  | Target Enrichment Homozygous Reference (0/0) | Target Enrichment Heterozygous (0/1) | Target Enrichment Homozygous Alternate (1/1) | Target Enrichment Missing (./.) |
| --- | --- | --- | --- | --- |
| WGS Homozygous Reference (0/0) | A | B | C | D |
| WGS Heterozygous (0/1) | E | F | G | H |
| WGS Heterozygous Alternate (1/1) | I | J | K | L |
| WGS Missing (./.) | M | N | O | P |

## Supplementary Figures


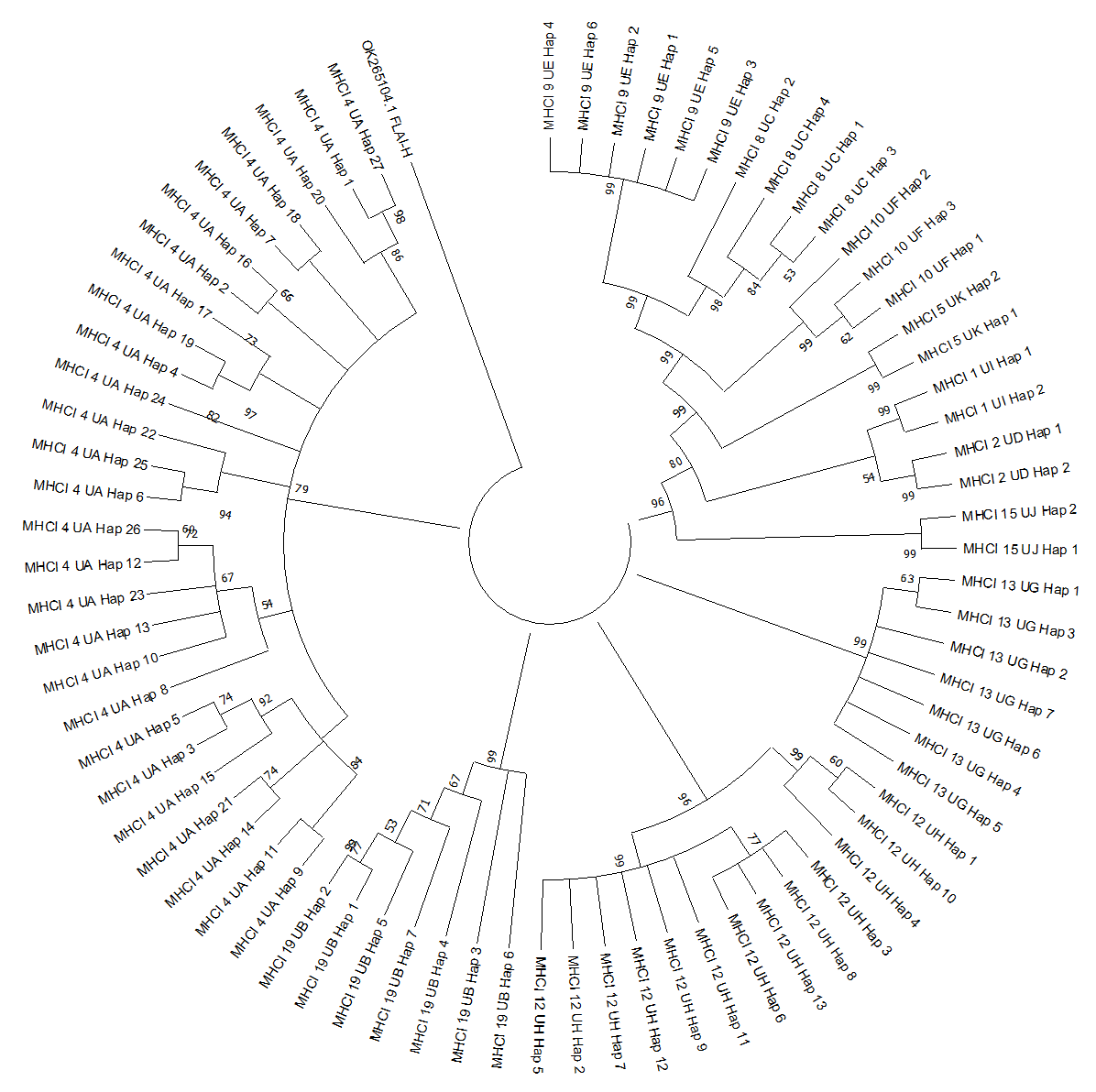


Supplementary Figure 1: Neighbour joining tree with 1000 bootstrapping replicated of koala MHC class I alleles identified in this study, rooted with Feline FLA sequence OK265104.1. Neighbour joining tree was run with a Tamura 3-parameter model with a Gamma distribution, node values represent bootsrapping support, only support values greater than 50 are shown


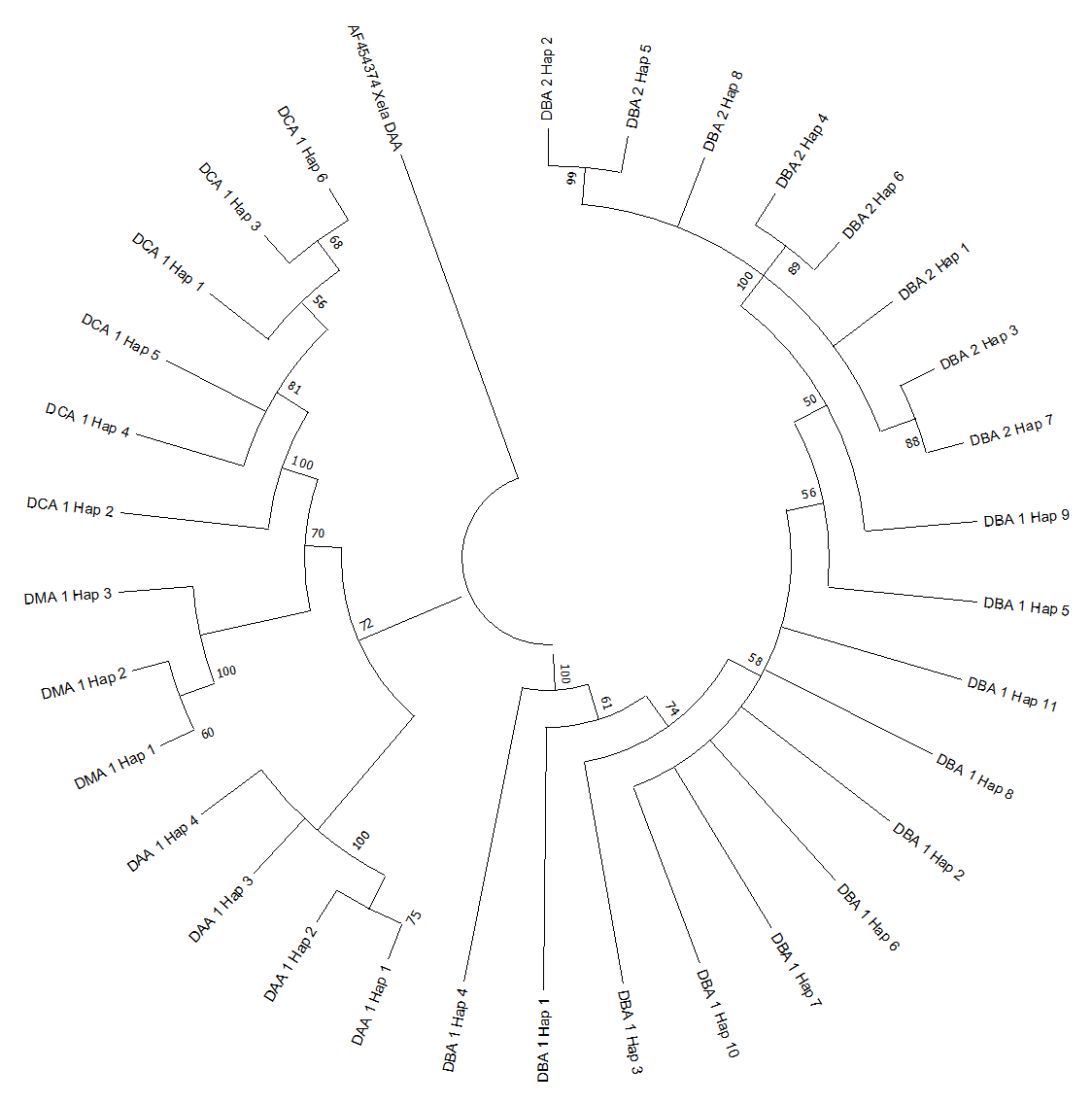


Supplementary Figure 2: Neighbour joining tree with 1000 bootstrapping replicated of koala MHC class II alpha gene alleles identified in this study, rooted with African clawed frog (*Xenopus laevis)* DAA sequence AF454374.1. Neighbour joining tree was run with a Kumara 2-parameter model with a Gamma distribution, node values represent bootsrapping support, only support values greater than 50 are shown


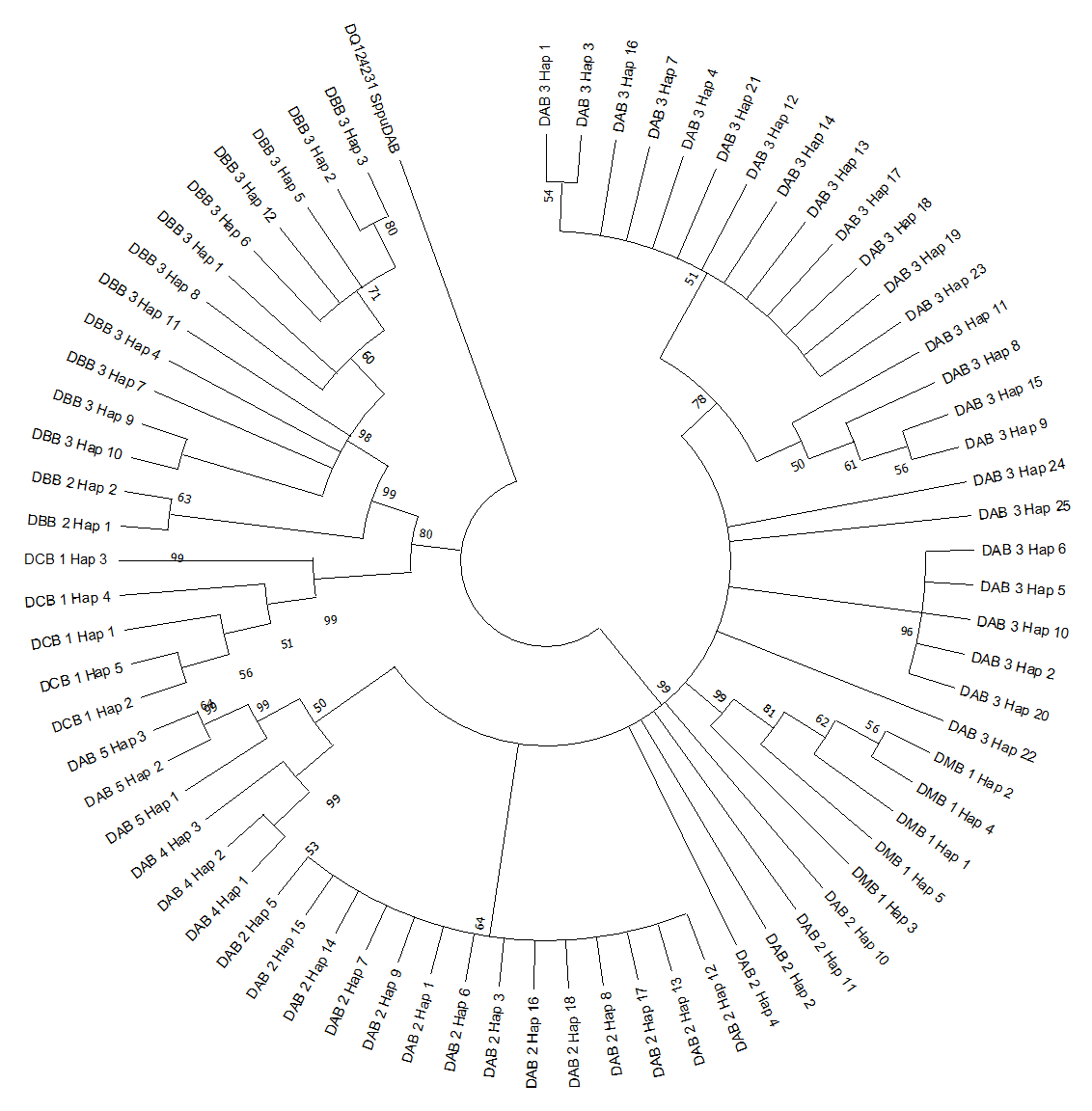


Supplementary Figure 3: Neighbour joining tree with 1000 bootstrapping replicated of koala MHC class II beta gene alleles identified in this study, rooted with Tuatara (*Sphenodon punctatus)* DAB sequence DQ124321.1. Neighbour joining tree was run with a Kumara 2-parameter model with a Gamma distribution, node values represent bootsrapping support, only support values greater than 50 are shown


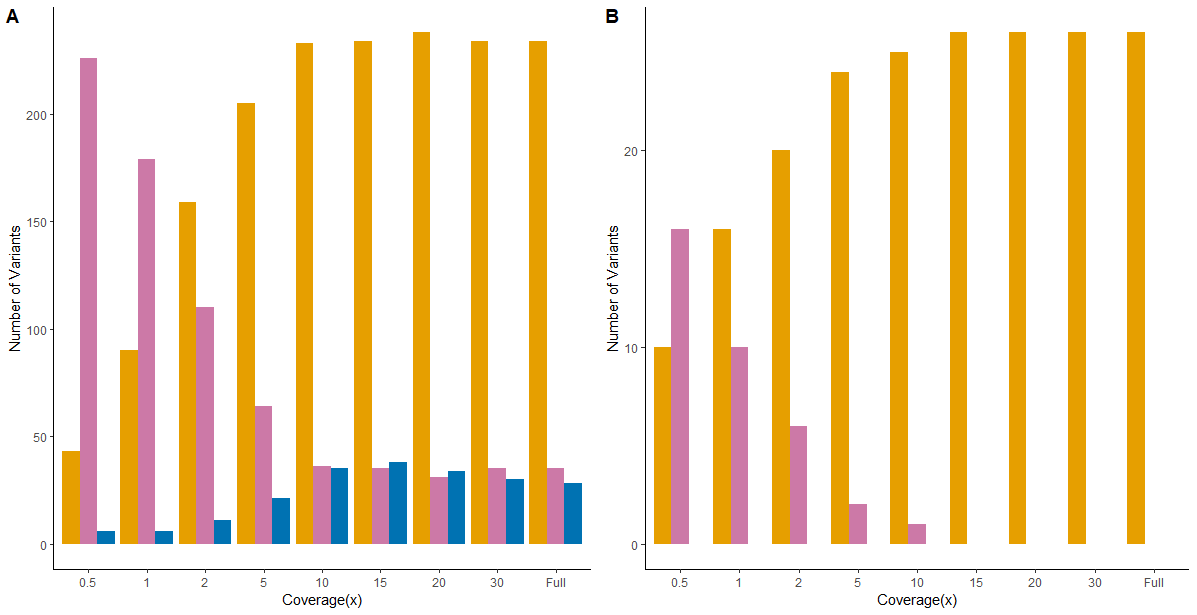


Supplementary Figure 4 Stacked column chart showing the number of variants present in MHC genes (A) and TLR genes (B) at different sequencing depth. Colours indicate the number of variants present only identified through target enrichment (pink), only through whole genome sequencing (blue) and overlapping between the two sequencing types (orange)


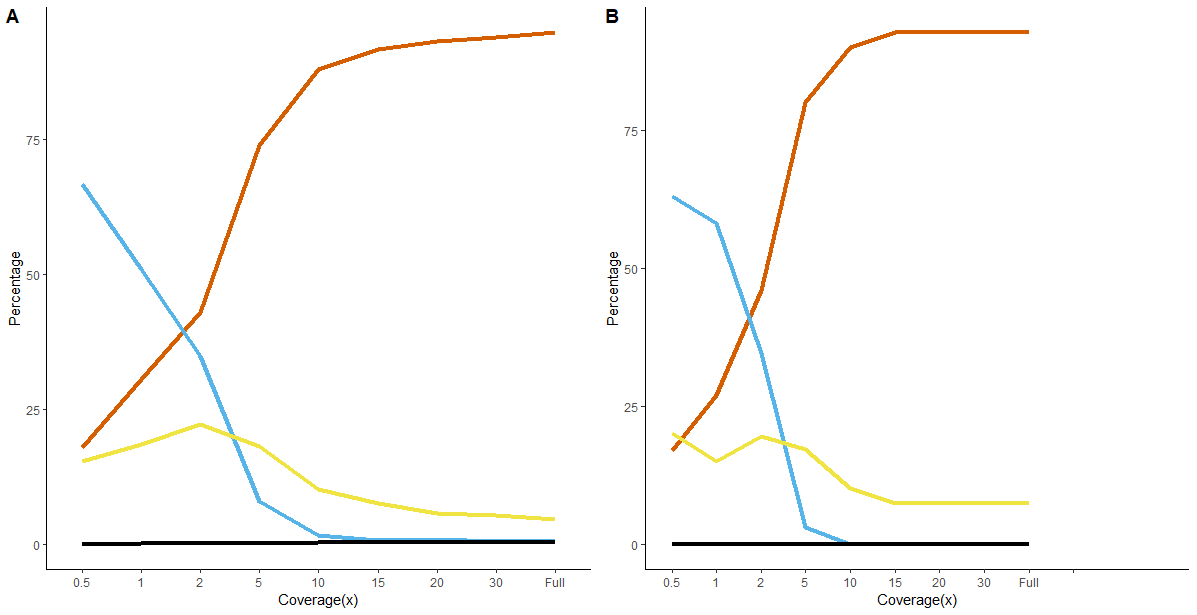


Supplementary Figure 5: Stacked line chart showing the concordance of variant calls between target enrichment and whole genome sequencing at different sequencing depth in MHC genes (A) and TLR genes (B). Red lines show the percentage correct calls, blue lines show the percentage of variant calls present in target enrichment but missing in WGS, yellow lines show the percentage of non-concordance calls and black lines shows percentage variant calls present in WGS but missing in target enrichment


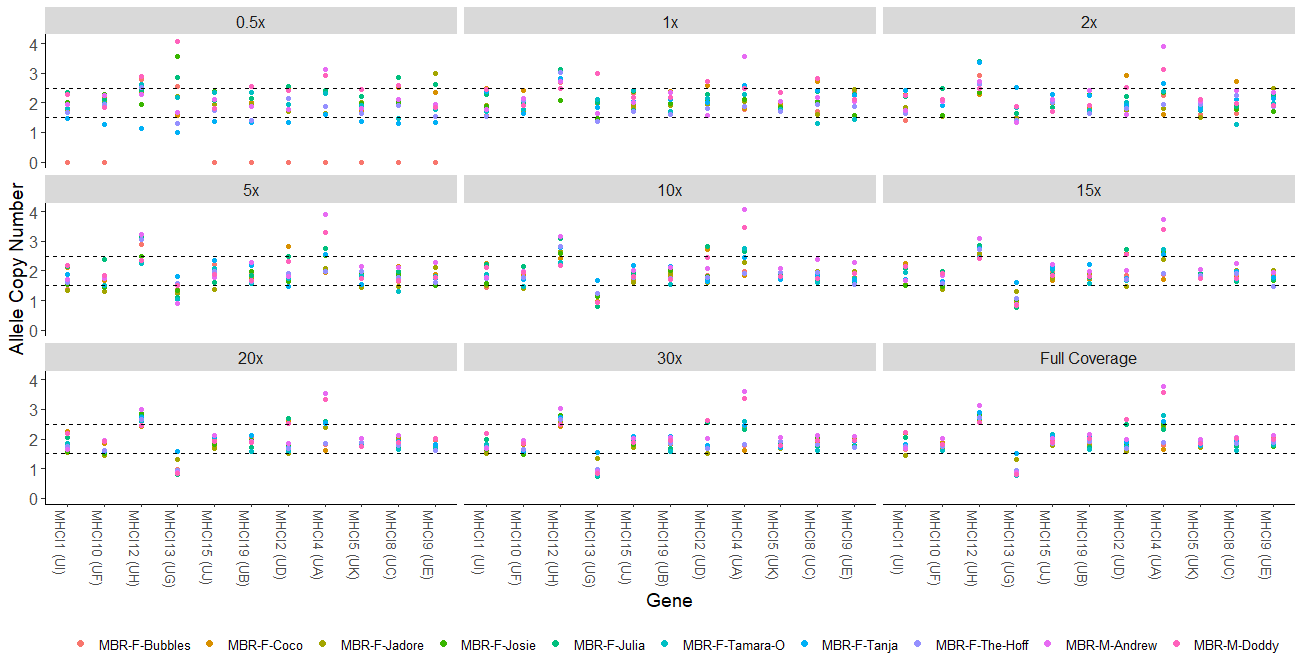


Supplementary Figure 6: A dot plot showing the calculated copy number at 11 class I genes at nine different sequencing depths. Each coloured dot represents a single individual. Points that fall between the two dotted lines (1.5 and 2.5) are presumed to be single copy genes in those individuals, points falling below the lower dotted line (1.5) are presumed to be deletions in those individuals and points above the higher dotted line (2.5) are putative duplications in those individuals. Genes names are labelled as identified in Cheng et al. (2018) and Silver et al. (2022).


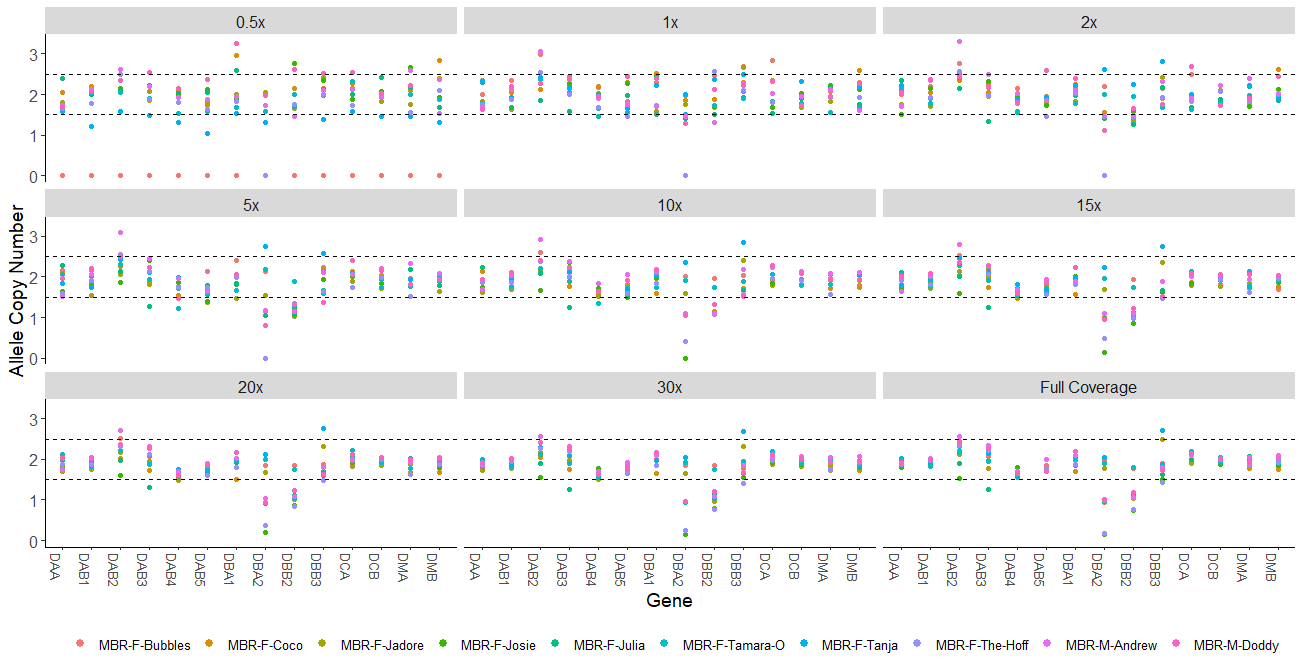


Supplementary Figure 7: A dot plot showing the calculated copy number at 14 class II genes at nine different sequencing depths. Each coloured dot represents a single individual. Points that fall between the two dotted lines (1.5 and 2.5) are presumed to be single copy genes in those individuals, points falling below the lower dotted line (1.5) are presumed to be deletions in those individuals and points above the higher dotted line (2.5) are putative duplications in those individuals. Genes names are labelled as identified in Cheng et al. (2018) and Silver et al. (2022)

Cheng, Y., Polkinghorne, A., Gillett, A., Jones, E. A., O'Meally, D., Timms, P., & Belov, K. (2018). Characterisation of MHC class I genes in the koala. *Immunogenetics*, *70*(2), 125-133. <https://doi.org/10.1007/s00251-017-1018-2>

Silver, L. W., Cheng, Y., Quigley, B. L., Robbins, A., Timms, P., Hogg, C. J., & Belov, K. (2022). A targeted approach to investigating immune genes of an iconic Australian marsupial. *Mol Ecol*, *31*(12), 3286-3303. <https://doi.org/10.1111/mec.16493>

Additional Data 1

>DAA_1_Hap_3

ATGACCCCCAACAAAGCCTTGATCCTAGGAGTCTTCATCCTGGCAGTGCTGTTGAGTCCCTGGGGTGCCAGAGCTGTTAAAGAGAAGCATGTGATCATCCAGGCAGAGTTCTACCAGACCCACGACCCCTTGGGAGAGTTCATGTTTGACTTTGATGGAGATGAGATTTTCCATGTGGATTTGAAGAGCAAAGAGACAGTCTGGAGGCTTCCTGACTTCAGCAAATTTGCCAGTTTTGAGGCTCAGGGTGCTCTGGCCAACCTTGCTGTGGACAAAGCCAACCTGGAAACCATGATGAAACGGTCCAACAACACCCCTGACACCAACGTACCCCCTGAAGTGACAGTGTTTCCCAAGGGCCCAGTGGAGCTGGGCCAGCCCAACGTCCTTGTCTGCTTTGTTGACAAGTTCTCTCCTCCCGTGCTTACTGTGACATGGCTTCATAATGGGCGTCCCATCACTGATGGTGTGTTTGAGACTGTCTTCCTCCCCCGTCCTGACCACTCCTTCAGAAAATTCCACTACCTCACTTTCATCCCCTCTGCCACCGATTACTATGACTGCAAGGTCGAGCACTGGGGACTGGAACAACCTGCTGTCAAACATTGGGAACCAGAAATACGGACCCCACTGCCAGAGACAACAGAGACTGTGGTCTGTGCCCTGGGCCTGGCCATAGGCCTGGTGGGCATCGTCGTAGGCACCATCCTTATTATTAAGGGCATGCGAGCAAGCAACGCTTCCCGTGGTGGCCCTCGTGGAACCCTGTAA

>DAA_1_Hap_1

ATGACCCCCAACAAAGCCTTGATCCTAGGAGTCTTCATCCTGGCAGTGCTGTTGAGTCCCTGGGGTGCCAGAGCTGTTAAAGAGAAGCATGTGATCATCCAGGCAGAGTTCTATCAGACCCATGACCCCTTGGGAGAGTTCATGTTTGACTTTGATGGAGATGAGATTTTCCATGTGGATTTGAAGAGCAAAGAGACAGTCTGGAGGCTTCCTGACTTCAGCAAATTTGCCAGTTTTGAGGCTCAGGGTGCTCTGGCCAACCTTGCTGTGGACAAAGCCAACCTGGAAACCATGATGAAACGGTCCAACAACACCCCTGACACCAACGTACCCCCTGAAGTGACAGTGTTTCCCAAGGGCCCAGTGGAGCTGGGACAGCCCAACGTCCTTGTCTGCTTTGTTGACAAGTTCTCTCCTCCCGTGCTTACTGTGACATGGCTTCATAATGGGCGTCCCATCACTGATGGTGTGTTTGAGACTGTCTTCCTCCCCCGCCCTGACCACTCCTTCAGAAAATTCCACTACCTCACTTTCATCCCCTCTGCCACCGATTACTATGACTGCAAGGTCGAGCACTGGGGACTGGAACAACCTGCTGTCAAACATTGGGAACCAGAAATACGGACCCCACTGCCAGAGACAACAGAGACTGTGGTCTGTGCCCTGGGCCTGGCCATAGGCCTGGTGGGCATTGTCGTAGGCACCATCCTTATTATTAAGGGCATGCGAGCAAGCAACGCTTCCCGTGGTGGCCCTCGTGGAACCCTGTAA

>DAA_1_Hap_2

ATGACCCCCAACAAAGCCTTGATCCTAGGAGTCTTCATCCTGGCAGTGCTGTTGAGTCCCTGGGGTGCCAGAGCTGTTAAAGAGAAGCATGTGATCATCCAGGCAGAGTTCTACCAGACCCACGACCCCTTGGGAGAGTTCATGTTTGACTTTGATGGAGATGAGATTTTCCATGTGGATTTGAAGAGCAAAGAGACAGTCTGGAGGCTTCCTGACTTCAGCAAATTTGCCAGTTTTGAGGCTCAGGGTGCTCTGGCCAACCTTGCTGTGGACAAAGCCAACCTGGAAACCATGATGAAACGGTCCAACAACACCCCTGACACCAACGTACCCCCTGAAGTGACAGTGTTTCCCAAGGGCCCAGTGGAGCTGGGACAGCCCAACGTCCTTGTCTGCTTTGTTGACAAGTTCTCTCCTCCCGTGCTTACTGTGACATGGCTTCATAATGGGCGTCCCATCACTGATGGTGTGTTTGAGACTGTCTTCCTCCCCCGCCCTGACCACTCCTTCAGAAAATTCCACTACCTCACTTTCATCCCCTCTGCCACCGATTACTATGACTGCAAGGTCGAGCACTGGGGACTGGAACAACCTGCTGTCAAACATTGGGAACCAGAAATACGGACCCCACTGCCAGAGACAACAGAGACTGTGGTCTGTGCCCTGGGCCTGGCCATAGGCCTGGTGGGCATTGTCGTAGGCACCATCCTTATTATTAAGGGCATGCGAGCAAGCAACGCTTCCCGTGGTGGCCCTCGTGGAACCCTGTAA

>DAA_1_Hap_4

ATGACCCCCAACAAAGCCTTGATCCTAGGAGTCTTCATCCTGGCAGTGCTGTTGAGTCCCTGGGGTGCCAGAGCTGTTAAAGAGAAGCATGTGATCATCCAGGCAGAGTTCTACCAGACCCATGACCCCTTGGGAGAGTTCATGTTTGACTTTGATGGAGATGAGATTTTCCATGTGGATTTGAAGAGCAAAGAGACAGTCTGGAGGCTTCCTGACTTCAGCAAATTTGCCAGTTTTGAGGCTCAGGGTGCTCTGGCCAACCTTGCTGTGGACAAAGCCAACCTGGAAACCATGATGAAACGGTCCAACAACACCCCTGACACCAACGTACCCCCTGAAGTGACAGTGTTTCCCAAGGGCCCAGTGGAGCTGGGCCAGCCCAACGTCCTTGTCTGCTTTGTTGACAAGTTCTCTCCTCCCGTGCTTACTGTGACATGGCTTCATAATGGGCGTCCCATCACTGATGGTGTGTTTGAGACTGTCTTCCTCCCCCGTCCTGACCACTCCTTCAGAAAATTCCACTACCTCACTTTCATCCCCTCTGCCACCGATTACTATGACTGCAAGGTCGAGCACTGGGGACTGGAACAACCTGCTGTCAAACATTGGGAACCAGAAATACGGACCCCACTGCCAGAGACAACAGAGACTGTGGTCTGTGCCCTGGGCCTGGCCATAGGCCTGGTGGGCATCGTCGTAGGCACCATCCTTATTATTAAGGGCATGCGAGCAAGCAACGCTTCCCGTGGTGGCCCTCGTGGAACCCTGTAA

>DAB_2_Hap_3

ATGGTGTGTGTGCTGTTCCCCGGGGGAGTCTGGACAGAGGTTCTGGCTGTGACCCTGCTGGTGCTGAATTCCCAGGTGGCTGCAGGCAGACATGCCCCAAAGCACTTCACTGAGTATAAGTCCGAGTGTTACTTCGGGAACGGGACGGAGCGCGTGCGGTTTGTGGAGAGATACATCCACAACCGGGAGGAGTATGTGCGCTTCGACAGCGACGTGGGGGAGTTTGTGGCGGTGACGGAGCTGGGACGGCCCGATGCTGAGCGTTGGAACAGCCAGAAGGAGATCCTGGAGGACGAACGGGCCCGGGGACACTTGTGCAGGCACAACTACGGGGTGTCTGAGCCCTTCTTAGTGCGCAGGAGCGTTGAGCCTGAGGTGATTGTGTATCCATCAAAGATGGCTCCCCTGGGACACCACAACCTGCTTGTCTGCTCTGTCAGTGGTTTCTATCCTGGGGACATTGAGGTCAGGTGGTTCCTGAATGGGCAGGAGGAGACGGCTGGGGTTGTGTCCACAGGCCTGATCAGCAATGGAGACTGGACCTACCAGCTCCTGGTGATGCTGGAAATGACCCCCAAGCGTGGAGACGTCTACACCTGCCAAGTGGAGCACTCCAGCCTTCAGAAACCTGTTGTCTTGGACTGGAAAGCACAGTCTGAATCTGCCCAGAGTAAGATGCTGAGTGGAGTCGGGGGCCTCGTGCTGGGCCTGATCTTCTTTGGGGTTGGCCTCTTTGTCTACAAGAGGAGTCAGAAAGGTGAGACACTCTGGGGAAAACCCTGA

>DAB_2_Hap_1

ATGGTGTGTGTGCTGTTCCCCGGGGGAGTCTGGACAGAGGTTCTGGCTGTGACCCTGCTGGTGCTGAATTCCCAGGTGGCTGCAGGCAGACATGCCCCAAAGCACTTCACTGAGTATAAGTCCGAGTGTTACTTCGGGAACGGGACGGAGCGCGTGCGGTTTGTGGAGAGATACATCCACAACCGGGAGGAGTATGCGCGCTTCGACAGCGACGTGGGGGAGTTTGTGGCGGTGACGGAGCTGGGACGGCCCGATGCTGAGCGTTGGAACAGCCAGAAGGAGATCCTGGAGGACGAACGGGCCCGGGGACACTTGTGCAGGCACAACTACGGGGTGTCTGAGCCCTTCTTAGTGCGCAGGAGCGTTGAGCCTGAGGTGATTGTGTATCCATCAAAGATGGCTCCCCTGGGACACCACAACCTGCTTGTCTGCTCTGTCAGTGGTTTCTATCCTGGGGACATTGAGGTCAGGTGGTTCCTGAATGGGCAGGAGGAGACGGCTGGGGTTGTGTCCACAGGCCTGATCAGCAATGGAGACTGGACCTACCAGCTCCTGGTGATGCTGGAAATGACCCCCAAGCGTGGAGACGTCTACACCTGCCAAGTGGAGCACTCCAGCCTTCAGAAACCTGTTGTCTTGGACTGGAAAGCACAGTCTGAATCTGCCCAGAGTAAGATGCTGAGTGGAGTCGGGGGCCTCGTGCTGGGCCTGATCTTCTTTGGGGTTGGCCTCTTTGTCTACAAGAGGAGTCAGAAAGGTGAGACACTCTGGGGAAAACCCTGA

>DAB_2_Hap_5

ATGGTGTGTGTGCTGTTCCCCGGGGGAGTCTGGACAGAGGTTCTGGCTGTGACCCTGCTGGTGCTGAATTCCCAGGTGGCTGCAGGCAGACATGCCCCAAAGCACTTCACTGAGTATAAGTCCGAGTGTTACTTCGGGAACGGGACGGAGCGCGTGCGGTTTGTGGAGAGACACATCCACAACCGGGAGGAGTATGCGCGCTTCGACAGCGACGTGGGGGAGTTTGTGGCGGTGACGGAGCTGGGACGGCCCGATGCTGAGCGTTGGAACAGCCAGAAGGAGATCCTGGAGGACGAACGGGCCCGGGGACACTTGTGCAGGCACAACTACGGGGTGTCTGAGCCCTTCTTAGTGCGCAGGAGCGTTGAGCCTGAGGTGATTGTGTATCCATCAAAGATGGCTCCCCTGGGACACCACAACCTGCTTGTCTGCTCTGTCAGTGGTTTCTATCCTGGGGACATTGAGGTCAGGTGGTTCCTGAATGGGCAGGAGGAGACGGCTGGGGTTGTGTCCACAGGCCTGATCAGCAATGGAGACTGGACCTACCAGCTCCTGGTGATGCTGGAAATGACCCCCAAGCGTGGAGACGTCTACACCTGCCAAGTGGAGCACTCCAGCCTTCAGAAACCTGTTGTCTTGGACTGGAAAGCACAGTCTGAATCTGCCCAGAGTAAGATGCTGAGTGGAGTCGGGGGCCTCGTGCTGGGCCTGATCTTCTTTGGGGTTGGCCTCTTTGTCTACAAGAGGAGTCAGAAAGGTGAGACACTCTGGGGAAAACCCTGA

>DAB_2_Hap_6

ATGGTGTGTGTGCTGTTCCCCGGGGGAGTCTGGACAGAGGTTCTGGCTGTGACCCTGCTGGTGCTGAATTCCCAGGTGGCTGCAGGCAGACATGCCCCAAAGCACTTCACTGAGTATAAGTCCGAGTGTTACTTCGGGAACGGGACGGAGCGCGTGCGGTTTGTGGAGAGATACATCTACAACCGGGAGGAGTATGTGCGCTTCGACAGCGACGTGGGGGAGTTTGTGGCGGTGACGGAGCTGGGACGGCCCGATGCTGAGCGTTGGAACAGCCAGAAGGAGATCCTGGAGGACGAACGGGCCCGGGGACACTTGTGCAGGCACAACTACGGGGTGTCTGAGCCCTTCTTAGTGCGCAGGAGCGTTGAGCCTGAGGTGATTGTGTATCCATCAAAGATGGCTCCCCTGGGACACCACAACCTGCTTGTCTGCTCTGTCAGTGGTTTCTATCCTGGGGACATTGAGGTCAGGTGGTTCCTGAATGGGCAGGAGGAGACGGCTGGGGTTGTGTCCACAGGCCTGATCAGCAATGGAGACTGGACCTACCAGCTCCTGGTGATGCTGGAAATGACCCCCAAGCGTGGAGACGTCTACACCTGCCAAGTGGAGCACTCCAGCCTTCAGAAACCTGTTGTCTTGGACTGGAAAGCACAGTCTGAATCTGCCCAGAGTAAGATGCTGAGTGGAGTCGGGGGCCTCGTGCTGGGCCTGATCTTCTTTGGGGTTGGCCTCTTTGTCTACAAGAGGAGTCAGAAAGGTGAGACACTCTGGGGAAAACCCTGA

>DAB_2_Hap_7

ATGGTGTGTGTGCTGTTCCCCGGGGGAGTCTGGACAGAGGTTCTGGCTGTGACCCTGCTGGTGCTGAATTCCCAGGTGGCTGCAGGCAGACATGCCCCAAAGCACTTCACTGAGTATAAGTCCGAGTGTTACTTCGGGAACGGGACGGAGCGCGTGCGGTTTGTGGAGAGATACATCTACAACCGGGAGGAGTATGCGCGCTTCGACAGCGACGTGGGGGAGTTTGTGGCGGTGACGGAGCTGGGACGGCCCGATGCTGAGCGTTGGAACAGCCAGAAGGAGATCCTGGAGGACGAACGGGCCCGGGGACACTTGTGCAGGCACAACTACGGGGTGTCTGAGCCCTTCTTAGTGCGCAGGAGCGTTGAGCCTGAGGTGATTGTGTATCCATCAAAGATGGCTCCCCTGGGACACCACAACCTGCTTGTCTGCTCTGTCAGTGGTTTCTATCCTGGGGACATTGAGGTCAGGTGGTTCCTGAATGGGCAGGAGGAGACGGCTGGGGTTGTGTCCACAGGCCTGATCAGCAATGGAGACTGGACCTACCAGCTCCTGGTGATGCTGGAAATGACCCCCAAGCGTGGAGACGTCTACACCTGCCAAGTGGAGCACTCCAGCCTTCAGAAACCTGTTGTCTTGGACTGGAAAGCACAGTCTGAATCTGCCCAGAGTAAGATGCTGAGTGGAGTCGGGGGCCTCGTGCTGGGCCTGATCTTCTTTGGGGTTGGCCTCTTTGTCTACAAGAGGAGTCAGAAAGGTGAGACACTCTGGGGAAAACCCTGA

>DAB_2_Hap_4

ATGGTGTGTGTGCTGTTCCCCGGGGGAGTCTGGACAGAGGTTCTGGCTGTGACCCTGCTGGTGCTGAATTCCCAGGTGGCTGCAGGCAGACATGCCCCAAAGCACTTCACGGAGCAGAAGTCCGACTGTCACTTCGAGAACGGGACGGAGCGCGTGCGGTTTGTGGAGAGATACATCCACAACCGGGAGGAGTACGTGCGCTTCGACAGCGACGTGGGGGAGTTTGTGGCGGTGACGGAGCTGGGACGGCCCGATGCTGAGCGTTGGAACAGCCAGAAGGAGATCCTGGAGGACGAACGGGCCCGGGGACACTTGTGCAGGCACAACTACGGGGTGTCTGAGCCCTTCTTAGTGCGCAGGAGCGTTGAGCCCGAGGTGATTGTGTATCCATCAAAGATGGCTCCCCTGGGACACCACAACCTGCTTGTCTGCTCTGTCAGTGGTTTCTATCCTGGGGACATTGAGGTCAGGTGGTTCCTGAATGGGCAGGAGGAGACGGCTGGGGTTGTGTCCACAGGCCTGATCAGCAATGGAGACTGGACCTACCAGCTCCTGGTGATGCTGGAAATGACCCCCAAGCGTGGAGACGTCTACACCTGCCAAGTGGAGCACTCCAGCCTTCAGAAACCTGTTGTCTTGGACTGGAAAGCACAGTCTGAATCTGCCCAGAGTAAGATGCTGAGTGGAGTCGGGGGCCTCGTGCTGGGCCTGATCTTCTTTGGGGTTGGCCTCTTTGTCTACAAGAGGAGTCAGAAAGGTGAGACACTCTGGGGAAAACCCTGA

>DAB_2_Hap_9

ATGGTGTGTGTGCTGTTCCCCGGGGGAGTCTGGACAGAGGTTCTGGCTGTGACCCTGCTGGTGCTGAATTCCCAGGTGGCTGCAGGCAGACATGCCCCAAAGCACTTCACTGAGTATAAGTCCGAGTGTTACTTCGGGAACGGGACGGAGCGCGTGCGGTTTGTGGAGAGATACATCCACAACCGGGAGGAGTATGCGCGCTTCGACAGCGACGTGGGGGAGTTTGTGGCGGTGACGGAGCTGGGACGGCCCGATGCTGAGCGTTGGAACAGCCAGAAGGAGATCCTGGAGGACGAACGGGCCCGGGGACACTTGTGCAGGCACAACTACGGGGTGTCTGAGCCCTTCTTAGTGCGCAGGAGCGTTGAGCCTGAGGTGATTGTGTATCCATCAAAGATGGCTCCCCTGGGACACCACAACCTGCTTGTCTGCTCTGTCAGTGGTTTCTATCCTGGGGACATTGAGGTCAGGTGGTTCCTGAATGGGCAGGAGGAGACGGCTGGGGTTGTGTCCACAGGCCTGATCAGCAATGGAGACTGGACCTACCAGCTCCTGGTGATGCTGGAAATGACCCCCAAGCGTGGAGACGTCTACACCTGCCAAGTGGAGCACTCCAGCCTTCAGAAACCTGTTGTCTTGGACTGGAAAGCACAGTCTGAATCTGCCCAGAGTAAGATGCTGAGTGGAGTCGGGGGCCTCGTGCTGGGCCTGATCTTCTTTGGGGTTGGCCTCTTTGTCTACAAGAGGAGTCAGAAAGGTGAGACACTCTGGGGAAAACCCTGA

>DAB_2_Hap_11

ATGGTGTGTGTGCTGTTCCCCGGGGGAGTCTGGACAGAGGTTCTGGCTGTGACCCTGCTGGTGCTGAATTCCCAGGTGGCTGCAGGCAGACATGCCCCAAAGCACTTCACGGAGCAGAAGTCCGACTGTCACTTCGAGAACGGGACGGAGCGCGTGCGGTTTGTGGAGAGATACATCCACAACCGGGAGGAGTACGTGCGCTTCGACAGCGACGTGGGGGAGTTTGTGGCGGTGACGGAGCTGGGACGGCCCGATGCTGAGCGTTGGAACAGCCAGAAGGAGATCCTGGAGGACGAACGGGCCCGGGGACACTTGTGCAGGCACAACTACGGGGTGTCTGAGCCCTTCTTAGTGCGCAGGAGCGTTGAGCCTGAGGTGATTGTGTATCCATCAAAGATGGCTCCCCTGGGACACCACAACCTGCTTGTCTGCTCTGTCAGTGGTTTCTATCCTGGGGACATTGAGGTCAGGTGGTTCCTGAATGGGCAGGAGGAGACGGCTGGGGTTGTGTCCACAGGCCTGATCAGCAATGGAGACTGGACCTACCAGCTCCTGGTGATGCTGGAAATGACCCCCAAGCGTGGAGACGTCTACACCTGCCAAGTGGAGCACTCCAGCCTTCAGAAACCTGTTGTCTTGGACTGGAAAGCACAGTCTGAATCTGCCCAGAGTAAGATGCTGAGTGGAGTCGGGGGCCTCGTGCTGGGCCTGATCTTCTTTGGGGTTGGCCTCTTTGTCTACAAGAGGAGTCAGAAAGGTGAGACACTCTGGGGAAAACCCTGA

>DAB_2_Hap_12

ATGGTGTGTGTGCTGTTCCCCGGGGGAGTCTGGACAGAGGTTCTGGCTGTGACCCTGCTGGTGCTGAATTCCCAGGTGGCTGCAGGCAGACATGCCCCAAAGCACTTCACTGAGTAGAAGTCCGAGTGTTACTTCGAGAACGGGACGGAGCGCGTGCGGTTTGTGGAGAGATACATCTACAACCGGGAGGAGTATGCGCGCTTCGACAGCGACGTGGGGGAGTTTGTGGCGGTGACGGAGCTGGGACGGCCCGATGCTGAGCGTTGGAACAGCCAGAAGGAGATCCTGGAGGACGAACGGGCCCGGGGACACTTGTGCAGGCACAACTACGGGGTGTCTGAGCCCTTCTTAGTGCGCAGGAGCGTTGAGCCCGAGGTGATTGTGTATCCATCAAAGATGGCTCCCCTGGGACACCACAACCTGCTTGTCTGCTCTGTCAGTGGTTTCTATCCTGGGGACATTGAGGTCAGGTGGTTCCTGAATGGGCAGGAGGAGACGGCTGGGGTTGTGTCCACAGGCCTGATCAGCAATGGAGACTGGACCTACCAGCTCCTGGTGATGCTGGAAATGACCCCCAAGCGTGGAGACGTCTACACCTGCCAAGTGGAGCACTCCAGCCTTCAGAAACCTGTTGTCTTGGACTGGAAAGCACAGTCTGAATCTGCCCAGAGTAAGATGCTGAGTGGAGTCGGGGGCCTCGTGCTGGGCCTGATCTTCTTTGGGGTTGGCCTCTTTGTCTACAAGAGGAGTCAGAAAGGTGAGACACTCTGGGGAAAACCCTGA

>DAB_2_Hap_2

ATGGTGTGTGTGCTGTTCCCCGGGGGAGTCTGGACAGAGGTTCTGGCTGTGACCCTGCTGGTGCTGAATTCCCAGGTGGCTGCAGGCAGACATGCCCCAAAGCACTTCACGGAGCAGAAGTCCGACTGTCACTTCGAGAACGGGACGGAGCGCGTGCGGTTTGTGGAGAGATACATCCACAACCGGGAGGAGTACGTGCGCTTCGACAGCGACGTGGGGGAGTTTGTGGCGGTGACGGAGCTGGGACGGCCCGATGCTGAGCGTTGGAACAGCCAGAAGGAGATCCTGGAGGACGAACGGGCCCGGGGACACTTGTGCAGGCACAACTACGGGGTGTCTGAGCCCTTCTTAGTGCGCAGGAGCGTTGAGCCCGAGGTGATTGTGTATCCATCAAAGATGGCTCCCCTAGGACACCACAACCTGCTTGTCTGCTCTGTCAGTGGTTTCTATCCTGGGGACATTGAGGTCAGGTGGTTCCTGAATGGGCAGGAGGAGACGGCTGGGGTTGTGTCCACAGGCCTGATCAGCAATGGAGACTGGACCTACCAGCTCCTGGTGATGCTGGAAATGACCCCCAAGCGTGGAGACGTCTACACCTGCCAAGTGGAGCACTCCAGCCTTCAGAAACCTGTTGTCTTGGACTGGAAAGCACAGTCTGAATCTGCCCAGAGTAAGATGCTGAGTGGAGTCGGGGGCCTCGTGCTGGGCCTGATCTTCTTTGGGGTTGGCCTCTTTGTCTACAAGAGGAGTCAGAAAGGTGAGACACTCTGGGGAAAACCCTGA

>DAB_2_Hap_14

ATGGTGTGTGTGCTGTTCCCCGGGGGAGTCTGGACAGAGGTTCTGGCTGTGACCCTGCTGGTGCTGAATTCCCAGGTGGCTGCAGGCAGACATGCCCCAAAGCACTTCACTGAGTATAAGTCCGAGTGTTACTTCGGGAACGGGACGGAGCGCGTGCGGTTTGTGGAGAGACACATCTACAACCGGGAGGAGTATGTGCGCTTCGACAGCGACGTGGGGGAGTTTGTGGCGGTGACGGAGCTGGGACGGCCCGATGCTGAGCGTTGGAACAGCCAGAAGGAGATCCTGGAGGACGAACGGGCCCGGGGACACTTGTGCAGGCACAACTACGGGGTGTCTGAGCCCTTCTTAGTGCGCAGGAGCGTTGAGCCTGAGGTGATTGTGTATCCATCAAAGATGGCTCCCCTGGGACACCACAACCTGCTTGTCTGCTCTGTCAGTGGTTTCTATCCTGGGGACATTGAGGTCAGGTGGTTCCTGAATGGGCAGGAGGAGACGGCTGGGGTTGTGTCCACAGGCCTGATCAGCAATGGAGACTGGACCTACCAGCTCCTGGTGATGCTGGAAATGACCCCCAAGCGTGGAGACGTCTACACCTGCCAAGTGGAGCACTCCAGCCTTCAGAAACCTGTTGTCTTGGACTGGAAAGCACAGTCTGAATCTGCCCAGAGTAAGATGCTGAGTGGAGTCGGGGGCCTCGTGCTGGGCCTGATCTTCTTTGGGGTTGGCCTCTTTGTCTACAAGAGGAGTCAGAAAGGTGAGACACTCTGGGGAAAACCCTGA

>DAB_2_Hap_16

ATGGTGTGTGTGCTGTTCCCCGGGGGAGTCTGGACAGAGGTTCTGGCTGTGACCCTGCTGGTGCTGAATTCCCAGGTGGCTGCAGGCAGACATGCCCCAAAGCACTTCACTGAGTATAAGTCCGAGTGTTACTTCGGGAACGGGACGGAGCGCGTGCGGTTTGTGGAGAGATACATCTACAACCGGGAGGAGTATGTGCGCTTCGACAGCGACGTGGGGGAGTTTGTGGCGGTGACGGAGCTGGGACGGCCCGATGCTGAGCGTTGGAACAGCCAGAAGGAGATCCTGGAGGACGAACGGGCCCGGGGACACTTGTGCAGGCACAACTACGGGGTGTCTGAGCCCTTCTTAGTGCGCAGGAGCGTTGAGCCCGAGGTGATTGTGTATCCATCAAAGATGGCTCCCCTGGGACACCACAACCTGCTTGTCTGCTCTGTCAGTGGTTTCTATCCTGGGGACATTGAGGTCAGGTGGTTCCTGAATGGGCAGGAGGAGACGGCTGGGGTTGTGTCCACAGGCCTGATCAGCAATGGAGACTGGACCTACCAGCTCCTGGTGATGCTGGAAATGACCCCCAAGCGTGGAGACGTCTACACCTGCCAAGTGGAGCACTCCAGCCTTCAGAAACCTGTTGTCTTGGACTGGAAAGCACAGTCTGAATCTGCCCAGAGTAAGATGCTGAGTGGAGTCGGGGGCCTCGTGCTGGGCCTGATCTTCTTTGGGGTTGGCCTCTTTGTCTACAAGAGGAGTCAGAAAGGTGAGACACTCTGGGGAAAACCCTGA

>DAB_2_Hap_15

ATGGTGTGTGTGCTGTTCCCCGGGGGAGTCTGGACAGAGGTTCTGGCTGTGACCCTGCTGGTGCTGAATTCCCAGGTGGCTGCAGGCAGACATGCCCCAAAGCACTTCACTGAGTATAAGTCCGAGTGTTACTTCGGGAACGGGACGGAGCGCGTGCGGTTTGTGGAGAGACACATCTACAACCGGGAGGAGTATGCGCGCTTCGACAGCGACGTGGGGGAGTTTGTGGCGGTGACGGAGCTGGGACGGCCCGATGCTGAGCGTTGGAACAGCCAGAAGGAGATCCTGGAGGACGAACGGGCCCGGGGACACTTGTGCAGGCACAACTACGGGGTGTCTGAGCCCTTCTTAGTGCGCAGGAGCGTTGAGCCTGAGGTGATTGTGTATCCATCAAAGATGGCTCCCCTGGGACACCACAACCTGCTTGTCTGCTCTGTCAGTGGTTTCTATCCTGGGGACATTGAGGTCAGGTGGTTCCTGAATGGGCAGGAGGAGACGGCTGGGGTTGTGTCCACAGGCCTGATCAGCAATGGAGACTGGACCTACCAGCTCCTGGTGATGCTGGAAATGACCCCCAAGCGTGGAGACGTCTACACCTGCCAAGTGGAGCACTCCAGCCTTCAGAAACCTGTTGTCTTGGACTGGAAAGCACAGTCTGAATCTGCCCAGAGTAAGATGCTGAGTGGAGTCGGGGGCCTCGTGCTGGGCCTGATCTTCTTTGGGGTTGGCCTCTTTGTCTACAAGAGGAGTCAGAAAGGTGAGACACTCTGGGGAAAACCCTGA

>DAB_2_Hap_18

ATGGTGTGTGTGCTGTTCCCCGGGGGAGTCTGGACAGAGGTTCTGGCTGTGACCCTGCTGGTGCTGAATTCCCAGGTGGCTGCAGGCAGACATGCCCCAAAGCACTTCACTGAGTATAAGTCCGAGTGTTACTTCGGGAACGGGACGGAGCGCGTGCGGTTTGTGGAGAGACACATCTACAACCGGGAGGAGTATGTGCGCTTCGACAGCGACGTGGGGGAGTTTGTGGCGGTGACGGAGCTGGGACGGCCCGATGCTGAGCGTTGGAACAGCCAGAAGGAGATCCTGGAGGACGAACGGGCCCGGGGACACTTGTGCAGGCACAACTACGGGGTGTCTGAGCCCTTCTTAGTGCGCAGGAGCGTTGAGCCCGAGGTGATTGTGTATCCATCAAAGATGGCTCCCCTGGGACACCACAACCTGCTTGTCTGCTCTGTCAGTGGTTTCTATCCTGGGGACATTGAGGTCAGGTGGTTCCTGAATGGGCAGGAGGAGACGGCTGGGGTTGTGTCCACAGGCCTGATCAGCAATGGAGACTGGACCTACCAGCTCCTGGTGATGCTGGAAATGACCCCCAAGCGTGGAGACGTCTACACCTGCCAAGTGGAGCACTCCAGCCTTCAGAAACCTGTTGTCTTGGACTGGAAAGCACAGTCTGAATCTGCCCAGAGTAAGATGCTGAGTGGAGTCGGGGGCCTCGTGCTGGGCCTGATCTTCTTTGGGGTTGGCCTCTTTGTCTACAAGAGGAGTCAGAAAGGTGAGACACTCTGGGGAAAACCCTGA

>DAB_2_Hap_17

ATGGTGTGTGTGCTGTTCCCCGGGGGAGTCTGGACAGAGGTTCTGGCTGTGACCCTGCTGGTGCTGAATTCCCAGGTGGCTGCAGGCAGACATGCCCCAAAGCACTTCACTGAGTATAAGTCCGAGTGTTACTTCGGGAACGGGACGGAGCGCGTGCGGTTTGTGGAGAGACACATCTACAACCGGGAGGAGTACGCGCGCTTCGACAGCGACGTGGGGGAGTTTGTGGCGGTGACGGAGCTGGGACGGCCCGATGCTGAGCGTTGGAACAGCCAGAAGGAGATCCTGGAGGACGAACGGGCCCGGGGACACTTGTGCAGGCACAACTACGGGGTGTCTGAGCCCTTCTTAGTGCGCAGGAGCGTTGAGCCCGAGGTGATTGTGTATCCATCAAAGATGGCTCCCCTGGGACACCACAACCTGCTTGTCTGCTCTGTCAGTGGTTTCTATCCTGGGGACATTGAGGTCAGGTGGTTCCTGAATGGGCAGGAGGAGACGGCTGGGGTTGTGTCCACAGGCCTGATCAGCAATGGAGACTGGACCTACCAGCTCCTGGTGATGCTGGAAATGACCCCCAAGCGTGGAGACGTCTACACCTGCCAAGTGGAGCACTCCAGCCTTCAGAAACCTGTTGTCTTGGACTGGAAAGCACAGTCTGAATCTGCCCAGAGTAAGATGCTGAGTGGAGTCGGGGGCCTCGTGCTGGGCCTGATCTTCTTTGGGGTTGGCCTCTTTGTCTACAAGAGGAGTCAGAAAGGTGAGACACTCTGGGGAAAACCCTGA

>DAB_2_Hap_8

ATGGTGTGTGTGCTGTTCCCCGGGGGAGTCTGGACAGAGGTTCTGGCTGTGACCCTGCTGGTGCTGAATTCCCAGGTGGCTGCAGGCAGACATGCCCCAAAGCACTTCACTGAGTATAAGTCCGAGTGTTACTTCGGGAACGGGACGGAGCGCGTGCGGTTTGTGGAGAGACACATCTACAACCGGGAGGAGTATGCGCGCTTCGACAGCGACGTGGGGGAGTTTGTGGCGGTGACGGAGCTGGGACGGCCCGATGCTGAGCGTTGGAACAGCCAGAAGGAGATCCTGGAGGACGAACGGGCCCGGGGACACTTGTGCAGGCACAACTACGGGGTGTCTGAGCCCTTCTTAGTGCGCAGGAGCGTTGAGCCCGAGGTGATTGTGTATCCATCAAAGATGGCTCCCCTGGGACACCACAACCTGCTTGTCTGCTCTGTCAGTGGTTTCTATCCTGGGGACATTGAGGTCAGGTGGTTCCTGAATGGGCAGGAGGAGACGGCTGGGGTTGTGTCCACAGGCCTGATCAGCAATGGAGACTGGACCTACCAGCTCCTGGTGATGCTGGAAATGACCCCCAAGCGTGGAGACGTCTACACCTGCCAAGTGGAGCACTCCAGCCTTCAGAAACCTGTTGTCTTGGACTGGAAAGCACAGTCTGAATCTGCCCAGAGTAAGATGCTGAGTGGAGTCGGGGGCCTCGTGCTGGGCCTGATCTTCTTTGGGGTTGGCCTCTTTGTCTACAAGAGGAGTCAGAAAGGTGAGACACTCTGGGGAAAACCCTGA

>DAB_2_Hap_10

ATGGTGTGTGTGCTGTTCCCCGGGGGAGTCTGGACAGAGGTTCTGGCTGTGACCCTGCTGGTGCTGAATTCCCAGGTGGCTGCAGGCAGACATGCCCCAAAGCACTTCACGGAGCAGAAGTCCGACTGTCACTTCGAGAACGGGACGGAGCGCGTGCGGTTTGTGGAGAGATACATCCACAACCGGGAGGAGTACGTGCGCTTCGACAGCGACGTGGGGGAGTTTGTGGCGGTGACGGAGCTGGGACGGCCCGATGCTGAGCGTTGGAACAGCCAGAAGGAGATCCTGGAGGACGAACGGGCCCGGGGACACTTGTGCAGGCACAACTACGGGGTGTCTGAGCCCTTCTTAGTGCGCAGGAGCGTTGAGCCTGAGGTGATTGTGTATCCATCAAAGATGGCTCCCCTAGGACACCACAACCTGCTTGTCTGCTCTGTCAGTGGTTTCTATCCTGGGGACATTGAGGTCAGGTGGTTCCTGAATGGGCAGGAGGAGACGGCTGGGGTTGTGTCCACAGGCCTGATCAGCAATGGAGACTGGACCTACCAGCTCCTGGTGATGCTGGAAATGACCCCCAAGCGTGGAGACGTCTACACCTGCCAAGTGGAGCACTCCAGCCTTCAGAAACCTGTTGTCTTGGACTGGAAAGCACAGTCTGAATCTGCCCAGAGTAAGATGCTGAGTGGAGTCGGGGGCCTCGTGCTGGGCCTGATCTTCTTTGGGGTTGGCCTCTTTGTCTACAAGAGGAGTCAGAAAGGTGAGACACTCTGGGGAAAACCCTGA

>DAB_2_Hap_13

ATGGTGTGTGTGCTGTTCCCCGGGGGAGTCTGGACAGAGGTTCTGGCTGTGACCCTGCTGGTGCTGAATTCCCAGGTGGCTGCAGGCAGACATGCCCCAAAGCACTTCACTGAGTAGAAGTCCGAGTGTTACTTCGAGAACGGGACGGAGCGCGTGCGGTTTGTGGAGAGACACATCTACAACCGGGAGGAGTACGCGCGCTTCGACAGCGACGTGGGGGAGTTTGTGGCGGTGACGGAGCTGGGACGGCCCGATGCTGAGCGTTGGAACAGCCAGAAGGAGATCCTGGAGGACGAACGGGCCCGGGGACACTTGTGCAGGCACAACTACGGGGTGTCTGAGCCCTTCTTAGTGCGCAGGAGCGTTGAGCCCGAGGTGATTGTGTATCCATCAAAGATGGCTCCCCTAGGACACCACAACCTGCTTGTCTGCTCTGTCAGTGGTTTCTATCCTGGGGACATTGAGGTCAGGTGGTTCCTGAATGGGCAGGAGGAGACGGCTGGGGTTGTGTCCACAGGCCTGATCAGCAATGGAGACTGGACCTACCAGCTCCTGGTGATGCTGGAAATGACCCCCAAGCGTGGAGACGTCTACACCTGCCAAGTGGAGCACTCCAGCCTTCAGAAACCTGTTGTCTTGGACTGGAAAGCACAGTCTGAATCTGCCCAGAGTAAGATGCTGAGTGGAGTCGGGGGCCTCGTGCTGGGCCTGATCTTCTTTGGGGTTGGCCTCTTTGTCTACAAGAGGAGTCAGAAAGGTGAGACACTCTGGGGAAAACCCTGA

>DAB_3_Hap_1

ATGGCGTGTGTGCTGCTCCCCAGGGGCATCTGGACAGAGGTTCTGGCTGTGACCCTGCTGGTGCTGAATTCCCAGGTGGCTGCAGGCAGACATGCCCCAGAGCACTTCACGCAGCAGATGAAGTCCGACTGTTACTTCGAGAACGGGACGGAGCGCGTGCGGTTTGTGGAGAGATACATCCACAACCGGGAGGAGTACGTGCGCTTCGACAGCGACGTGGGGGAGTATGTGGCGGTGACGGAGCTGGGACGGCTCAGTGCTGAGCGTTTGAACAGCCAGAAGGAGATCCTGGAGGACAAACGGGCCCAGGTGGACACTTGTGCAGGCACAACTACGGGGTGTCTGAGCCCTTCTTACTGCGCAGGAGCGGTTGAGCCCGAGGTGATTGTGTATCCATCAAAGATGGCTCCCCTGGGACACCACAACCTGCTTGTCTGCTCTGTCAGTGGTTTCTATCCTGGGGACATTGAGGTCAGGTGGTTCCTGAATGGGCAGGAGGAGACGGCTGGGGTTGTGTCCACAGGCCTGATCAGCAATGGAGACTGGACCTACCAGCTCCTGGTGATGCTGGAAATGACCCCCAAGCGTGGAGACGTCTACACCTGCCAAGTGGAGCACTCCAGCCTTCAGAAACCTGTTGTCTTGGACTGGAAAGCACAGTCTGAATCTGCCCAGAGTAAGATGCTGAGTGGAGTCGGGGGCCTCGTGCTGGGCCTGATCTTCTTTGGGGTTGGCCTCTTTGTCTACAAGAGGAGTGAAAAAGGTGAGACACTCTGGGGAAAGCCCTGA

>DAB_3_Hap_3

ATGGCGTGTGTGCTGCTCCCCAGGGGCATCTGGACAGAGGTTCTGGCTGTGACCCTGCTGGTGCTGAATTCCCAGGTGGCTGCAGGCAGACATGCCCCAGAGCACTTCACGCAGCAGATGAAGTCCGACTGTCACTTCGAGAACGGGACGGAGCGCGTGCGGTTTGTGGAGAGATACATCCACAACCGGGAGGAGTACGTGCGCTTCGACAGCGACGTGGGGGAGTATGTGGCGGTGACGGAGCTGGGACGGCTCAGTGCTGAGCGTTTGAACAGCCAGAAGGAGATCCTGGAGGACAAACGGGCCCAGGTGGACACTTGTGCAGGCACAACTACGGGGTGTCTGAGCCCTTCTTACTGCGCAGGAGCGGTTGAGCCCGAGGTGATTGTGTATCCATCAAAGATGGCTCCCCTGGGACACCACAACCTGCTTGTCTGCTCTGTCAGTGGTTTCTATCCTGGGGACATTGAGGTCAGGTGGTTCCTGAATGGGCAGGAGGAGACGGCTGGGGTTGTGTCCACAGGCCTGATCAGCAATGGAGACTGGACCTACCAGCTCCTGGTGATGCTGGAAATGACCCCCAAGCGTGGAGACGTCTACACCTGCCAAGTGGAGCACTCCAGCCTTCAGAAACCTGTTGTCTTGGACTGGAAAGCACAGTCTGAATCTGCCCAGAGTAAGATGCTGAGTGGAGTCGGGGGCCTCGTGCTGGGCCTGATCTTCTTTGGGGTTGGCCTCTTTGTCTACAAGAGGAGTGAAAAAGGTGAGACACTCTGGGGAAAGCCCTGA

>DAB_3_Hap_4

ATGGCGTGTGTGCTGCTCCCCAGGGGCATCTGGACAGAGGTTCTGGCTGTGACCCTGCTGGTGCTGAATTCCCAGGTGGCTGCAGGCAGACATGCCCCAGAGCACTTCACGCAGCAGATGAAGTCCGACTGTTACTTCGAGAACGGGACGGAGCGCGTGCGGTTTGTGGAGAGATACATCCACAACCGGGAGGAGTACGTGCGCTTCGACAGCGACGTGGGGGAGTTTGTGGCGGTGACGGAGCTGGGACGGCTCAGTGCTGAGCGTTTGAACAGCCAGAAGGAGATCCTGGAGGACAAACGGGCCCAGGTGGACACTTGTGCAGGCACAACTACGGGGTGTCTGAGCCCTTCTTACTGCGCAGGAGCGGTTGAGCCCGAGGTGATTGTGTATCCATCAAAGATGGCTCCCCTGGGACACCACAACCTGCTTGTCTGCTCTGTCAGTGGTTTCTATCCTGGGGACATTGAGGTCAGGTGGTTCCTGAATGGGCAGGAGGAGACGGCTGGGGTTGTGTCCACAGGCCTGATCAGCAATGGAGACTGGACCTACCAGCTCCTGGTGATGCTGGAAATGACCCCCAAGCGTGGAGACGTCTACACCTGCCAAGTGGAGCACTCCAGCCTTCAGAAACCTGTTGTCTTGGACTGGAAAGCACAGTCTGAATCTGCCCAGAGTAAGATGCTGAGTGGAGTCGGGGGCCTCGTGCTGGGCCTGATCTTCTTTGGGGTTGGCCTCTTTGTCTACAAGAGGAGTGAAAAAGGTGAGACACTCTGGGGAAAGCCCTGA

>DAB_3_Hap_6

ATGGCGTGTGTGCTGCTCCCCAGGGGCATCTGGACAGAGGTTCTGGCTGTGACCCTGCTGGTGCTGAATTCCCAGGTGGCTGCAGGCAGACATGCCCCAGAGCACTTCACGGAGCAGATGAAGGCCGAGTGTTACTTCGAGAATGGGACGGAGCGCGTGCGGTTTGTGGAGAGATACATCCACAACCGGGAGGAGTTTGTGCGCTTTGACAGCGACGTGGGGGAGTATGTGGCGGTGACGGAGCTGGGGCGGCTCAGTGCTGAGCGTTTGAACAGCCAGAAGGAGATCCTGGACGACGAACGGGCCGCGGTGGACACTTCTGCAGGCACAACTACGAGGTGTCTGAGCCCTTCTTAGTGCGCAGGAGCGGTTGAGCCCGAGGTGATTGTGTATCCATCAAAGATGGCTCCCCTGGGACACCACAACCTGCTTGTCTGCTCTGTCAGTGGTTTCTATCCTGGGGACATTGAGGTCAGGTGGTTCCTGAATGGGCAGGAGGAGACGGCTGGGGTTGTGTCCACAGGCCTGATCAGCAATGGAGACTGGACCTACCAGCTCCTGGTGATGCTGGAAATGACCCCCAAGCGTGGAGACGTCTACACCTGCCAAGTGGAGCACTCCAGCCTTCAGAAACCTGTTGTCTTGGACTGGAAAGCACAGTCTGAATCTGCCCAGAGTAAGATGCTGAGTGGAGTCGGGGGCCTCGTGCTGGGCCTGATCTTCTTTGGGGTTGGCCTCTTTGTCTACAAGAGGAGTCAGAAAGGTGAGATACTCTGGGGAAAACCCTGA

>DAB_3_Hap_7

ATGGCGTGTGTGCTGCTCCCCAGGGGCATCTGGACAGAGGTTCTGGCTGTGACCCTGCTGGTGCTGAATTCCCAGGTGGCTGCAGGCAGACATGCCCCAGAGCACTTCACGCAGCAGATGAAGTCCGACTGTCACTTCGAGAACGGGACGGAGCGCGTGCGGTTTGTGGAGAGATACATCCACAACCGGGAGGAGTACGTGCGCTTCGACAGCGACGTGGGGGAGTTTGTGGCGGTGACGGAGCTGGGACGGCTCAGTGCTGAGCGTTTGAACAGCCAGAAGGAGATCCTGGAGGACAAACGGGCCCAGGTGGACACTTGTGCAGGCACAACTACGGGGTGTCTGAGCCCTTCTTACTGCGCAGGAGCGGTTGAGCCCGAGGTGATTGTGTATCCATCAAAGATGGCTCCCCTGGGACACCACAACCTGCTTGTCTGCTCTGTCAGTGGTTTCTATCCTGGGGACATTGAGGTCAGGTGGTTCCTGAATGGGCAGGAGGAGACGGCTGGGGTTGTGTCCACAGGCCTGATCAGCAATGGAGACTGGACCTACCAGCTCCTGGTGATGCTGGAAATGACCCCCAAGCGTGGAGACGTCTACACCTGCCAAGTGGAGCACTCCAGCCTTCAGAAACCTGTTGTCTTGGACTGGAAAGCACAGTCTGAATCTGCCCAGAGTAAGATGCTGAGTGGAGTCGGGGGCCTCGTGCTGGGCCTGATCTTCTTTGGGGTTGGCCTCTTTGTCTACAAGAGGAGTGAAAAAGGTGAGACACTCTGGGGAAAGCCCTGA

>DAB_3_Hap_2

ATGGCGTGTGTGCTGCTCCCCAGGGGCATCTGGACAGAGGTTCTGGCTGTGACCCTGCTGGTGCTGAATTCCCAGGTGGCTGCAGGCAGACATGCCCCAGAGCACTTCACGGAGCAGATGAAGGCCGAGTGTTACTTCGAGAATGGGACGGAGCGCGTGCGGTTTGTGCAGAGATACATCCACAACCGGGAGGAGTTTGCGCGCTTTGACAGCGACGTGGGGGAGTATGTGGCGGTGACGGAGCTGGGGCGGCTCAGTGCTGAGCGTTTGAACAGCCAGAAGGAGATCCTGGACGACGAACGGGCCGCGGTGGACACTTCTGCAGGCACAACTACGAGGTGTCTGAGCCCTTCTTAGTGCGCAGGAGCGGTTGAGCCCGAGGTGATTGTGTATCCATCAAAGATGGCTCCCCTGGGACACCACAACCTGCTTGTCTGCTCTGTCAGTGGTTTCTATCCTGGGGACATTGAGGTCAGGTGGTTCCTGAATGGGCAGGAGGAGACGGCTGGGGTTGTGTCCACAGGCCTGATCAGCAATGGAGACTGGACCTACCAGCTCCTGGTGATGCTGGAAATGACCCCCAAGCGTGGAGACGTCTACACCTGCCAAGTGGAGCACTCCAGCCTTCAGAAACCTGTTGTCTTGGACTGGAAAGCACAGTCTGAATCTGCCCAGAGTAAGATGCTGAGTGGAGTCGGGGGCCTCGTGCTGGGCCTGATCTTCTTTGGGGTTGGCCTCTTTGTCTACAAGAGGAGTCAGAAAGGTGAGATACTCTGGGGAAAACCCTGA

>DAB_3_Hap_12

ATGGCGTGTGTGCTGCTCCCCAGGGGCATCTGGACAGAGGTTCTGGCTGTGACCCTGCTGGTGCTGAATTCCCAGGTGGCTGCAGGCAGACATGCCCCAGAGCACTTCACGGAGCAGATGAAGTCCGACTGTTACTTCGAGAATGGGACGGAGCGCGTGCGGTTTGTGGAGAGATACATCCACAACCGGGAGGAGTACGTGCGCTTCGACAGCGACGTGGGGGAGTTTGTGGCGGTGACGGAGCTGGGACGGCTCAGTGCTGAGCGTTTGAACAGCCAGAAGGAGATCCTGGAGGACAAACGGGCCCAGGTGGACACTTGTGCAGGCACAACTACGGGGTGTCTGAGCCCTTCTTACTGCGCAGGAGCGGTTGAGCCCGAGGTGATTGTGTATCCATCAAAGATGGCTCCCCTGGGACACCACAACCTGCTTGTCTGCTCTGTCAGTGGTTTCTATCCTGGGGACATTGAGGTCAGGTGGTTCCTGAATGGGCAGGAGGAGACGGCTGGGGTTGTGTCCACAGGCCTGATCAGCAATGGAGACTGGACCTACCAGCTCCTGGTGATGCTGGAAATGACCCCCAAGCGTGGAGACGTCTACACCTGCCAAGTGGAGCACTCCAGCCTTCAGAAACCTGTTGTCTTGGACTGGAAAGCACAGTCTGAATCTGCCCAGAGTAAGATGCTGAGTGGAGTCGGGGGCCTCGTGCTGGGCCTGATCTTCTTTGGGGTTGGCCTCTTTGTCTACAAGAGGAGTGAAAAAGGTGAGACACTCTGGGGAAAGCCCTGA

>DAB_3_Hap_13

ATGGCGTGTGTGCTGCTCCCCAGGGGCATCTGGACAGAGGTTCTGGCTGTGACCCTGCTGGTGCTGAATTCCCAGGTGGCTGCAGGCAGACATGCCCCAGAGCACTTCACGGAGCAGATGAAGGCCGACTGTTACTTCGAGAACGGGACGGAGCGCGTGCGGTTTGTGGAGAGATACATCCACAACCGGGAGGAGTACGTGCGCTTCGACAGCGACGTGGGGGAGTTTGTGGCGGTGACGGAGCTGGGACGGCTCAGTGCTGAGCGTTTGAACAGCCAGAAGGAGATCCTGGAGGACAAACGGGCCCAGGTGGACACTTGTGCAGGCACAACTACGGGGTGTCTGAGCCCTTCTTACTGCGCAGGAGCGGTTGAGCCCGAGGTGATTGTGTATCCATCAAAGATGGCTCCCCTGGGACACCACAACCTGCTTGTCTGCTCTGTCAGTGGTTTCTATCCTGGGGACATTGAGGTCAGGTGGTTCCTGAATGGGCAGGAGGAGACGGCTGGGGTTGTGTCCACAGGCCTGATCAGCAATGGAGACTGGACCTACCAGCTCCTGGTGATGCTGGAAATGACCCCCAAGCGTGGAGACGTCTACACCTGCCAAGTGGAGCACTCCAGCCTTCAGAAACCTGTTGTCTTGGACTGGAAAGCACAGTCTGAATCTGCCCAGAGTAAGATGCTGAGTGGAGTCGGGGGCCTCGTGCTGGGCCTGATCTTCTTTGGGGTTGGCCTCTTTGTCTACAAGAGGAGTGAAAAAGGTGAGACACTCTGGGGAAAGCCCTGA

>DAB_3_Hap_14

ATGGCGTGTGTGCTGCTCCCCAGGGGCATCTGGACAGAGGTTCTGGCTGTGACCCTGCTGGTGCTGAATTCCCAGGTGGCTGCAGGCAGACATGCCCCAGAGCACTTCACGGAGCAGATGAAGTCCGACTGTTACTTCGAGAACGGGACGGAGCGCGTGCGGTTTGTGGAGAGATACATCCACAACCGGGAGGAGTACGTGCGCTTCGACAGCGACGTGGGGGAGTTTGTGGCGGTGACGGAGCTGGGACGGCTCAGTGCTGAGCGTTTGAACAGCCAGAAGGAGATCCTGGAGGACAAACGGGCCCAGGTGGACACTTGTGCAGGCACAACTACGGGGTGTCTGAGCCCTTCTTACTGCGCAGGAGCGGTTGAGCCCGAGGTGATTGTGTATCCATCAAAGATGGCTCCCCTGGGACACCACAACCTGCTTGTCTGCTCTGTCAGTGGTTTCTATCCTGGGGACATTGAGGTCAGGTGGTTCCTGAATGGGCAGGAGGAGACGGCTGGGGTTGTGTCCACAGGCCTGATCAGCAATGGAGACTGGACCTACCAGCTCCTGGTGATGCTGGAAATGACCCCCAAGCGTGGAGACGTCTACACCTGCCAAGTGGAGCACTCCAGCCTTCAGAAACCTGTTGTCTTGGACTGGAAAGCACAGTCTGAATCTGCCCAGAGTAAGATGCTGAGTGGAGTCGGGGGCCTCGTGCTGGGCCTGATCTTCTTTGGGGTTGGCCTCTTTGTCTACAAGAGGAGTGAAAAAGGTGAGACACTCTGGGGAAAGCCCTGA

>DAB_3_Hap_16

ATGGCGTGTGTGCTGCTCCCCAGGGGCATCTGGACAGAGGTTCTGGCTGTGACCCTGCTGGTGCTGAATTCCCAGGTGGCTGCAGGCAGACATGCCCCAGAGCACTTCACGCAGCAGATGAAGTCCGACTGTCACTTCGAGAACGGGACGGAGCGCGTGCGGTTTGTGGAGAGATACATCTACAACCGGGAGGAGTACGTGCGCTTCGACAGCGACGTGGGGGAGTTTGTGGCGGTGACGGAGCTGGGACGGCTCAGTGCTGAGCGTTTGAACAGCCAGAAGGAGATCCTGGAGGACAAACGGGCCCAGGTGGACACTTGTGCAGGCACAACTACGGGGTGTCTGAGCCCTTCTTACTGCGCAGGAGCGGTTGAGCCCGAGGTGATTGTGTATCCATCAAAGATGGCTCCCCTGGGACACCACAACCTGCTTGTCTGCTCTGTCAGTGGTTTCTATCCTGGGGACATTGAGGTCAGGTGGTTCCTGAATGGGCAGGAGGAGACGGCTGGGGTTGTGTCCACAGGCCTGATCAGCAATGGAGACTGGACCTACCAGCTCCTGGTGATGCTGGAAATGACCCCCAAGCGTGGAGACGTCTACACCTGCCAAGTGGAGCACTCCAGCCTTCAGAAACCTGTTGTCTTGGACTGGAAAGCACAGTCTGAATCTGCCCAGAGTAAGATGCTGAGTGGAGTCGGGGGCCTCGTGCTGGGCCTGATCTTCTTTGGGGTTGGCCTCTTTGTCTACAAGAGGAGTGAAAAAGGTGAGACACTCTGGGGAAAGCCCTGA

>DAB_3_Hap_10

ATGGCGTGTGTGCTGCTCCCCAGGGGCATCTGGACAGAGGTTCTGGCTGTGACCCTGCTGGTGCTGAATTCCCAGGTGGCTGCAGGCAGACATGCCCCAGAGCACTTCACGGAGCAGATGAAGGCCGAGTGTTACTTCGAGAATGGGACGGAGCGCGTGCGGTTTGTGCAGAGATACATCCACAACCGGGAGGAGTTTGCGCGCTTTGACAGCGACGTGGGGGAGTATGTGGCGGTGACGGAGCTGGGGCGGCTCAGTGCTGAGCGTTTGAACAGCCAGAAGGAGATCCTGGACGACGAACGGGCCGCGGTGGACACTTCTGCAGGCACAACTACGGGGTGTCTGAGCCCTTCTTAGTGCGCAGGAGCGGTTGAGCCCGAGGTGATTGTGTATCCATCAAAGATGGCTCCCCTGGGACACCACAACCTGCTTGTCTGCTCTGTCAGTGGTTTCTATCCTGGGGACATTGAGGTCAGGTGGTTCCTGAATGGGCAGGAGGAGACGGCTGGGGTTGTGTCCACAGGCCTGATCAGCAATGGAGACTGGACCTACCAGCTCCTGGTGATGCTGGAAATGACCCCCAAGCGTGGAGACGTCTACACCTGCCAAGTGGAGCACTCCAGCCTTCAGAAACCTGTTGTCTTGGACTGGAAAGCACAGTCTGAATCTGCCCAGAGTAAGATGCTGAGTGGAGTCGGGGGCCTCGTGCTGGGCCTGATCTTCTTTGGGGTTGGCCTCTTTGTCTACAAGAGGAGTCAGAAAGGTGAGATACTCTGGGGAAAACCCTGA

>DAB_3_Hap_18

ATGGCGTGTGTGCTGCTCCCCAGGGGCATCTGGACAGAGGTTCTGGCTGTGACCCTGCTGGTGCTGAATTCCCAGGTGGCTGCAGGCAGACATGCCCCAGAGCACTTCACGGAGCAGATGAAGGCCGAGTGTCACTTCGAGAACGGGACGGAGCGCGTGCGGTTTGTGGAGAGATACATCCACAACCGGGAGGAGTACGTGCGCTTCGACAGCGACGTGGGGGAGTTTGTGGCGGTGACGGAGCTGGGACGGCTCAGTGCTGAGCGTTTGAACAGCCAGAAGGAGATCCTGGAGGACAAACGGGCCCAGGTGGACACTTGTGCAGGCACAACTACGGGGTGTCTGAGCCCTTCTTACTGCGCAGGAGCGGTTGAGCCCGAGGTGATTGTGTATCCATCAAAGATGGCTCCCCTGGGACACCACAACCTGCTTGTCTGCTCTGTCAGTGGTTTCTATCCTGGGGACATTGAGGTCAGGTGGTTCCTGAATGGGCAGGAGGAGACGGCTGGGGTTGTGTCCACAGGCCTGATCAGCAATGGAGACTGGACCTACCAGCTCCTGGTGATGCTGGAAATGACCCCCAAGCGTGGAGACGTCTACACCTGCCAAGTGGAGCACTCCAGCCTTCAGAAACCTGTTGTCTTGGACTGGAAAGCACAGTCTGAATCTGCCCAGAGTAAGATGCTGAGTGGAGTCGGGGGCCTCGTGCTGGGCCTGATCTTCTTTGGGGTTGGCCTCTTTGTCTACAAGAGGAGTGAAAAAGGTGAGACACTCTGGGGAAAGCCCTGA

>DAB_3_Hap_5

ATGGCGTGTGTGCTGCTCCCCAGGGGCATCTGGACAGAGGTTCTGGCTGTGACCCTGCTGGTGCTGAATTCCCAGGTGGCTGCAGGCAGACATGCCCCAGAGCACTTCACGGAGCAGATGAAGGCCGAGTGTTACTTCGAGAATGGGACGGAGCGCGTGCGGTTTGTGGAGAGATACATCCACAACCGGGAGGAGTTTGCGCGCTTTGACAGCGACGTGGGGGAGTATGTGGCGGTGACGGAGCTGGGGCGGCTCAGTGCTGAGCGTTTGAACAGCCAGAAGGAGATCCTGGACGACGAACGGGCCGCGGTGGACACTTCTGCAGGCACAACTACGAGGTGTCTGAGCCCTTCTTAGTGCGCAGGAGCGGTTGAGCCCGAGGTGATTGTGTATCCATCAAAGATGGCTCCCCTGGGACACCACAACCTGCTTGTCTGCTCTGTCAGTGGTTTCTATCCTGGGGACATTGAGGTCAGGTGGTTCCTGAATGGGCAGGAGGAGACGGCTGGGGTTGTGTCCACAGGCCTGATCAGCAATGGAGACTGGACCTACCAGCTCCTGGTGATGCTGGAAATGACCCCCAAGCGTGGAGACGTCTACACCTGCCAAGTGGAGCACTCCAGCCTTCAGAAACCTGTTGTCTTGGACTGGAAAGCACAGTCTGAATCTGCCCAGAGTAAGATGCTGAGTGGAGTCGGGGGCCTCGTGCTGGGCCTGATCTTCTTTGGGGTTGGCCTCTTTGTCTACAAGAGGAGTCAGAAAGGTGAGATACTCTGGGGAAAACCCTGA

>DAB_3_Hap_21

ATGGCGTGTGTGCTGCTCCCCAGGGGCATCTGGACAGAGGTTCTGGCTGTGACCCTGCTGGTGCTGAATTCCCAGGTGGCTGCAGGCAGACATGCCCCAGAGCACTTCACGGAGCAGATGAAGTCCGACTGTCACTTCGAGAACGGGACGGAGCGCGTGCGGTTTGTGGAGAGATACATCCACAACCGGGAGGAGTACGTGCGCTTCGACAGCGACGTGGGGGAGTTTGTGGCGGTGACGGAGCTGGGACGGCTCAGTGCTGAGCGTTTGAACAGCCAGAAGGAGATCCTGGAGGACAAACGGGCCCAGGTGGACACTTGTGCAGGCACAACTACGGGGTGTCTGAGCCCTTCTTACTGCGCAGGAGCGGTTGAGCCCGAGGTGATTGTGTATCCATCAAAGATGGCTCCCCTGGGACACCACAACCTGCTTGTCTGCTCTGTCAGTGGTTTCTATCCTGGGGACATTGAGGTCAGGTGGTTCCTGAATGGGCAGGAGGAGACGGCTGGGGTTGTGTCCACAGGCCTGATCAGCAATGGAGACTGGACCTACCAGCTCCTGGTGATGCTGGAAATGACCCCCAAGCGTGGAGACGTCTACACCTGCCAAGTGGAGCACTCCAGCCTTCAGAAACCTGTTGTCTTGGACTGGAAAGCACAGTCTGAATCTGCCCAGAGTAAGATGCTGAGTGGAGTCGGGGGCCTCGTGCTGGGCCTGATCTTCTTTGGGGTTGGCCTCTTTGTCTACAAGAGGAGTGAAAAAGGTGAGACACTCTGGGGAAAGCCCTGA

>DAB_3_Hap_22

ATGGTGTGTGTGCTGCTCCCCAGGGGCATCTGGACAGAGGTTCTGGCTGTGACCCTGCTGGTGCTGAATTCCCAGGTGGCTGCAGGCAGACATGCCCCAGAGCACTTCACGCAGCAGATGAAGTCCGACTGTTACTTCGAGAACGGGACGGAGCGCGTGCGGTTTGTGGAGAGATACATCTACAACCGGGAGGAGTACGTGCGCTTCGACAGCGACGTGGGGGAGTATGTGGCGGTGACGGAGCTGGGGCGGCTCAGTGCTGAGCGTTTGAACAGCCAGAAGGAGATCCTGGACGACGAACGGGCCGCGGTGGACACTTCTGCAGGCACAACTACGAGGTGTCTGAGCCCTTCTTAGTGCGCAGGAGCGGTTGAGCCCGAGGTGATTGTGTATCCATCAAAGATGGCTCCCCTGGGACACCACAACCTGCTTGTCTGCTCTGTCAGTGGTTTCTATCCTGGGGACATTGAGGTCAGGTGGTTCCTGAATGGGCAGGAGGAGACGGCTGGGGTTGTGTCCACAGGCCTGATCAGCAATGGAGACTGGACCTACCAGCTCCTGGTGATGCTGGAAATGACCCCCAAGCGTGGAGACGTCTACACCTGCCAAGTGGAGCACTCCAGCCTTCAGAAACCTGTTGTCTTGGACTGGAAAGCACAGTCTGAATCTGCCCAGAGTAAGATGCTGAGTGGAGTCGGGGGCCTCGTGCTGGGCCTGATCTTCTTTGGGGTTGGCCTCTTTGTCTACAAGAGGAGTCAGAAAGGTGAGACACTCTGGGGAAAACCCTGA

>DAB_3_Hap_23

ATGGCGTGTGTGCTGCTCCCCAGGGGCATCTGGACAGAGGTTCTGGCTGTGACCCTGCTGGTGCTGAATTCCCAGGTGGCTGCAGGCAGACATGCCCCAGAGCACTTCACGGAGCAGATGAAGGCCGAGTGTTACTTCGAGAATGGGACGGAGCGCGTGCGGTTTGTGGAGAGATACATCCACAACCGGGAGGAGTACGTGCGCTTCGACAGCGACGTGGGGGAGTTTGTGGCGGTGACGGAGCTGGGACGGCTCAGTGCTGAGCGTTTGAACAGCCAGAAGGAGATCCTGGAGGACAAACGGGCCCAGGTGGACACTTGTGCAGGCACAACTACGGGGTGTCTGAGCCCTTCTTACTGCGCAGGAGCGGTTGAGCCCGAGGTGATTGTGTATCCATCAAAGATGGCTCCCCTGGGACACCACAACCTGCTTGTCTGCTCTGTCAGTGGTTTCTATCCTGGGGACATTGAGGTCAGGTGGTTCCTGAATGGGCAGGAGGAGACGGCTGGGGTTGTGTCCACAGGCCTGATCAGCAATGGAGACTGGACCTACCAGCTCCTGGTGATGCTGGAAATGACCCCCAAGCGTGGAGACGTCTACACCTGCCAAGTGGAGCACTCCAGCCTTCAGAAACCTGTTGTCTTGGACTGGAAAGCACAGTCTGAATCTGCCCAGAGTAAGATGCTGAGTGGAGTCGGGGGCCTCGTGCTGGGCCTGATCTTCTTTGGGGTTGGCCTCTTTGTCTACAAGAGGAGTGAAAAAGGTGAGACACTCTGGGGAAAGCCCTGA

>DAB_3_Hap_19

ATGGCGTGTGTGCTGCTCCCCAGGGGCATCTGGACAGAGGTTCTGGCTGTGACCCTGCTGGTGCTGAATTCCCAGGTGGCTGCAGGCAGACATGCCCCAGAGCACTTCACGGAGCAGATGAAGGCCGAGTGTTACTTCGAGAACGGGACGGAGCGCGTGCGGTTTGTGGAGAGATACATCCACAACCGGGAGGAGTACGTGCGCTTCGACAGCGACGTGGGGGAGTTTGTGGCGGTGACGGAGCTGGGACGGCTCAGTGCTGAGCGTTTGAACAGCCAGAAGGAGATCCTGGAGGACAAACGGGCCCAGGTGGACACTTGTGCAGGCACAACTACGGGGTGTCTGAGCCCTTCTTACTGCGCAGGAGCGGTTGAGCCCGAGGTGATTGTGTATCCATCAAAGATGGCTCCCCTGGGACACCACAACCTGCTTGTCTGCTCTGTCAGTGGTTTCTATCCTGGGGACATTGAGGTCAGGTGGTTCCTGAATGGGCAGGAGGAGACGGCTGGGGTTGTGTCCACAGGCCTGATCAGCAATGGAGACTGGACCTACCAGCTCCTGGTGATGCTGGAAATGACCCCCAAGCGTGGAGACGTCTACACCTGCCAAGTGGAGCACTCCAGCCTTCAGAAACCTGTTGTCTTGGACTGGAAAGCACAGTCTGAATCTGCCCAGAGTAAGATGCTGAGTGGAGTCGGGGGCCTCGTGCTGGGCCTGATCTTCTTTGGGGTTGGCCTCTTTGTCTACAAGAGGAGTGAAAAAGGTGAGACACTCTGGGGAAAGCCCTGA

>DAB_3_Hap_20

ATGGCGTGTGTGCTGCTCCCCAGGGGCATCTGGACAGAGGTTCTGGCTGTGACCCTGCTGGTGCTGAATTCCCAGGTGGCTGCAGGCAGACATGCCCCAGAGCACTTCACGGAGCAGATGAAGGCCGAGTGTTACTTCGAGAATGGGACGGAGCGCGTGCGGTTTGTGCAGAGATACATCCACAACCGGGAGGAGTTTGCGCGCTTTGACAGCGACGTGGGGGAGTATGTGGCGGTGACGGAGCTGGGGCGGCTCAGTGCTGAGCGTTTGAACAGCCAGAAGGAGATCCTGGACGACGAACGGGCCGCGGTGGACACTTCTGCAGGCACAACTACGAGGTGTCTGAGCCCTTCTTAGTGCGCAGGAGCGGTTGAGCCCGAGGTGATTGTGTATCCATCAAAGATGGCTCCCCTGGGACACCACAACCTGCTTGTCTGCTCTGTCAGTGGTTTCTATCCTGGGGACATTGAGGTCAGGTGGTTCCTGAATGGGCAGGAGGAGACGGCTGGGGTTGTGTCCACAGGCCTGATCAGCAATGGAGACTGGACCTACCAGCTCCTGGTGATGCTGGAAATGACCCCCAAGCGTGGAGACGTCTACACCTGCCAAGTGGAGCACTCCAGCCTTCAGAAACCTGTTGTCTTGGACTGGAAAGCACAGTCTGAATCTGCCCAGAGTAAGATGCTGAGTGGAGTCGGGGGCCTCGTGCTGGGCCTGATCTTCTTTGGGGTTGGCCTCTTTGTCTACAAGAGGAGTCAGAAAGGTGAGACACTCTGGGGAAAACCCTGA

>DAB_3_Hap_17

ATGGCGTGTGTGCTGCTCCCCAGGGGCATCTGGACAGAGGTTCTGGCTGTGACCCTGCTGGTGCTGAATTCCCAGGTGGCTGCAGGCAGACATGCCCCAGAGCACTTCACGGAGCAGATGAAGTCCGAGTGTTACTTCGAGAACGGGACGGAGCGCGTGCGGTTTGTGGAGAGATACATCCACAACCGGGAGGAGTACGTGCGCTTCGACAGCGACGTGGGGGAGTTTGTGGCGGTGACGGAGCTGGGACGGCTCAGTGCTGAGCGTTTGAACAGCCAGAAGGAGATCCTGGAGGACAAACGGGCCCAGGTGGACACTTGTGCAGGCACAACTACGGGGTGTCTGAGCCCTTCTTACTGCGCAGGAGCGGTTGAGCCCGAGGTGATTGTGTATCCATCAAAGATGGCTCCCCTGGGACACCACAACCTGCTTGTCTGCTCTGTCAGTGGTTTCTATCCTGGGGACATTGAGGTCAGGTGGTTCCTGAATGGGCAGGAGGAGACGGCTGGGGTTGTGTCCACAGGCCTGATCAGCAATGGAGACTGGACCTACCAGCTCCTGGTGATGCTGGAAATGACCCCCAAGCGTGGAGACGTCTACACCTGCCAAGTGGAGCACTCCAGCCTTCAGAAACCTGTTGTCTTGGACTGGAAAGCACAGTCTGAATCTGCCCAGAGTAAGATGCTGAGTGGAGTCGGGGGCCTCGTGCTGGGCCTGATCTTCTTTGGGGTTGGCCTCTTTGTCTACAAGAGGAGTGAAAAAGGTGAGACACTCTGGGGAAAGCCCTGA

>DAB_3_Hap_24

ATGGCGTGTGTGCTGCTCCCCAGGGGCATCTGGACAGAGGTTCTGGCTGTGACCCTGCTGGTGCTGAATTCCCAGGTGGCTGCAGGCAGACATGCCCCAGAGCACTTCACGGAGCAGATGAAGGCCGAGTGTCACTTCGAGAACGGGACGGAGCGCGTGCGGTTTGTGGAGAGATACATCTACAACCGGGAGGAGTTTGCGCGCTTCGACAGCGACGTGGGGGAGTATGTGGCGGTGACGGAGCTGGGGCGGCTCAGTGCTGAGCGTTTGAACAGCCAGAAGGAGATCCTGGAGGACAAACGGGCCCAGGTGGACACTTGTGCAGGCACAACTACGGGGTGTCTGAGCCCTTCTTAGTGCGCAGGAGCGGTTGAGCCCGAGGTGATTGTGTATCCATCAAAGATGGCTCCCCTGGGACACCACAACCTGCTTGTCTGCTCTGTCAGTGGTTTCTATCCTGGGGACATTGAGGTCAGGTGGTTCCTGAATGGGCAGGAGGAGACGGCTGGGGTTGTGTCCACAGGCCTGATCAGCAATGGAGACTGGACCTACCAGCTCCTGGTGATGCTGGAAATGACCCCCAAGCGTGGAGACGTCTACACCTGCCAAGTGGAGCACTCCAGCCTTCAGAAACCTGTTGTCTTGGACTGGAAAGCACAGTCTGAATCTGCCCAGAGTAAGATGCTGAGTGGAGTCGGGGGCCTCGTGCTGGGCCTGATCTTCTTTGGGGTTGGCCTCTTTGTCTACAAGAGGAGTCAGAAAGGTGAGATACTCTGGGGAAAACCCTGA

>DAB_3_Hap_15

ATGGTGTGTGTGCTGCTCCCCAGGGGCGTCTGGACAGAGGTTCTGGCTGTGACCCTGCTGGTGCTGAATTCCCAGGTGGCTGCAGGCAGACATGCCCCAAAGCACTTCACGGAGCAGATGAAGGCCGAGTGTCACTTCGAGAACGGGACGGAGCGCGTGCGGTTTGTGGAGAGATACATCCACAACCGGGAGGAGTACGTGCGCTTCGACAGCGACGTGGGGGAGTTTGTGGCGGTGACGGAGCTGGGACGGCTCAGTGCTGAGCGTTTGAACAGCCAGAAGGAGATCCTGGAGGACAAACGGGCCCAGGTGGACACTTGTGCAGGCACAACTACGGGGTGTCTGAGCCCTTCTTACTGCGCAGGAGCGGTTGAGCCCGAGGTGATTGTGTATCCATCAAAGATGGCTCCCCTGGGACACCACAACCTGCTTGTCTGCTCTGTCAGTGGTTTCTATCCTGGGGACATTGAGGTCAGGTGGTTCCTGAATGGGCAGGAGGAGACGGCTGGGGTTGTGTCCACAGGCCTGATCAGCAATGGAGACTGGACCTACCAGCTCCTGGTGATGCTGGAAATGACCCCCAAGCGTGGAGACGTCTACACCTGCCAAGTGGAGCACTCCAGCCTTCAGAAACCTGTTGTCTTGGACTGGAAAGCACAGTCTGAATCTGCCCAGAGTAAGATGCTGAGTGGAGTCGGGGGCCTCGTGCTGGGCCTGATCTTCTTTGGGGTTGGCCTCTTTGTCTACAAGAGGAGTGAAAAAGGTGAGACACTCTGGGGAAAGCCCTGA

>DAB_3_Hap_8

ATGGTGTGTGTGCTGCTCCCCAGGGGCGTCTGGACAGAGGTTCTGGCTGTGACCCTGCTGGTGCTGAATTCCCAGGTGGCTGCAGGCAGACATGCCCCAAAGCACTTCACGGAGCAGATGAAGGCCGAGTGTCACTTCGAGAACGGGACGGAGCGCGTGCGGTTTGTGGAGAGATACATCTACAACCGGGAGGAGTACGTGCGCTTCGACAGCGACGTGGGGGAGTTTGTGGCGGTGACGGAGCTGGGACGGCTCAGTGCTGAGCGTTTGAACAGCCAGAAGGAGATCCTGGAGGACAAACGGGCCCAGGTGGACACTTGTGCAGGCACAACTACGGGGTGTCTGAGCCCTTCTTACTGCGCAGGAGCGGTTGAGCCCGAGGTGATTGTGTATCCATCAAAGATGGCTCCCCTGGGACACCACAACCTGCTTGTCTGCTCTGTCAGTGGTTTCTATCCTGGGGACATTGAGGTCAGGTGGTTCCTGAATGGGCAGGAGGAGACGGCTGGGGTTGTGTCCACAGGCCTGATCAGCAATGGAGACTGGACCTACCAGCTCCTGGTGATGCTGGAAATGACCCCCAAGCGTGGAGACGTCTACACCTGCCAAGTGGAGCACTCCAGCCTTCAGAAACCTGTTGTCTTGGACTGGAAAGCACAGTCTGAATCTGCCCAGAGTAAGATGCTGAGTGGAGTCGGGGGCCTCGTGCTGGGCCTGATCTTCTTTGGGGTTGGCCTCTTTGTCTACAAGAGGAGTGAAAAAGGTGAGACACTCTGGGGAAAGCCCTGA

>DAB_3_Hap_9

ATGGTGTGTGTGCTGCTCCCCAGGGGCGTCTGGACAGAGGTTCTGGCTGTGACCCTGCTGGTGCTGAATTCCCAGGTGGCTGCAGGCAGACATGCCCCAAAGCACTTCACGGAGCAGATGAAGGCCGAGTGTCACTTCGAGAACGGGACGGAGCGCGTGCGGTTTGTGGAGAGATACATCCACAACCGGGAGGAGTACGCGCGCTTCGACAGCGACGTGGGGGAGTTTGTGGCGGTGACGGAGCTGGGACGGCTCAGTGCTGAGCGTTTGAACAGCCAGAAGGAGATCCTGGAGGACAAACGGGCCCAGGTGGACACTTGTGCAGGCACAACTACGGGGTGTCTGAGCCCTTCTTACTGCGCAGGAGCGGTTGAGCCCGAGGTGATTGTGTATCCATCAAAGATGGCTCCCCTGGGACACCACAACCTGCTTGTCTGCTCTGTCAGTGGTTTCTATCCTGGGGACATTGAGGTCAGGTGGTTCCTGAATGGGCAGGAGGAGACGGCTGGGGTTGTGTCCACAGGCCTGATCAGCAATGGAGACTGGACCTACCAGCTCCTGGTGATGCTGGAAATGACCCCCAAGCGTGGAGACGTCTACACCTGCCAAGTGGAGCACTCCAGCCTTCAGAAACCTGTTGTCTTGGACTGGAAAGCACAGTCTGAATCTGCCCAGAGTAAGATGCTGAGTGGAGTCGGGGGCCTCGTGCTGGGCCTGATCTTCTTTGGGGTTGGCCTCTTTGTCTACAAGAGGAGTGAAAAAGGTGAGACACTCTGGGGAAAGCCCTGA

>DAB_3_Hap_11

ATGGTGTGTGTGCTGCTCCCCAGGGGCGTCTGGACAGAGGTTCTGGCTGTGACCCTGCTGGTGCTGAATTCCCAGGTGGCTGCAGGCAGACATGCCCCAAAGCACTTCACGCAGCAGATGAAGTCCGACTGTCACTTCGAGAACGGGACGGAGCGCGTGCGGTTTGTGGAGAGATACATCTACAACCGGGAGGAGTACGTGCGCTTCGACAGCGACGTGGGGGAGTTTGTGGCGGTGACGGAGCTGGGACGGCTCAGTGCTGAGCGTTTGAACAGCCAGAAGGAGATCCTGGAGGACAAACGGGCCCAGGTGGACACTTGTGCAGGCACAACTACGGGGTGTCTGAGCCCTTCTTACTGCGCAGGAGCGGTTGAGCCCGAGGTGATTGTGTATCCATCAAAGATGGCTCCCCTGGGACACCACAACCTGCTTGTCTGCTCTGTCAGTGGTTTCTATCCTGGGGACATTGAGGTCAGGTGGTTCCTGAATGGGCAGGAGGAGACGGCTGGGGTTGTGTCCACAGGCCTGATCAGCAATGGAGACTGGACCTACCAGCTCCTGGTGATGCTGGAAATGACCCCCAAGCGTGGAGACGTCTACACCTGCCAAGTGGAGCACTCCAGCCTTCAGAAACCTGTTGTCTTGGACTGGAAAGCACAGTCTGAATCTGCCCAGAGTAAGATGCTGAGTGGAGTCGGGGGCCTCGTGCTGGGCCTGATCTTCTTTGGGGTTGGCCTCTTTGTCTACAAGAGGAGTGAAAAAGGTGAGACACTCTGGGGAAAGCCCTGA

>DAB_3_Hap_25

ATGGCGTGTGTGCTGCTCCCCAGGGGCATCTGGACAGAGGTTCTGGCTGTGACCCTGCTGGTGCTGAATTCCCAGGTGGCTGCAGGCAGACATGCCCCAGAGCACTTCACGGAGCAGATGAAGGCCGAGTGTCACTTCGAGAACGGGACGGAGCGCGTGCGGTTTGTGGAGAGATACATCTACAACCGGGAGGAGTTTGCGCGCTTCGACAGCGACGTGGGGGAGTATGTGGCGGTGACGGAGCTGGGGCGGCTCAGTGCTGAGCGTTTGAACAGCCAGAAGGAGATCCTGGAGGACGAACGGGCCGCGGTGGACACTTGTGCAGGCACAACTACGGGGTGTCTGAGCCCTTCTTAGTGCGCAGGAGCGGTTGAGCCCGAGGTGATTGTGTATCCATCAAAGATGGCTCCCCTGGGACACCACAACCTGCTTGTCTGCTCTGTCAGTGGTTTCTATCCTGGGGACATTGAGGTCAGGTGGTTCCTGAATGGGCAGGAGGAGACGGCTGGGGTTGTGTCCACAGGCCTGATCAGCAATGGAGACTGGACCTACCAGCTCCTGGTGATGCTGGAAATGACCCCCAAGCGTGGAGACGTCTACACCTGCCAAGTGGAGCACTCCAGCCTTCAGAAACCTGTTGTCTTGGACTGGAAAGCACAGTCTGAATCTGCCCAGAGTAAGATGCTGAGTGGAGTCGGGGGCCTCGTGCTGGGCCTGATCTTCTTTGGGGTTGGCCTCTTTGTCTACAAGAGGAGTCAGAAAGGTGAGATACTCTGGGGAAAACCCTGA

>DAB_4_Hap_1

ATGGTGTGTGTGCTGTTCCCCAGGGGAGTCTGGACAGAGGTTCTGGCTGTGACCCTGCTGGTGCTGAATTCCCAGGTGGCTGCAGGCAGACATGCCCCAAAGCACTTCACCGAGCAGGCTAAGTCCGAGTATCACTTTGAGAACAGGAGGGAGCACGTGCGGTTTGTGGATAGATACATCCACAACCGCGAGGAGTTTATGCGCTTCGACAGCTACCTGGGGGAGTATGAGGCGCTGACGGAGCTGGGGCGGCCCAGTGCTGAGTATTATAACAGCCGCAAGGAGATTCTGGAGCAGAGACGAGCGGCAGTGGACTGGTTTTGCAGGGTCTGCTACAAGGTGTCTGAGCTCTTTTTAGTGCACAGGAGCGTTGAGCCCGAGGTGATTGTGCATCCATCAAAGATGGCTCCCCTGGGACACCACAATCTGCTTATCTGCTCTGTCAGTGGTTTCTATCCTGGGGACATTGAGGTCAGCCTGATCAGCAATGGAGACTGGACCTACCAGCTCCTGGTGATGCTGGAAATGACCCCCAAGCATGGAGATGTCTACACCTGCCAAGTGGAGCACTCCAGCCTTCAAAGACCTGTCATGTTGGATTGGAAAGCACAGTCTGAATCCGCCCAGAGTAAGATGCTGAGTGGAGTGGGAGTCCTAGTGCTGGGCCTGATCTGCGTTTGGGGTTGGCCTCATTGTCCACAAGAAGAATCAGAAAGGTGA

>DAB_4_Hap_2

ATGGTGTGTGTGCTGTTCCCCAAGGGAGTCTGGACAGAGGTTCTGGCTGTGACCCTGCTGGTGCTGAATTCCCAGGTGGCTGCAGGCAGACATGCCCCAAAGCACTTCACCGAGCAGGCTAAGTCCGAGTATCACTTTGAGAACAGGAGGGAGCACGTGCGGTTTGTGGATAGATACATCCACAACCGCGAGGAGTTTATGCGCTTCGACAGCTACCTGGGGGAGTATGAGGCGCTGACGGAGCTGGGGCGGCCCAGTGCTGAGTATTATAACAGCCGCAAGGAGATTCTGGAGCAGAGACGAGCGGCAGTGGACTGGTTTTGCAGGGTCTGCTACAAGGTGTCTGAGCTCTTTTTAGTGCACAGGAGCGTTGAGCCCGAGGTGATTGTGCATCCATCAAAGATGGCTCCCCTGGGACACCACAATCTGCTTATCTGCTCTGTCAGTGGTTTCTATCCTGGGGACATTGAGGTCAGCCTGATCAGCAATGGAGACTGGACTTACCAGCTCCTGGTGATGCTGGAAATGACCCCCAAGCATGGAGATGTCTACACCTGCCAAGTGGAGCACTCCAGCCTTCAAAGACCTGTCATGTTGGATTGGAAAGCACAGTCTGAATCCGCCCAGAGTAAGATGCTGAGTGGAGTGGGAGTCCTAGTGCTGGGCCTGATCTGCGTTTGGGGTTGGCCTCATTGTCCACAAGAAGAGTCAGAAAGGTGA

>DAB_4_Hap_3

ATGGTGTGTGTGCTGTTCCCCAGGGGAGTCTGGACAGAGGTTCTGGCTGTGACCCTGCTGGTGCTGAATTCCCAGGTGGCTGCAGGCAGACATGCCCCAAAGCACTTCACCGAGCAGGCTAAGTCCGAGTATCACTTTGAGAACAGGAGGGAGCACGTGCGGTTTGTGGATAGATACATCCACAACCGCGAGGAGTTTATGCGCTTCGACAGCTACCTGGGGGAGTATGAGGCGCTGACGGAGCTGGGGCGGCCCAGTGCTGAGTATTATAACAGCCGCAAGGAGATTCTGGAGCAGAGACGAGCGGCAGTGGACTGGTTTTGCAGGGTCTGCTACAAGGTGTCTGAGCTCTTTTTAGTGCACAGGAGCGTTGAGCCCGAGGTGATTGTGCATCCATCAAAGATGGCTCCCCTGGGACACCACAATCTGCTTATCTGCTCTGTCAGTGGTTTCTATCCTGGGGACATTGAGGTCAGCCTGATCAGCAATGGAGACTGGACCTACCAGCTCCTGGTGATGCTGGAAATGACCCCCAAGCATGGAGATGTCTACACCTGCCAAGTGGAGCACTCCAGCCTTCAAAGACCTGTCATGTTGGATTGGAAAGCACAGTCTGAATCCGCCCAGAGTAAGATGCTGAGTGGAGTGGGAGTCCTAGTGCTGGGCCTGATCTGCGTTTGGGGTTGGCCTCATTGTCCACAAGAAGAGTCAGAAAGGTGA

>DAB_5_Hap_1

ATGCATTCGAGGAGGTGTCCATCAGGGCTGACTCGCCCCTGGGGTGTACCCCCAGAGTACTTCACGAGGCTGTTAAAGTTCGAGTGTTACTTTGAGAACGGGACGGAGCACGTGCGGTATGTGGAGAGACACATCCACAACCGGGAGGAGCTCATGCGCTTTGACAGCGACGTGGGAGAGTATGTGGTGCTGATGGAGCTGGGGCTGCGCGAAGCTGAGCATAGGAACAGTCAGAGAGAGATCCTGGAGAACGAACGGGCAGTGGTGGACACTTACTGCAGGCCCAACTACGAGGCTGCTGAGATCTTCTTACTGGGCAGGAGCTTTGAGCCCGAGGTGATTGTGTATCCATCAAAGATGGCTCCTCTGGGACACCACAACCTGCTTGTCTGCTCTGTCAGTGGTTTCTATCCTGGGAACATTGAGGTCAGGTCGTTACTGAATGGGCAGGAGGAGACAGCTGGGGTTGTGTCCACAGGCCTGATCAGCAATGGAGACTGGACTTACCAGATCCTGGTGATGCTGGAAATGACCCCCAAGCGTGGAGATGTCTACACCTGCCAAGTGGAGCACTCCAGCCTTCAAAGACCTGCCATCTTGGACTGGAAAGCACAGTCTGAATCTGCCCAGAATAAAATGCTGAGTAGAGTCGGGGGCCTCCTGCTGGGCCTGATGTTCTTTGGGGTTGGCCTCATTGTCCACAACAGGAGTCAGAAAGGAAGTCATGGTTCACAACCAACAGGTAAGATTCCTCTACCCAAGTTGCGTCAGGGTTCTCTTAGCTACAGTCTCTGCCCAGTGGGGCCTGAAGCTAAGAAGGGACTTGAGGGCCCTTAG

>DAB_5_Hap_2

ATGCATTCGAGGAGGTGTCCATCAGGGCTGACTCGCCCCTGGGGTGTACCCCCAGAGTACTTCACGAGGCTGTTAAAGTTCGAGTGTTACTTTGAGAACGGGACGGAGCACGTGCGGTATGTGGAGAGACACATCCACAACCGGGAGGAGCTCATGCGCTTTGACAGCGACGTGGGAGAGTATGTGGTGCTGATGGAGCTGGGGCTGCGCGAAGCTGAGCATAGGAACAGTCAGAGAGAGATCCTGGAGAACGAACGGGCAGTGGTGGACACTTACTGCAGGCCCAACTACGAGGCTGCTGAGATCTTCTTACTGGGCAGGAGCTTTGAGCCCGAGGTGATTGTGTATCCATCAAAGATGGCTCCTCTGGGACACCACAACCTGCTTGTCTGCTCTGTCAGTGGTTACTATCCTGGGAACATTGAGGTCAGGTCGTTACTGAATGGGCAGGAGGAGACAGCTGGGGTTGTGTCCACAGGCCTGATCAGCAATGGAGACTGGACTTACCAGATCCTGGTGATGCTGGAAATGACCCCCAAGCGTGGAGATGTCTACACCTGCCAAGTGGAGCACTCCAGCCTTCAAAGACCTGCCATCTTGGACTGGAAAGCACAGTCTGAATCTGCCCAGAATAAAATGCTGAGTAGAGTCGGGGGCCTCCTGCTGGGCCTGATGTTCTTTGGGGTTGGCCTCATTGTCCACAACAGGAGTCAGAAAGGAAGTCATGGTTCACAACCAACAGGTAAGATTCCTCTACCCAAGTTGCGTCAGGGTTCTCTTAGCTACAGTCTCTGCCCAGTGGGGCCTGAAGCTAAGAAGGGACTTGAGGGCCCTTAG

>DAB_5_Hap_3

ATGCATTCGAGGAGGTGTCCATCAGGGCTGACTCGCCCCTGGAGTGTACCCCCAGAGTACTTCACGAGGCTGTTAAAGTTCGAGTGTTACTTTGAGAACGGGACGGAGCACGTGCGGTATGTGGAGAGACACATCCACAACCGGGAGGAGCTCATGCGCTTTGACAGCGACGTGGGAGAGTATGTGGTGCTGATGGAGCTGGGGCTGCGCGAAGCTGAGCATAGGAACAGTCAGAGAGAGATCCTGGAGAACGAACGGGCAGTGGTGGACACTTACTGCAGGCCCAACTACGAGGCTGCTGAGATCTTCTTACTGGGCAGGAGCTTTGAGCCCGAGGTGATTGTGTATCCATCAAAGATGGCTCCTCTGGGACACCACAACCTGCTTGTCTGCTCTGTCAGTGGTTTCTATCCTGGGAACATTGAGGTCAGGTCGTTACTGAATGGGCAGGAGGAGACAGCTGGGGTTGTGTCCACAGGCCTGATCAGCAATGGAGACTGGACTTACCAGATCCTGGTGATGCTGGAAATGACCCCCAAGCGTGGAGATGTCTACACCTGCCAAGTGGAGCACTCCAGCCTTCAAAGACCTGCCATCTTGGACTGGAAAGCACAGTCTGAATCTGCCCAGAATAAAATGCTGAGTAGAGTCGGGGGCCTCCTGCTGGGCCTGATGTTCTTTGGGGTTGGCCTCATTGTCCACAACAGGAGTCAGAAAGGAAGTCATGGTTCACAACCAACAGGTAAGATTCCTCTACCCAAGTTGCGTCAGGGTTCTCTTAGCTACAGTCTCTGCCCAGTGGGGCCTGAAGCTAAGAAGGGACTTGAGGGCCCTTAG

>DBA_1_Hap_1

ATGGCTGCCAACAGAGTCCTGATCCTAGGGACCCTCACACTGACCTCATTGTTGAGTCCCCAAGGAGCTTCCGAATCCATTGAAGCTGACCACGTGGGGGTCCATGGCACACATATGTACCAGTCCTATGGGCCCTCAGGCCAGTATACACACGAATTTGATGGAGATGAGTTGTTTTATGTGGACCTGCAGAAGAAGGAGACTGTATGGCGGCTGCCAGAGTTTAGCCACTTTGCCAGCTTTGACCCTCAGGGAGGGCTGAGAAACATAGACATAGCCAAGCACAACCTGGACATCCTGATCAAACGCTCCAACAGAACCAGGGCCATCAGTGTGCCTCCTGAGGTGACTGTGTTCTCAGAGAGTCCCGTGGAGATGGGTCAGCCGAACATACTCATCTGCTTGGTGGACAACATCTTTCCCCCAGTGGTCAACATCACGTGGCTTCGTAATGGACAGTTGGTCACTGTAGGTGTGTCTGAGACAGACTTCTACCCTCGGTCTGACTACAAGTTCCGCAAGTTCCACTACCTCACTTTTCTCCCTAACACAGAAGACTTTTATGACTGCAAAGTGGAGCACTGGGGCCTGGAGCAGCCAGTTCTCAAGCACTGGGAGCCCCAGGTTCCATCCCCACTGCCAGAGACAACAGAAACTGTGGTCTGTGCCCTTGGTCTGGCTGTGGGCCTGGTGGGCATCGTTGTGGGCACCATCCTCATAATCAGGGGCATGCGTTCCAGCAGCAGGATCCAACATCAAGGGCCTCTGTGA

>DBA_1_Hap_4

ATGGCTGCCAACAGAGTCCTGATCCTAGGGACCCTCACACTGACCTCATTGTTGAGTCCCCAAGGAGCTTCCGAATCCATTGAAGCTGACCACGTGGGGGTCCATGGCACACATATGTACCAGTCCTATGGGCCCTCAGGCCAGTATACACACGAATTTGATGGAGATGAGTTGTTTTATGTGGACCTGCAGAAGAAGGAGACTGTATGGCGGCTGCCAGAGTTTAGCCACTTTGCCAGCTTTGACCCTCAGGGAGGGCTGAGAAACATAGACATAGCCAAGCACAACCTGGACATCCTGATCAAACGCTCCAACAGAACCAGGGCCATCAGTGTGCCTCCTGAGGTGACTGTGTTCTCAGAGAGTCCCGTGGAGATGGGTCAGCCGAACATACTCATCTGCTTGGTGGACAACATCTTTCCCCCAGTGGTCAACATCACATGGCTTCGTAATGGACAGTTGGTCACTGTAGGTGTGTCTGAGACAGACTTCTACCCTCGGTCTGACTACAAGTTCCGCAAGTTCCACTACCTCACTTTTCTCCCTAACACAGAAGACTTTTATGACTGCAAAGTGGAGCACTGGGGCCTGGAGCAGCCAGTTCTCAAGCACTGGGAGCCCCAGGTTCCATCCCCACTGCCAGAGACAACAGAAACTGTGGTCTGTGCCCTTGGTCTGGCTGTGGGCCTGGTGGGCATCGTTGTGGGCACCATCCTCATAATCAGGGGCATGCGTTCCAGCAGCAGGATCCAACATCAAGGGCCTCTGTGA

>DBA_1_Hap_7

ATGGCTGCCAACAGAGTCCTGATCCTAGGGACCCTCACACTGACCTCATTGTTGAGTCCCCAAGGAGCTTCCGAATCCATTGAAGCTGACCACGTGGGGGTCCATGGCACACATATGTACCAGTCCTATGGGCCCTCAGGCCAGTATACACAAGAATTTGGTGGAGATGAGTTGTTTTATGTGGACCTGCAGAAGAAGGAGACTGTATGGCGGCTGCCAGAGTTTAGCCACTTTGAAAGCTTTGACCCTCAGGGAGGGCTGAGAAACATAGCCATACTCAAGCACAACCTGGACATCCTGATCAAACGCTCCAACAGAACCAGGGCCATCAGTGTGCCTCCTGAGGTGACTGTGTTCTCAGAGAGTCCCGTGGAGATGGGTCAGCCGAACATACTCATCTGCTTGGTGGACAACATCTTTCCCCCAGTGGTCAACATCACGTGGCTTCGTAATGGACAGTTGGTCACTGTAGGTGTGTCTGAGACAGACTTCTACCCTCGGTCTGACTACAAGTTCCGCAAGTTCCACTACCTCACTTTTCTCCCTAACACAGAAGACTTTTATGACTGCAAAGTGGAGCACTGGGGCCTGGAGCAGCCAGTTCTCAAGCACTGGGAGCCCCAGGTTCCATCCCCACTGCCAGAGACAACAGAAACTGTGGTCTGTGCCCTTGGTCTGGCTGTGGGCCTGGTGGGCATCGTTGTGGGCACCATCCTCATAATCAGGGGCATGCGTTCCAGCAGCAGGATCCAACATCAAGGGCCTCTGTGA

>DBA_1_Hap_2

ATGGCTGCCAACAGAGTCCTGATCCTAGGGACCCTCACACTGACCTCATTGTTGAGTCCCCAAGGAGCTTCCGAATCCATTGAAGCTGACCACGTGGGGGTCCATGGCACACATATGTACCAGTCCTATGGGCCCTCAGGCCAGTATACACAAGAATTTGGTGGAGATGAGTTGTTTTATGTGGACCTGCAGAAGAAGGAGACTGTATGGCGGCTGCCAGAGTTTAGCCACTTTGAAAGCTTTGACCCTCAGGGAGGGCTGAGAAACATAGCCATACTCAAGCACAACCTGGACATCCTGATCAAACACTCCAATAGAACCAGGGCCATCAGTGTGCCTCCTGAGGTGACTGTGTTCTCAGAGAGTCCCGTGGAGATGGGTCAGCCGAACATACTCATCTGCTTGGTGGACAACATCTTTCCCCCAGTGGTCAACATCACGTGGCTTCGTAATGGACAGTTGGTCACTGTAGGTGTGTCTGAGACAGACTTCTACCCTCGGTCTGACTACAAGTTCCGCAAGTTCCACTACCTCACTTTTCTCCCTAACACAGAAGACTTTTATGACTGCAAAGTGGAGCACTGGGGCCTGGAGCAGCCAGTTCTCAAGCACTGGGAGCCCCAGGTTCCATCCCCACTGCCAGAGACAACAGAAACTGTGGTCTGTGCCCTTGGTCTGGCTGTGGGCCTGGTGGGCATCGTTGTGGGCACCATCCTCATAATCAGGGGCATGCGTTCCAGCAGCAGGATCCAACATCAAGGGCCTCTGTGA

>DBA_1_Hap_5

ATGGCTGCCAACAGAGTCCTGATCCTAGGGACCCTCACACTGACCTCATTGTTGAGTCCCCAAGGAGCTTCCGAATCCATTGAAGCTGACCACGTGGGGGTCTATGGCACACATATGTACCAGTCCTATGGGCCCTCAGGCCAGTATACACACGAATTTGATGGAGATGAGTTGTTTTATGTGGACCTGCAGAAGAAGGAGACTGTATGGCGGCTGCCAGAGTTTAGCCACTTTGAAAGCTTTGACCCTCAGGGAGGGCTGAGAAACATAGCCATACTCAAGCACAACCTGGACATCCTGATCAAACGCTCCAACAGAACCAGGGCCATCAGTGTGCCTCCTGAGGTGACTGTATTCTCAGAGAGTCCCATGGAGATGGGTCAGCCGAACATACTCATCTGCTTGGTGGACAACATCTTTCCCCCAGTGGTCAACATCACGTGGCTTCATAATGGACAGTTGGTCACTGTAGGTGTGTCTGAGACAGACTTCTACCCTCGGTCTGACTACAAGTTCCGCAAGTTCCACTACCTCACTTTTCTCCCTAACACAGAAGACTTTTATGACTGCAAAGTGGAGCACTGGGGCCTGGAGCAGCCAGTCCTCAAGCACTGGGAGCCCCAGGTTTCATCCCCACTGCCAGAGACAACAGAAACTGTGGTCTGTGCCCTTGGTCTGGCTGTGGGCCTGGTGGGCATCGTTGTGGGCACCATCCTCATAATCAAGGGCATGCGTTCCAGCAGCAGGATCCAACATCAAGGGCCTCTGTGA

>DBA_1_Hap_10

ATGGCTGCCAACAGAGTCCTGATCCTAGGGACCCTCACACTGACCTCATTGTTGAGTCCCCAAGGAGCTTCCGAATCCATTGAAGCTGACCACGTGGGGGTCCATGGCACACATATGTACCAGTCCTATGGGCCCTCAGGCCAGTATACACACGAATTTGATGGAGATGAGTTGTTTTATGTGGACCTGCAGAAGAAGGAGACTGTATGGCGGCTGCCAGAGTTTAGCCACTTTGAAAGCTTTGACCCTCAGGGAGGGCTGAGAAACATAGCCATACTCAAGCACAACCTGGACATCCTGATCAAACGCTCCAACAGAACCAGGGCCATCAGTGTGCCTCCTGAGGTGACTGTGTTCTCAGAGAGTCCCGTGGAGATGGGTCAGCCGAACATACTCATCTGCTTGGTGGACAACATCTTTCCCCCAGTGGTCAACATCACGTGGCTTCGTAATGGACAGTTGGTCACTGTAGGTGTGTCTGAGACAGACTTCTACCCTCGGTCTGACTACAAGTTCCGCAAGTTCCACTACCTCACTTTTCTCCCTAACACAGAAGACTTTTATGACTGCAAAGTGGAGCACTGGGGCCTGGAGCAGCCAGTTCTCAAGCACTGGGAGCCCCAGGTTCCATCCCCACTGCCAGAGACAACAGAAACTGTGGTCTGTGCCCTTGGTCTGGCTGTGGGCCTGGTGGGCATCGTTGTGGGCACCATCCTCATAATCAGGGGCATGCGTTCCAGCAGCAGGATCCAACATCAAGGGCCTCTGTGA

>DBA_1_Hap_8

ATGGCTGCCAACAGAGTCCTGATCCTAGGGACCCTCACACTGACCTCATTGTTGAGTCCCCAAGGAGCTTCCGAATCCATTGAAGCTGACCACGTGGGGGTCCATGGCACACATATGTACCAGTCCTATGGGCCCTCAGGCCAGTATACACACGAATTTGATGGAGATGAGTTGTTTTATGTGGACCTGCAGAAGAAGGAGACTGTATGGCGGCTGCCAGAGTTTAGCCACTTTGAAAGCTTTGACCCTCAGGGAGGGCTGAGAAACATAGCCATACTCAAGCACAACCTGGACATCCTGATCAAACACTCCAATAGAACCAGGGCCATCAGTTTGCCTCCTGAGGTGACTGTGTTCTCAGAGAGTCCCGTGGAGATGGGTCAGCCGAACATACTCATCTGCTTGGTGGACAACATCTTTCCCCCAGTGGTCAACATCACGTGGCTTCGTAATGGACAGTTGGTCACTGTAGGTGTGTCTGAGACAGACTTCTACCCTCGGTCTGACTACAAGTTCCGCAAGTTCCACTACCTCACTTTTCTCCCTAACACAGAAGACTTTTATGACTGCAAAGTGGAGCACTGGGGCCTGGAGCAGCCAGTTCTCAAGCACTGGGAGCCCCAGGTTCCATCCCCACTGCCAGAGACAACAGAAACTGTGGTCTGTGCCCTTGGTCTGGCTGTGGGCCTGGTGGGCATCGTTGTGGGCACCATCCTCATAATCAGGGGCATGCGTTCCAGCAGCAGGATCCAACATCAAGGGCCTCTGTGA

>DBA_1_Hap_6

ATGGCTGCCAACAGAGTCCTGATCCTAGGGACCCTCACACTGACCTCATTGTTGAGTCCCCAAGGAGCTTCCGAATCCATTGAAGCTGACCACGTGGGGGTCCATGGCACACATATGTACCAGTCCTATGGGCCCTCAGGCCAGTATACACAAGAATTTGGTGGAGATGAGTTGTTTTATGTGGACCTGCAGAAGAAGGAGACTGTATGGCGGCTGCCAGAGTTTAGCCACTTTGAAAGCTTTGACCCTCAGGGAGGGCTGAGAAACATAGCCATACTCAAGCACAACCTGGACATCCTGATCAAACACTCCAATAGAACCAGGGCCATCAGTTTGCCTCCTGAGGTGACTGTGTTCTCAGAGAGTCCCGTGGAGATGGGTCAGCCGAACATACTCATCTGCTTGGTGGACAACATCTTTCCCCCAGTGGTCAACATCACGTGGCTTCGTAATGGACAGTTGGTCACTGTAGGTGTGTCTGAGACAGACTTCTACCCTCGGTCTGACTACAAGTTCCGCAAGTTCCACTACCTCACTTTTCTCCCTAACACAGAAGACTTTTATGACTGCAAAGTGGAGCACTGGGGCCTGGAGCAGCCAGTTCTCAAGCACTGGGAGCCCCAGGTTCCATCCCCACTGCCAGAGACAACAGAAACTGTGGTCTGTGCCCTTGGTCTGGCTGTGGGCCTGGTGGGCATCGTTGTGGGCACCATCCTCATAATCAGGGGCATGCGTTCCAGCAGCAGGATCCAACATCAAGGGCCTCTGTGA

>DBA_1_Hap_3

ATGGCTGCCAACAGAGTCCTGATCCTAGGGACCCTCACACTGACCTCATTGTTGAGTCCCCAAGGAGCTTCCGAATCCATTGAAGCTGACCACGTGGGGGTCTATGGCACACATATGTACCAGTCCTATGGGCCCTCAGGCCAGTATACACACGAATTTGATGGAGATGAGTTGTTTTATGTGGACCTGCAGAAGAAGGAGACTGTATGGCGGCTGCCAGAGTTTAGCCACTTTGAAAGCTTTGACCCTCAGGGAGGGCTGAGAAACATAGCCATACTCAAGCACAACCTGGACATCCTGATCAAACGCTCCAACAGAACCAGGGCCATCAGTGTGCCTCCTGAGGTGACTGTGTTCTCAGAGAGTCCCGTGGAGATGGGTCAGCCGAACATACTCATCTGCTTGGTGGACAACATCTTTCCCCCAGTGGTCAACATCACATGGCTTCGTAATGGACAGTTGGTCACTGTAGGTGTGTCTGAGACAGACTTCTACCCTCGGTCTGACTACAAGTTCCGCAAGTTCCACTACCTCACTTTTCTCCCTAACACAGAAGACTTTTATGACTGCAAAGTGGAGCACTGGGGCCTGGAGCAGCCAGTTCTCAAGCACTGGGAGCCCCAGGTTCCATCCCCACTGCCAGAGACAACAGAAACTGTGGTCTGTGCCCTTGGTCTGGCTGTGGGCCTGGTGGGCATCGTTGTGGGCACCATCCTCATAATCAAGGGCATGCGTTCCAGCAGCAGGATCCAACATCAAGGGCCTCTGTGA

>DBA_1_Hap_9

ATGGCTGCCAACAGAGTCCTGATCCTAGGGACCCTCACACTGACCTCATTGTTGAGTCCCCAAGGAGCTTCCGAATCCATTGAAGCTGACCACGTGGGGGTCTATGGCACACATATGTACCAGTCCTATGGGCCCTCAGGCCAGTATACACAAGAATTTGGTGGAGATGAGTTGTTTTATGTGGACCTGCAGAAGAAGGAGACTGTATGGCGGCTGCCAGAGTTTAGCCACTTTGAAAGCTTTGACCCTCAGGGAGGGCTGAGAAACATAGCCATACTCAAGCACAACCTGGACATCCTGATCAAACACTCCAATAGAACCAGGGCCATCAGTTTGCCTCCTGAGGTGACTGTATTCTCAGAGAGTCCCATGGAGATGGGTCAGCCGAACATACTCATCTGCTTGGTGGACAACATCTTTCCCCCAGTGGTCAACATCACGTGGCTTCATAATGGACAGTTGGTCACTGTAGGTGTGTCTGAGACAGACTTCTACCCTCGGTCTGACTACAAGTTCCGCAAGTTCCACTACCTCACTTTTCTCCCTAACACAGAAGACTTTTATGACTGCAAAGTGGAGCACTGGGGCCTGGAGCAGCCAGTCCTCAAGCACTGGGAGCCCCAGGTTTCATCCCCACTGCCAGAGACAACAGAAACTGTGGTCTGTGCCCTTGGTCTGGCTGTGGGCCTGGTGGGCATCGTTGTGGGCACCATCCTCATAATCAAGGGCATGCGTTCCAGCAGCAGGATCCAACATCAAGGGCCTCTGTGA

>DBA_1_Hap_11

ATGGCTGCCAACAGAGTCCTGATCCTAGGGACCCTCACACTGACCTCATTGTTGAGTCCCCAAGGAGCTTCCGAATCCATTGAAGCTGACCACGTGGGGGTCTATGGCACACATATGTACCAGTCCTATGGGCCCTCAGGCCAGTATACACAAGAATTTGGTGGAGATGAGTTGTTTTATGTGGACCTGCAGAAGAAGGAGACTGTATGGCGGCTGCCAGAGTTTAGCCACTTTGAAAGCTTTGACCCTCAGGGAGGGCTGAGAAACATAGCCATACTCAAGCACAACCTGGACATCCTGATCAAACACTCCAATAGAACCAGGGCCATCAGTTTGCCTCCTGAGGTGACTGTGTTCTCAGAGAGTCCCGTGGAGATGGGTCAGCCGAACATACTCATCTGCTTGGTGGACAACATCTTTCCCCCAGTGGTCAACATCACATGGCTTCGTAATGGACAGTTGGTCACTGTAGGTGTGTCTGAGACAGACTTCTACCCTCGGTCTGACTACAAGTTCCGCAAGTTCCACTACCTCACTTTTCTCCCTAACACAGAAGACTTTTATGACTGCAAAGTGGAGCACTGGGGCCTGGAGCAGCCAGTTCTCAAGCACTGGGAGCCCCAGGTTCCATCCCCACTGCCAGAGACAACAGAAACTGTGGTCTGTGCCCTTGGTCTGGCTGTGGGCCTGGTGGGCATCGTTGTGGGCACCATCCTCATAATCAAGGGCATGCGTTCCAGCAGCAGGATCCAACATCAAGGGCCTCTGTGA

>DBA_2_Hap_1

ATGGCTGCCAACAGAGTCCTGATCCTAGGGACCCTCACACTGACCTCATTGTTGAGTCCCCAAGGAGCTTCCGAATCCATTGAAGCTGACCATGTGGGGGTCTACTGCACAGGTGTGTACCAGTCCTGTGGGCCCTCAGGCCAGTATACACAAGAATTTGGTGGAGATGAGTTGTTTTATGTGGACCTGCAGAAGAAGGAGACTATATGGCGGCTGCCAGAGTTTAGCCACTTTGAAAGCTTTGACCCTCAGGGAGGGCTGAGGGAAAGAGCCATACTCAAGCACAACCTGGACATCCTGATCAAACACTCCAATAGAACCAGGGCCATCAAGCCCCCTGAGATGACTGTATTCTCAGAGAGTCCCTTGGAGATGGATGAGCTGAACATACTCATCTGCTTGGTGGACAACAACTTCCCCCCAGTGGTCAACATCATGTGGTTTCGTAATGGGCCGTTTGTCACTGTAGGTGCATCTGAGACAGACTTCTACCCTCGGTCTTACTACAAATTCCACAAGCTCCACTACTTCACTTTTCTCCCAAGCACAGAAGACTTTTATGACTGCAAAGTGGAGCACTGGGGCCTGGAGCAGCCAGTCCTCAAGCACTGGGATCCCCAGGTTTCATCCCCACTGCCAGAGACAACAGAAACTGTGGTCTGTGCCCTTGGTCTGGCTGTGGGCCTGGTGGGCATCGTTGTGGGCACCATCCTCATAATCAGGGGCATGCATTCCAGCAGTAGGATCCAACATCAAGGACCTCTGTGA

>DBA_2_Hap_3

ATGGCTGCCAACAGAGTCCTGATCCTAGGGACCCTCACACTGACCTCATTGTTGAGTCCCCAAGGAGCTTCCGAATCCATTGAAGCTGACCATGTGGGGGTCTACTGCACAGGTGTGTACCAGTCCTGTGGGCCCTCAGGCCAGTATACACAAGAATTTGGTGGAGATGAGTTGTTTTATGTGGACCTGCAGAAGAAGGAGACCATGTGGCGGCTGCCAGAGTTTAGCCACTTTGAAAGCTTTGACCCTCAGGGAGGGCTGAGGGAAAGAGCCATACTCAAGCACAACCTGGACATCCTGATCAAACACTCCAATAGAACCAGGGCCATCAAGCCCCCTGAGATGACTGTATTCTCAGAGAGTCCCTTGGAGATGGATGAGCTGAACATACTCATCTGCTTGGTGGACAACAACTTCCCCCCAGTGGTCAACATCATGTGGTTTCGTAATGGGCCGTTTGTCACTGTAGGTGCATCTGAGACAGACTTCTACCCTCGGTCTTACTACAAATTCCACAAGCTCCACTACTTCACTTTTCTCCCAAGCACAGAAGACTTTTATGACTGCAAAGTGGAGCACTGGGGCCTGGAGCAGCCAGTCCTCAAGCACTGGGATCCCCAGGTTTCATCCCCACTGCCAGAGACAACAGAAACTGTGGTCTGTGCCCTTGGTCTGGCTGTGGGCCTGGTGGGCATCGTTGTGGGCACCATCCTCATAATCAGGGGCATGCATTCCAGCAGTAGGATCCAACATCAAGGACCTCTGTGA

>DBA_2_Hap_4

ATGGCTGCCAACAGAGTCCTGATCCTAGGGACCCTCACACTGATCTCCTTGTTGAGTCCCCAAGGAGCTTCTGAATCCATTTAAGCTGACCATGTGGGGGTCTACTGCACAGGTGTGTACCAGTCCTATGGGCCCTCAGGCCAGTATACACAAGAATTTGGTGGAGATGAGTTGTTTTATGTGGACCTGCAGAAGAAGGAGACTATATGGCGGCTGCCAGAGTTTAACCACTTTGCTGGCTTTGACCCTCAGGGAGGGCTGAGGGAAAGAGCCACAGTCAAGCACAACCTGGAAATCCTGATCAAACACTCCAATAGAACCAGGGCCATCAAGCCCCCTGAGGTGACTGTATTCTCAGAGAGTCCCTTGGAGATGGATGAGCTGAACATACTCATCTGCTTGGTGGACAACAACTTCCCCCCAGTGGTCAACATCATGTGGTTTCGTAATGGGCCGTTTGTCACTGTAGGTGCATCTGAGACAGACTTCTACCCTCGGTCTTACTACAAATTCCACAAGCTCCACTACTTCACTTTTCTCCCAAGCACAGAAGACTTTTATGACTGCAAAGTGGAGCACTGGGGCCTGGAGCAGCCAGTTCTCAAGCACTGGGATCCCCAGGTTTCATCCCCACTGCCAGAGACAACAGAAACTGTGGTCTGTGCCCTTGGTCTGGCTGTGGGCCTGGTGGGCATCGTTGTGGGCACCATCCTCATAATCAGGGGCATGCATTCCAGCAGTAGGATCCAACATCAAGGACCTCTGTGA

>DBA_2_Hap_2

ATGGCTGCCAACAGAGTCCTGATCCTAGGGACCCTCACACTGACCTCATTGTTGAGTCCCCAAGGAGCTTCCGAATCCATTGAAGCTGACCATGTGGGGGTCTACTGCACAGGTGTGTACCAGTCCTATGGGCCCTCAGGCCAGTATACACAAGAATTTGGTGGAGATGAGTTGTTTTATGTGGACCTGCAGAAGAAGGAGACCATGTGGTGGCTGCCAGAATTTAACCACTTTGCTGGCTTTGACCCTCAGGGAGGGCTGAGGGAAAGAGCCACAGTCAAGCACAACCTGGAAATCCTGATCAAACACTCCAATAGAACCAGGGCCATCAAGCCCCCTGAGGTGACTGTATTATCAGAGAGTCCCTTGGAGATGGATGAGCTGAACATACTCATCTGCTTGGTGGACAACAACTTCCCCCCAGTGGTCAACATCACGTGGTTTCGTAATGGGCCGTTTGTCACTGTAGGTGCATCTGAGACAGACTTCTACCCTCGGTCTGACTACAAATTCCACAAGCTCCACTACTTCACTTTTCTCCCAAGCACAGAAGACTTTTATGACTGCAAAGTGGAGCACTGGGGCCTGGAGCAGCCAGTTCTCAAGCACTGGGATCCCCAGGTTTCATTCCCACTGCCAAAGACAACAGAAACTGTGGTCTGTGCCCTTGGTCTGGCTGTGGGCCTGGTGGGCATCGTTGTGGGCACCATCCTCATAATCAGGGGCATGCATTCCAGCAGTAGGATCCAACATCAAGGACCTCTGTGA

>DBA_2_Hap_5

ATGGCTGCCAACAGAGTCCTGATCCTAGGGACCCTCACACTGACCTCATTGTTGAGTCCCCAAGGAGCTTCCGAATCCATTGAAGCTGACCATGTGGGGGTCTACTGCACAGGTGTGTACCAGTCCTATGGGCCCTCAGGCCAGTATACACAAGAATTTGGTGGAGATGAGTTGTTTTATGTGGACCTGCAGAAGAAGGAGACCATGTGGTGGCTGCCAGAATTTAACCACTTTGCTGGCTTTGACCCTCAGGGAGGGCTGAGGGAAAGAGCCACAGTCAAGCACAACCTGGAAATCCTGATCAAACACTCCAATAGAACCAGGGCCATCAAGCCCCCTGAGGTGACTGTATTATCAGAGAGTCCCTTGGAGATGGATGAGCTGAACATACTCATCTGCTTGGTGGACAACAACTTCCCCCCAGTGGTCAACATCACGTGGTTTCGTAATGGGCCGTTTGTCACTGTAGGTGCATCTGAGACAGACTTCTACCCTCGGTCTGACTACAAATTCCACAAGCTCCACTACTTCACTTTTCTCCCAAGCACAGAAGACTTTTATGACTGCAAAGTGGAGCACTGGGGCCTGGAGCAGCCAGTCCTCAAGCACTGGGATCCCCAGGTTTCATTCCCACTGCCAAAGACAACAGAAACTGTGGTCTGTGCCCTTGGTCTGGCTGTGGGCCTGGTGGGCATCGTTGTGGGCACCATCCTCATAATCAGGGGCATGCATTCCAGCAGTAGGATCCAACATCAAGGACCTCTGTGA

>DBA_2_Hap_6

ATGGCTGCCAACAGAGTCCTGATCCTAGGGACCCTCACACTGATCTCCTTGTTGAGTCCCCAAGGAGCTTCTGAATCCATTTAAGCTGACCATGTGGGGGTCTACTGCACAGGTGTGTACCAGTCCTATGGGCCCTCAGGCCAGTATACACAAGAATTTGGTGGAGATGAGTTGTTTTATGTGGACCTGCAGAAGAAGGAGACTATATGGCGGCTGCCAGAGTTTAACCACTTTGCTGGCTTTGACCCTCAGGGAGGGCTGAGGGAAAGAGCCACAGTCAAGCACAACCTGGAAATCCTGATCAAACACTCCAATAGAACCAGGGCCATCAAGCCCCCTGAGGTGACTGTATTATCAGAGAGTCCCTTGGAGATGGATGAGCTGAACATACTCATCTGCTTGGTGGACAACAACTTCCCCCCAGTGGTCAACATCACGTGGTTTCGTAATGGGCCGTTTGTCACTGTAGGTGCATCTGAGACAGACTTCTACCCTCGGTCTGACTACAAATTCCACAAGCTCCACTACTTCACTTTTCTCCCAAGCACAGAAGACTTTTATGACTGCAAAGTGGAGCACTGGGGCCTGGAGCAGCCAGTTCTCAAGCACTGGGATCCCCAGGTTTCATCCCCACTGCCAGAGACAACAGAAACTGTGGTCTGTGCCCTTGGTCTGGCTGTGGGCCTGGTGGGCATCGTTGTGGGCACCATCCTCATAATCAGGGGCATGCATTCCAGCAGTAGGATCCAACATCAAGGACCTCTGTGA

>DBA_2_Hap_7

ATGGCTGCCAACAGAGTCCTGATCCTAGGGACCCTCACACTGACCTCATTGTTGAGTCCCCAAGGAGCTTCCGAATCCATTGAAGCTGACCATGTGGGGGTCTACTGCACAGGTGTGTACCAGTCCTGTGGGCCCTCAGGCCAGTATACACAAGAATTTGGTGGAGATGAGTTGTTTTATGTGGACCTGCAGAAGAAGGAGACCATGTGGCGGCTGCCAGAGTTTAGCCACTTTGAAAGCTTTGACCCTCAGGGAGGGCTGAGGGAAAGAGCCACACTCAAGCACAACCTGGACATCCTGATCAAACACTCCAATAGAACCAGGGCCATCAAGCCCCCTGAGATGACTGTATTCTCAGAGAGTCCCTTGGAGATGGATGAGCTGAACATACTCATCTGCTTGGTGGACAACAACTTCCCCCCAGTGGTCAACATCATGTGGTTTCGTAATGGGCCGTTTGTCACTGTAGGTGCATCTGAGACAGACTTCTACCCTCGGTCTTACTACAAATTCCACAAGCTCCACTACTTCACTTTTCTCCCAAGCACAGAAGACTTTTATGACTGCAAAGTGGAGCACTGGGGCCTGGAGCAGCCAGTCCTCAAGCACTGGGATCCCCAGGTTTCATCCCCACTGCCAGAGACAACAGAAACTGTGGTCTGTGCCCTTGGTCTGGCTGTGGGCCTGGTGGGCATCGTTGTGGGCACCATCCTCATAATCAGGGGCATGCATTCCAGCAGTAGGATCCAACATCAAGGACCTCTGTGA

>DBA_2_Hap_8

ATGGCTGCCAACAGAGTCCTGATCCTAGGGACCCTCACACTGACCTCATTGTTGAGTCCCCAAGGAGCTTCCGAATCCATTGAAGCTGACCATGTGGGGGTCTACTGCACAGGTGTGTACCAGTCCTGTGGGCCCTCAGGCCAGTATACACAAGAATTTGGTGGAGATGAGTTGTTTTATGTGGACCTGCAGAAGAAGGAGACCATGTGGTGGCTGCCAGAATTTAACCACTTTGCTGGCTTTGACCCTCAGGGAGGGCTGAGGGAAAGAGCCATACTCAAGCACAACCTGGACATCCTGATCAAACACTCCAATAGAACCAGGGCCATCAAGCCCCCTGAGATGACTGTATTCTCAGAGAGTCCCTTGGAGATGGATGAGCTGAACATACTCATCTGCTTGGTGGACAACAACTTCCCCCCAGTGGTCAACATCATGTGGTTTCGTAATGGGCCGTTTGTCACTGTAGGTGCATCTGAGACAGACTTCTACCCTCGGTCTTACTACAAATTCCACAAGCTCCACTACTTCACTTTTCTCCCAAGCACAGAAGACTTTTATGACTGCAAAGTGGAGCACTGGGGCCTGGAGCAGCCAGTCCTCAAGCACTGGGATCCCCAGGTTTCATCCCCACTGCCAGAGACAACAGAAACTGTGGTCTGTGCCCTTGGTCTGGCTGTGGGCCTGGTGGGCATCGTTGTGGGCACCATCCTCATAATCAGGGGCATGCATTCCAGCAGTAGGATCCAACATCAAGGACCTCTGTGA

>DBB_2_Hap_1

ATGGTTGATGTTTGGATCTCTGCTGGCTGCGGGAAGATTGGTCTGTTAATGACATCGATGGTGTTGAGTCTACCTTCTTCTTGGGCCAGGGAGATCCCAGAGGATTTCGTGCTTCAGTTCAAGGCTTACTGTTACTTCACCAACGGCACGCAGCTGGTGCGGCATGTGTGTAGATATATCTACAATGATGAGGAATACGCTCACTTCGACAGCGACGTGGGGGAGTTCGTGGCTGTGACGGAGCTGGGGCGGCCCGATGCTAAGTACTGGAACAGTCAGAAGGAAATCCTGGAGGAACAACGAGCCATTGTGGACACGGTGTGCCGACACAACTACGAGATAGATAAGCCCTTCACGGTGGACAGAAGAGTCCAGCCCAGAGTGACCATCTCCCCCTCCAAGACAGAGGCCCTGCAGCACCTGCTGGTCTGCTCTGTCACTGGCTTCTATCCAAGCAAGATCAAGGTCACCTGGCTCAAGAATGGGCAGGAGGAGACAGCTGGGGTTGTGTCCACGGGTGTGATACAACATGGAGACTGGACCTACCAGATCCTAGTCATGTTGGAAATGATTCCCCAGAGCAGAGACATCTACACCTGCAGTGTGGAGCATGCCAGCCTGCAGAGCCCCATCAGTGTGGAATGGAGAGCACAGTCTGACTCTGCCCAGAGCAAATTGCTGAGTGGAATTGGAGGCTTTGTCCTGGGGCTGATATTGCTAGGTGTAGGACTGTTCATCCACCTCAAGAACCAGAAAGGTAAGGAGCATGTCGGGGAGGCCATGGGGCTCTGGCTTTGGCCCCAGCTCCTGCCCCTTATCCTGTAG

>DBB_2_Hap_2

ATGGTTGATGTTTGGATCTCTGCTGGCTGCGGGAAGATTGGTCTGTTAATGACATCGATGGTGTTGAGTCTACCTTCTTCTTGGGCCAGGGAGATCCCAGAGGATTTCGTGCTTCAGTTCAAGGCTTACTGTTACTTCACCAACGGCACGCAGCTGGTGCGGCATGTGTGTAGATATATCTACAATGATGAGGAATACGCTCACTTCGACAGCGACGTGGGGGAGTTCGTGGCTGTGACGGAGCTGGGGCGGCCCGATGCTAAGTACTGGAACAGTCAGAAGGAAATCCTGGAGGAACAACGAGCCATTGTGGACACGGTGTGCCGACACAACTACGAGATAGATAAGCCCTTCACGGTGGACAGAAGAGTCCAGCCCAGAGTGACCATCTCCCCCTCCAAGACAGAGGCCCTGCAGCACCTGCTGGTCTGCTCTGTCACTGGCTTCTATCCAAGCAAGATCAAGGTCACCTGGCTCAAGAATGGGCAGGAGGAGACAGCTGGGGTTGTGTCCACGGGTGTGATACAACATGGAGACTGGACCTACCAGATCCTAGTCATGTTGGAAATGATTCCCCAGAGCAGAGACATCTACACCTGCAGTGTGGAGCATGCCAGCCTGCAGAGCCCCATCAGTGTGGAATGGAGAGCACAGTCTGACTCTGCCCAGAGCAAATTGCTGAGTGGAATTGGAGGCTTTGTCCTGGGGCTGATATTGCTAGGTGTAGGACTGTTCATCCACCTCAAGAACCAGAAAGGTAAGGAGCATGTCGGGGAGGCCATGGGGCTCTGGCTTTGGTCCCAGCCCCTGCCCCTTACCCCATAG

>DBB_3_Hap_3

ATGGTTGATGTTTGGATCTCTGCTGGCTGCGGGAAGATTGGTCTGTTAATGACATCGATGGTGTTGAGTCTACCTGCTTCTTGGGCCAGGGACATCCCAGAGGATTTCGTGTATCAGTTCAAGGGAGAGTGTTACTTCACCAACAGCACGGAGCGGGTGCGGCTTGTGGCCAGACGCTTCTACAATGACCAGGAAATTGTCCGCTTCGACAGCGACGTGGGGGAGTTCGTGGCTCTGACGGAGCTGGGGCGGCCCGATGCTAAGTACTGGAACAGTCAGGAGGAAATCCTGGAGGAATATCGAGCTTACGTGGACACGGTGTGCAGACACAACTACGAGATAGAGAAGCCCTTCGCGGTGGACAGAAGAGTCCAGCCCAGAGTGACCATCTCCCCCTCCAAGACAGAGGCCCTGCAGCACCTGCTGGTGTGCTCTGTCACTGGCTTCTATCCAAGCAAGATCAAGGTCACCTGGCTCAAGAATGGGCAGGAGGAGACAGCTGGGGTTGTGTCCACGGGTGTGATACAACATGGAGACTGGACCTACCAGATCCTAGTCATGTTGGAAATGATTCCCCAGAGCAGAGACATCTACACCTGCAGTGTGGAGCATGCCAGCCTGCAGAGCCCCATCAGTGTGGAATGGAGAGCACAGTCTGACTCTGCCCAGAGCAAATTGCTGAGTGGAATTGGAGGCTTTGTCCTGGGGCTGATCTTCCTAGGTGTAGGACTTTTCATCCACCTCAAGAACCAGAAAGGTAAGGAGCATGTCGGGGAGGCCATGGGGCTCTGGCTTTGGCCCCAGCTCCTGCCCCGTATCCTGTAG

>DBB_3_Hap_1

ATGGTTGATGTTTGGATCTCTGCTGGCTGCTGGAAGATTGGTCTGTTAATGACATCGATGGTGTTGAGTCTACCTGCTTCTTGGGCCAGGGACATCCCAGAGGATTTCGTGTATCAGTTCAAGGGAGAGTGTTACTTCACCAACGGCACGGAGCGGGTGCGGCTTGTGGCCAGATACTTCTACAATGACCAGGAAATTGTCCGCTTCGACAGCGACGTGGGGGAGTTCGTGGCTCTGACGGAGCTGGGGCGGCCCGATGCTAAGTACTGGAACAGTCAGGAGGAAATCCTGGAGGAATATCGAGCTTACGTGGACACGCTGTGCAGACACAACTACGAGATAGAGAAGCCCTTCGCGGTGGACAGAAGAGTCCAGCCCAGAGTGACCATCTCCCCCTCCAAGACAGAGGCCCTGCAGCACCTGCTGGTGTGCTCTGTCACTGGCTTCTATCCAAGCAAGATCAAGGTCACCTGGCTCAAGAATGGGCAGGAGGAGACAGCTGGGGTTGTGTCCACGGGTGTGATACAACATGGAGACTGGACCTACCAGATCCTAGTCATGTTGGAAATGATTCCCCAGAGCAGAGACATCTACACCTGCAGTGTGGAGCATGCCAGCCTGCAGAGCCCCATCAGTGTGGAATGGAGAGCACAGTCTGACTCTGCCCAGAGCAAATTGCTGAGTGGAATTGGAGGCTTTGTCCTGGGGCTGATCTTCCTAGGTGTAGGACTTTTCATCCACCTCAAGAACCAGAAAGGTAAGGAGCATGTCGGGGAGGCCATGGGGCTCTGGCTTTGGCCCCAGCTCCTGCCCCGTATCCTGTAG

>DBB_3_Hap_4

ATGGTTGATGTTTGGATCTCTGCTGGCTGCTGGAAGATTGGTCTGTTAATGACATCGATGGTGTTGAGTCTACCTGCTTCTTGGGCCAGGGACATCCCAGAGGATTTCGTGTATCAGCTCAAGGGAGAGTGTTACTTCACCAACGGCACGGAGCGGGTGCGGCTTGTGGCCAGACACTTCTACAATGACCAGGAAATTGTCCGCTTCGACAGCGACGTGGGGGAGTTCGTGGCTCTGACGGAGCTGGGGCGGCCCGATGCTAAGTACTGGAACAGTCAGGAGGAAATCCTGGAGGAAAAACGAGCCTACGTGGACACACTGTGCAGACACAACTACGAGATAGAGAAGCCCTTCGCGGTGGACAGAAGAGTCCAGCCCAGAGTGACCATCTCCCCCTCCAAGACAGAGGCCCTGCAGCACCTGCTGGTGTGCTCTGTCACTGGCTTCTATCCAAGCAAGATCAAGGTCACCTGGCTCAAGAATGGGCAGGAGGAGACAGCTGGGGTTGTGTCCACGGGTGTGATACAACATGGAGACTGGACCTACCAGATCCTAGTCATGTTGGAAATGATTCCCCAGAGCAGAGACATCTACACCTGCAGTGTGGAGCATGCCAGCCTGCAGAGCCCCATCAGTGTGGAATGGAGAGCACAGTCTGACTCTGCCCAGAGCAAATTGCTGAGTGGAATTGGAGGCTTTGTCCTGGGGCTGATCTTCCTAGGTGTAGGACTTTTCATCCACCTCAAGAACCAGAAAGGTAAGGAGCATGTCGGGGAGGCCATGGGGCTCTGGCTTTGGCCCCAGCTCCTGCCCCGTATCCTGTAG

>DBB_3_Hap_6

ATGGTTGATGTTTGGATCTCTGCTGGCTGCTGGAAGATTGGTCTGTTAATGACATCGATGGTGTTGAGTCTACCTGCTTCTTGGGCCAGGGACATCCCAGAGGATTTCGTGTATCAGCTCAAGGGAGAGTGTTACTTCACCAACGGCACGGAGCGGGTGCGGCTTGTGGCCAGATGCTTCTACAATGACCAGGAAATTGTCCGCTTCGACAGCGACGTGGGGGAGTTCGTGGCTCTGACGGAGCTGGGGCGGCCCGATGCTAAGTACTGGAACAGTCAGGAGGAAATCCTGGAGGAATATCGAGCTTACGTGGACACGGTGTGCAGACACAACTACGAGATAGAGAAGCCCTTCGCGGTGGACAGAAGAGTCCAGCCCAGAGTGACCATCTCCCCCTCCAAGACAGAGGCCCTGCAGCACCTGCTGGTGTGCTCTGTCACTGGCTTCTATCCAAGCAAGATCAAGGTCACCTGGCTCAAGAATGGGCAGGAGGAGACAGCTGGGGTTGTGTCCACGGGTGTGATACAACATGGAGACTGGACCTACCAGATCCTAGTCATGTTGGAAATGATTCCCCAGAGCAGAGACATCTACACCTGCAGTGTGGAGCATGCCAGCCTGCAGAGCCCCATCAGTGTGGAATGGAGAGCACAGTCTGACTCTGCCCAGAGCAAATTGCTGAGTGGAATTGGAGGCTTTGTCCTGGGGCTGATCTTCCTAGGTGTAGGACTTTTCATCCACCTCAAGAACCAGAAAGGTAAGGAGCATGTCGGGGAGGCCATGGGGCTCTGGCTTTGGCCCCAGCTCCTGCCCCGTATCCTGTAG

>DBB_3_Hap_7

ATGGTTGATGTTTGGATCTCTGCTGGCTGCTGGAAGATTGGTCTGTTAATGACATCGATGGTGTTGAGTCTACCTGCTTCTTGGGCCAGGGACATCCCAGAGGATTTCGTGTATCAGCTCAAGGGAGAGTGTTACTTCACCAACGGCACGGAGCGGGTGCGGCTTGTGGCCAGATGCTTCTACAATGACCAGGAAATTGTCCGCTTCGACAGCGACGTGGGGGAGTTCGTGGCTCTGACGGAGCTGGGGCGGCCCGATGCTAAGTACTGGAACAGTCAGGAGGAAATCCTGGAGGAAAAACGAGCCTACGTGGACACACTGTGCAGACACAACTACGAGATAGAGAAGCCCTTCGCGGTGGACAGAAGAGTCCAGCCCAGAGTGACCATCTCCCCCTCCAAGACAGAGGCCCTGCAGCACCTGCTGGTGTGCTCTGTCACTGGCTTCTATCCAAGCAAGATCAAGGTCACCTGGCTCAAGAATGGGCAGGAGGAGACAGCTGGGGTTGTGTCCACGGGTGTGATACAACATGGAGACTGGACCTACCAGATCCTAGTCATGTTGGAAATGATTCCCCAGAGCAGAGACATCTACACCTGCAGTGTGGAGCATGCCAGCCTGCAGAGCCCCATCAGTGTGGAATGGAGAGCACAGTCTGACTCTGCCCAGAGCAAATTGCTGAGTGGAATTGGAGGCTTTGTCCTGGGGCTGATCTTCCTAGGTGTAGGACTTTTCATCCACCTCAAGAACCAGAAAGGTAAGGAGCATGTCGGGGAGGCCATGGGGCTCTGGCTTTGGCCCCAGCTCCTGCCCCGTATCCTGTAG

>DBB_3_Hap_8

ATGGTTGATGTTTGGATCTCTGCTGGCTGCTGGAAGATTGGTCTGTTAATGACATCGATGGTGTTGAGTCTACCTGCTTCTTGGGCCAGGGACATCCCAGAGGATTTCGTGTATCAGTTCAAGGGAGAGTGTTACTTCACCAACGGCACGGAGCGGGTGCGGCTTGTGGCCAGATACTTCTACAATGACCAGGAAATTGTCCGCTTCGACAGCGACGTGGGGGAGTTCGTGGCTCTGACGGAGCTGGGGCGGCCCGATGCTAAGTACTGGAACAGTCAGGAGGAAATCCTGGAGGAATATCGAGCTTACGTGGACACGCTGTGCAGACACAACTACGAGATAGAGAAGCCCTTCGCGGTGGACAGAAGAGTCCAGCCCAGAGTGACCATCTCCCCCTCCAAGACAGAGGCCCTGCAGCACCTGCTGGTGTGCTCTGTCACTGGCTTCTATCCAAGCAAGATCAAGGTCACCTGGCTCAAGAATGGGCAGGAGGAGACAGCTGGGGTTGTGTCCACGGGTGTGATACAACATGGAGACTGGACCTACCAGATCCTAGTCATGTTGGAAATGATTCCCCAGAGCAGAGACATCTACACCTGCAGTGTGGAGCATGCCAGCCTGCAGAGCCCCATCAGTGTGGAATGGAGAGCACAGTCTGACTCTGCCCAGAGCAAATTGCTGAGTGGAATTGGAGGCTTTGTCCTGGGGCTGATCTTCCTAGGTGTAGGACTTTTCATCCACCTCAAGAACCAGAAAGGTAAGGAGCATGTCGGGGAGGCCATGGGGCTCTGGCTTTGGCCCCAGCTCCTGCCCCTTATCCTGTAG

>DBB_3_Hap_11

ATGGTTGATGTTTGGATCTCTGCTGGCTGCTGGAAGATTGGTCTGTTAATGACATCGATGGTGTTGAGTCTACCTGCTTCTTGGGCCAGGGACATCCCAGAGGATTTCGTGTATCAGCTCAAGGGAGAGTGTTACTTCACCAACGGCACGGAGCGGGTGCGGCTTGTGGCCAGACACTTCTACAATGACCAGGAAATTGTCCGCTTCGACAGCGACGTGGGGGAGTTCGTGGCTCTGACGGAGCTGGGGCGGCCCGATGCTAAGTACTGGAACAGTCAGGAGGAAATCCTGGAGGAAAAACGAGCCTACGTGGACACACTGTGCAGACACAACTACGAGATAGAGAAGCCCTTCGCGGTGGACAGAAGAGTCCAGCCCAGAGTGACCATCTCCCCCTCCAAGACAGAGGCCCTGCAGCACCTGCTGGTGTGCTCTGTCACTGGCTTCTATCCAAGCAAGATCAAGGTCACCTGGCTCAAGAATGGGCAGGAGGAGACAGCTGGGGTTGTGTCCACGGGTGTGATACAACATGGAGACTGGACCTACCAGATCCTAGTCATGTTGGAAATGATTCCCCAGAGCAGAGACATCTACACCTGCAGTGTGGAGCATGCCAGCCTGCAGAGCCCCATCAGTGTGGAATGGAGAGCACAGTCTGACTCTGCCCAGAGCAAATTGCTGAGTGGAATTGGAGGCTTTGTCCTGGGGCTGATCTTCCTAGGTGTAGGACTTTTCATCCACCTCAAGAACCAGAAAGGTAAGGAGCATGTCGGGGAGGCCATGGGGCTCTGGCTTTGGCCCCAGCTCCTGCCCCTTATCCTGTAG

>DBB_3_Hap_12

ATGGTTGATGTTTGGATCTCTGCTGGCTGCTGGAAGATTGGTCTGTTAATGACATCGATGGTGTTGAGTCTACCTGCTTCTTGGGCCAGGGACATCCCAGAGGATTTCGTGTATCAGCTCAAGGGAGAGTGTTACTTCACCAACGGCACGGAGCGGGTGCGGCTTGTGGCCAGATGCTTCTACAATGACCAGGAAATTGTCCGCTTCGACAGCGACGTGGGGGAGTTCGTGGCTCTGACGGAGCTGGGGCGGCCCGATGCTAAGTACTGGAACAGTCAGGAGGAAATCCTGGAGGAATATCGAGCTTACGTGGACACGGTGTGCAGACACAACTACGAGATAGAGAAGCCCTTCGCGGTGGACAGAAGAGTCCAGCCCAGAGTGACCATCTCCCCCTCCAAGACAGAGGCCCTGCAGCACCTGCTGGTGTGCTCTGTCACTGGCTTCTATCCAAGCAAGATCAAGGTCACCTGGCTCAAGAATGGGCAGGAGGAGACAGCTGGGGTTGTGTCCACGGGTGTGATACAACATGGAGACTGGACCTACCAGATCCTAGTCATGTTGGAAATGATTCCCCAGAGCAGAGACATCTACACCTGCAGTGTGGAGCATGCTAGCCTGCAGAGCCCCATCAGTGTGGAATGGAGAGCACAGTCTGACTCTGCCCAGAGCAAATTGCTGAGTGGAATTGGAGGCTTTGTCCTGGGGCTGATCTTCCTAGGTGTAGGACTTTTCATCCACCTCAAGAACCAGAAAGGTAAGGAGCATGTCGGGGAGGCCATGGGGCTCTGGCTTTGGCCCCAGCTCCTGCCCCGTATCCTGTAG

>DBB_3_Hap_2

ATGGTTGATGTTTGGATCTCTGCTGGCTGCGGGAAGATTGGTCTGTTAATGACATCGATGGTGTTGAGTCTACCTGCTTCTTGGGCCAGGGACATCCCAGAGGATTTCGTGTATCAGTTCAAGGGAGAGTGTTACTTCACCAACAGCACGGAGCGGGTGCGGCTTGTGGCCAGACGCTTCTACAATGACCAGGAAATTGTCCGCTTCGACAGCGACGTGGGGGAGTTCGTGGCTCTGACGGAGCTGGGGCGGCCCGATGCTAAGTACTGGAACAGTCAGGAGGAAATCCTGGAGGAATATCGAGCTTACGTGGACACGGTGTGCAGACACAACTACGAGATAGAGAAGCCCTTCGCGGTGGACAGAAGAGTCCAGCCCAGAGTGACCATCTCCCCCTCCAAGACAGAGGCCCTGCAGCACCTGCTGGTGTGCTCTGTCACTGGCTTCTATCCAAGCAAGATCAAGGTCACCTGGCTCAAGAATGGGCAGGAGGAGACAGCTGGGGTTGTGTCCACGGGTGTGATACAACATGGAGACTGGACCTACCAGATCCTAGTCATGTTGGAAATGATTCCCCAGAGCAGAGACATCTACACCTGCAGTGTGGAGCATGCTAGCCTGCAGAGCCCCATCAGTGTGGAATGGAGAGCACAGTCTGACTCTGCCCAGAGCAAATTGCTGAGTGGAATTGGAGGCTTTGTCCTGGGGCTGATCTTCCTAGGTGTAGGACTTTTCATCCACCTCAAGAACCAGAAAGGTAAGGAGCATGTCGGGGAGGCCATGGGGCTCTGGCTTTGGCCCCAGCTCCTGCCCCTTATCCTGTAG

>DBB_3_Hap_5

ATGGTTGATGTTTGGATCTCTGCTGGCTGCGGGAAGATTGGTCTGTTAATGACATCGATGGTGTTGAGTCTACCTGCTTCTTGGGCCAGGGACATCCCAGAGGATTTCGTGTATCAGCTCAAGGGAGAGTGTTACTTCACCAACGGCACGGAGCGGGTGCGGCTTGTGGCCAGATGCTTCTACAATGACCAGGAAATTGTCCGCTTCGACAGCGACGTGGGGGAGTTCGTGGCTCTGACGGAGCTGGGGCGGCCCGATGCTAAGTACTGGAACAGTCAGGAGGAAATCCTGGAGGAATATCGAGCTTACGTGGACACGGTGTGCAGACACAACTACGAGATAGAGAAGCCCTTCGCGGTGGACAGAAGAGTCCAGCCCAGAGTGACCATCTCCCCCTCCAAGACAGAGGCCCTGCAGCACCTGCTGGTGTGCTCTGTCACTGGCTTCTATCCAAGCAAGATCAAGGTCACCTGGCTCAAGAATGGGCAGGAGGAGACAGCTGGGGTTGTGTCCACGGGTGTGATACAACATGGAGACTGGACCTACCAGATCCTAGTCATGTTGGAAATGATTCCCCAGAGCAGAGACATCTACACCTGCAGTGTGGAGCATGCCAGCCTGCAGAGCCCCATCAGTGTGGAATGGAGAGCACAGTCTGACTCTGCCCAGAGCAAATTGCTGAGTGGAATTGGAGGCTTTGTCCTGGGGCTGATCTTCCTAGGTGTAGGACTTTTCATCCACCTCAAGAACCAGAAAGGTAAGGAGCATGTCGGGGAGGCCATGGGGCTCTGGCTTTGGCCCCAGCTCCTGCCCCGTATCCTGTAG

>DBB_3_Hap_10

ATGGTTGATGTTTGGATCTCTGCTGGCTGCGGGAAGATTGGTCTGTTAATGACATCGATGGTGTTGAGTCTACCTGCTTCTTGGGCCAGGGACATCCCAGAGGATTTCGTGTATCAGTTCAAGGGAGAGTGTTACTTCACCAACGGCACGGAGCGGGTGCGGCTTGTGGCCAGACGCTTCTACAATGACCAGGAAATTGTCCGCTTCGACAGCGACGTGGGGGAGTTCGTGGCTCTGACGGAGCTGGGGCGGCCCGATGCTAAGTACTGGAACAGTCAGGAGGAAATCCTGGAGGAAAAACGAGCCTACGTGGACACACTGTGCAGACACAACTACGAGATAGAGAAGCCCTTCGCGGTGGACAGAAGAGTCCAGCCCAGAGTGACCATCTCCCCCTCCAAGACAGAGGCCCTGCAGCACCTGCTGGTGTGCTCTGTCACTGGCTTCTATCCAAGCAAGATCAAGGTCACCTGGCTCAAGAATGGGCAGGAGGAGACAGCTGGGGTTGTGTCCACGGGTGTGATACAACATGGAGACTGGACCTACCAGATCCTAGTCATGTTGGAAATGATTCCCCAGAGCAGAGACATCTACACCTGCAGTGTGGAGCATGCCAGCCTGCAGAGCCCCATCAGTGTGGAATGGAGAGCACAGTCTGACTCTGCCCAGAGCAAATTGCTGAGTGGAATTGGAGGCTTTGTCCTGGGGCTGATCTTCCTAGGTGTAGGACTTTTCATCCACCTCAAGAACCAGAAAGGTAAGGAGCATGTCGGGGAGGCCATGGGGCTCTGGCTTTGGCCCCAGCTCCTGCCCCGTATCCTGTAG

>DBB_3_Hap_9

ATGGTTGATGTTTGGATCTCTGCTGGCTGCGGGAAGATTGGTCTGTTAATGACATCGATGGTGTTGAGTCTACCTGCTTCTTGGGCCAGGGACATCCCAGAGGATTTCGTGTATCAGCTCAAGGGAGAGTGTTACTTCACCAACGGCACGGAGCGGGTGCGGCTTGTGGCCAGACGCTTCTACAATGACCAGGAAATTGTCCGCTTCGACAGCGACGTGGGGGAGTTCGTGGCTCTGACGGAGCTGGGGCGGCCCGATGCTAAGTACTGGAACAGTCAGGAGGAAATCCTGGAGGAAAAACGAGCCTACGTGGACACACTGTGCAGACACAACTACGAGATAGAGAAGCCCTTCGCGGTGGACAGAAGAGTCCAGCCCAGAGTGACCATCTCCCCCTCCAAGACAGAGGCCCTGCAGCACCTGCTGGTGTGCTCTGTCACTGGCTTCTATCCAAGCAAGATCAAGGTCACCTGGCTCAAGAATGGGCAGGAGGAGACAGCTGGGGTTGTGTCCACGGGTGTGATACAACATGGAGACTGGACCTACCAGATCCTAGTCATGTTGGAAATGATTCCCCAGAGCAGAGACATCTACACCTGCAGTGTGGAGCATGCCAGCCTGCAGAGCCCCATCAGTGTGGAATGGAGAGCACAGTCTGACTCTGCCCAGAGCAAATTGCTGAGTGGAATTGGAGGCTTTGTCCTGGGGCTGATCTTCCTAGGTGTAGGACTTTTCATCCACCTCAAGAACCAGAAAGGTAAGGAGCATGTCGGGGAGGCCATGGGGCTCTGGCTTTGGCCCCAGCTCCTGCCCCGTATCCTGTAG

>DCA_1_Hap_3

ATGACTTCCAGTCTAGACTCAGTCTTGGGGATCCTCATCCTGGCTGCCCTGCTGATCAAACAAAGAACCTGGGCCACTAATGACCATGTAATCAGCTCTGTGACATTTGTTCAGACCCACAAACCATCTGGGCAGTACCTGCATGAATTTGATGAGCATGAGCTATTCCATGTGGACTTTGACCAGAAGGAAACAGTCTGGAGGCTTCCTGAGTTTGGGCACATCTTCAGTTTCGATGCACAGATTGGGCTAGGTGACATTGCTGTGGACATGGCTAACTTGAACCAACTTATCAAGCAAACCAACCACACCCAGGCCACCATTGTGACTCCAGAGGTGACAGTGTTTCCCAAGGAGCCCGTGGAACTGGAAGAACCCAACATCCTCATCTGCCACGTTGATAAGTTTTCACCCCCAGTGGTTAATGTCACATGGTTGTGCAATGGAGAACCAGTCACCACAGGGGTATCTGAGACTTCCTTAATGCCTCGGGATGACTATTCTTTCCATAAGTTCCATTATCTCACTTTCCTCCCCTCAGCCGATGATGTCTATGACTGTGTGGTTGAACACTGGAGCCTGGAAAAGCCACTTTTCAAGCATTGGGAGCCTGAGATGTTAACACCACCATCTGAGACAATGGAGACACTCCTCTGCATTCTAGGCCTGGCCGTGGGCCTGGTGGGCATCACTGTGGCTGCCTCCCTTATTATCAAAAGCTTGCACTTAGGCGAATGA

>DCA_1_Hap_1

ATGACTTCCAGTCTAGACTCAGTCTTGGGGATCCTCATCCTGGCTGCCCTGCTGATCAAACAAAGAACCTGGGCCACTAATGACCATGTAATCAGCTCTGTGACATTTGTTCAGACCCACAAACCATCTGGGCAGTACCTGCATGAATTTGATGAGCATGAGCTATTCCATGTGGACTTTGACCAGAAGGAAACAGTCTGGAGGCTTCCTGAGTTTGGGCACATCTTCAGTTTCGATGCACAGATTGGGCTAGGTGACATTGCTGTGGACATGGCTAACTTGAACCAACTTATCAAGCAAACCAACCACACCCAGGCCACCATTGTGACTCCAGAGGTGACAGTGTTTCCCAAGGAGCCCGTGGAACTGGAAGAACCCAACATCCTCATCTGCCACGTTGATAAGTTTTCACCCCCAGTGGTTAATGTCACATGGTTGTGCAATGGAGAACCAGTCACCACAGGGGTATCTGAGACTGCCTTAATGCCTCGGGATGACTATTCTTTCCATAAGTTCCATTATCTCACTTTCCTCCCCTCAGCCGATGATGTCTATGACTGTGTGGTTGAACACTGGAGCCTGGAAAAGCCACTTTTCAAGCATTGGGAGCCTGAGATGTTAACACCACCATCTGAGACAATGGAGACACTCCTCTGCATTCTAGGCCTGGCCGTGGGCCTGGTGGGCATCACTGTGGCTGCCTCCCTTATTATCAAAAGCTTGCACTTAGGCGAATGA

>DCA_1_Hap_4

ATGACTTCCAGTCTAGACTCAGTCTTGGGGATCCTCATCCTGGCTGCCCTGCTGATCAAACAAAGAACCTGGGCCACTAATGACCATGTAATCAGCTCTGTGACATTTGTTCAGACCCACAAACCATCTGGGCAGTACCTGCATGAATTTGATGAGCATGAGCTATTCCATGTGGACTTTGACCAGAAGGAAACAGTCTGGAGGCTTCCTGAGTTTGGGCACATCTTCAGTTTCGATGCACAGATTGGGCTAGGTGACATTGCTGTGGACATGGCTAACTTGAACCAACTTATCAAGCAAACCAACCACACCCAGGCCACCATTGTGACTCCAGAGGTGACAGTGTTTCCCAAGGAGCCCGTGGAACTGGAAGAACCCAACATCCTCATCTGCCACGTTGATAAGTTTTCACCCCCAGTGGTTAATGTCACATGGTTGTGCAATGGAGAACCAGTCACCACAGGGGTATCTGAGACTGCCTTAATGCCTCGGGATGACTATTCTTTCCATAAGTTCCATTATCTCACTTTCCTCCCCTCAGCCAATGATGTCTATGACTGTGTGGTTGAACACTGGAGCCTGGAAAAGCCACTTTTCAAGCATTGGGAGCCTGAGATATTAACACCACCATCTGAGACAATGGAGACACTCCTCTGTATTCTAGGCCTGGCCGTGGGCCTGGTGGGCATCACTGTGGCTGCCTCCCTTATTATCAAAAGCTTGCACTTAGGCGAATGA

>DCA_1_Hap_5

ATGACTTCCAGTCTAGACTCAGTCTTGGGGATCCTCATCCTGGCTGCCCTGCTGATCAAACAAAGAACCTGGGCCACTAATGACCATGTAATCAGCTCTGTGACATTTGTTCAGACCCACAAACCATCTGGGCAGTACCTGCATGAATTTGATGAGCATGAGCTATTCCATGTGGACTTTGACCAGAAGGAAACAGTCTGGAGGCTTCCTGAGTTTGGGCACATCTTCAGTTTCGATGCACAGATTGGGCTAGGTGACATTGCTGTGGACATGGCTAACTTGAACCAACTTATCAAGCAAACCAACCACACCCAGGCCACCATTGTGACTCCAGAGGTGACAGTGTTTCCCAAGGAGCCCGTGGAACTGGAAGAACCCAACATCCTCATCTGCCACGTTGATAAGTTTTCACCCCCAGTGGTTAATGTCACATGGTTGTGCAATGGAGAACCAGTCACCACAGGGGTATCTGAGACTGCCTTAATGCCTCGGGATGACTATTCTTTCCATAAGTTCCATTATCTCACTTTCCTCCCCTCAGCCGATGATGTCTATGACTGTGTGGTTGAACACTGGAGCCTGGAAAAGCCACTTTTCAAGCATTGGGAGCCTGAGATGTTAACACCACCATCTGAGACAATGGAGACACTCCTCTGTATTCTAGGCCTGGCCGTGGGCCTGGTGGGCATCACTGTGGCTGCCTCCCTTATTATCAAAAGCTTGCACTTAGGCGAATGA

>DCA_1_Hap_6

ATGACTTCCAGTCTAGACTCAGTCTTGGGGATCCTCATCCTGGCTGCCCTGCTGATCAAACAAAGAACCTGGGCCACTAATGACCATGTAATCAGCTCTGTGACATTTGTTCAGACCCACAAACCATCTGGGCAGTACCTGCATGAATTTGATGAGCATGAGCTATTCCATGTGGACTTTGACCAGAAGGAAACAGTCTGGAGGCTTCCTGAGTTTGGGCACATCTTCAGTTTCGATGCACAGATTGGGCTAGGTGACATTGCTGTGGACATGGCTAACTTGAACCAACTTATCAAGCAAACCAACCACACCCAGGCCACCATTGTGACTCCAGAGGTGACAGTGTTTCCCAAGGAGCCCGTGGAACTGGAAGAACCCAACATCCTCATCTGCCACGTTGATAAGTTTTCACCCCCAGTGGTTAATGTCACATGGTTGTGCAATGGAGAACCAGTCACCACAGGGGTATCTGAGACTGCCTTAATGCCTCGGGATGACTATTCTTTCCATAAGTTCCATTATCTCACTTTCCTCCCCTCAGCCAATGATGTCTATGACTGTGTGGTTGAACACTGGAGCCTGGAAAAGCCACTTTTCAAGCATTGGGAGCCTGAGATGTTAACACCACCATCTGAGACAATGGAGACACTCCTCTGCATTCTAGGCCTGGCCGTGGGCCTGGTGGGCATCACTGTGGCTGCCTCCCTTATTATCAAAAGCTTGCACTTAGGCGAATGA

>DCA_1_Hap_2

ATGACTTCCAGTCTAGACTCAGTCTTGGGGATCCTCATCCTGGCTGCCCTGCTGATCAAACAAAGAACCTGGGCCACTAATGACCATGTAATCAGCTCTGTGACATTTGTTCAGACCCACAAACCATCTGGGCAGTACCTGCATGAATTTGATGAGCATGAGCTATTCCATGTGGACTTTGACCAGAAGGAAACAGTCTGGAGGCTTCCTGAGTTTGGGCACATCTTCAGTTTCGATGCACAGATTGGGCTAGGTGACATTGCTGTGGACATGGCTAACTTGAACCAACTTATCAAGCAAACCAACCACACCCAGGCCACCATTGTGACTCCAGAGGTGACGGTGTTTCCCAAGGAGCCCGTGGAACTGGAAGAACCCAACATCCTCATCTGCCACGTTGATAAGTTTTCACCCCCAGTGGTTAATGTCACATGGTTGTGCAATGGAGAACCAGTCACCACAGGGGTATCTGAGACTGCCTTAATGCCTCGGGATGACTATTCTTTCCATAAGTTCCATTATCTCACTTTCCTCCCCTCAGCCGATGATGTCTATGACTGTGTAGTTGAACACTGGAGCCTGGAAAAGCCACTTTTCAAGCATTGGGAGCCTGAGATGTTAACACCACCATCTGAGACAATGGAGACACTCCTCTGTATTCTAGGCCTGGCCGTGGGCTTGGTGGGCATCACTGTGGCTGCCTCCCTTATTATCAAAAGCTTGCACTTAGGCAAATGA

>DCB_1_Hap_3

ATGTGGAGGGCAGACCTTATTGGAAGCATGCTGCAACTTTTAATCACCAGTTCCATCCTTGTCTCCAATATGGTATGTGTGGAGCTTCTGGGAAGCCTCTGTATGACAGTCTTATTAATGGTGCTGAGCAGGCCCACAGCCTGGGGCAGGGACATTCCAGAGAATTACCTACATCAGGTGAGGTCTGAGTGTCACATGACCAATGGAACCCAACGGGTCCGCTTTGTGGGAAGACTCATCTATGACCGGAAGGAGTTCGTGCACTTTGACAGTGACGTGGGACTATTTGAGGCAAAAATGGAGTTGTGGAGATCCCAAGTCCAGAAATGGAACAGTCAGAAGGAAATAGTCAAGACTGCAAGGTCCATAGTGAATGTGTGCAGACACAATTACCTTTTATATGATAAACTCATAGTGCAAAGGAAAGTTCAGCCCCGAGTGAAGGTTTTCCCCTCAAAGATACAACCACTTGGGCACCACAACCTGCTCCTCTGTTCCGTGACCAGTTTCTATCCTGGTGAGATCAAGGTCAGCTGGTTCAGGAATGCAAAAGAAGAAAAGTCTGGAGTCCTGTCCACAGGCCAAATCCAGAATGGTGACTGGACCTTCCAGACCCTTGTGATGCTAGAAGTGACCCCCCAAAGAGGAGATGTCTTTACTTGCCATGTGGACCATGTCAGCTTGCAGAGCCCTATCACCGTAGACTGGAGAGCACAGTCTGAATCTGCCCGGACTAAGATGCTGACTGGAATTGGGGGCTTGGTGCTTGGACTGATCTTACTTGGAGTGGGACTTGTCATCCACCTTAGAAGTTTGAAAGATTCCTATTCTGCGACCAAAGAAGACTCAAATTTGGAAGGGATTGTGAACATTGCCCCATTGCAACAAGATTTTCCCAGAGCTGTTGCCCAGTCCTAG

>DCB_1_Hap_1

ATGTGGAGGGCAGACCTTATTGGAAGCATGCTGCAACTTTTAATCACCAGTTCCATCCTTGTCTCCAATATGGTATGTGTGGAGCTTCTGGGAAGCCTCTGTATGACAGTCTTATTAATGGTGCTGAGCAGGCCCACAGCCTGGGGCAGGGACATTCCAGAGAATTACCTACATCAGGTGAGGTCTGAGTGTCACATGACCAATGGAACCCAACGGGTCCGCTTTGTGGGAAGACTCATCTATGACCGGAAGGAGTTTGTGCACTTTGACAGTGACGTGGGACTATTTGAGGCAAAAATGGAGTTGTGGAGATCCCAAGTCCAGAAATGGAACAGTCAGAAGGAAATAGTCAAGACTGCAAGGTCCATAGTGAATGTGTGCAGACACAATTACCTTTTATATGATAAACTCATAGTGCAAAGGAAAGTTCAGCCCCGAGTGAAGGTTTTCCCCTCAAAGATACAACCACTTGGGCACCACAACCTGCTCCTCTGTTCCGTGACCAGTTTCTATCCTGGTGAGATCAAGGTCAGCTGGTTCAGGAATGCAAAAGAAGAAAAGTCTGGAGTCCTGTCCACAGGCCAAATCCAGAATGGTGACTGGACCTTCCAGACCCTTGTGATGCTAGAAGTGACCCCCCAAAGAGGAGATGTCTTTACTTGCCATGTGGACCATGTCAGCTTGCAGAGCCCTACCACCGTAGACTGGAGAGCACAGTCTGAATCTGCCCGGACTAAGATGCTGACTGGAATTGGGGGCTTGGTGCTTGGACTGATCTTACTTGGAGTGGGACTTGTCATCCACCTTAGAAGTTTGAAAGATTCCTATTCTGCGACCAAAGAAGACTCAAATTTGGAAGGGATTGTGAACATTGCCCCATTGCAACAAGATTTTCCCAGAGATGTTGCCCAGTCCTAG

>DCB_1_Hap_4

ATGTGGAGGGCAGACCTTATTGGAAGCATGCTGCAACTTTTAATCACCAGTTCCATCCTTGTCTCCAATATGGTATGTGTGGAGCTTCTGGGAAGCCTCTGTATGACAGTCTTATTAATGGTGCTGAGCAGGCCCACAGCCTGGGGCAGGGACATTCCAGAGAATTACCTACATCAGGTGAGGTCTGAGTGTCACATGACCAATGGAACCCAACGGGTCCGCTTTGTGGGAAGACTCATCTATGACCGGAAGGAGTTTGTGCACTTTGACAGTGACGTGGGACTATTTGAGGCAAAAATGGAGTTGTGGAGATCCCAAGTCCAGAAATGGAACAGTCAGAAGGAAATAGTCAAGACTGCAAGGTCCATAGTGAATGTGTGCAGACACAATTACCTTTTATATGATAAACTCATAGTGCAAAGGAAAGTTCAGCCCCGAGTGAAGGTTTTCCCCTCAAAGATACAACCACTTGGGCACCACAACCTGCTCCTCTGTTCCGTGACCAGTTTCTATCCTGGTGAGATCAAGGTCAGCTGGTTCAGGAATGCAAAAGAAGAAAAGTCTGGAGTCCTGTCCACAGGCCAAATCCAGAATGGTGACTGGACCTTCCAGACCCTTGTGATGCTAGAAGTGACCCCCCAAAGAGGAGATGTCTTTACTTGCCATGTGGACCATGTCAGCTTGCAGAGCCCTATCACCGTAGACTGGAGAGCACAGTCTGAATCTGCCCGGACTAAGATGCTGACTGGAATTGGGGGCTTGGTGCTTGGACTGATCTTACTTGGAGTGGGACTTGTCATCCACCTTAGAAGTTTGAAAGATTCCTATTCTGCGACCAAAGAAGACTCAAATTTGGAAGGGATTGTGAACATTGCCCCATTGCAACAAGATTTTCCCAGAGATGTTGCCCAGTCCTAG

>DCB_1_Hap_5

ATGTGGAGGGCAGACCTTATTGGAAGCATGCTGCAACTTTTAATCACCAGTTCCATCCTTGTCTCCAATATGGTATGTGTGGAGCTTCTGGGAAGCCTCTGTATGACAGTCTTATTAATGGTGCTGAGCAGGCCCACAGCCTGGGGCAGGGACATTCCAGAGAATTACCTACATCAGGTGAGGTCTGAGTGTCACATGACCAATGGAACCCAACGGGTCCGCTTTGTGGGAAGACTCATCTATGACCGGAAGGAGTTTGTGCACTTTGACAGTGACGTGGGACTATTTGAGGCAAAAATGGAGTTGTGGAGATCCCAAGTCCAGAAATGGAACAGTCAGAAGGAAATAGTCAAGACTGCAAGGTCCATAGTGAATGTGTGCAGACACAATTACCTTTTATATGATAAACTCATAGTGCAAAGGAAAGTTCAGCCCCGAGTGAAGGTTTTCCCCTCAAAGATACAACCACTTGGGCACCACAACCTGCTCCTCTGTTCCGTGACCAGTTTCTATCCTGGTGAGATCAAGGTCAGCTGGTTCAGGAATGCAAAAGAAGAAAAGTCTGGAGTCCTGTCCACAGGCCAAATCCAGAATGGTGACTGGACCTTCCAGACCCTTGTGATGCTAGAAGTGACCCCCCAAAGAGGAGATGTCTTTACTTGCCATGTGGACCATGTCAGCTTGCAGAGCCCTACCACCGTAGACTGGAGAGCACAGTCTGAATCTGCCCGGACTAAGATGCTGACTGGAATTGGGGGCTTGGTGCTTGGACTGATCTTACTTGGAGTGGGACTTGTCATCCACCTTAGAAGTTTGAAAGATTCCTATTCTGCGACCAAAGAAGACTCAAATTTGGAAGGGATTGTGAACATTGCCCCATTGCAACAAGATTTTCCCAGAGCTGTTGCCCAGTCCTAG

>DCB_1_Hap_2

ATGTGGAGGGCAGACCTTATTGGAAGCATGCTGCAACTTTTAATCACCAGTTCCATCCTTGTCTCCAATATGGTATGTGTGGAGCTTCTGGGAAGCCTCTGTATGACAGTCTTATTAATGGTGCTGAGCAGGCCCACAGCCTGGGGCAGGGACATTCCAGAGAATTACCTACATCAGGTGAGGTCTGAGTGTCACATGACCAATGGAACCCAACGGGTCCGCTTTGTGGGAAGACTCATCTATGACCGGAAGGAGTTTGTGCACTTTGACAGTGACGTGGGACTATTTGAGGCAAAAATGGAGTTGTGGAGATCCCAAGTCCAGAAATGGAACAGTCAGAAGGAAATAGTCAGGACTGCAAGGTCCATAGTGAATGTGTGCAGACACAATTACCTTTTATATGATAAACTCATAGTGCAAAGGAAAGTTCAGCCCCGAGTGAAGGTTTTCCCCTCAAAGATACAACCACTTGGGCACCACAACCTGCTCCTCTGTTCCGTGACCAGTTTCTATCCTGGTGAGATCAAGATCAGCTGGTTCAGGAATGCAAAAGAAGAAAAGTCTGGAGTCCTGTCCACAGGCCAAATCCAGAATGGTGACTGGACCTTCCAGACCCTTGTGATGCTAGAAGTGACCCCCCAAAGAGGAGATGTCTTTACTTGCCATGTGGACCATGTCAGCTTGCAGAGCCCTATCACCGTAGACTGGAGAGCACAGTCTGAATCTGCCCGGACTAAGATGCTGACTGGAATTGGGGGCTTGGTGCTTGGACTGATCTTACTTGGAGTGGGACTTGTCATCCACCTTAGAAGTTTGAAAGATTCCTATTCTGCGACCAAAGAAGACTCAAATTTGGAAGGGATTGTGAACATTGCCCCATTGCAACAAGATTTTCCCAGAGCTGTTGCCCAGTCCTAG

>DMA_1_Hap_2

ATGGGACCTGAGCAAAGCCTGGGAGCTATGCTGCTACTGCTGCAGCTACAGTCATCCCTGCTTTTATCCCTGTCCTGGGGAGCCACTCCAGTGTTGGCATCATTATTGGAGAATAGCCTACAGAACTACACGTTCTCTCACACACTGTTCTGCCAGGATGAGGATCCTGTCCTGAGTCTGTCAGAGGCCTTCAATGAAGACCAGCTCTTCTCCTTTGACTTCTCCAGGAACTCCCGGGTGCCCCGGCTGCCTGAATTTGCTCCTTGGGCCAGTGACAAGGGAGATATCGAGGCCATAAAAGCTGACCAGCGGCTCTGCCAGGAACTGCAAAAAGAATTGAGTAAACTATTGGAAGGCCACATCCCTGAGGCTAGAGGAAACCCTGTGGCTGAAGTTTTCACTCTGGAGCCCCTGGAGTATGGGAAGCCCAACACTCTCATCTGCTTTGTTAGTAATATCTTCCCACCTCAAGTAACTGTGAGCTGGCAGTACCAACAAGTCCCTGTGCAAAGCAGCAGCCCTACTTTTCTCTCAGCTATAGATGGACTTGCCTTCCAGGCCTTCTCTTATCTGAACTTCACACCCACATCCTCTGATGTCTTCTCTTGCACTGTGACCTGGGAAGGTGACCTCTTCAGCACTATAGCCTTCTGGGTGCCTCAGAATCCTATACCCTCTGAATTGTTGGAAAATATATTGTGTGGCATTGCCCTTGGCCTGGGAATTGTTGGCATCATAGTGGGTACTGCGCTCATCATCTACTTCCAAAAACCATGTGCAAGTGGTGCAGACTGA

>DMA_1_Hap_1

ATGGGACCTGAGCAAAGCCTGGGAGCTATGCTGCTACTGCTGCAGCTACAGTCATCCCTGCTTTTATCCCTGTCCTGGGGAGCCACTCCAGTGTTGGCATCATTATTGGAGAATAGCCTACAGAACTACACGTTCTCTCACACACTGTTCTGCCAGGATGAGGATCCTGTCCTGAGTCTGTCAGAGGCCTTCAATGAAGACCAGCTCTTCTCCTTTGACTTCTCCAGGAACTCCCGGGTGCCCCGGCTGCCTGAATTTGCTCCTTGGGCCAGTGACAAGGGAGATATCGAGGCCATAAAAGCTGACCAGCGGCTCTGCCAGGAACTGCAAAAAGAATTGAGTAAAATATTGGAAGGCCACATCCCTGAGGCTAGAGGAAACCCTGTGGCTGAAGTTTTCACTCTGGAGCCCCTGGAGTATGGGAAGCCCAACACTCTCATCTGCTTTGTTAGTAATATCTTCCCACCTCAAGTAACTGTGAGCTGGCAGTACCAACAAGTCCCTGTGCAAAGCAGCAGCCCTACTTTTCTCTCAGCTATAGATGGACTTGCCTTCCAGGCCTTCTCTTATCTGAACTTCACACCCACATCCTCTGATGTCTTCTCTTGCACTGTGACCTGGGAAGGTGACCTCTTCAGCACTATAGCCTTCTGGGTGCCTCAGAATCCTATACCCTCTGAATTGTTGGAAAATATATTGTGTGGCATTGCCCTTGGCCTGGGAATTGTTGGCATCATAGTGGGTACTGCGCTCATCATCTACTTCCAAAAACCATGTGCAAGTGGTGCAGACTGA

>DMA_1_Hap_3

ATGGGACCTGAGCAAAGCCTGGGAGCTATGCTGCTACTGCTGCAGCTACAGTCATCCCTGCTTTTATCCCTGTCCTGGGGAGCCACTCCAGTGTTGGCATCATTATTGGAGAATAGCCTACAGAACTACACGTTCTCTCACACACTGTTCTGCCAGGATGAGGATCCTGTCCTGAGTCTGTCAGAGGCCTTCAATGAAGACCAGCTCTTCTCCTTTGACTTCTCCAGGAACTCCCGGGTGCCCCGGCTGCCTGAGTTTGCTCCTTGGGCCAGTGACAAGGGAGATATCGAGGCCATAAAAGCTGACCAGCGGCTCTGCCAGGAACTGCAAAAAGAATTGAGTAAACTATTGGAAGGCCACATCCCTGAGGCTAGAGGAAACCCTGTGGCTGAAGTTTTCACTCTGGAGCCCCTGGAGTATGGGAAGCCCAACACTCTCATCTGCTTTGTTAGTAATATCTTCCCACCTCAAGTAACTGTGAGCTGGCAGTACCAACAAGTCCCTGTGCAAAGCAGCAGCCCTACTTTTCTCTCAGCTATAGATGGACTTGCCTTCCAGGCCTTCTCTTATCTGAACTTCACACCCACATCCTCTGATGTCTTCTCTTGCACTGTGACCTGGGAAGGTGACCTCTTCAGCACTATAGCCTTCTGGGTGCCTCAGAATCCTATACCCTCTGAATTGTTGGAAAATATATTGTGTGGCATTGCCCTTGGCTTGGGAATTGTTGGCATCATAGTGGGTACTGCGCTCATCATCTACTTCCAAAAACCATGTGCAAGTGGTGCAGACTGA

>DMB_1_Hap_1

ATGCTCGAGCAAAGAATGAGGTTACTCCACCTACTACTAGTGGGCTTCAGCCTGGGTTTTTCAGGAGCAGCAGGGGGCTTTGTGACCCATGTGGAGAGTGGCTGTATGCTGGATGAAGAAGGATCAGTAAAGGACTTCACATATTGTATCTCCTTCAACAAGGATGTGCTGACCTGCTGGGACTCAGAGGCTAACAAGATGGCTGCTGTTGATTTTGGGATCCTGAATCCATTAGCTAAAAAGCTTTCTGAAATCTTCAGTAACGATAGTGCTTTGATGGACCGTCTGAGCAAAGGACTCCAGGACTGCGCCATTCACACAAAGCCCTTCTGGGGAGCGTTGACCCAAAGGACACGGCCACCATCAGTGCAAATAGCCCAGACCACACCTTTCAACACAAGGGAGTCAGTGATGCTGGCCTGTTATGTATGGGGCTTCTATCCTGCTGATGTGGCCATTTTATGGTTGAAGAATGGGCAGCCTATCCCAAACAGTGGCATCCAGAAGGCTGTACAATCCAATGGGGACTGGACTTATCAGACACGATCCTACTTACCCCTTACCCCCTCTAGTGGGGATATTTACACTTGCCATGTAGAGCACAGTGGAACTTCCCAGCCTATCTTGCAAACCTGGACACCTGGCCTCTCTCTGAAGCAGACAGTGAAGATCTCTGTGTCTGTATTGACTCTGAGCCTTGGACTCACCTTCTTCTTCCTTGGCCTGGTTGCCTGCCGAAAAGCTGGCCCCTCTGACTACACTCCTCTCTCGGGGTCCAATTATCCTGAAGGTAGAAACTTCTCATAA

>DMB_1_Hap_2

ATGCTCGAGCAAAGAATGAGGTTACTCCACCTACTACTAGTGGGCTTCAGCCTGGGTTTTTCAGGAGCAGCAGGGGGCTTTGTGACCCATGTGGAGAGTGGCTGTATGCTGGATGAAGAAGGATCAGTAAAGGACTTCACATATTGTATCTCCTTCAACAAGGATGTGCTGACCTGCTGGGACTCAGAGGCTAACAAGATGGCTGCTGTTGATTTTGGGATCCTGAATCCATTAGCTAAAAAGCTTTCTGAAATCCTCAGTAACGATAGTGCTTTGATGGACCGTCTGAGCAAAGGACTCCAGGACTGCGCCATTCACACAAAGCCCTTCTGGGGAGCGTTGACCCAAAGGACACGGCCACCATCAGTGCAAATAGCCCAGACCACACCTTTCAACACAAGGGAGTCAGTGATGCTGGCCTGTTATGTATGGGGCTTCTATCCTGCTGATGTGGCCATTTTATGGTTGAAGAATGGGCAGCCTATCCCAAACAGTGGCATCCAGAAGGCTGTACAATCCAATGGGGACTGGACTTATCAGACACGATCCTACTTACCCCTTACCCCCTCTAGTGGGGATATTTACACTTGCCATGTAGAGCACAGTGGAACTTCCCAGCCTATCTTGCAAACCTGGACACCTGGCCTCTCTCTGAAGCAGACAGTGAAGATCTCTGTGTCTGTATTGACTCTGAGCCTTGGACTCACCTTCTTCTTCCTTGGCCTGGTTGCCTGCCGAAAAGCTGGCCCCTCTGACTACACTCCTCTCTCGGGGTCCAATTATCCTGAAGGTAGAAACTTCTCATAA

>DMB_1_Hap_4

ATGCTCGAGCAAAGAATGAGGTTACTCCACCTACTACTAGTGGGCTTCAGCCTGGGTTTTTCAGGAGCAGCAGGGGGCTTTGTGACCCATGTGGAGAGTGGCTGTATGCTGGATGAAGAAGGATCAGTAAAGGACTTCACATATTGTATCTCCTTCAACAAGGATGTGCTGACCTGCTGGGACTCAGAGGCTAACAAGATGGCTGCTGTTGATTTTGGGATCCTGAATCCATTAGCTAAAGAGCTTTCTGAAATCCTCAGTAACGATAGTGCTTTGATGGACCGTCTGAGCAAAGGACTCCAGGACTGCGCCATTCACACAAAGCCCTTCTGGGGAGCGTTGACCCAAAGGACACGGCCACCATCAGTGCAAATAGCCCAGACCACACCTTTCAACACAAGGGAGTCAGTGATGCTGGCCTGTTATGTATGGGGCTTCTATCCTGCTGATGTGGCCATTTTATGGTTGAAGAATGGGCAGCCTATCCCAAACAGTGGCATCCAGAAGGCTGTACAATCCAATGGGGACTGGACTTATCAGACACGATCCTACTTACCCCTTACCCCCTCTAGTGGGGATATTTACACTTGCCATGTAGAGCACAGTGGAACTTCCCAGCCTATCTTGCAAACCTGGACACCTGGCCTCTCTCTGAAGCAGACAGTGAAGATCTCTGTGTCTGTATTGACTCTGAGCCTTGGACTCACCTTCTTCTTCCTTGGCCTGGTTGCCTGCCGAAAAGCTGGCCCCTCTGACTACACTCCTCTCTCGGGGTCCAATTATCCTGAAGGTAGAAACTTCTCATAA

>DMB_1_Hap_3

ATGCTCGAGCAAAGAATGAGGTTACTCCACCTACTACTAGTGGGCTTCAGCCTGGGTTTTTCAGGAGCAGCAGGGGGCTTTGTGACCCATGTGGAGAGTGGCTGTATGCTGGATGAAGAAGGATCAGTAAAGGACTTCACATATTGTATCTCCTTCAACAAGGATGTGCTGACCTGCTGGGACTCAGAGGCTAACAAGATGGCTGCTGTTGATTTTGGGATCCTGAATCCATTAGCTAAAAAGCTTTCTGAAATCCTCAGTAACGATAGTGCTTTGATGGACCGTCTGAGCAAAGGACTCCAGGACTGCGCCATTCACACAAAGCCCTTCTGGGGAGCGTTGACCCAAAGGACACGGCCACCATCAGTGCAAATAGCCCAGACCACACCTTTCAACACAAGGGAGTCAGTGATGCTGGCCTGTTATGTATGGGGCTTCTATCCTGCTGATGTGGCCATTTTATGGTTGAAGAATGGGCAGCCTATCCCAAACAGTGGCATCCAGAGGGCTGTACAATCCAATGGGGACTGGACTTATCAGACACGATCCTACTTACCCCTTACCCCCTCTAGTGGGGATATTTACACTTGCCATGTAGAGCACAGTGGAACTTCCCAGCCTATCTTGCAAACCTGGACACCTGGCCTCTCTCTGAAGCAGACAGTGAAGATCTCTGTGTCTGTATTGACTCTGAGCCTTGGACTCACCTTCTTCTTCCTTGGCCTGGTTGCCTGCCGAAAAGCTGGCCCCTCTGACTACACTCCTCTCTCGGGGTCCAATTATCCTGAAGGTAGAAACTTCTCATAA

>DMB_1_Hap_5

ATGCTCGAGCAAAGAATGAGGTTACTCCACCTACTACTAGTGGGCTTCAGCCTGGGTTTTTCAGGAGCAGCAGGGGGCTTTGTGACCCATGTGGAGAGTGGCTGTATGCTGGATGAAGAAGGATCAGTAAAGGACTTCACATATTGTATCTCCTTCAACAAGGATGTGCTGACCTGCTGGGACTCAGAGGCTAACAAGATGGCTGCTGTTGATTTTGGGATCCTGAATCCATTAGCTAAAGAGCTTTCTGAAATCCTCAGTAACGATAGTGCTTTGATGGACCGTCTGAGCAAAGGACTCCAGGACTGCGCCATTCACACAAAGCCCTTCTGGGGAGCGTTGACCCAAAGGACACGGCCACCATCAGTGCAAATAGCCCAGACCACACCTTTCAACACAAGGGAGTCAGTGATGCTGGCCTGTTATGTATGGGGCTTCTATCCTGCTGATGTGGCCATTTTATGGTTGAAGAATGGGCAGCCTATCCCAAACAGTGGCATCCAGAGGGCTGTACAATCCAATGGGGACTGGACTTATCAGACACGATCCTACTTACCCCTTACCCCCTCTAGTGGGGATATTTACACTTGCCATGTAGAGCACAGTGGAACTTCCCAGCCTATCTTGCAAACCTGGACACCTGGCCTCTCTCTGAAGCAGACAGTGAAGATCTCTGTGTCTGTATTGACTCTGAGCCTTGGACTCACCTTCTTCTTCCTTGGCCTGGTTGCCTGCCGAAAAGCTGGCCCCTCTGACTACACTCCTCTCTCGGGGTCCAATTATCCTGAAGGTAGAAACTTCTCATAA

>MHCI_1_UI_Hap_1

ATGAACCTTTACGTGTGCTCTCTCTTTTTGTTGGGGACCCTGGCCCTGGGGGAGACCTGGGCAGGCTCTCACTCCCTGAGGTATTTCTCCGCCGCTGTGTCCCGGCCCGAGCTCGGGGAGCCGCGGTTCTTCTCCGTGGGCTACGTGGACGATCAGCAGTTCGTGGGCTTTGACAGCGACAGCGAGAGTCAGAGGCAGGAGCCGCGGGCGCCGTGGATGGAGCAGCTGGAGCCTGACTACTGGGAGCGGAACACGCGCATCTCCAGGGCGAACACACAGAGTTCCCGAGTGACCCTGCGGAACATGCGCAGCTACTACAACCAGAGCGAGGGCGGGGTCCACATCTTCCAGACCATGTACGGCTGCGAGGTTTCCCCCGACGTCACCTTCAAGCGAGGTTTTTTTCAACACGCCTACGACGGGCACGACTACCTCTCCCTGGACACGGAGACCTACACGTGGACGGCGTCGGTGCCCCAGGCTCTGAACTCCAAGCGCAAGTGGGAGGCGGAGAGGAGCATAGTAGAGGGATATAAAGCCTATCTGGAGGAGACGTGCGTGCTGTGGCTGAAGAAGTACCTGGAAATGGGGAAGGAGACGCTGAAGAAGGCAGATCCGCCCTCTACCCGAGTGACCCACCACACTGCCCCCAATGGGGAAGTGACCCTGCGGTGCCGGGCCCAGGACTTCTACCCTGCCGACATCTCCCTGACTTGGCTGAGGGATGGGGAGGAACAACTCCAGGACACAGAGTTCATTGAGACCAGGCCTGCAGGAGACGGGACCTTCCAGAAGTGGGCAGGTGTGGATGTGACCTCGGGCCAGGAAGGGAAATATACCTGCCGAGTTCAGCACGAGGGACTGCCTGAGCCCCTCACCCTGAAGTGGGAGCCAGAGTCCTCATCCCCCTGGCTCATTATGGGGGGTGTTGCTTTTGTCCTCCCCGTTGCACTCATTGCTGGAGTTGGGATCTGGAGGAAGAAGACTTCAGGTGGAAAAGGAGGGGACTATGTTCCTGCTACAGGCAATGAGAGTGCACAGGGGTCAGATGTGTCCCCTACAGCCAAAGGTTGA

>MHCI_1_UI_Hap_2

ATGAACCTTTACGTGTGCTCTCTCTTTTTGTTGGGGACCCTGGCCCTGGGGGAGACCTGGGCAGGCTCTCACTCCCTGAGGTATTTCTCCGCCGCTGTGTCCCGGCCCGAGCTCGGGGAGCCGCGGTTCTTCTCCGTGGGCTACGTGGACGATCAGCAGTTCGTGGGCTTTGACAGCGACAGCGAGAGTCAGAGGCAGGAGCCGCGGGCGCCGTGGATGGAGCAGCTGGAGCCTGACTACTGGGAGCGGAACACGCGCATCTCCAGGGCGAACGCACAGAGTTCCCGAGTGACCCTGCGGAACATGCGCAGCTACTACAACCAGAGCGAGGGCGGGGTCCACATCTTCCAGACCATGTACGGCTGCGAGGTTTCCCCCGACGTCACCTTCAAGCGAGGTTTTTTTCAACACGCCTACGACGGGCACGACTACCTCTCCCTGGACACGGAGACCTACACGTGGACGGCGTCGGTGCCCCAGGCTCTGAACTCCAAGCGCAAGTGGGAGGCGGAGAGGAGCATAGTAGAGGGATATAAAGCCTATCTGGAGGAGACGTGCGTGCTGTGGCTGAAGAAGTACCTGGAAATGGGGAAGGAGACGCTGAAGAAGGCAGATCCGCCCTCTACCCGAGTGACCCACCACACTGCCCCCAATGGGGAAGTGACCCTGCGGTGCCGGGCCCAGGACTTCTACCCTGCCGACATCTCCCTGACTTGGCTGAGGGATGGGGAGGAACAACTCCAGGACACAGAGTTCATTGAGACCAGGCCTGCAGGAGACGGGACCTTCCAGAAGTGGGCAGGTGTGGATGTGACCTCGGGCCAGGAAGGGAAATATACCTGCCGAGTTCAGCACGAGGGACTGCCTGAGCCCCTCACCCTGAAGTGGGAGCCAGAGTCCTCATCCCCCTGGCTCATTATGGGGGGTGTTGCTTTTGTCCTCCCCGTTGCACTCATTGCTGGAGTTGGGATCTGGAGGAAGAAGACTTCAGGTGGAAAAGGAGGGGACTATGTTCCTGCTACAGGCAATGAGAGTGCACAGGGGTCAGATGTGTCCCCTACAGCCAAAGGTTGA

>MHCI_10_UF_Hap_3

ATGTATGTGCTGTCTCTACTTTTGTTGGGTGTCCTGGTCCTTACGGAGACCTCGGCTGGCTCTCACTCCCTGAAGTATTTCTACGCTGTCATGTCTCGCCCCGAGCTAGCAAAACCAAGGTTCATCGCCGTAACCTACGTGGACGATCAGCAGGTCTTGAGCTTCGACAGCGACGGTGAGAGCCAGAGCACGGAGCCCAGGACGCCATGGATCAAGCAGAAGATCTCGGATTACTGGGAGCGTGAGACGCGGATCTCCAGGGAAGCCACGCAGCGTTATCGAATGTGCCTGCGGAAAGTGTCTGCGTACTACAACCATAGTGAGGGAGGGGTTCACACATACCAGCGACTGTCGGGCTGCGAGGCATTCTCCAACGGGAGCTTCAGCCGCGGCTTCGTGCAATATGCCTACGACGGACAGGACTACCTGGCCCTGGATACCGAGACTCTGCGCTGGATCGCGGGGAACACAGGGGCCCTGAACAATAAGCGCAAGTGGGAGGCAGATCAAAGTGTTGCGAAATATTGGAAGGGATATATGGAGAAGGAGTGCGTGTACTGGCTTCACAGATACCTGGAGAACGGAAAGGACACACTGCTTCGGACAGATCCACCTTCTGTACAAGTGACCAGACACACAAGTTCTGACGGAGAAGTGACCTTGAGGTGCCGGGCCCAGGGCTTTTATCCTGCAGAGATCTCACTGACTTGGCTGAGGGATGGGGAGGAACAGCTGCAGGAGACGGAGCTCATTGATACCAGACCTGCGGGAGATGGGACCTTCCAGAAGTGGGCAGCTGTGGGGATGCTCTCTGGAAGTGAACAGAAATATGCCTGCCGAGTGCAGCACAAGGGACTACCTGAGCCACTCTTCCTGAAATGGGAGCCACAGTCCTCACCCGTAAGGGCTCTCTACAGGAGTCATCATTGCTCTCCTCCTCCTCCTCGCTGCAGTCATTGTCGGGGTTGTGATCTGGAGGAAAAATACTTCAGACGAAAAAAGAGGGAGGTATACTACAACTGCAGTAGTGATAGTGCCCAGGGATCAGATGTCTCTCTTACAGCAAAAGCCTAA

>MHCI_10_UF_Hap_1

ATGTATGTGCTGTCTCTACTTTTGTTGGGTGTCCTGGTCCTTACGGAGACCTCGGCTGGCTCTCACTCCCTGAAGTATTTCTACGCTGTCATGTCTCGCCCCGAGCTAGCAAAACCAAGGTTCATCGCCGTAACCTACGTGGACGATCAGCAGGTCTTGAGCTTCGACAGCGACGGTGAGAGCCAGAGCACGGAGCCCAGGACGCCATGGATCAAGCAGAAGATCTCGGATTACTGGGAGCGTGAGACGCGGATCTCCAGGGAAGCCACGCAGCGTTATCGAATGTGCCTGCGGAAAGTGTCTGCGTACTACAACCATAGTGAGGGAGGGGTTCACACATACCAGCGACTGTCGGGCTGCGAGGCATTCTCCAACGGGAGCTTCAGCCGCGGCTTCGTGCAATATGCCTACGACGGACAGGACTACCTGGCCCTGGATACCGAGACTCTGCGCTGGATCGCGGGGAACACAGGGGCCCTGAACAATAAGCGCAAGTGGGAGGCAGATCAAAGTGTTGCGAAATATTGGAAGGGATATATGGAGAAGGAGTGCGTGTACTGGCTTCACAGATACCTGGAGAACGGAAAGGACACACTGCTTCGGACAGATCCACCTTCTGTACAAGTGACCAGACACACAAGTTCTGACGGAGAAGTGACCTTGAGGTGCCGGGCCCAGGGCTTTTATCCTGCAGAGATCTCACTGACTTGGCTGAGGGATGGGGAGGAACAGCTGCAGGAGACGGAGCTCATTGATACCAGACCTGCGGGAGATGGGACCTTCCAGAAGTGGGCAGCTGTGGGGATGCTCTCTGGAAGTGAACAGAAATATGCCTGCCGAGTGCAGCACAAGGGACTACCTGAGCCACTCTTCCTGAAATGGGAGCCACAGTCCTCACCCGTAAGGGCTCTCTACAGGAGTCATCATTGCTCTCCTCCTCCTCCTCGCTGCAGTCATTGTCGGGGTTGTGATCTGGAGGAAAAATACTTCAGATGAAAAAAGAGGGAGGTATACTACAACTGCAGTAGTGATAGTGCCCAGGGATCAGATGTCTCTCTTACAGCAAAAGCCTAA

>MHCI_10_UF_Hap_2

ATGTATGTGCTGTCTCTACTTTTGTTGGGTGTCCTGGTCCTTACGGAGACCTCGGCTGGCTCTCACTCCCTGAAGTATTTCTACGCTGTCATGTCTCGCCCCGAGCTAGCAAAACCAAGGTTCATCGCCGTAACCTACGTGGACGATCAGCAGGTCTTGAGCTTCGACAGCGACGGTGAGAGCCAGAGCACGGAGCCCAGGACGCCATGGATCAAGCAGAAGATCTCGGATTACTGGGAGCGTGAGACGCGGATCTCCAGGGAAGCCACGCAGCGTTATCGAATGTGCCTGCGGAAAGTGTCTGCGTACTACAACCATAGTGAGGGAGGGGTTCACACATACCAGCGACTGTCGGGCTGCGAGGCATTCTCCAACGGGAGCTTCAGCCGCGGCTTCGTGCAATATGCCTACGACGGACAGGACTACCTGGCCCTGGATACCGAGACTCTGCGCTGGATCGCGGGGAACACAGGGGCCCTGAACAATAAGCGCAAGTGGGAGGCAGATCAAAGTGTTGCGAAATATTGGAAGGGATATATGGAGAAGGAGTGCGTGTACTGGCTTCACAGATACCTGGAGAACGGAAAGGACACACTGCTTCGGACAGATCCACCTTCTGTACAAGTGACCAGACACACAAGTTCTGACGGAGAAGTGACCTTGAGGTGCCGGGCCCAGGGCTTTTATCCTGCAGAGATCTCACTGACTTGGCTGAGGGATGGGGAGGAGCAGCTGCAGGAGACGGAGCTCATTGATACCAGACCTGCGGGAGATGGGACCTTCCAGAAGTGGGCAGCTGTGGGGATGCTCTCTGGAAGTGAACAGAAATATGCCTGCCGAGTGCAGCACAAGGGACTACCTGAGCCACTCTTCCTGAAATGGGAGCCACAGTCCTCACCCGTAAGGGCTCTCTACAGGAGTCATCATTGCTCTCCTCCTCCTCCTCGCTGCAGTCATTGTCGGGGTTGTGATCTGGAGGAAAAATACTTCAGATGAAAAAAGAGGGAGGTATACTACAACTGCAGTAGTGATAGTGCCCAGGGATCAGATGTCTCTCTTACAGCAAAAGCCTAA

>MHCI_12_UH_Hap_1

ATGGAGCGTTATCTGTGCTCTTTGTTTTTGTTGGGGACCCTGGCCCTGCCCGAGACCTGGGCAGGCTCTCACTCCCTGAGGTATTACTACATCGCCATGACCTCGCCCGAGCTCGCGGAGCCGCGGTTTCTCGCCGTGGGCTACGTGGACGATCAGCAGGGCGGGCGCTTCGACAGTGCCAGCGCGAATCAGAGGGCGGAGCCGCGGGCGGCGTGGGTAGAGCAGGTGGAGCAGGAGGAGCCGGGCTACTGGGAGCGGAACACGCGGATCGCCAGGGGAAACACACAGGCTTACCGAGTGTACCTGCAGACCCTGCGCGGCTACTTCAACCAGAGCGAAGGCGGGGTCCACACCATCCAGATCATGTACGGCTGCGAGGTCTCACCGGAGCTCACCTTCAAGCGGGGGTTTTACCAGTACGCCTACGACGGGCAAGACTACATCGCTCTGGACAGGGAGACCTCCACGTGGACGGCGGCGGTGCCGCAGGCTGTGAACACCAAGCTGGAGTGGGAGGCGGACAGGGGCTACACGGAGAGAAAGAAAGCCTATCAGCAGGAGGAGTGCGTGCTGTGGCTGAAGAAGTACCTGGATATGGGGAAGGAGACTCTGAAGAGGGCAGACCCGCCCTCAGTCCGAGTGACCCGCCACACTGGCCCCAACGGGGAGGTGGCCTTGCGGTGTCGAGCCCAGAACTTCTACCCCGAGGACATCTCCCTGACTTGGCTGAGGGATGGGGAGGAGCAGCTCCAGGACACGGAGTTCATTGAGACCAGGCCTGCTGGGGACGGGACCTTCCAGAAGTGGGCAGGTGTGGACGTGACCTCGGGCCAGGAAGGGAAATATACCTGCCGAGTTCAGCACGAGGGACTGCCTGAGCCCCTCACCCTGAAGTGGGAGCCAGAGTCCTCATCTACCCGGGTCATCGTGGGGGCCATTGCTGTCCTCATCTTCATCACTGCAGTCATTGCTGGAGTTGTGATCTGGAGGAAGAACACTTCAGGTGGAAAAGGAGGGAACTATGTTCCTGCTGCAGGCAATGACTGTGCACAGGGGTCACATGTCTTCCCCACAGCCAAAGCTTGA

>MHCI_12_UH_Hap_4

ATGGAGCGTTATCTGTGCTCTTTGTTTTTGTTGGGGACCCTGGCCCTGCCCGAGACCTGGGCAGGCTCTCACTCCCTGAGGTATTACTACATCGCCATGACCTCGCCCGAGCTCGCGGAGCCGCGGTTTCTCGCCGTGGGCTACGTGGACGATCAGCAGGGCGGGCGCTTCGACAGTGCCAGCGCGAATCAGAGGGCGGAGCCGCGGGCGGCGTGGGTAGAGCAGGTGGAGCAGGAGGAGCCGGGCTACTGGGAGCGGAACACGCGGATCGCCAGGGGGAACACACAGGCTTACCGAGTGTACCTGCAGACCCTGCGCGGCTACTTCAACCAGAGCGAAGGCGGGGTCCACACCATCCAGATCATGTACGGCTGCGAGGTCTCACCGGAGCTCACCTTCAAGCGGGGGTTTTACCAGTACGCCTACGACGGGCAAGACTACATCGCTCTGGACAGGGAGACCTCCACGTGGACGGCGGCGGTGCCGCAGGCTGTGAACACCAAGCTGGAGTGGGAGGCGGACAGGGGCTACACGGAGAGAAAGAAAGCCTATCAGCAGGAGGAGTGCGTGCTGTGGCTGAAGAAGTACCTGGATATGGGGAAGGAGACTCTGAAGAGGGCAGACCCGCCCTCAGTCCGAGTGACCCGCCACACTGGCCCCAACGGGGAGGTGGCCTTGCGGTGTCGAGCCCAGAACTTCTACCCCGAGGACATCTCCCTGACTTGGCTGAGGGATGGGGAGGAGCAGCTCCAGGACACGGAGTTCATTGAGACCAGGCCTGCTGGGGACGGGACCTTCCAGAAGTGGGCAGGTGTGGACGTGACCTCGGGCCAGGAAGGGAAATATACCTGCCGAGTTCAGCACGAGGGACTGCCTGAGCCCCTCACCCTGAAGTGGGAGCCAGAGTCCTCATCTACCCGGGTCATCGTGGGGGCCATTGCTGTCCTCATCTTCATCACTGCAGTCATTGCTGGAGTTGTGATCTGGAGGAAGAACACTTCAGGTGGAAAAGGAGGGAACTATGTTCCTGCTGCAGGCAATGACTGTGCACAGGGGTCACATGTCTTCCCCACAGCCAAAGCTTGA

>MHCI_12_UH_Hap_10

ATGGAGCGTTATCTGTGCTCTTTGTTTTTGTTGGGGACCCTGGCCCTGCCCGAGACCTGGGCAGGCTCTCACTCCCTGAGGTATTACTACATCGCCATGACCTCGCCCGAGCTCGCGGAGCCGCGGTTTCTCGCCGTGGGCTACGTGGACGATCAGCAGGGCGGGCGCTTCGACAGTGCCAGCGCGAATCAGAGGGCGGAGCCGCGGGCGGCGTGGGTAGAGCAGGTGGAGCAGGAGGAGCCGGGCTACTGGGAGCGAAACACGCGGATCGCCAGGGGGAACACACAGGCTTACCGAGTGTACCTGCAGACCCTGCGCGGCTACTTCAACCAGAGCGAAGGCGGGGTCCACACCATCCAGATCATGTACGGCTGCGAGGTCTCACCGGAGCTCACCTTCAAGCGGGGGTTTTACCAGTACGCCTACGACGGGCAAGACTACATCGCTCTGGACAGGGAGACCTCCACGTGGACGGCGGCGGTGCCGCAGGCTGTGAACACCAAGCTGGAGTGGGAGGCGGACAGGGGCTACACGGAGAGAAAGAAAGCCTATCAGCAGGAGGAGTGCGTGCTGTGGCTGAAGAAGTACCTGGATATGGGGAAGGAGACTCTGAAGAGGGCAGACCCGCCCTCAGTCCGAGTGACCCGCCACACTGGCCCCAACGGGGAGGTGGCCTTGCGGTGTCGAGCCCAGAACTTCTACCCCGAGGACATCTCCCTGACTTGGCTGAGGGATGGGGAGGAGCAGCTCCAGGACACGGAGTTCATTGAGACCAGGCCTGCTGGGGACGGGACCTTCCAGAAGTGGGCAGGTGTGGACGTGACCTCGGGCCAGGAAGGGAAATATACCTGCCGAGTTCAGCACGAGGGACTGCCTGAGCCCCTCACCCTGAAGTGGGAGCCAGAGTCCTCATCTACCCGGGTCATCGTGGGGGCCATTGCTGTCCTCATCTTCATCACTGCAGTCATTGCTGGAGTTGTGATCTGGAGGAAGAACACTTCAGGTGGAAAAGGAGGGAACTATGTTCCTGCTGCAGGCAATGACTGTGCACAGGGGTCACATGTCTTCCCCACAGCCAAAGCTTGA

>MHCI_12_UH_Hap_2

ATGGAGCGTTATCTGTGCTCTTTGTTTTTGTTGGGGACCCTGGCCCTGCCCGAGACCTGGGCAGGATCTCATTCCCTGAGGTATTACTACATCGCCATGACCTCTCCCGAGCTCGCGGAGCCGCGGTTTCTCGCCGTGGGCTACGTGGACGATCAGCAGGGCGGGCGCTTCGACAGTGCCAGCGCTAATCAGAGGGCGGAGCCGCGGGCGGCGTGGATAGAGAAGGTGGAGGAGGAGGAGCCGGGCTACTGGGAGCGAAACACGCGAATCGCCAGGGGGAACACACAGGTTTACCGAGTGTACCTCCAGACCTTGCGCGGCTACTTCAATCAGAGCGAGGGCGGGGTCCACACCATCCAGGTTATGTTCGGCTGCGAGGTCTCCCCGGACCTCACCTTCAAGCGAGGGTTTTTCCAATACGCCTACGACGGGCAAGACTACATCGCCCTGGACAGGGAGACCTCCACATGGACGGCGGCGGTGCCCCAGGCTGTGAACACCAAGCTGGAGTGGGAGGCGGACAGGAGCATCGCTGAGAGACGGAAAGCCTATCTGGAGGAGGAGTGCGTGATGTGGCTGAAGAAGTACCTGGATATGGGGAAGGAGATGCTGAAGAGGGCAGACCCGCCCGCAGTCCGAGTGACCCGCCACACTGGCCCCAACGGGGAGGTGGCCTTGCGGTGTCGAGCCCAGAACTTCTACCCCGAGGACATCTCCCTGACTTGGCTGAGGGATGGGGAGGAGCAGCTCCAGGACACGGAGTTCATTGAGACCAGGCCTTCGGGAGACGGGACCTTCCAGAAGTGGGCAGGTGTGGACGTGACCTCGGGCCAGGAAGGGAAATATACCTGCCGAGTTCAGCACGAGGGACTGCCTGAGCCCCTCACCCTGAAGTGGGAGCCAGAGTCCTCATCTACCCGGGTCATCGTGGGGGCCATTGCTGTCCTCATCTTCATCACTGCAGTCATTGCTGGAGTTGTGATCTGGAGGAAGAACACTTCAGGTGGAAAAGGAGGGAACTATGTTCCTGCTGCAGGCAATGACAGTGCACAGGGGTCACATGTCTTCCCCACAGCCAAAGCTTGA

>MHCI_12_UH_Hap_3

ATGGAGCGTTATCTGCGTTCTCTCTTTTTGTTGGGTACCCTGGCCCTGTCGGAGACCTGGGCAGGATCTCATTCCCTGAGGTATTACTACATCGCCATGACCTCTCCCGAGCTCGCGGAGCCGCGGTTTCTCGCCGTGGGCTACGTGGACGATCAGCAGGGCGGGCGCTTCGACAGTGCCAGCGCTAATCAGAGGGCGGAGCCGCGGGCGGCGTGGATAGAGAAGGTGGAGGAGGAGGAGCCGGGCTACTGGGAGCGAAACACGCGAATCGCCAGGGGGAACACACAGGTTTACCGAGTGTACCTCCAGACCTTGCGCGGCTACTTCAATCAGAGCGAGGGCGGGGTCCACACCATCCAGGTTATGTTCGGCTGCGAGGTCTCCCCGGACCTCACCTTCAAGCGAGGGTTTTTCCAATACGCCTACGACGGGCAAGACTACATCGCCCTGGACAGGGAGACCTCCACATGGACGGCGGCGGTGCCCCAGGCTGTGAACACCAAGCTGGAGTGGGAGGCGGACAGGAGCATCGCTGAGAGACGGAAAGCCTATCTGGAGGAGGAGTGCGTGATGTGGCTGAAGAAGTACCTGGATATGGGGAAGGAGATGCTGAAGAGGGCAGACCCGCCCTCAGTCCGAGTGACCCGCCACACTGGCCCCAACGGGGAGGTGGCCTTGCGGTGTCGAGCCCAGAACTTCTACCCCGAGGACATCTCCCTGACTTGGCTGAGGGATGGGGAGGAGCAGCTCCAGGACACGGAGTTCATTGAGACCAGGCCTTCGGGAGACGGGACCTTCCAGAAGTGGGCAGGTGTGGACGTGACCTCGGGCCAGGAAGGGAAATATACCTGCCGAGTTCAGCACGAGGGACTGCCTGAGCCCCTCACCCTGAAGTGGGAGCCAGAGTCCTCATCTACCCGGGTCATCGTGGGGGCCATTGCTGTCCTCATCTTCATCACTGCAGTCATTGCTGGAGTTGTGATCTGGAGGAAGAACACTTCAGGTGGAAAAGGAGGGAACTATGTTCCTGCTGCAGGCAATGACAGTGCACAGGGGTCACATGTCTTCCCCACAGCCAAAGCTTGA

>MHCI_12_UH_Hap_5

ATGGAGCGTTATCTGTGCTCTTTGTTTTTGTTGGGGACCCTGGCCCTGCCCGAGACCTGGGCAGGATCTCATTCCCTGAGGTATTACTACATCGCCATGACCTCTCCCGAGCTCGCGGAGCCGCGGTTTCTCGCCGTGGGCTACGTGGACGATCAGCAGGGCGGGCGCTTCGACAGTGCCAGCGCTAATCAGAGGGCGGAGCCGCGGGCGGCGTGGATAGAGAAGGTGGAGGAGGAGGAGCCGGGCTACTGGGAGCGAAACACGCGAATCGCCAGGGGGAACACACAGGTTTACCGAGTGTACCTCCAGACCTTGCGCGGCTACTTCAATCAGAGCGAGGGCGGGGTCCACACCATCCAGGTTATGTTCGGCTGCGAGGTCTCCCCGGACCTCACCTTCAAGCGAGGGTTTTTCCAATACGCCTACGACGGGCAAGACTACATCGCCCTGGACAGGGAGACCTCCACATGGACGGCGGCGGTGCCCCAGGCTGTGAACACCAAGCTGGAGTGGGAGGCGGACAGGAGCATCGCTGAGAGACGGAAAGCCTATCTGGAGGAGGAGTGCGTGATGTGGCTGAAGAAGTACCTGGATATGGGGAAGGAGATGCTGAAGAGGGCAGACCCGCCCTCAGTCCGAGTGACCCGCCACACTGGCCCCAACGGGGAGGTGGCCTTGCGGTGTCGAGCCCAGAACTTCTACCCCGAGGACATCTCCCTGACTTGGCTGAGGGATGGGGAGGAGCAGCTCCAGGACACGGAGTTCATTGAGACCAGGCCTTCGGGAGACGGGACCTTCCAGAAGTGGGCAGGTGTGGACGTGACCTCGGGCCAGGAAGGGAAATATACCTGCCGAGTTCAGCACGAGGGACTGCCTGAGCCCCTCACCCTGAAGTGGGAGCCAGAGTCCTCATCTACCCGGGTCATCGTGGGGGCCATTGCTGTCCTCATCTTCATCACTGCAGTCATTGCTGGAGTTGTGATCTGGAGGAAGAACACTTCAGGTGGAAAAGGAGGGAACTATGTTCCTGCTGCAGGCAATGACAGTGCACAGGGGTCACATGTCTTCCCCACAGCCAAAGCTTGA

>MHCI_12_UH_Hap_6

ATGGAGCGTTATCTGCGTTCTCTCTTTTTGTTGGGTACCCTGGCCCTGTCGGAGACCTGGGCAGGATCTCATTCCCTGAGGTATTACTACATCGCCATGACCTCTCCCGAGCTCGCGGAGCCGCGGTTTCTCGCCGTGGGCTACGTGGACGATCAGCAGGGCGGGCGCTTCGACAGTGCCAGCGCTAATCAGAGGGCGGAGCCGCGGGCGGCGTGGATAGAGAAGGTGGAGGAGGAGGAGCCGGGCTACTGGGAGCGAAACACGCGAATCGCCAGGGGAAACACACAGGCTTACCGAGTGTACCTGCAGACCTTGCGCGGCTACTTCAATCAGAGCGAGGGCGGGGTCCACACCATCCAGGTTATGTTCGGCTGCGAGGTCTCCCCGGACCTCACCTTCAAGCGAGGGTTTTTCCAATACGCCTACGACGGGCAAGACTACATCGCCCTGGACAGGGAGACCTCCACATGGACGGCGGCGGTGCCCCAGGCTGTGAACACCAAGCTGGAGTGGGAGGCGGACAGGAGCATCGCTGAGAGACGGAAAGCCTATCTGGAGGAGGAGTGCGTGATGTGGCTGAAGAAGTACCTGGATATGGGGAAGGAGATGCTGAAGAGGGCAGACCCGCCCTCAGTCCGAGTGACCCGCCACACTGGCCCCAACGGGGAGGTGGCCTTGCGGTGTCGAGCCCAGAACTTCTACCCCGAGGACATCTCCCTGACTTGGCTGAGGGATGGGGAGGAGCAGCTCCAGGACACGGAGTTCATTGAGACCAGGCCTTCGGGAGACGGGACCTTCCAGAAGTGGGCAGGTGTGGACGTGACCTCGGGCCAGGAAGGGAAATATACCTGCCGAGTTCAGCACGAGGGACTGCCTGAGCCCCTCACCCTGAAGTGGGAGCCAGAGTCCTCATCTACCCGGGTCATCGTGGGGGCCATTGCTGTCCTCATCTTCATCACTGCAGTCATTGCTGGAGTTGTGATCTGGAGGAAGAACACTTCAGGTGGAAAAGGAGGGAACTATGTTCCTGCTGCAGGCAATGACAGTGCACAGGGGTCACATGTCTTCCCCACAGCCAAAGCTTGA

>MHCI_12_UH_Hap_7

ATGGAGCGTTATCTGTGCTCTTTGTTTTTGTTGGGGACCCTGGCCCTGCCCGAGACCTGGGCAGGATCTCATTCCCTGAGGTATTACTACATCGCCATGACCTCTCCCGAGCTCGCGGAGCCGCGGTTTCTCGCCGTGGGCTACGTGGACGATCAGCAGGGCGGGCGCTTCGACAGTGCCAGCGCTAATCAGAGGGCGGAGCCGCGGGCGGCGTGGATAGAGAAGGTGGAGGAGGAGGAGCCGGGCTACTGGGAGCGAAACACGCGAATCGCCAGGGGGAACACACAGGTTTACCGAGTGTACCTCCAGACCTTGCGCGGCTACTTCAATCAGAGCGAGGGCGGGGTCCACACCATCCAGGTTATGTTCGGCTGCGAGGTCTCCCCGGACCTCACCTTCAAGCGAGGGTTTTTCCAATACGCCTACGACGGGCAAGACTACATCGCCCTGGACAGGGAGACCTCCACATGGACGGCGGCGGTGCCCCAGGCTGTGAACACCAAGCTGGAGTGGGAGGCGGACAGGAGCATCGCTGAGAGACGGAAAGCCTATCTGGAGGAGGAGTGCGTGATGTGGCTGAAGAAGTACCTGGATATGGGGAAGGAGACTCTGAAGAGGGCAGACCCGCCCGCAGTCCGAGTGACCCGCCACACTGGCCCCAACGGGGAGGTGGCCTTGCGGTGTCGAGCCCAGAACTTCTACCCCGAGGACATCTCCCTGACTTGGCTGAGGGATGGGGAGGAGCAGCTCCAGGACACGGAGTTCATTGAGACCAGGCCTTCGGGAGACGGGACCTTCCAGAAGTGGGCAGGTGTGGACGTGACCTCGGGCCAGGAAGGGAAATATACCTGCCGAGTTCAGCACGAGGGACTGCCTGAGCCCCTCACCCTGAAGTGGGAGCCAGAGTCCTCATCTACCCGGGTCATCGTGGGGGCCATTGCTGTCCTCATCTTCATCACTGCAGTCATTGCTGGAGTTGTGATCTGGAGGAAGAACACTTCAGGTGGAAAAGGAGGGAACTATGTTCCTGCTGCAGGCAATGACAGTGCACAGGGGTCACATGTCTTCCCCACAGCCAAAGCTTGA

>MHCI_12_UH_Hap_8

ATGGAGCGTTATCTGCGTTCTCTCTTTTTGTTGGGTACCCTGGCCCTGTCGGAGACCTGGGCAGGATCTCATTCCCTGAGGTATTACTACATCGCCATGACCTCTCCCGAGCTCGCGGAGCCGCGGTTTCTCGCCGTGGGCTACGTGGACGATCAGCAGGGCGGGCGCTTCGACAGTGCCAGCGCTAATCAGAGGGCGGAGCCGCGGGCGGCGTGGATAGAGAAGGTGGAGGAGGAGGAGCCGGGCTACTGGGAGCGAAACACGCGAATCGCCAGGGGGAACACACAGGTTTACCGAGTGTACCTCCAGACCTTGCGCGGCTACTTCAATCAGAGCGAGGGCGGGGTCCACACCATCCAGGTTATGTTCGGCTGCGAGGTCTCCCCGGACCTCACCTTCAAGCGAGGGTTTTTCCAATACGCCTACGACGGGCAAGACTACATCGCCCTGGACAGGGAGACCTCCACATGGACGGCGGCGGTGCCCCAGGCTGTGAACACCAAGCTGGAGTGGGAGGCGGACAGGAGCATCGCTGAGAGACGGAAAGCCTATCTGGAGGAGGAGTGCGTGATGTGGCTGAAGAAGTACCTGGATATGGGGAAGGAGACTCTGAAGAGGGCAGACCCGCCCGCAGTCCGAGTGACCCGCCACACTGGCCCCAACGGGGAGGTGGCCTTGCGGTGTCGAGCCCAGAACTTCTACCCCGAGGACATCTCCCTGACTTGGCTGAGGGATGGGGAGGAGCAGCTCCAGGACACGGAGTTCATTGAGACCAGGCCTTCGGGAGACGGGACCTTCCAGAAGTGGGCAGGTGTGGACGTGACCTCGGGCCAGGAAGGGAAATATACCTGCCGAGTTCAGCACGAGGGACTGCCTGAGCCCCTCACCCTGAAGTGGGAGCCAGAGTCCTCATCTACCCGGGTCATCGTGGGGGCCATTGCTGTCCTCATCTTCATCACTGCAGTCATTGCTGGAGTTGTGATCTGGAGGAAGAACACTTCAGGTGGAAAAGGAGGGAACTATGTTCCTGCTGCAGGCAATGACAGTGCACAGGGGTCACATGTCTTCCCCACAGCCAAAGCTTGA

>MHCI_12_UH_Hap_9

ATGGAGCGTTATCTGTGCTCTTTGTTTTTGTTGGGGACCCTGGCCCTGCCCGAGACCTGGGCAGGATCTCATTCCCTGAGGTATTACTACATCGCCATGACCTCTCCCGAGCTCGCGGAGCCGCGGTTTCTCGCCGTGGGCTACGTGGACGATCAGCAGGGCGGGCGCTTCGACAGTGCCAGCGCTAATCAGAGGGCGGAGCCGCGGGCGGCGTGGATAGAGAAGGTGGAGGAGGAGGAGCCGGGCTACTGGGAGCGAAACACGCGAATCGCCAGGGGAAACACACAGGCTTACCGAGTGTACCTGCAGACCTTGCGCGGCTACTTCAATCAGAGCGAGGGCGGGGTCCACACCATCCAGGTTATGTTCGGCTGCGAGGTCTCCCCGGACCTCACCTTCAAGCGAGGGTTTTTCCAATACGCCTACGACGGGCAAGACTACATCGCCCTGGACAGGGAGACCTCCACATGGACGGCGGCGGTGCCCCAGGCTGTGAACACCAAGCTGGAGTGGGAGGCGGACAGGAGCATCGCTGAGAGACGGAAAGCCTATCTGGAGGAGGAGTGCGTGATGTGGCTGAAGAAGTACCTGGATATGGGGAAGGAGATGCTGAAGAGGGCAGACCCGCCCTCAGTCCGAGTGACCCGCCACACTGGCCCCAACGGGGAGGTGGCCTTGCGGTGTCGAGCCCAGAACTTCTACCCCGAGGACATCTCCCTGACTTGGCTGAGGGATGGGGAGGAGCAGCTCCAGGACACGGAGTTCATTGAGACCAGGCCTTCGGGAGACGGGACCTTCCAGAAGTGGGCAGGTGTGGACGTGACCTCGGGCCAGGAAGGGAAATATACCTGCCGAGTTCAGCACGAGGGACTGCCTGAGCCCCTCACCCTGAAGTGGGAGCCAGAGTCCTCATCTACCCGGGTCATCGTGGGGGCCATTGCTGTCCTCATCTTCATCACTGCAGTCATTGCTGGAGTTGTGATCTGGAGGAAGAACACTTCAGGTGGAAAAGGAGGGAACTATGTTCCTGCTGCAGGCAATGACAGTGCACAGGGGTCACATGTCTTCCCCACAGCCAAAGCTTGA

>MHCI_12_UH_Hap_11

ATGGAGCGTTATCTGTGCTCTTTGTTTTTGTTGGGGACCCTGGCCCTGCCCGAGACCTGGGCAGGATCTCATTCCCTGAGGTATTACTACATCGCCATGACCTCTCCCGAGCTCGCGGAGCCGCGGTTTCTCGCCGTGGGCTACGTGGACGATCAGCAGGGCGGGCGCTTCGACAGTGCCAGCGCTAATCAGAGGGCGGAGCCGCGGGCGGCGTGGATAGAGAAGGTGGAGGAGGAGGAGCCGGGCTACTGGGAGCGAAACACGCGAATCGCCAGGGGGAACACACAGGCTTACCGAGTGTACCTGCAGACCCTGCGCGGCTACTTCAACCAGAGCGAAGGCGGGGTCCACACCATCCAGGTTATGTTCGGCTGCGAGGTCTCCCCGGACCTCACCTTCAAGCGAGGGTTTTTCCAATACGCCTACGACGGGCAAGACTACATCGCCCTGGACAGGGAGACCTCCACATGGACGGCGGCGGTGCCCCAGGCTGTGAACACCAAGCTGGAGTGGGAGGCGGACAGGAGCATCGCTGAGAGACGGAAAGCCTATCTGGAGGAGGAGTGCGTGATGTGGCTGAAGAAGTACCTGGATATGGGGAAGGAGATGCTGAAGAGGGCAGACCCGCCCGCAGTCCGAGTGACCCGCCACACTGGCCCCAACGGGGAGGTGGCCTTGCGGTGTCGAGCCCAGAACTTCTACCCCGAGGACATCTCCCTGACTTGGCTGAGGGATGGGGAGGAGCAGCTCCAGGACACGGAGTTCATTGAGACCAGGCCTTCGGGAGACGGGACCTTCCAGAAGTGGGCAGGTGTGGACGTGACCTCGGGCCAGGAAGGGAAATATACCTGCCGAGTTCAGCACGAGGGACTGCCTGAGCCCCTCACCCTGAAGTGGGAGCCAGAGTCCTCATCTACCCGGGTCATCGTGGGGGCCATTGCTGTCCTCATCTTCATCACTGCAGTCATTGCTGGAGTTGTGATCTGGAGGAAGAACACTTCAGGTGGAAAAGGAGGGAACTATGTTCCTGCTGCAGGCAATGACAGTGCACAGGGGTCACATGTCTTCCCCACAGCCAAAGCTTGA

>MHCI_12_UH_Hap_12

ATGGAGCGTTATCTGTGCTCTTTGTTTTTGTTGGGGACCCTGGCCCTGCCCGAGACCTGGGCAGGATCTCATTCCCTGAGGTATTACTACATCGCCATGACCTCTCCCGAGCTCGCGGAGCCGCGGTTTCTCGCCGTGGGCTACGTGGACGATCAGCAGGGCGGGCGCTTCGACAGTGCCAGCGCTAATCAGAGGGCGGAGCCGCGGGCGGCGTGGATAGAGAAGGTGGAGGAGGAGGAGCCGGGCTACTGGGAGCGAAACACGCGAATCGCCAGGGGGAACACACAGGTTTACCGAGTGTACCTCCAGACCTTGCGCGGCTACTTCAATCAGAGCGAGGGCGGGGTCCACACCATCCAGGTTATGTTCGGCTGCGAGGTCTCCCCGGACCTCACCTTCAAGCGAGGGTTTTTCCAATACGCCTACGACGGGCAAGACTACATCGCCCTGGACAGGGAGACCTCCACATGGACGGCGGCGGTGCCCCAGGCTGTGAACACCAAGCTGGAGTGGGAGGCGGACAGGAGCATCGCTGAGAGACGGAAAGCCTATCTGGAGGAGGAGTGCGTGATGTGGCTGAAGAAGTACCTGGATATGGGGAAGGAGATGCTGAAGAGGGCAGACCCGCCCTCAGTCCGAGTGACCCGCCACACTGGCCCCAACGGGGAGGTGGCCTTGCGGTGTCGAGCCCAGAACTTCTACCCCGAGGACATCTCCCTGACTTGGCTGAGGGATGGGGAGGAGCAGCTCCAGGACACGGAGTTCATTGAGACCAGGCCTGCTGGGGACGGGACCTTCCAGAAGTGGGCAGGTGTGGACGTGACCTCGGGCCAGGAAGGGAAATATACCTGCCGAGTTCAGCACGAGGGACTGCCTGAGCCCCTCACCCTGAAGTGGGAGCCAGAGTCCTCATCTACCCGGGTCATCGTGGGGGCCATTGCTGTCCTCATCTTCATCACTGCAGTCATTGCTGGAGTTGTGATCTGGAGGAAGAACACTTCAGGTGGAAAAGGAGGGAACTATGTTCCTGCTGCAGGCAATGACAGTGCACAGGGGTCACATGTCTTCCCCACAGCCAAAGCTTGA

>MHCI_12_UH_Hap_13

ATGGAGCGTTATCTGCGTTCTCTCTTTTTGTTGGGTACCCTGGCCCTGTCGGAGACCTGGGCAGGATCTCATTCCCTGAGGTATTACTACATCGCCATGACCTCTCCCGAGCTCGCGGAGCCGCGGTTTCTCGCCGTGGGCTACGTGGACGATCAGCAGGGCGGGCGCTTCGACAGTGCCAGCGCTAATCAGAGGGCGGAGCCGCGGGCGGCGTGGATAGAGAAGGTGGAGGAGGAGGAGCCGGGCTACTGGGAGCGAAACACGCGAATCGCCAGGGGGAACACACAGGCTTACCGAGTGTACCTGCAGACCTTGCGCGGCTACTTCAATCAGAGCGAGGGCGGGGTCCACACCATCCAGGTTATGTTCGGCTGCGAGGTCTCCCCGGACCTCACCTTCAAGCGAGGGTTTTTCCAATACGCCTACGACGGGCAAGACTACATCGCCCTGGACAGGGAGACCTCCACATGGACGGCGGCGGTGCCCCAGGCTGTGAACACCAAGCTGGAGTGGGAGGCGGACAGGAGCATCGCTGAGAGACGGAAAGCCTATCTGGAGGAGGAGTGCGTGATGTGGCTGAAGAAGTACCTGGATATGGGGAAGGAGATGCTGAAGAGGGCAGACCCGCCCGCAGTCCGAGTGACCCGCCACACTGGCCCCAACGGGGAGGTGGCCTTGCGGTGTCGAGCCCAGAACTTCTACCCCGAGGACATCTCCCTGACTTGGCTGAGGGATGGGGAGGAGCAGCTCCAGGACACGGAGTTCATTGAGACCAGGCCTTCGGGAGACGGGACCTTCCAGAAGTGGGCAGGTGTGGACGTGACCTCGGGCCAGGAAGGGAAATATACCTGCCGAGTTCAGCACGAGGGACTGCCTGAGCCCCTCACCCTGAAGTGGGAGCCAGAGTCCTCATCTACCCGGGTCATCGTGGGGGCCATTGCTGTCCTCATCTTCATCACTGCAGTCATTGCTGGAGTTGTGATCTGGAGGAAGAACACTTCAGGTGGAAAAGGAGGGAACTATGTTCCTGCTGCAGGCAATGACAGTGCACAGGGGTCACATGTCTTCCCCACAGCCAAAGCTTGA

>MHCI_13_UG_Hap_2

ATGGAGCGTTATCTGTGCTCTTTGTTTTTGTTGGGGACCCTGGCCCTGCCCGAGACCTGGGCAGGCTCCCACTCCCTCAGGTATTTCGACACCGCGATGGCCTCGCCCGAGCTTGCGGAGCCGCGGTATATCTCTGTGGGCTACGTAGACGATCAGCAGTTCGTGCGCTTCGACAGCGCCAGTGCAAGTCAGAGAGAGGAGCCGCGGGCTTCGTGGATGGAGCGGATGGATCAGGAGGACCCGGACTACTGGGAGCGGAACACGCGGATCTCCAGGGGGAACGCGCAGGTTTTACTAGAGGGCCTGCAGACCCTGCGCGGCTACTTCAACCAGAGCAATGGCGGGGTCCACACCATCCAGATTATGTTCGGCTGCGAGGTCTCCCCGGACCTCACCTTCAAGCGCGGGTTTCAACAACACGCCTACGACGGTCAAGACTACATCACCCTGGACACGGAGGCCTCCAAGTGGAGGGCGGCGGTGCCCGCGGCTGTGAACACCAAGCGCAAGTGGGAGGCGGACAGGAGCATCGCCGAGGGATATAAAGCCTACGTGGAGGAGACGTGCGTGCCGTGGCTGAAGAAGTACCTAGAGATGGGGGAGGAGACGCTGAAGAGGGCAGACCCGCCCGCAGTCCGAGTGACCCACCACACTGGCCCCCACGGGGAGGTGACCCTGCGGTGCCGGGCCCAGGACTTCTACCCTGCGGACATCTCCCTGACTTGGCTGAGGGATGGGGAGGAGCAGCTCCAGGACACGGAGTTCATTGAGACCAGGCCTTCGGGAGACGGGACCTTCCAGAAGTGGGCAGGTGTGGACGTGACCTCGGGCCAGGAAGAGAAATATACCTGCCGAGTTCAGCACGAGGGACTGCCTGAGCCCCTCACCCTGAAGTGGGAGCCAGAGTCCTCATACACCCTGGTCATTGTGGGGGGCATTGCTGTCCTCCTCCTCATCACTGCAGTCATTGCTGGAGTTGTGCTCTGGAGGAAGAAGACTTCAGGTGGAAAAGGAGGGGACTATGTTCCTGCTGCAGGCAATGACAGTGCACAGGAGTCAGATGTCTCCCTCACTGACAAAGCTTGAGAGAATTGCCAAGTATGCACCTGA

>MHCI_13_UG_Hap_1

ATGGAGCGTTATCTGTGCTCTTTGTTTTTGTTGGGGACCCTGGCCCTGCCCGAGACCTGGGCAGGCTCCCACTCCCTCAGGTATTTCGACACCGCGATGGCCTCGCCCGAGCTTGCGGAGCCGCGGTATATCTCTGTGGGCTACGTAGACGATCAGCAGTTCGTGCGCTTCGACAGCGCCAGTGCAAGTCAGAGAGAGGAGCCGCGGGCTTCGTGGATGGAGCGGATGGATCAGGAGGACCCGGACTACTGGGAGCGGAACACGCGGATCTCCAGGGGGAACGCGCAGGTTTTACTAGAGGGCCTGCAGACCCTGCGCGGCTACTTCAACCAGAGCAATGGCGGGGTCCACACCATCCAGATTATGTTCGGCTGCGAGGTCTCCCCGGACCTCACCTTCAAGCGCGGGTTTCAACAACACGCCTACGACGGTCAAGACTACATCACCCTGGACACGGAGGCCTCCAAGTGGAGGGCGGCGGTGCCCGCGGCTGTGAACACCAAGCGCAAGTGGGAGGCGGACAGGAGCATCGCCGAGGGATATAAAGCCTACGTGGAGGAGACGTGCGTGCCGTGGCTGAAGAAGTACCTAGAGATGGGGGAGGAGACGCTGAAGAGGGCAGACCGGCCCGCAGTCCGAGTGACCCACCACACTGGCCCCCACGGGGAGGTGACCCTGCGGTGCCGGGCCCAGGACTTCTACCCTGCGGACATCTCCCTGACTTGGCTGAGGGATGGGGAGGAGCAGCTCCAGGACACGGAGTTCATTGAGACCAGGCCTTCGGGAGACGGGACCTTCCAGAAGTGGGCAGGTGTGGACGTGACCTCGGGCCAGGAAGAGAAATATACCTGCCGAGTTCAGCACGAGGGACTGCCTGAGCCCCTCACCCTGAAGTGGGAGCCAGAGTCCTCATACACCCTGGTCATTGTGGGGGGCATTGCTGTCCTCCTCCTCATCACTGCAGTCATTGCTGGAGTTGTGCTCTGGAGGAAGAAGACTTCAGGTGGAAAAGGAGGGGACTATGTTCCTGCTGCAGGCAATGACAGTGCACAGGAGTCAGATGTCTCCCTCACTGACAAAGCTTGAGAGAATTGCCAAGTATGCACCTGA

>MHCI_13_UG_Hap_5

ATGGAGCGTTATCTGTGCTCTTTGTTTTTGTTGGGGACCCTGGCCCTGCCCGAGACCTGGGCAGGCTCCCACTCCCTCAGGTATTTCGACACCGCGATGGCCTCGCCCGAGCTTGCGGAGCCGCGGTATATCTCTGTGGGCTACGTAGACGATCAGCAGTTCGTGCGCTTCGACAGCGCCAGTGCAAGTCAGAGAGAGGAGCCGCGGGCTTCGTGGATGGAGCGGATGGATCAGGAGGACCCGGACTACTGGGAGCGGAACACGCGGATCTCCAGGGGGAACGCGCAGGTTTTACTAGAGGGCCTGCAGACCCTGCGCGGCTACTTCAACCAGAGCAATGGCGGGGTCCACACCATCCAGATTATGTTCGGCTGCGAGGTCTCCCCGGACCTCACCTTCAAGCGCGGGTTTCAACAACACGCCTACGACGGTCAAGACTACATCACCCTGGACACGGAGGCCTCCAAGTGGAGGGCGGCGGTGCCCGCGGCTGTGAACACCAAGCGCAAGTGGGAGGCGGACAGGAGCATCGCCGAGGGATATAAAGCCTACGTGGAGGAGACGTGCGTGCCGTGGCTGAAGAAGTACCTAGAGATGGGGGAGGAGACGCTGAAGAGGGCAGACCCGCCCGCAGTCCGAGTGACCCACCACACTGGCCCCCACGGGGAGGTGACCCTGCGGTGCCGGGCCCAGGACTTCTACCCCGAGGACATCTCCCTGACTTGGCTGAGGGATGGGGAGGAGCAGCTCCAGGACACGGAGTTCATTGAGACCAGGCCTTCGGGAGACGGGACCTTCCAGAAGTGGGCAGGTGTGGACGTGACCTCGGGCCAGGAAGAGAAATATACCTGCCGAGTTCAGCACGAGGGACTGCCTGAGCCCCTCACCCTGAAGTGGGAGCCAGAGTCCTCATACACCCTGGTCATTGTGGGGGGCATTGCTGTCCTCCTCCTCATCACTGCAGTCATTGCTGGAGTTGTGCTCTGGAGGAAGAAGACTTCAGGTGGAAAAGGAGGGGACTATGTTCCTGCTGCAGGCAATGACAGTGCACAGGAGTCAGATGTCTCCCTCACTGACAAAGCTTGAGAGAATTGCCAAGTATGCACCTGA

>MHCI_13_UG_Hap_6

ATGGAGCGTTATCTGTGCTCTTTGTTTTTGTTGGGGACCCTGGCCCTGCCCGAGACCTGGGCAGGCTCCCACTCCCTCAGGTATTTCGACACCGCGATGGCCTCGCCCGAGCTTGCGGAGCCGCGGTATATCTCTGTGGGCTACGTAGACGATCAGCAGTTCGTGCGCTTCGACAGCGCCAGTGCAAGTCAGAGAGAGGAGCCGCGGGCTTCGTGGATGGAGCGGATGGATCAGGAGGACCCGGACTACTGGGAGCGGAACACGCGGATCTCCAGGGGGAACGCGCAGGTTTTACTAGAGGGCCTGCAGACCCTGCGCGGCTACTTCAACCAGAGCAATGGCGGGGTCCACACCATCCAGATTATGTTCGGCTGCGAGGTCTCCCCGGACCTCACCTTCAAGCGCGGGTTTCAACAACACGCCTACGACGGTCAAGACTACATCACCCTGGACACGGAGGCCTCCAAGTGGAGGGCGGCGGTGCCCGCGGCTGTGAACACCAAGCGCAAGTGGGAGGCGGACAGGAGCATCGCCGAGGGATATAAAGCCTACGTGGAGGAGACGTGCGTGCCGTGGCTGAAGAAGTACCTAGAGATGGGGGAGGAGACGCTGAAGAGGGCAGACCCGCCCGCAGTCCGAGTGACCCACCACACTGGCCCCCACGGGGAGGTGACCCTGCGGTGCCGGGCCCAGGACTTCTACCCTGCGGACATCTCCCTGACTTGGCTGAGGGATGGGGAGGAGCAGCTCCAGGACACGGAGTTCATTGAGACCAGGCCTTCGGGAGACGGGACCTTCCAGAAGTGGGCAGGTGTGGACGTGACCTCGGGCCAGGAAGAGAAATATACCTGCCGAGTTCAGCACGAGGGACTGCCTGAGCCCCTCACCCTGAAGTGGGAGCCAGAGTCCTCATACACCCTGGTCATTGTGGGGGGCATTGCTGTCCTCCTCCTCATCACTGCAGTCATTGCTGGAGTTGTGCTCTGGAGGAAGAAGACTTCAGGTGGAAAAGGAGGGGACTATGTTCCTGCTGCAGGCAATGACAGTGCACAGGAGTCAGATGTCTCCCTCACTGACAAAGCTTCAGAGAATTACCCGGTACGCACCTGA

>MHCI_13_UG_Hap_7

ATGGAGCGTTATCTGTGCTCTTTGTTTTTGTTGGGGACCCTGGCCCTGCCCGAGACCTGGGCAGGCTCCCACTCCCTCAGGTATTTCGACACCGCGATGGCCTCGCCCGAGCTTGCGGAGCCGCGGTATATCTCTGTGGGCTACGTAGACGATCAGCAGTTCGTGCGCTTCGACAGCGCCAGTGCAAGTCAGAGAGAGGAGCCGCGGGCTTCGTGGATGGAGCGGATGGATCAGGAGGACCCGGACTACTGGGAGCGGAACACGCGGATCTCCAGGGGGAACGCGCAGGTTTTACTAGAGGGCCTGCAGACCCTGCGCGGCTACTTCAACCAGAGCAATGGCGGGGTCCACACCATCCAGATTATGTTCGGCTGCGAGGTCTCCCCGGACCTCACCTTCAAGCGCGGGTTTCAACAACACGCCTACGACGGTCAAGACTACATCACCCTGGACACGGAGGCCTCCAAGTGGAGGGCGGCGGTGCCCGCGGCTGTGAACACCAAGCGCAAGTGGGAGGCGGACAGGAGCATCGCCGAGGGATATAAAGCCTACGTGGAGGAGACGTGCGTGCCGTGGCTGAAGAAGTACCTAGAGATGGGGGAGGAGACGCTGAAGAGGGCAGACCCGCCCGCAGTCCGAGTGACCCGCCGCGCTGGCCCCCACGGGGAGGTGACCCTGCGGTGCCGGGCCCAGGACTTCTACCCTGCGGACATCTCCCTGACTTGGCTGAGGGATGGGGAGGAGCAGCTCCAGGACACGGAGTTCATTGAGACCAGGCCTTCGGGAGACGGGACCTTCCAGAAGTGGGCAGGTGTGGACGTGACCTCGGGCCAGGAAGAGAAATATACCTGCCGAGTTCAGCACGAGGGACTGCCTGAGCCCCTCACCCTGAAGTGGGAGCCAGAGTCCTCATACACCCTGGTCATTGTGGGGGGCATTGCTGTCCTCCTCCTCATCACTGCAGTCATTGCTGGAGTTGTGCTCTGGAGGAAGAAGACTTCAGGTGGAAAAGGAGGGGACTATGTTCCTGCTGCAGGCAATGACAGTGCACAGGAGTCAGATGTCTCCCTCACTGACAAAGCTTGAGAGAATTGCCAAGTATGCACCTGA

>MHCI_13_UG_Hap_3

ATGGAGCGTTATCTGTGCTCTTTGTTTTTGTTGGGGACCCTGGCCCTGCCCGAGACCTGGGCAGGCTCCCACTCCCTCAGGTATTTCGACACCGCGATGGCCTCGCCCGAGCTTGCGGAGCCGCGGTATATCTCTGTGGGCTACGTAGACGATCAGCAGTTCGTGCGCTTCGACAGCGCCAGTGCAAGTCAGAGAGAGGAGCCGCGGGCTTCGTGGATGGAGCGGATGGATCAGGAGGACCCGGACTACTGGGAGCGGAACACGCGGATCTCCAGGGGGAACGCGCAGGTTTTACTAGAGGGCCTGCAGACCCTGCGCGGCTACTTCAACCAGAGCAATGGCGGGGTCCACACCATCCAGATTATGTTCGGCTGCGAGGTCTCCCCGGACCTCACCTTCAAGCGCGGGTTTCAACAACACGCCTACGACGGTCAAGACTACATCACCCTGGACACGGAGGCCTCCAAGTGGAGGGCGGCGGTGCCCGCGGCTGTGAACACCAAGCGCAAGTGGGAGGCGGACAGGAGCATCGCCGAGGGATATAAAGCCTACGTGGAGGAGACGTGCGTGCCGTGGCTGAAGAAGTACCTAGAGATGGGGGAGGAGACGCTGAAGAGGGCAGACCGGCCCGCAGTCCGAGTGACCCACCACACTGGCCCCCACGGGGAGGTGACCCTGCGGTGCCGGGCCCAGGACTTCTACCCTGCGGACATCTCCCTGACTTGGCTGAGGGATGGGGAGGAGCAGCTCCAGGACACGGAGTTCATTGAGACCAGGCCTTCGGGAGACGGGACCTTCCAGAAGTGGGCAGGTGTGGACGTGACCTCGGGCCAGGAAGAGAAATATACCTGCCGAGTTCAGCACGAGGGACTGCCTGAGCCCCTCACCCTGAAGTGGGAGCCAGAGTCCTCATACACCCTGGTCATTGTGGGGGGCATTGCTGTCCTCCTCCTCATCACTGCAGTCATTGCTGGAGTTGTGCTCTGGAGGAAGAAGACTTCAGGTGGAAAAGGAGGGGACTATGTTCCTGCTGCAGGCAATGACAGTGCACAGGAGTCAGATGTCTCCCTCACTGACAAAGCTTGAGAGAATTGCCCAGTATGCACCTGA

>MHCI_13_UG_Hap_4

ATGGAGCGTTATCTGTGCTCTTTGTTTTTGTTGGGGACCCTGGCCCTGCCCGAGACCTGGGCAGGCTCCCACTCCCTCAGGTATTTCGACACCGCGATGGCCTCGCCCGAGCTTGCGGAGCCGCGGTATATCTCTGTGGGCTACGTAGACGATCAGCAGTTCGTGCGCTTCGACAGCGCCAGTGCAAGTCAGAGAGAGGAGCCGCGGGCTTCGTGGATGGAGCGGATGGATCAGGAGGACCCGGACTACTGGGAGCGGAACACGCGGATCTCCAGGGGGAACGCGCAGGTTTTACTAGAGGGCCTGCAGACCCTGCGCGGCTACTTCAACCAGAGCAATGGCGGGGTCCACACCATCCAGATTATGTTCGGCTGCGAGGTCTCCCCGGACCTCACCTTCAAGCGCGGGTTTCAACAACACGCCTACGACGGTCAAGACTACATCACCCTGGACACGGAGGCCTCCAAGTGGAGGGCGGCGGTGCCCGCGGCTGTGAACACCAAGCGCAAGTGGGAGGCGGACAGGAGCATCGCCGAGGGATATAAAGCCTACGTGGAGGAGACGTGCGTGCCGTGGCTGAAGAAGTACCTAGAGATGGGGGAGGAGACGCTGAAGAGGGCAGACCCGCCCGCAGTCCGAGTGACCCGCCGCGCTGGCCCCCACGGGGAGGTGACCCTGCGGTGCCGGGCCCAGGACTTCTACCCCGAGGACATCTCCCTGACTTGGCTGAGGGATGGGGAGGAGCAGCTCCAGGACACGGAGTTCATTGAGACCAGGCCTGCGGGAGACGGGACTTTCCAGAAGTGGGCAGGTGTGGACGTGACCTCGGGCCAGGAAGAGAAATATACCTGCCGAGTTCAGCACGAGGGACTGCCTGAGCCCCTCACCCTGAAGTGGGAGCCAGAGTCCTCATACACCCTGGTCATTGTGGGGGGCATTGCTGTCCTCATCGTCATCACTGCAGTCATTGCTGGAGTTGTGCTCTGGAGGAAGAAGACTTCAGGTGGAAAAGGAGGGGACTATGTTCCTGCTGCAGGCAATGACAGTGCACAGGAGTCAGATGTCTCCCTCACTGACAAAGCTTCAGAGAATTACCCGGTACGCACCTGA

>MHCI_15_UJ_Hap_2

ATGGAGCTTAATGTGCCCTCTCTCTTTTTGTTGGGGACCCTGGCCCTGACGGAGATCTGGGCAGGCTGTCACTCCCTGAGGTACTTCGACGCCGCCATGGCCTCGCACGAGCTCGCGGAGCCCCGGTTCCTCGCCGTGGGCTACGTGGACGATCAGCAGTTCGTGCGCTTCGACAGCCACAGCGCGAGTCCGAGGGAGGAGCCGCGGGCGCCGTGGATGGAGCAGATGGACCCGGGCTACTGGGAGCGGAACACGCGGGTCAATAGGGAGAATGCACAGAGTTACCGAGTGGGCCTGCAGAACCTCCGCGGCTACTTCAACCAGAGCGAGGGCGGGGTCCACACCTACCAGAGCATTTGCGGCTGCGAGGTCTCCCCCGATCTCACCTTCAAGCGCGGGTTTTACCAATTTGCCTACGACGGCCAAGACTACATCGCCCTGGACACGGAGACCTCCACATGGATCGCGTTGGTGCCCCAGGCCGTGAACACCAAGCTCAAGTGGGAGGTGGACAGCAGCCTAGTTGAGAGAGAGAAAGCCTATCTGAAGGAGAAGTGCGTGCAGTGGCTGAAGAAGTACCTGGAGATCGGGCAGAAGACTCTGAAGAGGGCAGACCCGCCCAATGCCCGAGTGACCCGCCACACTGCCCCCTACGGGGAGGTGACCCTGCGGTGCCGGGCCCAAGACTTCTACCCCTCGGACATCTCCCTGACTTGGCTGAGGGATGGGGAGGAACAGCTGCAGGACACAGAGTTCATTGAGACCAGGCCTGCAGGAGATGGAACCTTCCAGAAGTGGGCAGGTGTGGATGTGACCTCGGGCCAAGAAGAGAAATATACCTGCCGAGTTCAGCACGAGGGACTGCCTGAGCCCCTCACCCTGAAGTGGGCCAGAATCCTCATCCACCTGGCTCATTGTGGGAGGCCTTGCTGTCCTCCACCTCCTCCTCACTGCAGCCATTGCTGA

>MHCI_15_UJ_Hap_1

ATGGAGCTTAATGTGCCCTCTCTCTTTTTGTTGGGGACCCTGGCCCTGACGGAGATCTGGGCAGGCTGTCACTCCCTGAGGTACTTCGACGCCGCCATGGCCTCGCACGAGCTCGCGGAGCCCCGGTTCCTCGCCGTGGGCTACGTGGACGATCAGCAGTTCGTGCGCTTCGACAGCCACAGCGCGAGTCCGAGGGAGGAGCCGCGGGCGCCGTGGATGGAGCAGATGGACCCGGGCTACTGGGAGCGGAACACGCGGGTCAATAGGGAGAATGCACAGAGTTACCGAGTGGGCCTGCAGAACCTCCGCGGCTACTTCAACCAGAGCGAGGGCGGGGTCCACACCTACCAGAGCATTTGCGGCTGCGAGGTCTCCCCCGATCTCACCTTCAAGCGCGGGTTTTACCAATTTGCCTACGACGGCCAAGACTACATCGCCCTGGACACGGAGACCTCCACATGGATCGCGTTGGTGCCCCAGGCCGTGAACACCAAGCTCAAGTGGGAGGTGGACAGCAGCCTAGTTGAGAGAGAGAAAGCCTATCTGAAGGAGAAGTGCGTGCAGTGGCTGAAGAAGTACCTGGAGATCGGGCAGAAGACTCTGAAGAGGGCAGACCCGCCCAATGCCCGAGTGACCCGCCACACTGCCCCCTACGGGGAGGTGACCCTGCGGTGCCGGGCCCAAGACTTCTACCCCTCGGACATCTCCCTGACTTGGCTGAGGGATGGGGAGGAACAGCTGCAGGACACAGAGTTCATTGAGACCAGGCCTGCAGGAGATGGAACCTTCCAGAAGTGGGCAGGTGTGGATGTGACCTCGGGCCAAGAAGAGAAATATACCTGCCGAGTTCAGCACGAGGGACTGCCTGAGCCCCTCACCCTGAAGTGGGCCAGAATCCTCATCCACCTGGCTCATTGTGGGAGGCCTTGCTGTCCTCCACCTCCTCCTCACTGCAGTCATTGCTGA

>MHCI_19_UB_Hap_1

ATGGAGCCTTATGTGCGTTGTCTCTTTTTGTTGGGGACCTTGGCCCTGAAGGAGACCTGGGCAGGCTCTCACTCCATGAGGTACTTCGATGCCGCCATGGCCTCCCCTGAGCTCGAGGAGCCGCGGTTCCTCACCGTGGGCTACGTGGACGATCAGCAGTTCATGCGCTTCGACAGCGCCAGCGCGAGTCCGAGGGAGGAGCCGCGGGCGGCGTGGATGGAGCGGGTGGAGCAGGAGGAGCCGGGCTACTGGGAGCAGGAGACGGGGAAGCATAAGGCGAACGCACAGACTACCCGAGTGAACCTGCAGACTGCTCTCGGCTACTTCAACCAGAGCGAGGGCGGGGTCCACACCATCCAGCGCATGTACGGCTGCGAGGTCTCCCCCGAGCTCACCTTCAAGCGCGGGTTTGAACAACACGCCTACGACGGTCAAGACTACATTGCCCTGGACATGGAGACCTCCACGTGGACGGCGGCGGTGCCGCAGGCTCTGAACACCAAGCGCAAGTGGGAGGCAGAAAAGAGCATCGCGGAGGGAGTTAAAGCCTACCTGGAGGAGACGTGCGTGCTGTGGCTGAAGAAGTACCTGGAGATGGGGAAGGAGACGCTGAAGAGGACAGACCCGCCCTCCGCCCAAGTGACCCGCCACACTGCCCCCCAAGGGGAGGTGACCCTGCGGTGCCGGGCCCAGGATTTCTACCCCGAGGACATCTCCCTGACGTGGCTGAGGAATGGGGAGGAGCAGCTCCAGGACACGGAGTTCATTGAGACCAGGCCCGCGGGGGACGGGACCTTCCAGAAATGGGCAGGTGTGGATGTGACCTCGGGCCAGGAAGGGAAATATACCTGCCGAGTTCAGCACGAAGGACTGCCTGAGCCCCTCACCCTGAAGTGGGAGTCAGAGTCCTCCTCCACCTGGTACATTGTGGGGGTCCTTCTCCTCACCATTGCAGTCGTCGCTGGATTTGTGATCTGGAGAAACACTTCAGGTGGAAAAGGAGGGGACTATGTTCAGGCTGCAGGCAATGACAGTGCACAGGGGTCAGATGTCTCTGTCACAGCCAAAGCTTGA

>MHCI_19_UB_Hap_3

ATGGAGCCTTATGTGCGTTGTCTCTTTTTGTTGGGGACCTTGGCCCTGAAGGAGACCTGGGCAGGCTCTCACTCCATGAGGTACTTCGATGCCGCCATGGCCTCCCCTGAGCTCGAGGAGCCGCGGTTCCTCACCGTGGGCTACGTGGACGATCAGCAGTTCATGCGCTTCGACAGCGCCAGCGCGAGTCCGAGGGAGGAGCCGCGGGCGGCGTGGATGGAGCGGGTGGAGCAGGAGGAGCCGGGCTACTGGGAGCAGGAGACGGGCAAGCATAAGGCGAACGCACAGACTTACCGAGTGAACCTGCAGACTGCTCTCGGCTACTTCAACCAGAGCGAGGGCGGGGTCCACACCATCCAGCGCATGTACGGCTGCGAGGTCTCCCCCGAGCTCACCTTCAAGCGCGGGTTTGAACAACACGCCTACGACGGTCAAGACTACATTGCCCTGGACATGGAGACCTCCACGTGGACGGCGGCGGTGCCGCAGGCTCTGAACACCAAGCGCAAGTGGGAGGCAGAAAAGAGCATCGCGGAGGGAGTTAAAGCCTACCTGGAGGAGACGTGCGTGCTGTGGCTGAAGAAGTACCTGGAGATGGGGAAGGAGACGCTGAAGAGGACAGACCCGCCCTCCGCCCAAGTGACCCGCCACACTGCCCCCCAAGGGGAGGTGACCCTGCGGTGCCGGGCCCAGGATTTCTACCCCGAGGACATCTCCCTGACGTGGCTGAGGAATGGGGAGGAGCAGCTCCAGGACACGGAGTTCATTGAGACCAGGCCCGCGGGGGACGGGACCTTCCAGAAATGGGCAGGTGTGGATGTGACCTCGGGCCAGGAAGGGAAATATACCTGCCGAGTTCAGCACGAAGGACTGCCTGAGCCCCTCACCCTGAAGTGGGAGCCAGAGTCCTCATACACCTGGTACATTGTGGGGGTCCTCCTCCTCACCATTGCAGTCGTCGCTGGATTTGTGATCTGGAGAAACACTTCAGGTGGAAAAGGAGGGGACTATGTTCAGGCTGCAGGCAATGACAGTGCACAGGGGTCAGATGTCTCTCTCACAGCCAAAGCTTGA

>MHCI_19_UB_Hap_4

ATGGAGCCTTATGTGCGTTGTCTCTTTTTGTTGGGGACCTTGGCCCTGAAGGAGACCTGGGCAGGCTCTCACTCCATGAGGTACTTCGATGCCGCCATGGCCTCCCCTGAGCTCGAGGAGCCGCGGTTCCTCACCGTGGGCTACGTGGACGATCAGCAGTTCATGCGCTTCGACAGCGCCAGCGCGAGTCCGAGGGAGGAGCCGCGGGCGGCGTGGATGGAGCGGGTGGAGCAGGAGGAGCCGGGCTACTGGGAGCAGGAGACGGGGAAGCATAAGGCGAACGCACAGACTACCCGAGTGAACCTGCAGACTGCTCTCGGCTACTTCAACCAGAGCGAGGGCGGGGTCCACACCATCCAGCGCATGTACGGCTGCGAGGTCTCCCCCGAGCTCACCTTCAAGCGCGGGTTTGAACAACACGCCTACGACGGTCAAGACTACATTGCCCTGGACATGGAGACCTCCACGTGGACGGCGGCGGTGCCGCAGGCTCTGAACACCAAGCGCAAGTGGGAGGCAGAAAAGAGCATCGCGGAGGGAGTTAAAGCCTACCTGGAGGAGACGTGCGTGCTGTGGCTGAAGAAGTACCTGGAGATGGGGAAGGAGACGCTGAAGAGGACAGACCCGCCCTCCGCCCAAGTGACCCGCCACACTGCCCCCCAAGGGGAGGTGACCCTGCGGTGCCGGGCCCAGGATTTCTACCCCGAGGACATCTCCCTGACGTGGCTGAGGAATGGGGAGGAGCAGCTCCAGGACACGGAGTTCATTGAGACCAGGCCCGCGGGGGACGGGACCTTCCAGAAATGGGCAGGTGTGGATGTGACCTCGGGCCAGGAAGGGAAATATACCTGCCGAGTTCAGCACGAAGGACTGCCTGAGCCCCTCACCCTGAAGTGGGAGCCAGAGTCCTCATACACCTGGTACATTGTGGGGGTCCTCCTCCTCACCATTGCAGTCGTCGCTGGATTTGTGATCTGGAGAAACACTTCAGGTGGAAAAGGAGGGGACTATGTTCAGGCTGCAGGCAATGACAGTGCACAGGGGTCAGATGTCTCTCTCACAGCCAAAGCTTGA

>MHCI_19_UB_Hap_2

ATGGAGCCTTATGTGCGTTGTCTCTTTTTGTTGGGGACCTTGGCCCTGAAGGAGACCTGGGCAGGCTCTCACTCCATGAGGTACTTCGATGCCGCCATGGCCTCCCCTGAGCTCGAGGAGCCGCGGTTCCTCACCGTGGGCTACGTGGACGATCAGCAGTTCATGCGCTTCGACAGCGCCAGCGCGAGTCCGAGGGAGGAGCCGCGGGCGGCGTGGATGGAGCGGGTGGAGCAGGAGGAGCCGGGCTACTGGGAGCAGGAGACGGGGAAGCATAAGGCGAACGCACAGACTACCCGAGTGAACCTGCAGACTGCTCTCGGCTACTTCAACCAGAGCGAGGGCGGGGTCCATACCATCCAGCGCATGTACGGCTGCGAGGTCTCCCCCGAGCTCACCTTCAAGCGCGGGTTTGAACAACACGCCTACGACGGTCAAGACTACATTGCCCTGGACATGGAGACCTCCACGTGGACGGCGGCGGTGCCGCAGGCTCTGAACACCAAGCGCAAGTGGGAGGCAGAAAAGAGCATCGCGGAGGGAGTTAAAGCCTACCTGGAGGAGACGTGCGTGCTGTGGCTGAAGAAGTACCTGGAGATGGGGAAGGAGACGCTGAAGAGGACAGACCCGCCCTCCGCCCAAGTGACTCGCCACACTGCCCCCCAAGGGGAGGTGACCCTGCGGTGCCGGGCCCAGGATTTCTACCCCGAGGACATCTCCCTGACGTGGCTGAGGAATGGGGAGGAGCAGCTCCAGGACACGGAGTTCATTGAGACCAGGCCCGCGGGGGACGGGACCTTCCAGAAATGGGCAGGTGTGGATGTGACCTCGGGCCAGGAAGGGAAATATACCTGCCGAGTTCAGCACGAAGGACTGCCTGAGCCCCTCACCCTGAAGTGGGAGTCAGAGTCCTCCTCCACCTGGTACATTGTGGGGGTCCTTCTCCTCACCATTGCAGTCGTCGCTGGATTTGTGATCTGGAGAAACACTTCAGGTGGAAAAGGAGGGGACTATGTTCAGGCTGCAGGCAATGACAGTGCACAGGGGTCAGATGTCTCTGTCACAGCCAAAGCTTGA

>MHCI_19_UB_Hap_5

ATGGAGCCTTATGTGCGTTGTCTCTTTTTGTTGGGGACCTTGGCCCTGAAGGAGACCTGGGCAGGCTCTCACTCCATGAGGTACTTCGATGCCGCCATGGCCTCCCCTGAGCTCGAGGAGCCGCGGTTCCTCACCGTGGGCTACGTGGACGATCAGCAGTTCATGCGCTTCGACAGCGCCAGCGCGAGTCCGAGGGAGGAGCCGCGGGCGGCGTGGATGGAGCGGGTGGAGCAGGAGGAGCCGGGCTACTGGGAGCAGGAGACGGGCAAGCATAAGGCGAACGCACAGACTTACCGAGTGAACCTGCAGACTGCTCTCGGCTACTTCAACCAGAGCGAGGGCGGGGTCCACACCATCCAGCGCATGTACGGCTGCGAGGTCTCCCCCGAGCTCACCTTCAAGCGCGGGTTTGAACAACACGCCTACGACGGTCAAGACTACATTGCCCTGGACATGGAGACCTCCACGTGGACGGCGGCGGTGCCGCAGGCTCTGAACACCAAGCGCAAGTGGGAGGCAGAAAAGAGCATCGCGGAGGGAGTTAAAGCCTACCTGGAGGAGACGTGCGTGCTGTGGCTGAAGAAGTACCTGGAGATGGGGAAGGAGACGCTGAAGAGGACAGACCCGCCCTCCGCCCAAGTGACCCGCCACACTGCCCCCCAAGGGGAGGTGACCCTGCGGTGCCGGGCCCAGGATTTCTACCCCGAGGACATCTCCCTGACGTGGCTGAGGAATGGGGAGGAGCAGCTCCAGGACACGGAGTTCATTGAGACCAGGCCCGCGGGGGACGGGACCTTCCAGAAATGGGCAGGTGTGGATGTGACCTCGGGCCAGGAAGGGAAATATACCTGCCGAGTTCAGCACGAAGGACTGCCTGAGCCCCTCACCCTGAAGTGGGAGTCAGAGTCCTCCTCCACCTGGTACATTGTGGGGGTCCTTCTCCTCACCATTGCAGTCGTCGCTGGATTTGTGATCTGGAGAAACACTTCAGGTGGAAAAGGAGGGGACTATGTTCAGGCTGCAGGCAATGACAGTGCACAGGGGTCAGATGTCTCTGTCACAGCCAAAGCTTGA

>MHCI_19_UB_Hap_6

ATGGAGCCTTATGTGCGTTGTCTCTTTTTGTTGGGGACCTTGGCCCTGAAGGAGACCTGGGCAGGCTCTCACTCCATGAGGTACTTCGATGCCGCCATGGCCTCCCCTGAGCTCGAGGAGCCGCGGTTCCTCACCGTGGGCTACGTGGACGATCAGCAGTTCATGCGCTTCGACAGCGCCAGCGCGAGTCCGAGGGAGGAGCCGCGGGCGGCGTGGATGGAGCGGGTGGAGCAGGAGGAGCCGGGCTACTGGGAGCAGGAGACGGGGAAGCATAAGGCGAACGCACAGACTTACCGAGTGAACCTGCAGACTGCTCTCGGCTACTTCAACCAGAGCGAGGGCGGGGTCCACACCATCCAGCGCATGTACGGCTGCGAGGTCTCCCCCGAGCTCACCTTCAAGCGCGGGTTTGAACAACACGCCTACGACGGTCAAGACTACATTGCCCTGGACATGGAGACCTCCACGTGGACGGCGGCGGTGCCGCAGGCTCTGAACACCAAGCGCAAGTGGGAGGCAGAAAAGAGCATCGCGGAGGGAGTTAAAGCCTACCTGGAGGAGACGTGCGTGCTGTGGCTGAAGAAGTACCTGGAGATGGGGAAGGAGACGCTGAAGAGGACAGACCCGCCCTCCGCCCAAGTGACCCGCCACACTGCCCCCCAAGGGGAGGTGACCCTGCGGTGCCGGGCCCAGGATTTCTACCCCGAGGACATCTCCCTGACGTGGCTGAGGAATGGGGAGGAGCAGCTCCAGGACACGGAGTTCATTGAGACCAGGCCCGCGGGGGACGGGACCTTCCAGAAATGGGCAGGTGTGGATGTGACCTCGGGCCAGGAAGGGAAATATACCTGCCGAGTTCAGCACGAAGGACTGCCTGAGCCCCTCACCCTGAAGTGGGAGCCAGAGTCCTCATACACCTGGTACATTGTGGGGGTCCTCCTCCTCACCATTGCAGTCGTCGCTGGATTTGTGATCTGGAGAAACACTTCAGGTGGAAAAGGAGGGGACTATGTTCAGGCTGCAGGCAATGACAGTGCACAGGGGTCAGATGTCTCTCTCACAGCCAAAGCTTGA

>MHCI_19_UB_Hap_7

ATGGAGCCTTATGTGCGTTGTCTCTTTTTGTTGGGGACCTTGGCCCTGAAGGAGACCTGGGCAGGCTCTCACTCCATGAGGTACTTCGATGCCGCCATGGCCTCCCCTGAGCTCGAGGAGCCGCGGTTCCTCACCGTGGGCTACGTGGACGATCAGCAGTTCATGCGCTTCGACAGCGCCAGCGCGAGTCCGAGGGAGGAGCCGCGGGCGGCGTGGATGGAGCGGGTGGAGCAGGAGGAGCCGGGCTACTGGGAGCAGGAGACGGGGAAGCATAAGGCGAACGCACAGACTACCCGAGTGAACCTGCAGACTGCTCTCGGCTACTTCAACCAGAGCGAGGGCGGGGTCCACACCATCCAGCGCATGTACGGCTGCGAGGTCTCCCCCGAGCTCACCTTCAAGCGCGGGTTTGAACAACACGCCTACGACGGTCAAGACTACATTGCCCTGGACATGGAGACCTCCACGTGGACGGCGGCGGTGCCGCAGGCTCTGAACACCAAGCGCAAGTGGGAGGCAGAAAAGAGCATCGCGGAGGGAGTTAAAGCCTACCTGGAGGAGACGTGCGTGCTGTGGCTGAAGAAGTACCTGGAGATGGGGAAGGAGACGCTGAAGAGGACAGACCCGCCCTCCGCCCAAGTGACCCGCCACACTGCCCCCCAAGGGGAGGTGACCCTGCGGTGCCGGGCCCAGGATTTCTACCCCGAGGACATCTCCCTGACGTGGCTGAGGAATGGGGAGGAGCAGCTCCAGGACACGGAGTTCATTGAGACCAGGCCCGCGGGGGACGGGACCTTCCAGAAATGGGCAGGTGTGGATGTGACCTCGGGCCAGGAAGGGAAATATACCTGCCGAGTTCAGCACGAAGGACTGCCTGAGCCCCTCACCCTGAAGTGGGAGCCAGAGTCCTCCTCCACCTGGTACATTGTGGGGGTCCTTCTCCTCACCATTGCAGTCGTCGCTGGATTTGTGATCTGGAGAAACACTTCAGGTGGAAAAGGAGGGGACTATGTTCAGGCTGCAGGCAATGACAGTGCACAGGGGTCAGATGTCTCTCTCACAGCCAAAGCTTGA

>MHCI_2_UD_Hap_1

ATGAACCGTTATGTGAGCTCTCTCTTTTTGTTAGCGACCCTGGCCTTGCCAGAAACCTGGGCAGGCTCTCACTCCCTGAAGTATTTCTCCGCCGCCGTGTCTTGGCCCGAACTCGCAGAACCACGCTTCTTCGCAGTGGGCTACGTGGACGATTTGCAGTTTGCGCGCTTCGATAGCGACAGCGAGAGCCAGAGGGAGGAGCCGCGGGCGCCATGGATGGATCAAGTAGACCAAGTGGACCCAGACTACTGGGACCGGAACACGCGATTCCACAAAGCGGCGGCCCAGAGTTTCCGAATTCGACTTCAGACCCTGCGAAGCTCCTACAACCAGAGCGAGGGCGGGGTCCACACCTTTCAGCACCTGTGCGGCTGCGAGGTCTCCCCAGAACTCACCTTTAAGCGCGGGTTTTACCAATTCGCTTATGACGGGCACGACTACCTGGCCCTGGACACGGAGACCTACACGTGGACTGCGTCAGTGCCCCAGGCTCTGAACTCCAAGCTTGAGTTGGAGGCGGACAGCAGCATCTCGAAGGAGAGAAAAGCGTATCTAGAAGAAACGTGCGTGCAGTGGGTGAAAAAGTACCTAGAAATCGGGAAGGAGTCTTTGAAGAGGACAGACCCGCCTTCTGTCCAAGTGACCCACCACACTGCCCCCAATGGGGAAGTGATCCTGCGGTGCCGGGCCCAGGACTTCTACCCTGCCGACATCTCCCTGACTTGGCTGAGGGATGGGGAGGAACAACTCCAGGACACAGAGTTCATTGAGACCAGGCCTGCGGGAGATGGGACCTTCCAGAAGTGGGCATCTGTGGGCGTGACCTCGGGCCAGGAAGGGAAATATGCCTGCCGAGTTCAGCATGAGGGACTGCCTGAGCCCCTCACCCTGAAGTGGGAGCCACAGTCGTCCTCCACCTGGTACATTGTGGGGGGCATTGCTGTCCTCACCATTGGAGTCATCGCTGGATTTGTGATCTGGAGGGAGACTTCAGGTGGAAATAGAGGGCACCGTATTCAGCCTCCAGTTTGA

>MHCI_2_UD_Hap_2

ATGAACCGTTATGTGAGCTCTCTCTTTTTGTTAGCGACCCTGGCCTTGCCAGAAACCTGGGCAGGCTCTCACTCCCTGAAGTATTTCTCCGCCGCCGTGTCTTGGCCCGAACTCGCAGAACCACGCTTCTTCGCAGTGGGCTACGTGGACGATTTGCAGTTTGCGCGCTTCGATAGCGACAGCGAGAGCCAGAGGGAGGAGCCGCGGGCGCCATGGATGGATCAAGTAGACCAAGTGGACCCAGACTACTGGGACCGGAACACGCGATTCCACAAAGCGGCGGCCCAGAGTTTCCGAATTCGACTTCAGACCCTGCGCAGCTCCTACAACCAGAGCGAGGGCGGGGTCCACACCTTTCAGCACCTGTGCGGCTGCGAGGTCTCCCCAGAACTCACCTTTAAGCGCGGGTTTTACCAATTCGCTTATGACGGGCACGACTACCTGGCCCTGGACACGGAGACCTACACGTGGACTGCGTCAGTGCCCCAGGCTCTGAACTCCAAGCTTGAGTTGGAGGCGGACAGCAGCATCTCGAAGGAGAGAAAAGCGTATCTAGAAGAAACGTGCGTGCAGTGGGTGAAAAAGTACCTAGAAATCGGGAAGGAGTCTTTGAAGAGGACAGACCCGCCTTCTGTCCAAGTGACCCACCACACTGCCCCCAATGGGGAAGTGATCCTGCGGTGCCGGGCCCAGGACTTCTACCCTGCCGACATCTCCCTGACTTGGCTGAGGGATGGGGAGGAACAACTCCAGGACACAGAGTTCATTGAGACCAGGCCTGCGGGAGATGGGACCTTCCAGAAGTGGGCATCTGTGGGCGTGACCTCGGGCCAGGAAGGGAAATATGCCTGCCGAGTTCAGCATGAGGGACTGCCTGAGCCCCTCACCCTGAAGTGGGAGCCACAGTCGTCCTCCACCTGGTACATTGTGGGGGGCATTGCTGTCCTCACCATTGGAGTCATCGCTGGATTTGTGATCTGGAGGGAGACTTCAGGTGGAAATAGAGGGCACCGTATTCAGCCTCCAGTTTGA

>MHCI_5_UK_Hap_2

ATGGGACCTGATATACTCTCTCTCTTTTTGTTGGGGACTCTGGCCTTGACCAAGACCTGGGCAGGATTTCACTCCCTGAAGTATTTCCAAACTTCCATAGCTCTGCCTGGCCTCGAAAAGCCGAAGTTCATTTCCGCAGGCTACGTGAACGATCGACAATTCGTACGCTTTGACAGCGACAGCTCGAGTCAGAGGGAGGAGCCCTTGGTGCCGTGGATGGACCAGATGGGTCAGGGGTACTGGGAGAGGAACTCACGTGTCGTCAGGGAGACCGCGCACACTTTCGAAGTAGGCCTGCAGAATCTGCAGGTTTATTACAATCAGAGCGAGGGAGAAGTTCACATCTATCAGCGCCTGGTCGGCTGCGAGACGTATTCCAACGGGACCTTTAGGCGTGGGTTTGAGCAATTCGCCTACGATGGGCAAGACTACATCTCTCCGGATCAGAAGACGCTGAGTTGGACGGCTTTGGTGCCCCCGGCTCTGAACACCAAGTACAAGTGGGAGGAGGATCGCAGCATGGCCAAGAGACAGAAAGTTTATCTGGAGGAGAAGTGTGTTGAGTGGCTGCACAAGTACCTGGAGTTGGGGAAGGAGACGCTGCTAAGGGCAGACCCTCCCTCTGTTCGAGTGACTTGTCACACTGCCCTGGATGGAAAGGTGACCCTTAGGTGTAGGGCCCAGAACTTTTACCCTTCAGAGATTTCCCTGACTTGGCTGAGGGATGGGGAGGAACAGCTCCAGGACATGGAATTCATTGAGACCAGGCCTGCTGGAGATGGGACTTTCCAGAAGTGGGCAGCCGTGGAGATCACCTTGGGCCAGGAAGAGAAATATATCTGCCTAGTTCAGCATGAGGGACTTCCTGAGCCCCTCATAGTGAAATGGGAGCCACAGTCCTCACCCACCTGGATCACTGTGGGGATTGCTGTTGTCCTCATTGCAGTCATTGCTGGAGTTGTGATCTGGAGGAAAAGGAATTCAGGTGGACAAGGAGGGGTCCATGTTCGGCTGCAGACAATGACAGTGTACAGTGGTCAGATGTCTGTCCCACAGAGAATGGTGAGATTATGGAGGCTGGGAAAGTGA

>MHCI_5_UK_Hap_1

ATGGGACCTGATATACTCTCTCTCTTTTTGTTGGGGACTCTGGCCTTGACCAAGACCTGGGCAGGATTTCACTCCCTGAAGTATTTCCAAACTTCCATATCTCTGCCTGGCCTCGAAAAGCCGAAGTTCATTTCCGCAGGCTACGTGAACTATCGACAATTCGTACGCTTTGACAGCGACAGCTCGAGTCAGAGGGAGGAGCCCTTGGTGCCGTGGATGGACCAGATGGGTCAGGGGTACTGGGAGAGGAACTCACGTGTCGTCAGGGAGACCGCGCACACTTTCGAAGTAGGCCTGCAGAATCTGCAGGTTTATTACAATCAGAGCGAGGGAGAAGTTCACATCTATCAGCGCCTGGTCGGCTGCGAGACGTATTCCAACGGGACCTTTAGGCGTGGGTTTGAGCAATTCGCCTACGATGGGCAAGACTACATCTCTCCGGATCAGAAGACGCTGAGTTGGACGGCTTTGGTGCCCCCGGCTCTGAACACCAAGTACAAGTGGGAGGAGGATCGCAGCATGGCCAAGAGACAGAAAGTTTATCTGGAGGAGAAGTGTGTTGAGTGGCTGCACAAGTACCTGGAGTTGGGGAAGGAGACGCTGCTAAGGGCAGACCCTCCCTCTGTTTGAGTGACTTGTCACACTGCCCTGGATGGAAAGGTGACCCTTAGGTGTAGGGCCCAGAACTTTTACCCTTCAGAGATTTCCCTGATTTGGCTGAGGGATGGGGAGGAACAGCTCCAGGACATGGAATTCATTGAGACCAGGCCTGCTGGAGATGGGACTTTCCAGAAGTGGGCAGCCGTGGAGATCACCTTGGGCCAGGAAGAGAAATATATCTGCCTAGTTCAGCATGAGGGACTTCCTGAGCCCCTCATAGTGAAATGGGAGCCACAGTCCTCACCCACCTGGATCACTGTGGGGATTGCTGTTGTCCTCATTGCAGTCATTGCTGGAGTTGTGATCTGGAGGAAAAGGAATTCAGGTGGACAAGGAGGGGTCCATGTTCGGCTGCAGACAATGACAGTGTACAGTGGTCAGATGTCTGTCCCACAGAGAATGGTGAGATTATGGAGGCTGGGAAAGTGA

>MHCI_8_UC_Hap_1

ATGGAAATTTATATGCTGCCTCTACTTTTGTTGAGTGTCCTGGTCCTTACAGAGACCTGGGCTGGCTCTCACTCCTTGAAGTATTTTTACGCCGTAATGTCTCGACCCGAGCTAGCAAAACCAAAGTTCATCTCTGTAACCTACGTGGACGATCAGCAGGTCTTGAGCTTTGACAGCGACCACGAGAGTCAGAGCCCAGCGCCCAGGACGCCGTGGATCCAGCCCGACTACTGGGAGCGGGAGACAGAGATCTTCAGGGAAGCCACTGAACGTTACCGAGTATGCCTGCGGAAAGTGTCTGGGGACTACAACCATAGTGAGGGAGGGGTTCATACATTCCAGCAACTGTCGGGATGCGAGGTATTCTCCAACGGGAGCTTCAGCCGCGGCTTCGTGCAATACGCCTACGACGGGCAGGACTTCTTAGCTCTGGATACCGAGACTCTGCGTTGGATTGCCGGGAACGCAGGGGCCCTAAACCATAAGCTCGAGCTGGAAGCAGATCAAAGCTTTACGAAATATTGGAAGGGCTATATAGAGGAGGAGTGCGTGTACTGGCTTCACAGATACCTGGAGAATGGAAAGGAGACACTGCTTGGGACAGATCCACCCTTTCTACAAGTGACCAGACACACAAGTGCTGACGGAGAAGTGACCTTGCAGTGCCGGGCCCAGGGCTTTTATCCTGCAGAGATCTCACTGACTTGGCTGAGGGATGGGGAGGAACAGCTCCAGGAGACGGAGCTCATTGAGACCAGACCTGCGGGAGATGGGACCTTCCAGAAGTGGGCAGCTGTGGGGATGCTCTCTGGAAGCGAACAGAAATATACCTGCCGAGTGCAGCATGAGGGATTACCTGAGCCAGTCTTCCTGAAATGGGAGCCACAGTCCTCATCCGTAGGGCTCTCGGTAGGGGTCACCACTGCTCTCCTCCTCCTCCTCGCTGCAGTCATTGTTGGGGTTGTGATCTGGAGGAAAAATGCTTCAGATAGTAAAAGAGGGAGCTACACTACAACTGCAAGTAGCGATAGCGCCCAGGAATCAGATGTCTCTCTTACAGCAAGAGCCTAA

>MHCI_8_UC_Hap_2

ATGGAAATTTATATGCTGCCTCTACTTTTGTTGAGTGTCCTGGTCCTTACAGAGACCTGGGCTGGCTCTCACTCCTTGAAGTATTTTTACGCCGTAATGTCTCGACCCGAGCTAGCAAAACCAAAGTTCATCTCTGTAACCTACGTGGACGATCAGCAGGTCTTGAGCTTTGACAGCGACCACGAGAGTCAGAGCCCAGAGCCCAGGACGCCGTGGATCCAGCCCGACTACTGGGAGCGGGAGACAGAGATCTTCAGGGAAGCCACTGAACGTTACCGAGTATGCCTGCGGAAAGTGTCTGGGGACTACAACCATAGTGAGGGAGGGGTTCATACATTCCAGCAACTGTCGGGATGCGAGGTATTCTCCAACGGGAGCTTCAGCCGCGGCTTCGTGCAATACGCCTACGACGGGCAGGACTTCTTAGCTCTGGATACCGAGACTCTGCGTTGGATTGCCGGGAACGCAGGGGCCCTAAACCATAAGCTCGAGTTGGAAGCAGATCAAAGCTTTACGAAATATTGGAAGGGCTATATAGAGGAGGAGTGCGTGTACTGGCTTCACAGATACCTGGAGAATGGAAAGGAGACACTGCTTGGGACAGATCCACCCTTTCTACAAGTGACCAGACACACAAGTGCTGACGGAGAAGTGACCTTGCAGTGCCGGGCCCAGGGCTTTTATCCTGCAGAGATCTCACTGACTTGGCTGAGGGATGGGGAGGAACAGCTCCAGGAGACGGAGCTCATTGAGACCAGACCTGCGGGAGATGGGACCTTCCAGAAGTGGGCAGCTGTGGGGATGCTCTCTGGAAGCGAACAGAAATATACCTGCCGAGTGCAGCATGAGGGATTACCTGAGCCAGTCTTCCTGAAATGGGAGCCACAGTCCTCATCCGTAGGGCTCTCGGTAGGGGTCACCACTGCTCTCCTCCTCCTCCTCGCTGCAGTCATTGTTGGGGTTGTGATCTGGAGGAAAAATGCTTCAGATAGTAAAAGAGGGAGCTACACTACAACTGCAAGTAGCGATAGCGCCCAGGAATCAGATGTCTCTCTTACAGCAAGAGCCTAA

>MHCI_8_UC_Hap_3

ATGGAAATTTATATGCTGCCTCTACTTTTGTTGAGTGTCCTGGTCCTTACAGAGACCTGGGCTGGCTCTCACTCCTTGAAGTATTTTTACGCCGTAATGTCTCGACCCGAGCTAGCAAAACCAAAGTTCATCTCTGTAACCTACGTGGACGATCAGCAGGTCTTGAGCTTTGACAGCGACCACGAGAGTCAGAGCCCAGAGCCCAGGACGCCGTGGATCCAGCCCGACTACTGGGAGCGGGAGACAGAGATCTTCAGGGAAGCCACTGAACGTTACCGAGTATGCCTGCGGAAAGTGTCTGGGGACTACAACCATAGTGAGGGAGGGGTTCATACATTCCAGCAACTGTCGGGATGCGAGGTATTCTCCAACGGGAGCTTCAGCCGCGGCTTCGTGCAATACGCCTACGACGGGCAGGACTTCTTAGCTCTGGATACCGAGACTCTGCGTTGGATTGCCGGGAACGCAGGGGCCCTAAACCATAAGCTCGAGCTGGAAGCAGATCAAAGCTTTACGAAATATTGGAAGGGCTATATAGAGGAGGAGTGCGTGTACTGGCTTCACAGATACCTGGAGAATGGAAAGGAGACACTGCTTGGGACAGATCCACCCTTTCTACAAGTGACCAGACACACAAGTGCTGACGGAGAAGTGACCTTGCAGTGCCGGGCCCAGGGCTTTTATCCTGCAGAGATCTCACTGACTTGGCTGAGGGATGGGGAGGAACAGCTCCAGGAGACGGAGCTCATTGAGACCAGACCTGCGGGAGATGGGACCTTCCAGAAGTGGGCAGCTGTGGGGATGCTCTCTGGAAGCGAACAGAAATATACCTGCCGAGTGCAGCATGAGGGATTACCTGAGCCAGTCTTCCTGAAATGGGAGCCACAGTCCTCATCCGTAGGGCTCTCGGTAGGGGTCACCACTGCTCTCCTCCTCCTCCTCGCTGCAGTCATTGTTGGGGTTGTGATCTGGAGGAAAAATGCTTCAGATAGTAAAAGAGGGAGCTACACTACAACTGCAAGTAGCGATAGCGCCCAGGAATCAGATGTCTCTCTTACAGCAAGAGCCTAA

>MHCI_8_UC_Hap_4

ATGGAAATTTATATGCTGCCTCTACTTTTGTTGAGTGTCCTGGTCCTTACAGAGACCTGGGCTGGCTCTCACTCCTTGAAGTATTTTTACGCCGTAATGTCTCGACCCGAGCTAGCAAAACCAAAGTTCATCTCTGTAACCTACGTGGACGATCAGCAGGTCTTGAGCTTTGACAGCGACCACGAGAGTCAGAGCCCAGCGCCCAGGACGCCGTGGATCCAGCCCGACTACTGGGAGCGGGAGACAGAGATCTTCAGGGAAGCCACTGAACGTTACCGAGTATGCCTGCGGAAAGTGTCTGGGGACTACAACCATAGTGAGGGAGGGGTTCATACATTCCAGCAACTGTCGGGATGCGAGGTATTCTCCAACGGGAGCTTCAGCCGCGGCTTCGTGCAATACGCCTACGACGGGCAGGACTTCTTAGCTCTGGATACCGAGACTCTGCGTTGGATTGCCGGGAACGCAGGGGCCCTAAACCATAAGCTCGAGTTGGAAGCAGATCAAAGCTTTACGAAATATTGGAAGGGCTATATAGAGGAGGAGTGCGTGTACTGGCTTCACAGATACCTGGAGAATGGAAAGGAGACACTGCTTGGGACAGATCCACCCTTTCTACAAGTGACCAGACACACAAGTGCTGACGGAGAAGTGACCTTGCAGTGCCGGGCCCAGGGCTTTTATCCTGCAGAGATCTCACTGACTTGGCTGAGGGATGGGGAGGAACAGCTCCAGGAGACGGAGCTCATTGAGACCAGACCTGCGGGAGATGGGACCTTCCAGAAGTGGGCAGCTGTGGGGATGCTCTCTGGAAGCGAACAGAAATATACCTGCCGAGTGCAGCATGAGGGATTACCTGAGCCAGTCTTCCTGAAATGGGAGCCACAGTCCTCATCCGTAGGGCTCTCGGTAGGGGTCACCACTGCTCTCCTCCTCCTCCTCGCTGCAGTCATTGTTGGGGTTGTGATCTGGAGGAAAAATGCTTCAGATAGTAAAAGAGGGAGCTACACTACAACTGCAAGTAGCGATAGCGCCCAGGAATCAGATGTCTCTCTTACAGCAAGAGCCTAA

>MHCI_9_UE_Hap_2

ATGGACATTTATATGCTGCCTCTACTTTTGTTGAGTGTCCTGGTCCTTACAGAGACCTGGGCTGGCTCTCACTCCTTGAAGTATTTTTACGCCGTAATGTCTCGACCCGAGCTAGCAAAACCAAGGTTCATCTCTGTAACCTACGTGGACGATCGGCAGGTCTTGAGCTTTGACAGCGACCATGAGAGTCAGAGCCCAGAGCCCAGGACGCCGTGGATCCGGCCCGACTACTGGGAGCGGGAGACAGAGATCTTCAGGGAAGACACTGAACGTTACCGAATATGCCTGCGGAAAGTGTCTGGATACTATAACCACAGTGAGGGAGGGGTTCATACATTCCAGCGACTGTCGGGCTGCGAGGTATTCTCCAACGGGAGCTTCAGCCGCGGCTTCGTGCAATACGCCTACGACGGGCAGGACTACCTGGCGCTGGATACCGAGACGCTGCGTTGGATTGCCGGGAACGCAGGGGCCCTAAACCATAAGCTCGAGCTGGAAGCAGATCAAAGCTTTACGAAATATTGGAAGGGCTACGTAGAGGAGGAGTGCGTGTACTGGCTTCACAGATATCTGGAGAATGGAAAGGAGACACTGCTTGGGACAGATCCGCCCTCTGTACAATTGACGAGACACACAACTTCTGAGGGTCAAGTGACTTTGCGGTGCCGGGCCCATGGCTTTTATCCTGCGGAGATCTCTCTTACTTGGCTGAGGGATGGGGAGGAACAGCTCCAGGAGACGGAGCTCATTGAGACCAGACCTGCGGGAGATGGGACCTTCCAGAAGTGGGCAGCTGTGGGGATGCTCTCTGGAAGTGAACAGAAATATACCTGCCGAGTACAGCACGAGGGACGACCTGAGCCGCTCTTTTTGAAATGGGAGCCACAGTCCTCATCCGTAGGGCTCTCGGTAGGGGTCACCACTGCTCTCCTCCTCCTCCTCGCTGCAGTCATTGTTGGGGTTGTGATCTGGAGGAAAAATGCTTCAGATGGTAAAAGAGGGAGCTACACTACAACTGCAAGTAGCGATAGCGCCCAGGAATCAGATGTCTCTCTTACAGCAAGAGCCTAA

>MHCI_9_UE_Hap_1

ATGGAAATTTATATGCTGCCTCTACTTTTGTTGAGTGTCCTGGTCCTTACAGAGACCTGGGCTGGCTCTCACTCCTTGAAGTATTTTTACGCCGTAATGTCTCGACCCGAGCTAGCAAAACCAAGGTTCATCTCTGTAACCTACGTGGACGATCGGCAGGTCTTGAGCTTTGACAGCGACCATGAGAGTCAGAGCCCAGAGCCCAGGACGCCGTGGATCCgGCCCGACTACTGGGAGCGGGAGACAGAGATCTTCAGGgAAGACACTGAACGTTACCGAATATGCCTGCGGAAAGTGTCTGGATACTATAACCACAGTGAGGGAGGGGTTCATACATTCCAGCGACTGTCGGGCTGCGAGGTATTCTCCAACGGGAGCTTCAGCCGCGGCTTCGTGCAATACGCCTACGACGGGCAGGACTACCTGGCGCTGGATACCGAGACGCTGCGTTGGATTGCCGGGAACGCAGGGGCCCTAAACCATAAGCTCGAGCTGGAAGCAGATCAAAGCTTTACGAAATATTGGAAGGGCTACGTAGAGGAGGAGTGCGTGTACTGGCTTCACAGATATCTGGAGAATGGAAAGGAGACACTGCTTGGGACAGATCCGCCCTCTGTACAATTGACGAGACACACAACTTCTGAGGGTCAAGTGACTTTGCGGTGCCGGGCCCATGGCTTTTATCCTGCGGAGATCTCTCTTACTTGGCTGAGGGATGGGGAGGAACAGCTCCAGGAGACGGAGCTCATTGAGACCAGACCTGCGGGAGATGGGACCTTCCAGAAGTGGGCAGCTGTGGGGATGCTCTCTGGAAGTGAACAGAAATATAGCTGCCGAGTACAGCACGAGGGACGACCTGAGCCGCTCTTTTTGAAATGGGAGCCACAGTCCTCATCCGTAGGGCTCTCGGTAGGGGTCACCACTGCTCTCCTCCTCCTCCTCGCTGCAGTCATTGTTGGGGTTGTGATCTGGAGGAAAAATGCTTCAGATGGTAAAAGAGGGAGCTACACTACAACTGCAAGTAGTGATAGCGCCCAGGAATCAGATGTCTCTCTTACAGCAAGAGCCTAA

>MHCI_9_UE_Hap_3

ATGGAAATTTATATGCTGCCTCTACTTTTGTTGAGTGTCCTGGTCCTTACAGAGACCTGGGCTGGCTCTCACTCCTTGAAGTATTTTTACGCCGTAATGTCTCGACCCGAGCTAGCAAAACCAAGGTTCATCTCTGTAACCTACGTGGACGATCGGCAGGTCTTGAGCTTTGACAGCGACCATGAGAGTCAGAGCCCAGAGCCCAGGACGCCGTGGATCCGGCCCGACTACTGGGAGCGGGAGACAGAGATCTTCAGGGAAGACACTGAACGTTACCGAATATGCCTGCGGAAAGTGTCTGGATACTATAACCACAGTGAGGGAGGGGTTCATACATTCCAGCGACTGTCGGGCTGCGAGGTATTCTCCAACGGGAGCTTCAGCCGCGGCTTCGTGCAATACGCCTACGACGGGCAGGACTACCTGGCGCTGGATACCGAGACGCTGCGTTGGATTGCCGGGAACGCAGGGGCCCTAAACCATAAGCTCGAGCTGGAAGCAGATCAAAGCTTTACGAAATATTGGAAGGGCTACGTAGAGGAGGAGTGCGTGTACTGGCTTCACAGATACCTGGAGAATGGAAAGGAGACACTGCTTGGGACAGATCCGCCCTCTGTACAATTGACGAGACACACAACTTCTGAGGGTCAAGTGACTTTGCGGTGCCGGGCCCATGGCTTTTATCCTGCGGAGATCTCTCTTACTTGGCTGAGGGATGGGGAGGAACAGCTCCAGGAGACGGAGCTCATTGAGACCAGACCTGCGGGAGATGGGACCTTCCAGAAGTGGGCAGCTGTGGGGATGCTCTCTGGAAGTGAACAGAAATATAGCTGCCGAGTACAGCACGAGGGACGACCTGAGCCGCTCTTTTTGAAATGGGAGCCACAGTCCTCATCCGTAGGGCTCTCGGTAGGGGTCACCACTGCTCTCCTCCTCCTCCTCGCTGCAGTCATTGTTGGGGTTGTGATCTGGAGGAAAAATGCTTCAGATGGTAAAAGAGGGAGCTACACTACAACTGCAAGTAGTGATAGCGCCCAGGAATCAGATGTCTCTCTTACAGCAAGAGCCTAA

>MHCI_9_UE_Hap_4

ATGGAAATTTATATGCTGCCTCTACTTTTGTTGAGTGTCCTGGTCCTTACAGAGACCTGGGCTGGCTCTCACTCCTTGAAGTATTTTTACGCCGTAATGTCTCGACCCGAGCTAGCAAAACCAAGGTTCATCTCTGTAACCTACGTGGACGATCGGCAGGTCTTGAGCTTTGACAGCGACCATGAGAGTCAGAGCCCAGAGCCCAGGACGCCGTGGATCCGGCCCGACTACTGGGAGCGGGAGACAGAGATCTTCAGGGAAGACACTGAACGTTACCGAATATGCCTGCGGAAAGTGTCTGGATACTATAACCACAGTGAGGGAGGGGTTCATACATTCCAGCGACTGTCGGGCTGCGAGGTATTCTCCAACGGGAGCTTCAGCCGCGGCTTCGTGCAATACGCCTACGACGGGCAGGACTACCTGGCGCTGGATACCGAGACGCTGCGTTGGATTGCCGGGAACGCAGGGGCCCTAAACCATAAGCTCGAGCTGGAAGCAGATCAAAGCTTTACGAAATATTGGAAGGGCTACGTAGAGGAGGAGTGCGTGTACTGGCTTCACAGATATCTGGAGAATGGAAAGGAGACACTGCTTGGGACAGATCCGCCCTCTGTACAATTGACGAGACACACAACTTCTGAGGGTCAAGTGACTTTGCGGTGCCGGGCCCATGGCTTTTATCCTGCGGAGATCTCTCTTACTTGGCTGAGGGATGGGGAGGAACAGCTCCAGGAGACGGAGCTCATTGAGACCAGACCTGCGGGAGATGGGACCTTCCAGAAGTGGGCAGCTGTGGGGATGCTCTCTGGAAGTGAACAGAAATATAGCTGCCGAGTACAGCACGAGGGACGACCTGAGCCGCTCTTTTTGAAATGGGAGCCACAGTCCTCATCCGTAGGGCTCTCGGTAGGGGTCACCACTGCTCTCCTCCTCCTCCTCGCTGCAGTCATTGTTGGGGTTGTGATCTGGAGGAAAAATGCTTCAGATGGTAAAAGAGGGAGCTACACTACAACTGCAAGTAGCGATAGCGCCCAGGAATCAGATGTCTCTCTTACAGCAAGAGCCTAA

>MHCI_9_UE_Hap_5

ATGGAAATTTATATGCTGCCTCTACTTTTGTTGAGTGTCCTGGTCCTTACAGAGACCTGGGCTGGCTCTCACTCCTTGAAGTATTTTTACGCCGTAATGTCTCGACCCGAGCTAGCAAAACCAAGGTTCATCTCTGTAACCTACGTGGACGATCGGCAGGTCTTGAGCTTTGACAGCGACCATGAGAGTCAGAGCCCAGAGCCCAGGACGCCGTGGATCCGGCCCGACTACTGGGAGCGGGAGACAGAGATCTTCAGGGAAGACACTGAACGTTACCGAATATGCCTGCGGAAAGTGTCTGGATACTATAACCACAGTGAGGGAGGGGTTCATACATTCCAGCGACTGTCGGGCTGCGAGGTATTCTCCAACGGGAGCTTCAGCCGCGGCTTCGTGCAATACGCCTACGACGGGCAGGACTACCTGGCGCTGGATACCGAGACGCTGCGTTGGATTGCCGGGAACGCAGGGGCCCTAAACCATAAGCTCGAGCTGGAAGCAGATCAAAGCTTTACGAAATATTGGAAGGGCTACGTAGAGGAGGAGTGCGTGTACTGGCTTCACAGATACCTGGAGAATGGAAAGGAGACACTGCTTGGGACAGATCCGCCCTCTGTACAATTGACGAGACACACAACTTCTGAGGGTCAAGTGACTTTGCGGTGCCGGGCCCATGGCTTTTATCCTGCGGAGATCTCTCTTACTTGGCTGAGGGATGGGGAGGAACAGCTCCAGGAGACGGAGCTCATTGAGACCAGACCTGCGGGAGATGGGACCTTCCAGAAGTGGGCAGCTGTGGGGATGCTCTCTGGAAGTGAACAGAAATATAGCTGCCGAGTACAGCACGAGGGACGACCTGAGCCGCTCTTTTTGAAATGGGAGCCACAGTCCTCATCCGTAGGGCTCTCGGTAGGGGTCACCACTGCTCTCCTCCTCCTCCTCGCTGCAGTCATTGTTGGGGTTGTGATCTGGAGGAAAAATGCTTCAGATGGTAAAAGAGGGAGCTACACTACAACTGCAAGTAGCGATAGCGCCCAGGAATCAGATGTCTCTCTTACAGCAAGAGCCTAA

>MHCI_9_UE_Hap_6

ATGGAAATTTATATGCTGCCTCTACTTTTGTTGAGTGTCCTGGTCCTTACAGAGACCTGGGCTGGCTCTCACTCCTTGAAGTATTTTTACGCCGTAATGTCTCGACCCGAGCTAGCAAAACCAAGGTTCATCTCTGTAACCTACGTGGACGATCGGCAGGTCTTGAGCTTTGACAGCGACCATGAGAGTCAGAGCCCAGAGCCCAGGACGCCGTGGATCCGGCCCGACTACTGGGAGCGGGAGACAGAGATCTTCAGGGAAGACACTGAACGTTACCGAATATGCCTGCGGAAAGTGTCTGGATACTATAACCACAGTGAGGGAGGGGTTCATACATTCCAGCGACTGTCGGGCTGCGAGGTATTCTCCAACGGGAGCTTCAGCCGCGGCTTCGTGCAATACGCCTACGACGGGCAGGACTACCTGGCGCTGGATACCGAGACGCTGCGTTGGATTGCCGGGAACGCAGGGGCCCTAAACCATAAGCTCGAGCTGGAAGCAGATCAAAGCTTTACGAAATATTGGAAGGGCTACGTAGAGGAGGAGTGCGTGTACTGGCTTCACAGATATCTGGAGAATGGAAAGGAGACACTGCTTGGGACAGATCCGCCCTCTGTACAATTGACGAGACACACAACTTCTGAGGGTCAAGTGACTTTGCGGTGCCGGGCCCATGGCTTTTATCCTGCGGAGATCTCTCTTACTTGGCTGAGGGATGGGGAGGAACAGCTCCAGGAGACGGAGCTCATTGAGACCAGACCTGCGGGAGATGGGACCTTCCAGAAGTGGGCAGCTGTGGGGATGCTCTCTGGAAGTGAACAGAAATATACCTGCCGAGTACAGCACGAGGGACGACCTGAGCCGCTCTTTTTGAAATGGGAGCCACAGTCCTCATCCGTAGGGCTCTCGGTAGGGGTCACCACTGCTCTCCTCCTCCTCCTCGCTGCAGTCATTGTTGGGGTTGTGATCTGGAGGAAAAATGCTTCAGATGGTAAAAGAGGGAGCTACACTACAACTGCAAGTAGCGATAGCGCCCAGGAATCAGATGTCTCTCTTACAGCAAGAGCCTAA

>MHCI_4_UA_Hap_2

ATGGAGCCTTATCTGCGCGCTCTCTTTTTGCTGGGGACCCTGGCCCTGCCGGAGACCTGGGCAGGCTCTCACTCCCTGAGGTATTTCGACGCCGCCGTGGCCTCCCCCGAGCTCGCGGAGCCGCGGTTCCTCACCGTGGGCTACGTGGACGATCAGCAGTTCGTGCGCTTCGACAGTGCCCGCGCGAGTCCGAGTATGGAGCCGCGGGCGGCGTGGATAGAGCGGGTGCAGCAGGAGGAGCCGGGCTACTGGGACCAGGAGACGCGGAACATGAAGGCGGTCACACAGACTTACCGAGTGAGCCTGCAGAACCTCCGCGGCTACTTCAACCAGAGCGAAGGCGGGGTCCACACCATCCAGCACATGTACGGCTGCGAGGTCTCCCCCGAGCTCACCTTCAAGCGCGGGTTTCTCCAATACGCCTACGACGGGCGGGACTACATCGCCCTGGACTCGGAGACCTCCACGTGGACGGCGGAGGTGCCGCAGGCTCTGAACACCAAGCGCAAGTGGGAGGCGGAAAAGAGCATCGCGGAGGGACAGAAAGCCTACCTGGAGGAGACGTGCGTGCTGTGGCTGAAGAAGTACCTGGAGATGGGGAAGGAGACGCTGAAGAGGACAGACCCGCCCTCTGCCCGAGTGACCCGCCACACTGGCCCCCACGGGGAGGTGACCCTGCGGTGCCGGGCCCAGGACTTCTACCCCGAGGACATCTCCCTGACGTGGCTGAGGGATGGGGAGGAGCAGCTCCAGGACGCGGAGTTCATTGAGACCAGGCCCGCGGGGGAGGGGACCTTCCAGAAGTGGGCAGGTGTGGACGTGACCTCGGGCCAGGAAGGGAAATATACCTGCCGAGTTCAGCACGAGGGACTGCCAGAGCCGCTCACCCTGAAGTGGGAGCCAGAGTCCTCATCCCCCTGGCTCATTGTGGGGGGCATTGCTGTCCTCCTCCTCCTCACTGCAGCCATTGCTGGAGTTGTGATCTGGAAGAAGAAGACTTCAGGTGGAAAAGGAGGGGACTATGTTCGTGCTGCAGGCAATGACAGTGCACAGGGGTCAGATGTCTCCCTCACAGTCAAAGCTTGA

>MHCI_4_UA_Hap_1

ATGGAGCCTTATCTGCGCGCTCTCTTTTTGCTGGGGACCCTGGCCCTGCCGGAGACCTGGGCAGGCTCTCACTCCCTGAGGTATTTCGACACCGCCGTGGCCTCCCCCGAGCTCGCGGAGCCGCGGTTCCTCACCGTGGGCTACGTGGACGATCAGCAGTTCGTGCGCTTCGACAGTGCCCGCGCGAGTCCGAGGGAGGAGCCGCGGGCGGCGTGGATAGAGCGGGTGGAGCAGGAGGAGCCGGGCTACTGGGAGCAGGAAACGCAGAACATGAAGGCGGTCACACAGACTTACCGAGTGAACCTGCAGAACCTCCGCGGCTACTTCAACCAGAGCGAAGGCGGGGTCCACACCATCCAGCACATGTACGGCTGCGAGGTCTCCCCCGAGCTCACCTTCAAGCGCGGGTTTCTCCAATACGCCTACGACGGGCGGGACTACATCGCCCTGGACTCGGAGACCTCCACGTGGACGGCGGAGGTGCCGCAGGCTCTGAACACCAAGCGCAAGTGGGAGGCGGAAAAGAGCATCGCGGAGGGACAGAAAGCCTACCTGGAGGAGACGTGCGTGCTGTGGCTGAAGAAGTACCTGGAGATGGGGAAGGAGACGCTGAAGAGGACAGACCCGCCCTCCGCCCGAGTGACCCGCCACACTGGCCCCCACGGGGAGGTGACCCTGCGGTGCCGGGCCCAGGACTTCTACCCCGCGGACATCTCCCTGACGTGGCTGAGGAATGGGGAGGAGCAGCTCCAGGACGCGGAGTTCATTGAGACCAGGCCCGCGGGGGAGGGGACCTTCCAGAAGTGGGCAGGTGTGGACGTGACCTCGGGCCAGGAAGGGAAATATACCTGCCGAGTTCAGCACGAGGGACTGCCAGAGCCGCTCACCCTGAAGTGGGAGCCAGAGTCCTCATCCCCCTGGCTCATTGTGGGGGGCATTGCTGTCCTCCTCCTCCTCACTGCAGCCATTGCTGGAGTTGTGATCTGGAAGAAGAAGACTTCAGGTGGAAAAGGAGGGGACTATGTTCGTGCTGCAGGCAATGACAGTGCACAGGGGTCAGATGTCTCCCTCACAGTCAAAGCTTGA

>MHCI_4_UA_Hap_4

ATGGAGCCTTATCTGCGCGCTCTCTTTTTGCTGGGGACCCTGGCCCTGCCGGAGACCTGGGCAGGCTCTCACTCCCTGAGGTATTTCTACACCGCCGTGGCCTCCCCCGAGCTCGCGGAGCCGCGGTTCCTCATCGTGGGCTACGTGGACGATCAGCAGTTCGTGCGCTTCGACAGTGCCCGCGCGAGTCCGAGTATGGAGCCGCGGGCGGCATGGATAGAGCGGGTGGAGCAGGAGGAGCCGGGCTACTGGGAGCGGCAAACGCAGAACATGAAGGCGGTCACACAGACTTACCGAGTGAGCCTGCAGAACCTCCGCGGCTACTTCAACCAGAGCGAAGGCGGGGTCCACACCTTTCAGAACATGTACGGCTGCGAGGTCTCCCCCGAGCTCACCTTCAAGCGCGGGTTTCTCCAATACGCCTACGACGGGCGGGACTATATCGCCCTGGACTCGGAGACCTCCACGTGGACGGCGGAGGTGCCGCAGGCTGTGAACACCAAACGCAAGTGGGAGGCAGAAAAGAGCATCGCGGAGGGACAGAAAGCCTACCTGGAGGAGACGTGCGTGCTGTGGCTGAAGAAGTACCTGGAGATGGGGAAGGAGACGCTGAAGAGGACAGACCCGCCCTCTGCCCGAGTGACCCGCCACACTGACCCCCACGGGGAGGTGACCCTGCGGTGCCGGGCCCAGGACTTCTACCCCGAGGACATCTCCCTGACTTGGCTGAGGGATGGGGAGGAGCAGCTCCAGGACGCGGAGTTCATTGAGACCAGGCCCGCGGGGGAGGGGACCTTCCAGAAGTGGGCAGGTGTGGACGTGACCTTGGGCCAGGAAGGGAAATATACCTGCCGAGTTCAGCACGAGGGACTGCCTGAGCCCCTCACCCTGAAGTGGGAGCCAGAGTCCTCATTCCCCTGGTTCATTGTGGGGGGCGTTGCTGTCCTCCTCCTCCTCATTGCAGCCATTGCTGGAGTTGTGATCTGGAAGAAGAAGACTTCAGGTGGAAAAGGAGGGGACTATGTTCGTGCTGCAGGCAATGACAGTGCACAGGGGTCAGATGTCTCCCTCACAGTCAAAGCTTGA

>MHCI_4_UA_Hap_7

ATGGAGCCTTATCTGCGCGCTCTCTTTTTGCTGGGGACCCTGGCCCTGCCGGAGACCTGGGCAGGCTCTCACTCCCTGAGGTATTTCGACGCCGCCGTGGCCTCCCCCGAGCTCGCGGAGCCGCGGTTCCTCATCGTGGGCTACGTGGACGATCAGCAGTTCGTGCGCTTCGACAGTGCCCGCGCGAGTCCGAGTATGGAGCCGCGGGCGGCGTGGATAGAGCGGGTGGAGCAGGAGGAGCCGGGCTACTGGGAGCGGCAAACGCAGAACATGAAGGCGGTCACACAGACTTACCGAGTGAGCCTGCAGAACCTCCGCGGCTACTTCAACCAGAGCGAAGGCGGGGTCCACACCATCCAGCACATGTACGGCTGCGAGGTCTCCCCCGAGCTCACCTTCAAGCGCGGGTTTCACCAATACGCCTACGACGGGCGGGACTACATCGCCCTGGACTCGGAGACCTCCACGTGGACGGCGGAGGTGCCGCAGGCTCTGAACACCAAGCGCAAGTGGGAGGCGGAAAAGAGCATCGCGGAGGGACAGAAAGCCTACCTGGAGGAGACGTGCGTGCTGTGGCTGAAGAAGTACCTGGAGATGGGGAAGGAGACGCTGAAGAGGACAGACCCGCCCTCCGCCCGAGTGACCCGCCACACTGGCCCCCACGGGGAGGTGACCCTGCGGTGCCGGGCCCAGGACTTCTACCCCGCGGACATCTCCCTGACGTGGCTGAGGAATGGGGAGGAGCAGCTCCAGGACGCGGAGTTCATTGAGACCAGGCCCGCGGGGGAGGGGACCTTCCAGAAGTGGGCAGGTGTGGACGTGACCTCGGGCCAGGAAGGGAAATATACCTGCCGAGTTCAGCACGAGGGACTGCCTGAGCCCCTCACCCTGAAGTGGGAGCCAGAGTCCTCATTCCCCTGGTTCATTGTGGGGGGCGTTGCTGTCCTCCTCCTCCTCACTGCAGCCATTGCTGGAGTTGTGATCTGGAAGAAGAATACTTCAGGTGGAAAAGGAGGGGACTATGTTCGTGCTGCAGGCAATGACAGTGCACAGGGGTCAGATGTCTCCCTCACAGTCAAAGCTTGA

>MHCI_4_UA_Hap_8

ATGGAGCCTTATCTGCGCGCTCTCTTTTTGCTGGGGACCCTGGCCCTGCCGGAGACCTGGGCAGGCTCTCACTCCCTGAGGTATTTCTACACCGCCGTGGCCTCCCCCGAGCTCGCGGAGCCGCGGTTCCTCATCGTGGGCTACGTGGACGATCAGCAGTTCGTGCGCTTCGACAGTGCCCGCGCGAGTCCGAGTATGGAGCCGCGGGCGGCATGGATAGAGCGGGTGCAGCAGGAGGAGCCGGGCTACTGGGACCAGGAGACGCGGAACATGAAGGCGGTCACACAGACTTACCGAGTGAGCCTGCAGAACCTCCGCGGCTACTTCAACCAGAGCGAAGGCGGGGTCCACACCATCCAGCACATGTACGGCTGCGAGGTCTCCCCCGAGCTCACCTTCAAGCGCGGGTTTCTCCAATACGCCTACGACGGGCGGGACTACATCGCCCTGGACTCGGAGACCTCCACGTGGACGGCGGAGGTGCCGCAGGCTCTGAACACCAAGCGCAAGTGGGAGGCGGAAAAGAGCTACACGGAGGGACAGAAAGCCTACCTGGAGGAGACGTGCGTGCTGTGGCTGAAGAAGTACCTGGAGATGGGGAAGGAGACGCTGAAGAGGACAGAACCGCCCTCCGCCCGAGTGACCCGCCACACTGGCCCCCACGGGGAGGTGACCCTGCGGTGCCGGGCCCAGGACTTCTACCCCGAGGACGTCTCCCTGACGTGGCTGAGGAATGGGGAGGAGCAGCTCCAGGACGCGGAGTTCATTGAGACCAGGCCGGCGGGGGAGGGGACCTTCCAGAAGTGGGCAGGTGTGGACGTGACCTCGGGCCAGGAAGGGAAATATACCTGCCGAGTTCAGCACGAGGGACTGCCTGAGCCCCTCACCCTGAAGTGGGAGCCAGAGTCCTCATTCCCCTGGTTCATCGTGGGGGGCATTGCTGTCCTCCTCCTCCTCATTGCAGCCATTGCTGGAGTTGTGATCTGGAAGAAGAAGACTTCAGGTGGAAAAGGAGGGGACTATGTTCGTGCTGCAGGCAATGACAGTGCACAGGGGTCAGATGTCTCCCTCACAGTCAAAGCTTGA

>MHCI_4_UA_Hap_10

ATGGAGCCTTATCTGCGCGCTCTCTTTTTGCTGGGGACCCTGGCCCTGCCGGAGACCTGGGCAGGCTCTCACTCCCTGAGGTATTTCTACACCGCCGTGGCCTCCCCCGAGCTCGCGGAGCCGCGGTTCCTCACCGTGGGCTACGTGGACGATCAGCAGTTCGTGCGCTTCGACAGTGCCCGCGCGAGTCCGAGTATGGAGCCGCGGGCGGCGTGGATAGAGCGGGTGCAGCAGGAGGAGCCGGGCTACTGGGAGCAGGAGACGCGGAACATGAAGGCGGTCACACAGACTTACCGAGTGAGCCTGCAGAACCTCCGCGGCTACTTCAACCAGAGCGAAGGCGGGGTCCACACCATCCAGCACATGTACGGCTGCGAGGTCTCCCCCGAGCTCACCTTCAAGCGCGGGTTTCACCAATACGCCTACGACGGGCGGGACTACATCGCCCTGGACTCGGAGACCTCCACGTGGACGGCGGAGGTGCCGCAGGCTCTGAACACCAAACGCAAGTGGGAGGCAGAAAAGAGCTACACGGAGGGACAGAAAGCCTACCTGGAGGAGACGTGCGTGCTGTGGCTGAAGAAGTACCTGGAGATGGGGAAGGAGACGCTGAAGAGGACAGAACCGCCCTCCGCCCGAGTGACCCGCCACACTGGCCCCCACGGGGAGGTGACCCTGCGGTGCCGGGCCCAGGACTTCTACCCCGAGGACGTCTCCCTGACGTGGCTGAGGAATGGGGAGGAGCAGCTCCAGGACGCGGAGTTCATTGAGACCAGGCCGGCGGGGGAGGGGACCTTCCAGAAGTGGGCAGGTGTGGACGTGACCTCGGGCCAGGAAGGGAAATATACCTGCCGAGTTCAGCACGAGGGACTGCCTGAGCCCCTCACCCTGAAGTGGGAGCCAGAGTCCTCATTCCCCTGGTTCATCGTGGGGGGCATTGCTGTCCTCCTCCTCCTCATTGCAGCCATTGCTGGAGTTGTGATCTGGAAGAAGAAGACTTCAGGTGGAAAAGGAGGGGACTATGTTCGTGCTGCAGGCAATGACAGTGCACAGGGGTCAGATGTCTCCCTCACAGTCAAAGCTTGA

>MHCI_4_UA_Hap_12

ATGGAGCCTTATCTGCGCGCTCTCTTTTTGCTGGGGACCCTGGCCCTGCCGGAGACCTGGGCAGGCTCTCACTCCCTGAGGTATTTCTACACCGCCGTGGCCTCCCCTGAGCTCGCGGAGCCGCGGTTCCTCACCGTGGGCTACGTGGACGATCAGGAGTTCGTGCGCTTCGACAGTGCCCGCGCGAGTCCGAGTATGGAGCCGCGGGCGGCGTGGATAGAGCGGGTGCAGCAGGAGGAGCCGGGCTACTGGGACCAGGAGACGCGGAACATGAAGGCGGTCACACAGACTTACCGAGTGAGCCTGCAGAACCTCCGCGGCTACTTCAACCAGAGCGAAGGCGGGGTCCACACCATCCAGCACATGTACGGCTGCGAGGTCTCCCCCGAGCTCACCTTCAAGCGCGGGTTTCACCAATACGCCTACGACGGGCGGGACTACATCGCCCTGGACTCGGAGACCTCCACGTGGACGGCGGAGGTGCCGCAGGCTCTGAACACCAAGCGCAAGTGGGAGGCAGAAAAGAGCTACACGGAGGGACAGAAAGCCTACCTGGAGGAGACGTGCGTGCTGTGGCTGAAGAAGTACCTGGAGATGGGGAAGGAGACGCTGAAGAGGACAGAACCGCCCTCCGCCCGAGTGACCCGCCACACTGGCCCCCACGGGGAGGTGACCCTGCGGTGCCGGGCCCAGGACTTCTACCCCGAGGACGTCTCCCTGACGTGGCTGAGGAATGGGGAGGAGCAGCTCCAGGACGCGGAGTTCATTGAGACCAGGCCGGCGGGGGAGGGGACCTTCCAGAAGTGGGCAGGTGTGGACGTGACCTCGGGCCAGGAAGGGAAATATACCTGCCGAGTTCAGCACGAGGGACTGCCTGAGCCCCTCACCCTGAAGTGGGAGCCAGAGTCCTCATTCCCCTGGTTCATCGTGGGGGGCATTGCTGTCCTCCTCCTCCTCATTGCAGCCATTGCTGGAGTTGTGATCTGGAAGAAGAAGACTTCAGGTGGAAAAGGAGGGGACTATGTTCGTGCTGCAGGCAATGACAGTGCACAGGGGTCAGATGTCTCCCTCACAGTCAAAGCTTGA

>MHCI_4_UA_Hap_13

ATGGAGCCTTATCTGCGCGCTCTCTTTTTGCTGGGGACCCTGGCCCTGCCGGAGACCTGGGCAGGCTCTCACTCCCTGAGGTATTTCTACACCGCCGTGGCCTCCCCCGAGCTCGCGGAGCCGCGGTTCCTCACCGTGGGCTACGTGGACGATCAGCAGTTCGTGCGCTTCGACAGTGCCCGCGCGAGTCCGAGTATGGAGCCGCGGGCGGCGTGGATAGAGCGGGTGCAGCAGGAGGAGCCGGGCTACTGGGACCAGGAGACGCGGAACATGAAGGCGGTCACACAGACTTACCGAGTGAGCCTGCAGAACCTCCGCGGCTACTTCAACCAGAGCGAAGGCGGGGTCCACACCATCCAGCACATGTACGGCTGCGAGGTCTCCCCCGAGCTCACCTTCAAGCGCGGGTTTCACCAATACGCCTACGACGGGCGGGACTACATCGCCCTGGACTCGGAGACCTCCACGTGGACGGCGGAGGTGCCGCAGGCTCTGAACACCAAACGCAAGTGGGAGGCAGAAAAGAGCTACACGGAGGGACAGAAAGCCTACCTGGAGGAGACGTGCGTGCTGTGGCTGAAGAAGTACCTGGAGATGGGGAAGGAGACGCTGAAGAGGACAGAACCGCCCTCCGCCCGAGTGACCCGCCACACTGGCCCCCACGGGGAGGTGACCCTGCGGTGCCGGGCCCAGGACTTCTACCCCGAGGACGTCTCCCTGACGTGGCTGAGGAATGGGGAGGAGCAGCTCCAGGACGCGGAGTTCATTGAGACCAGGCCGGCGGGGGAGGGGACCTTCCAGAAGTGGGCAGGTGTGGACGTGACCTCGGGCCAGGAAGGGAAATATACCTGCCGAGTTCAGCACGAGGGACTGCCTGAGCCCCTCACCCTGAAGTGGGAGCCAGAGTCCTCATTCCCCTGGTTCATCGTGGGGGGCATTGCTGTCCTCCTCCTCCTCATTGCAGCCATTGCTGGAGTTGTGATCTGGAAGAAGAAGACTTCAGGTGGAAAAGGAGGGGACTATGTTCGTGCTGCAGGCAATGACAGTGCACAGGGGTCAGATGTCTCCCTCACAGTCAAAGCTTGA

>MHCI_4_UA_Hap_17

ATGGAGCCTTATCTGCGCGCTCTCTTTTTGCTGGGGACCCTGGCCCTGCCGGAGACCTGGGCAGGCTCTCACTCCCTGAGGTATTTCTACACCGCCGTGGCCTCCCCCGAGCTCGCGGAGCCGCGGTTCCTCATCGTGGGCTACGTGGACGATCAGCAGTTCGTGCGCTTCGACAGTGCCCGCGCGAGTCCGAGTATGGAGCCGCGGGCGGCGTGGATAGAGCGGGTGGAGCAGGAGGAGCCGGGCTACTGGGACCAGGAGACGCGGAACATGAAGGCGGTCACACAGACTTACCGAGTGAACCTGCAGAACCTCCGCGGCTACTTCAACCAGAGCGAAGGCGGGGTCCACACCTTTCAGAACATGTACGGCTGCGAGGTCTCCCCCGAGCTCACCTTCAAGCGCGGGTTTCTCCAATACGCCTACGACGGGCGGGACTATATCGCCCTGGACTCGGAGACCTCCACGTGGACGGCGGAGGTGCCGCAGGCTGTGAACACCAAACGCAAGTGGGAGGCAGAAAAGAGCATCGCGGAGGGACAGAAAGCCTACCTGGAGGAGACGTGCGTGCTGTGGCTGAAGAAGTACCTGGAGATGGGGAAGGAGACGCTGAAGAGGACAGACCCGCCCTCTGCCCGAGTGACCCGCCACACTGACCCCCACGGGGAGGTGACCCTGCGGTGCCGGGCCCAGGACTTCTACCCCGAGGACATCTCCCTGACTTGGCTGAGGGATGGGGAGGAGCAGCTCCAGGACGCGGAGTTCATTGAGACCAGGCCCGCGGGGGAGGGGACCTTCCAGAAGTGGGCAGGTGTGGACGTGACCTTGGGCCAGGAAGGGAAATATACCTGCCGAGTTCAGCACGAGGGACTGCCTGAGCCCCTCACCCTGAAGTGGGAGCCAGAGTCCTCATTCCCCTGGTTCATTGTGGGGGGCGTTGCTGTCCTCCTCCTCCTCATTGCAGCCATTGCTGGAGTTGTGATCTGGAAGAAGAAGACTTCAGGTGGAAAAGGAGGGGACTATGTTCGTGCTGCAGGCAATGACAGTGCACAGGGGTCAGATGTCTCCCTCACAGTCAAAGCTTGA

>MHCI_4_UA_Hap_18

ATGGAGCCTTATCTGCGCGCTCTCTTTTTGCTGGGGACCCTGGCCCTGCCGGAGACCTGGGCAGGCTCTCACTCCCTGAGGTATTTCGACGCCGCCGTGGCCTCCCCCGAGCTCGCGGAGCCGCGGTTCCTCACCGTGGGCTACGTGGACGATCAGCAGTTCGTGCGCTTCGACAGTGCCCGCGCGAGTCCGAGGGAGGAGCCGCGGGCGGCGTGGATAGAGCGGGTGGAGCAGGAGGAGCCGGGCTACTGGGAGCAGGAAACGCAGAACATGAAGGCGGTCACACAGAATTACCGAGTGAGCCTGCAGAACCTCCGCGGCTACTTCAACCAGAGCGAAGGCGGGGTCCACACCATCCAGCACATGTACGGCTGCGAGGTCTCCCCCGAGCTCACCTTCAAGCGCGGGTTTCTCCAATACGCCTACGACGGGCGGGACTACATCGCCCTGGACTCGGAGACCTCCACGTGGACGGCGGAGGTGCCGCAGGCTCTGAACACCAAGCGCAAGTGGGAGGCGGAAAAGAGCATCGCGGAGGGACAGAAAGCCTACCTGGAGGAGACGTGCGTGCTGTGGCTGAAGAAGTACCTGGAGATGGGGAAGGAGACGCTGAAGAGGACAGACCCGCCCTCCGCCCGAGTGACCCGCCACACTGGCCCCCACGGGGAGGTGACCCTGCGGTGCCGGGCCCAGGACTTCTACCCCGCGGACATCTCCCTGACGTGGCTGAGGAATGGGGAGGAGCAGCTCCAGGACGCGGAGTTCATTGAGACCAGGCCCGCGGGGGAGGGGACCTTCCAGAAGTGGGCAGGTGTGGACGTGACCTCGGGCCAGGAAGGGAAATATACCTGCCGAGTTCAGCACGAGGGACTGCCTGAGCCCCTCACCCTGAAGTGGGAGCCAGAGTCCTCATTCCCCTGGTTCATTGTGGGGGGCGTTGCTGTCCTCCTCCTCCTCACTGCAGCCATTGCTGGAGTTGTGATCTGGAAGAAGAATACTTCAGGTGGAAAAGGAGGGGACTATGTTCGTGCTGCAGGCAATGACAGTGCACAGGGGTCAGATGTCTCCCTCACAGTCAAAGCTTGA

>MHCI_4_UA_Hap_6

ATGGAGGCTTATCTGCGCGCTCTCTTTTTGCTGGGGACCCTGGCCCTGCCGGAGACCTGGGCAGGCTCTCACTCCCTGAGGTATTTCTACACCGCCGTGGCCTCCCCCGAGCTCGCGGAGCCGCGGTTCCTCATCGTGGGCTACGTGGACGATCAGCAGTTCGTGCGCTTCGACAGTGCCCGCGCGAGTCCGAGGATGGAGCCGCGGGCGGCATGGATAGAGCGGGTGCAGCAGGAGGAGCCGGGCTACTGGGACCAGGAGACGCGGAACATGAAGGCGGTCACACAGACTTACCGAGTGAGCCTGCAGAACCTCCGCGGCTACTTCAACCAGAGCGAAGGCGGGGTCCACACCATCCAGCACATGTACGGCTGCGAGGTCTCCCCCGAGCTCACCTTCAAGCGCGGGTTTCTCCAATACGCCTACGACGGGCGGGACTATATCGCCCTGGACTCGGAGACCTCCACGTGGACGGCGGAGGTGCCGCAGGCTCTGAACACCAAGCGCAAGTGGGAGGCGGAAAAGAGCTACACGGAGGGACAGAAAGCCTACCTGGAGGAGACGTGCGTGCTGTGGCTGAAGAAGTACCTGGAGATGGGGAAGGAGACGCTGAAGAGGACAGACCCGCCCTCCGCCCGAGTGACCCGCCACACTGGCCCCCACGGGGAGGTGACCCTGCGGTGCCGGGCCCAGGACTTCTACCCCGAGGACATCTCCCTGACGTGGCTGAGGGATGGGGAGGAGCAGCTCCAGGACGCGGAGTTCATTGAGACCAGGCCGGCGGGGGAGGGGACCTTCCAGAAGTGGGCAGGTGTGGACGTGACCTCGGGCCAGGAAGGGAAATATACCTGCCGAGTTCAGCACGAGGGACTGCCTGAGCCCCTCACCCTGAAGTGGGAGCCAGAGTCCTCATCCCCCTGGTTCATCGTGGGGGGCATTGCTGTCCTCCTCCTCCTCACTGCAGCCATTGCTGGAGTTGTGATCTGGAAGAAGAATACTTCAGGTGGAAAAGGAGGGGACTATGTTCCTGCTGCAGGCAATGACAGTGCACAGGGGTCAGATGTCTCCCTCACAGTCAAAGCTTGA

>MHCI_4_UA_Hap_19

ATGGAGCCTTATCTGCGCGCTCTCTTTTTGCTGGGGACCCTGGCCCTGCCGGAGACCTGGGCAGGCTCTCACTCCCTGAGGTATTTCTACACCGCCGTGGCCTCCCCCGAGCTCGCGGAGCCGCGGTTCCTCATCGTGGGCTACGTGGACGATCAGCAGTTCGTGCGCTTCGACAGTGCCCGCGCGAGTCCGAGTATGGAGCCGCGGGCGGCGTGGATAGAGCGGGTGCAGCAGGAGGAGCCGGGCTACTGGGAGCGGCAAACGCAGAACATGAAGGCGGTCACACAGAATTACCGAGTGAACCTGCAGAACCTCCGCGGCTACTTCAACCAGAGCGAAGGCGGGGTCCACACCTTTCAGAACATGTACGGCTGCGAGGTCTCCCCCGAGCTCACCTTCAAGCGCGGGTTTCTCCAATACGCCTACGACGGGCGGGACTACATCGCCCTGGACTCGGAGACCTCCACGTGGACGGCGGAGGTGCCGCAGGCTGTGAACACCAAACGCAAGTGGGAGGCAGAAAAGAGCATCGCGGAGGGACAGAAAGCCTACCTGGAGGAGACGTGCGTGCTGTGGCTGAAGAAGTACCTGGAGATGGGGAAGGAGACGCTGAAGAGGACAGACCCGCCCTCTGCCCGAGTGACCCGCCACACTGACCCCCACGGGGAGGTGACCCTGCGGTGCCGGGCCCAGGACTTCTACCCCGAGGACATCTCCCTGACTTGGCTGAGGGATGGGGAGGAGCAGCTCCAGGACGCGGAGTTCATTGAGACCAGGCCCGCGGGGGAGGGGACCTTCCAGAAGTGGGCAGGTGTGGACGTGACCTTGGGCCAGGAAGGGAAATATACCTGCCGAGTTCAGCACGAGGGACTGCCTGAGCCCCTCACCCTGAAGTGGGAGCCAGAGTCCTCATTCCCCTGGTTCATTGTGGGGGGCGTTGCTGTCCTCCTCCTCCTCATTGCAGCCATTGCTGGAGTTGTGATCTGGAAGAAGAAGACTTCAGGTGGAAAAGGAGGGGACTATGTTCGTGCTGCAGGCAATGACAGTGCACAGGGGTCAGATGTCTCCCTCACAGTCAAAGCTTGA

>MHCI_4_UA_Hap_16

ATGGAGCCTTATCTGCGCGCTCTCTTTTTGCTGGGGACCCTGGCCCTGCCGGAGACCTGGGCAGGCTCTCACTCCCTGAGGTATTTCGACGCCGCCGTGGCCTCCCCCGAGCTCGCGGAGCCGCGGTTCCTCACCGTGGGCTACGTGGACGATCAGCAGTTCGTGCGCTTCGACAGTGCCCGCGCGAGTCCGAGTATGGAGCCGCGGGCGGCGTGGATAGAGCGGGTGCAGCAGGAGGAGCCGGGCTACTGGGACCAGGAGACGCGGAACATGAAGGCGGTCACACAGACTTACCGAGTGAGCCTGCAGAACCTCCGCGGCTACTTCAACCAGAGCGAAGGCGGGGTCCACACCATCCAGCACATGTACGGCTGCGAGGTCTCCCCCGAGCTCACCTTCAAGCGCGGGTTTCTCCAATACGCCTACGACGGGCGGGACTACATCGCCCTGGACTCGGAGACCTCCACGTGGACGGCGGAGGTGCCGCAGGCTCTGAACACCAAGCGCAAGTGGGAGGCGGAAAAGAGCATCGCGGAGGGACAGAAAGCCTACCTGGAGGAGACGTGCGTGCTGTGGCTGAAGAAGTACCTGGAGATGGGGAAGGAGACGCTGAAGAGGACAGACCCGCCCTCCGCCCGAGTGACCCGCCACACTGGCCCCCACGGGGAGGTGACCCTGCGGTGCCGGGCCCAGGACTTCTACCCCGCGGACATCTCCCTGACGTGGCTGAGGAATGGGGAGGAGCAGCTCCAGGACGCGGAGTTCATTGAGACCAGGCCCGCGGGGGAGGGGACCTTCCAGAAGTGGGCAGGTGTGGACGTGACCTCGGGCCAGGAAGGGAAATATACCTGCCGAGTTCAGCACGAGGGACTGCCTGAGCCCCTCACCCTGAAGTGGGAGCCAGAGTCCTCATCCCCCTGGCTCATTGTGGGGGGCATTGCTGTCCTCCTCCTCCTCACTGCAGCCATTGCTGGAGTTGTGATCTGGAAGAAGAAGACTTCAGGTGGAAAAGGAGGGGACTATGTTCGTGCTGCAGGCAATGACAGTGCACAGGGGTCAGATGTCTCCCTCACAGTCAAAGCTTGA

>MHCI_4_UA_Hap_20

ATGGAGCCTTATCTGCGCGCTCTCTTTTTGCTGGGGACCCTGGCCCTGCCGGAGACCTGGGCAGGCTCTCACTCCCTGAGGTATTTCGACACCGCCGTGGCCTCCCCCGAGCTCGCGGAGCCGCGGTTCCTCACCGTGGGCTACGTGGACGATCAGCAGTTCGTGCGCTTCGACAGTGCCCGCGCGAGTCCGAGGGAGGAGCCGCGGGCGGCGTGGATAGAGCGGGTGGAGCAGGAGGAGCCGGGCTACTGGGAGCAGGAAACGCAGAACATGAAGGCGGTCACACAGACTTACCGAGTGAACCTGCAGAACCTCCGCGGCTACTTCAACCAGAGCGAAGGCGGGGTCCACACCATCCAGCACATGTACGGCTGCGAGGTCTCCCCCGAGCTCACCTTCAAGCGCGGGTTTCTCCAATACGCCTACGACGGGCGGGACTACATCGCCCTGGACTCGGAGACCTCCACGTGGACGGCGGAGGTGCCGCAGGCTCTGAACACCAAACGCAAGTGGGAGGCAGAAAAGAGCTACACGGAGGGACAGAAAGCCTACCTGGAGGAGACGTGCGTGCTGTGGCTGAAGAAGTACCTGGAGATGGGGAAGGAGACGCTGAAGAGGACAGACCCGCCCTCCGCCCGAGTGACCCGCCACACTGGCCCCCACGGGGAGGTGACCCTGCGGTGCCGGGCCCAGGACTTCTACCCCGCGGACATCTCCCTGACGTGGCTGAGGAATGGGGAGGAGCAGCTCCAGGACGCGGAGTTCATTGAGACCAGGCCCGCGGGGGAGGGGACCTTCCAGAAGTGGGCAGGTGTGGACGTGACCTCGGGCCAGGAAGGGAAATATACCTGCCGAGTTCAGCACGAGGGACTGCCAGAGCCGCTCACCCTGAAGTGGGAGCCAGAGTCCTCATCCCCCTGGCTCATTGTGGGGGGCATTGCTGTCCTCCTCCTCCTCACTGCAGCCATTGCTGGAGTTGTGATCTGGAAGAAGAAGACTTCAGGTGGAAAAGGAGGGGACTATGTTCGTGCTGCAGGCAATGACAGTGCACAGGGGTCAGATGTCTCCCTCACAGTCAAAGCTTGA

>MHCI_4_UA_Hap_23

ATGGAGCCTTATCTGCGCGCTCTCTTTTTGCTGGGGACCCTGGCCCTGCCGGAGACCTGGGCAGGCTCTCACTCCCTGAGGTATTTCTACACCGCCGTGGCCTCCCCTGAGCTCGCGGAGCCGCGGTTCCTCACCGTGGGCTACGTGGACGATCAGCAGTTCGTGCGCTTCGACAGTGCCCGCGCGAGTCCGAGTATGGAGCCGCGGGCGGCGTGGATAGAGCGGGTGCAGCAGGAGGAGCCGGGCTACTGGGACCAGGAGACGCGGAACATGAAGGCGGTCACACAGACTTACCGAGTGAGCCTGCAGAACCTCCGCGGCTACTTCAACCAGAGCGAAGGCGGGGTCCACACCATCCAGCACATGTACGGCTGCGAGGTCTCCCCCGAGCTCACCTTCAAGCGCGGGTTTCACCAATACGCCTACGACGGGCGGGACTACATCGCCCTGGACTCGGAGACCTCCACGTGGACGGCGGAGGTGCCGCAGGCTCTGAACACCAAACGCAAGTGGGAGGCAGAAAAGAGCTACACGGAGGGACAGAAAGCCTACCTGGAGGAGACGTGCGTGCTGTGGCTGAAGAAGTACCTGGAGATGGGGAAGGAGACGCTGAAGAGGACAGAACCGCCCTCCGCCCGAGTGACCCGCCACACTGGCCCCCACGGGGAGGTGACCCTGCGGTGCCGGGCCCAGGACTTCTACCCCGAGGACGTCTCCCTGACGTGGCTGAGGAATGGGGAGGAGCAGCTCCAGGACGCGGAGTTCATTGAGACCAGGCCGGCGGGGGAGGGGACCTTCCAGAAGTGGGCAGGTGTGGACGTGACCTCGGGCCAGGAAGGGAAATATACCTGCCGAGTTCAGCACGAGGGACTGCCTGAGCCCCTCACCCTGAAGTGGGAGCCAGAGTCCTCATTCCCCTGGTTCATCGTGGGGGGCATTGCTGTCCTCCTCCTCCTCATTGCAGCCATTGCTGGAGTTGTGATCTGGAAGAAGAAGACTTCAGGTGGAAAAGGAGGGGACTATGTTCGTGCTGCAGGCAATGACAGTGCACAGGGGTCAGATGTCTCCCTCACAGTCAAAGCTTGA

>MHCI_4_UA_Hap_24

ATGGAGCCTTATCTGCGCGCTCTCTTTTTGCTGGGGACCCTGGCCCTGCCGGAGACCTGGGCAGGCTCTCACTCCCTGAGGTATTTCTACACCGCCGTGGCCTCCCCCGAGCTCGCGGAGCCGCGGTTCCTCATCGTGGGCTACGTGGACGATCAGCAGTTCGTGCGCTTCGACAGTGCCCGCGCGAGTCCGAGTATGGAGCCGCGGGCGGCATGGATAGAGCGGGTGCAGCAGGAGGAGCCGGGCTACTGGGACCAGGAGACGCGGAACATGAAGGCGGTCACACAGACTTACCGAGTGAGCCTGCAGAACCTCCGCGGCTACTTCAACCAGAGCGAAGGCGGGGTCCACACCATCCAGCACATGTACGGCTGCGAGGTCTCCCCCGAGCTCACCTTCAAGCGCGGGTTTCTCCAATACGCCTACGACGGGCGGGACTACATCGCCCTGGACTCGGAGACCTCCACGTGGACGGCGGAGGTGCCGCAGGCTCTGAACACCAAGCGCAAGTGGGAGGCGGAAAAGAGCATCGCGGAGGGACAGAAAGCCTACCTGGAGGAGACGTGCGTGCTGTGGCTGAAGAAGTACCTGGAGATGGGGAAGGAGACGCTGAAGAGGACAGACCCGCCCTCTGCCCGAGTGACCCGCCACACTGGCCCCCACGGGGAGGTGACCCTGCGGTGCCGGGCCCAGGACTTCTACCCCGAGGACATCTCCCTGACGTGGCTGAGGGATGGGGAGGAGCAGCTCCAGGACGCGGAGTTCATTGAGACCAGGCCGGCGGGGGAGGGGACCTTCCAGAAGTGGGCAGGTGTGGACGTGACCTCGGGCCAGGAAGGGAAATATACCTGCCGAGTTCAGCACGAGGGACTGCCTGAGCCCCTCACCCTGAAGTGGGAGCCAGAGTCCTCATCCCCCTGGTTCATCGTGGGGGGCATTGCTGTCCTCCTCCTCCTCACTGCAGCCATTGCTGGAGTTGTGATCTGGAAGAAGAAGACTTCAGGTGGAAAAGGAGGGGACTATGTTCGTGCTGCAGGCAATGACAGTGCACAGGGGTCAGATGTCTCCCTCACAGTCAAAGCTTGA

>MHCI_4_UA_Hap_26

ATGGAGCCTTATCTGCGCGCTCTCTTTTTGCTGGGGACCCTGGCCCTGCCGGAGACCTGGGCAGGCTCTCACTCCCTGAGGTATTTCTACACCGCCGTGGCCTCCCCTGAGCTCGCGGAGCCGCGGTTCCTCACCGTGGGCTACGTGGACGATCAGGAGTTCGTGCGCTTCGACAGTGCCCGCGCGAGTCCGAGTATGGAGCCGCGGGCGGCGTGGATAGAGCGGGTGCAGCAGGAGGAGCCGGGCTACTGGGAGCAGGAGACGCGGAACATGAAGGCGGTCACACAGACTTACCGAGTGAGCCTGCAGAACCTCCGCGGCTACTTCAACCAGAGCGAAGGCGGGGTCCACACCATCCAGCACATGTACGGCTGCGAGGTCTCCCCCGAGCTCACCTTCAAGCGCGGGTTTCACCAATACGCCTACGACGGGCGGGACTACATCGCCCTGGACTCGGAGACCTCCACGTGGACGGCGGAGGTGCCGCAGGCTCTGAACACCAAGCGCAAGTGGGAGGCAGAAAAGAGCTACACGGAGGGACAGAAAGCCTACCTGGAGGAGACGTGCGTGCTGTGGCTGAAGAAGTACCTGGAGATGGGGAAGGAGACGCTGAAGAGGACAGAACCGCCCTCCGCCCGAGTGACCCGCCACACTGGCCCCCACGGGGAGGTGACCCTGCGGTGCCGGGCCCAGGACTTCTACCCCGAGGACGTCTCCCTGACGTGGCTGAGGAATGGGGAGGAGCAGCTCCAGGACGCGGAGTTCATTGAGACCAGGCCGGCGGGGGAGGGGACCTTCCAGAAGTGGGCAGGTGTGGACGTGACCTCGGGCCAGGAAGGGAAATATACCTGCCGAGTTCAGCACGAGGGACTGCCTGAGCCCCTCACCCTGAAGTGGGAGCCAGAGTCCTCATTCCCCTGGTTCATCGTGGGGGGCATTGCTGTCCTCCTCCTCCTCATTGCAGCCATTGCTGGAGTTGTGATCTGGAAGAAGAAGACTTCAGGTGGAAAAGGAGGGGACTATGTTCGTGCTGCAGGCAATGACAGTGCACAGGGGTCAGATGTCTCCCTCACAGTCAAAGCTTGA

>MHCI_4_UA_Hap_25

ATGGAGGCTTATCTGCGCGCTCTCTTTTTGCTGGGGACCCTGGCCCTGCCGGAGACCTGGGCAGGCTCTCACTCCCTGAGGTATTTCTACACCGCCGTGGCCTCCCCCGAGCTCGCGGAGCCGCGGTTCCTCATCGTGGGCTACGTGGACGATCAGCAGTTCGTGCGCTTCGACAGTGCCCGCGCGAGTCCGAGGATGGAGCCGCGGGCGGCATGGATAGAGCGGGTGCAGCAGGAGGAGCCGGGCTACTGGGACCAGGAGACGCGGAACATGAAGGCGGTCACACAGACTTACCGAGTGAGCCTGCAGAACCTCCGCGGCTACTTCAACCAGAGCGAAGGCGGGGTCCACACCATCCAGCACATGTACGGCTGCGAGGTCTCCCCCGAGCTCACCTTCAAGCGCGGGTTTCTCCAATACGCCTACGACGGGCGGGACTACATCGCCCTGGACTCGGAGACCTCCACGTGGACGGCGGAGGTGCCGCAGGCTCTGAACACCAAGCGCAAGTGGGAGGCGGAAAAGAGCTACACGGAGGGACAGAAAGCCTACCTGGAGGAGACGTGCGTGCTGTGGCTGAAGAAGTACCTGGAGATGGGGAAGGAGACGCTGAAGAGGACAGACCCGCCCTCCGCCCGAGTGACCCGCCACACTGGCCCCCACGGGGAGGTGACCCTGCGGTGCCGGGCCCAGGACTTCTACCCCGAGGACATCTCCCTGACGTGGCTGAGGGATGGGGAGGAGCAGCTCCAGGACGCGGAGTTCATTGAGACCAGGCCGGCGGGGGAGGGGACCTTCCAGAAGTGGGCAGGTGTGGACGTGACCTCGGGCCAGGAAGGGAAATATACCTGCCGAGTTCAGCACGAGGGACTGCCTGAGCCCCTCACCCTGAAGTGGGAGCCAGAGTCCTCATCCCCCTGGTTCATCGTGGGGGGCATTGCTGTCCTCCTCCTCCTCACTGCAGCCATTGCTGGAGTTGTGATCTGGAAGAAGAATACTTCAGGTGGAAAAGGAGGGGACTATGTTCCTGCTGCAGGCAATGACAGTGCACAGGGGTCAGATGTCTCCCTCACAGTCAAAGCTTGA

>MHCI_4_UA_Hap_27

ATGGAGGCTTATCTGCGCGCTCTCTTTTTGCTGGGGACCCTGGCCCTGCCGGAGACCTGGGCAGGCTCTCACTCCCTGAGGTATTTCGACACCGCCGTGGCCTCCCCCGAGCTCGCGGAGCCGCGGTTCCTCACCGTGGGCTACGTGGACGATCAGCAGTTCGTGCGCTTCGACAGTGCCCGCGCGAGTCCGAGGGAGGAGCCGCGGGCGGCGTGGATAGAGCGGGTGGAGCAGGAGGAGCCGGGCTACTGGGAGCAGGAAACGCAGAACATGAAGGCGGTCACACAGACTTACCGAGTGAACCTGCAGAACCTCCGCGGCTACTTCAACCAGAGCGAAGGCGGGGTCCACACCATCCAGCACATGTACGGCTGCGAGGTCTCCCCCGAGCTCACCTTCAAGCGCGGGTTTCTCCAATACGCCTACGACGGGCGGGACTACATCGCCCTGGACTCGGAGACCTCCACGTGGACGGCGGAGGTGCCGCAGGCTCTGAACACCAAGCGCAAGTGGGAGGCGGAAAAGAGCATCGCGGAGGGACAGAAAGCCTACCTGGAGGAGACGTGCGTGCTGTGGCTGAAGAAGTACCTGGAGATGGGGAAGGAGACGCTGAAGAGGACAGACCCGCCCTCCGCCCGAGTGACCCGCCACACTGGCCCCCACGGGGAGGTGACCCTGCGGTGCCGGGCCCAGGACTTCTACCCCGCGGACATCTCCCTGACGTGGCTGAGGAATGGGGAGGAGCAGCTCCAGGACGCGGAGTTCATTGAGACCAGGCCCGCGGGGGAGGGGACCTTCCAGAAGTGGGCAGGTGTGGACGTGACCTCGGGCCAGGAAGGGAAATATACCTGCCGAGTTCAGCACGAGGGACTGCCAGAGCCGCTCACCCTGAAGTGGGAGCCAGAGTCCTCATCCCCCTGGCTCATTGTGGGGGGCATTGCTGTCCTCCTCCTCCTCACTGCAGCCATTGCTGGAGTTGTGATCTGGAAGAAGAAGACTTCAGGTGGAAAAGGAGGGGACTATGTTCGTGCTGCAGGCAATGACAGTGCACAGGGGTCAGATGTCTCCCTCACAGTCAAAGCTTGA

>MHCI_4_UA_Hap_3

ATGGAGGCTTATCTGCGCGCTCTCTTTTTGCTGGGGACCCTGGCCCTGCCGGAGACCTGGGCAGGCTCTCACTCCCTGAGGTATTTCTACACCGCCGTGGCCTCCCCTGAGCTCGCGGAGCCGCGGTTCCTCACCGTGGGCTACGTGGACGATCAGCAGTTCGTGCGCTTCGACAGTGCCCGCGCGAGTCCGAGTATGGAGCCGCGGGCGGCGTGGATAGAGCGGGTGCAGCAGGAGGAGCCGGGCTACTGGGAGCGGCAAACGCAGAACATGAAGGCGGTCACACAGAATTACCGAGTGAACCTGCAGAACCTCCTCGGCTACTTCAACCAGAGCGAAGGCGGGGTCCACACCTTTCAGAACATGTACGGCTGCGAGGTCTCCCCCGAGCTCACCTTCAAGCGCGGGTTTCAACAACACGCCTACGACGGGCGGGACTATATCACCCTGGACTCGGAGACCTCCACGTGAACGGCGGAGGTGCCGCAGGCTCTGAACACCAAACGCAAGTGGGAGGCAGAAAAGAGCTACACGGAGGGACAGAAAGCCTACCTGGAGGAGACGTGCGTGCTGTGGCTGAAGAAGTACCTGGAGATGGGGAAGGAGACGCTGAAGAGGACAGAACCGCCCTCTGCCCGAGTGACCCGCCACACTGGCCCCCACGGGGAGGTGACCCTGCGGTGCCGGGCCCAGGACTTCTACCCCGAGGACGTCTCCCTGACGTGGCTGAGGGATGGGGAGGAGCAGCTCCAGGACGCGGAGTTCATTGAGACCAGGCCGGCGGGGGAGGGGACCTTCCAGAAGTGGGCAGGTGTGGACGTGACCTCGGGCCAGGAAGGGAAATATACCTGCCGAGTTCAGCACGAGGGACTGCCTGAGCCCCTCACCCTGAAGTGGGAGCCAGAGTCCTCATTCCCCTGGTTCATCGTGGGGGGCATTGCTGTCCTCCTCCTCCTCATTGCAGCCATTGCTGGAGTTGTGATCTGGAAGAAGAATACTTCAGGTGGAAAAGGAGGGGACTATGTTCCTGCTGCAGGCAATGACAGTGCACAGGGGTCAGATGTCTCCCTCACAGTCAAAGCTTGA

>MHCI_4_UA_Hap_5

ATGGAGGCTTATCTGCGCGCTCTCTTTTTGCTGGGGACCCTGGCCCTGCCGGAGACCTGGGCAGGCTCTCACTCCCTGAGGTATTTCTACACCGCCGTGGCCTCCCCTGAGCTCGCGGAGCCGCGGTTCCTCACCGTGGGCTACGTGGACGATCAGCAGTTCGTGCGCTTCGACAGTGCCCGCGCGAGTCCGAGTATGGAGCCGCGGGCGGCGTGGATAGAGCGGGTGCAGCAGGAGGAGCCGGGCTACTGGGAGCGGCAAACGCAGAACATGAAGGCGGTCACACAGAATTACCGAGTGAACCTGCAGAACCTCCTCGGCTACTTCAACCAGAGCGAAGGCGGGGTCCACACCTTTCAGAACATGTACGGCTGCGAGGTCTCCCCCGAGCTCACCTTCAAGCGCGGGTTTCAACAACACGCCTACGACGGGCGGGACTATATCACCCTGGACTCGGAGACCTCCACGTGAACGGCGGAGGTGCCGCAGGCTCTGAACACCAAACGCAAGTGGGAGGCAGAAAAGAGCTACACGGAGGGACAGAAAGCCTACCTGGAGGAGACGTGCGTGCTGTGGCTGAAGAAGTACCTGGAGATGGGGAAGGAGACGCTGAAGAGGACAGAACCGCCCTCCGCCCGAGTGACCCGCCACACTGGCCCCCACGGGGAGGTGACCCTGCGGTGCCGGGCCCAGGACTTCTACCCCGAGGACGTCTCCCTGACGTGGCTGAGGAATGGGGAGGAGCAGCTCCAGGACGCGGAGTTCATTGAGACCAGGCCGGCGGGGGAGGGGACCTTCCAGAAGTGGGCAGGTGTGGACGTGACCTCGGGCCAGGAAGGGAAATATACCTGCCGAGTTCAGCACGAGGGACTGCCTGAGCCCCTCACCCTGAAGTGGGAGCCAGAGTCCTCATTCCCCTGGTTCATCGTGGGGGGCATTGCTGTCCTCCTCCTCCTCATTGCAGCCATTGCTGGAGTTGTGATCTGGAAGAAGAATACTTCAGGTGGAAAAGGAGGGGACTATGTTCCTGCTGCAGGCAATGACAGTGCACAGGGGTCAGATGTCTCCCTCACAGTCAAAGCTTGA

>MHCI_4_UA_Hap_9

ATGGAGGCTTATCTGCGCGCTCTCTTTTTGCTGGGGACCCTGGCCCTGCCGGAGACCTGGGCAGGCTCTCACTCCCTGAGGTATTTCTACACCGCCGTGGCCTCCCCTGAGCTCGCGGAGCCGCGGTTCCTCACCGTGGGCTACGTGGACGATCAGGAGTTCGTGCGCTTCGACAGTGCCCGCGCGAGTCCGAGGGAGGAGCCGCGGGCGGCGTGGATAGAGCGGGTGCAGCAGGAGGAGCCGGGCTACTGGGACCAGGAGACGCGGAACATGAAGGCGGTCACACAGACTTACCGAGTGAGCCTGCAGAACCTCCGCGGCTACTTCAACCAGAGCGAAGGCGGGGTCCACACCTTTCAGAACATGTACGGCTGCGAGGTCTCCCCCGAGCTCACCTTCAAGCGCGGGTTTCAACAACACGCCTACGACGGGCGGGACTATATCACCCTGGACTCGGAGACCTCCACGTGAACGGCTGAGGTGCCGCAGGCTCTGAACACCAAGCGCAAGTGGGAGGCAGAAAAGAGCATCGCGGAGGGACAGAAAGCCTACCTGGAGGAGACGTGCGTGCTGTGGCTGAAGAAGTACCTGGAGATGGGGAAGGAGACGCTGAAGAGGACAGACCCGCCCTCTGCCCGAGTGACCCGCCACACTGGCCCCCACGGGGAGGTGACCCTGCGGTGCCGGGCCCAGGACTTCTACCCCGAGGACATCTCCCTGACGTGGCTGAGGGATGGGGAGGAGCAGCTCCAGGACGCGGAGTTCATTGAGACCAGGCCGGCGGGGGAGGGGACCTTCCAGAAGTGGGCAGGTGTGGACGTGACCTCGGGCCAGGAAGGGAAATATACCTGCCGAGTTCAGCACGAGGGACTGCCTGAGCCCCTCACCCTGAAGTGGGAGCCAGAGTCCTCATCCCCCTGGTTCATCGTGGGGGGCATTGCTGTCCTCCTCCTCCTCACTGCAGCCATTGCTGGAGTTGTGATCTGGAAGAAGAATACTTCAGGTGGAAAAGGAGGGGACTATGTTCCTGCTGCAGGCAATGACAGTGCACAGGGGTCAGATGTCTCCCTCACAGTCAAAGCTTGA

>MHCI_4_UA_Hap_11

ATGGAGGCTTATCTGCGCGCTCTCTTTTTGCTGGGGACCCTGGCCCTGCCGGAGACCTGGGCAGGCTCTCACTCCCTGAGGTATTTCTACACCGCCGTGGCCTCCCCTGAGCTCGCGGAGCCGCGGTTCCTCACCGTGGGCTACGTGGACGATCAGGAGTTCGTGCGCTTCGACAGTGCCCGCGCGAGTCCGAGGGAGGAGCCGCGGGCGGCGTGGATAGAGCGGGTGCAGCAGGAGGAGCCGGGCTACTGGGAGCAGGAGACGCGGAACATGAAGGCGGTCACACAGACTTACCGAGTGAGCCTGCAGAACCTCCGCGGCTACTTCAACCAGAGCGAAGGCGGGGTCCACACCTTTCAGAACATGTACGGCTGCGAGGTCTCCCCCGAGCTCACCTTCAAGCGCGGGTTTCAACAACACGCCTACGACGGGCGGGACTATATCACCCTGGACTCGGAGACCTCCACGTGAACGGCTGAGGTGCCGCAGGCTCTGAACACCAAGCGCAAGTGGGAGGCAGAAAAGAGCATCGCGGAGGGACAGAAAGCCTACCTGGAGGAGACGTGCGTGCTGTGGCTGAAGAAGTACCTGGAGATGGGGAAGGAGACGCTGAAGAGGACAGACCCGCCCTCTGCCCGAGTGACCCGCCACACTGGCCCCCACGGGGAGGTGACCCTGCGGTGCCGGGCCCAGGACTTCTACCCCGAGGACATCTCCCTGACGTGGCTGAGGGATGGGGAGGAGCAGCTCCAGGACGCGGAGTTCATTGAGACCAGGCCGGCGGGGGAGGGGACCTTCCAGAAGTGGGCAGGTGTGGACGTGACCTCGGGCCAGGAAGGGAAATATACCTGCCGAGTTCAGCACGAGGGACTGCCTGAGCCCCTCACCCTGAAGTGGGAGCCAGAGTCCTCATCCCCCTGGTTCATCGTGGGGGGCATTGCTGTCCTCCTCCTCCTCACTGCAGCCATTGCTGGAGTTGTGATCTGGAAGAAGAATACTTCAGGTGGAAAAGGAGGGGACTATGTTCCTGCTGCAGGCAATGACAGTGCACAGGGGTCAGATGTCTCCCTCACAGTCAAAGCTTGA

>MHCI_4_UA_Hap_14

ATGGAGGCTTATCTGCGCGCTCTCTTTTTGCTGGGGACCCTGGCCCTGCCGGAGACCTGGGCAGGCTCTCACTCCCTGAGGTATTTCTACACCGCCGTGGCCTCCCCTGAGCTCGCGGAGCCGCGGTTCCTCACCGTGGGCTACGTGGACGATCAGGAGTTCGTGCGCTTCGACAGTGCCCGCGCGAGTCCGAGGGAGGAGCCGCGGGCGGCGTGGATAGAGCGGGTGCAGCAGGAGGAGCCGGGCTACTGGGACCAGGAGACGCGGAACATGAAGGCGGTCACACAGACTTACCGAGTGAGCCTGCAGAACCTCCGCGGCTACTTCAACCAGAGCGAAGGCGGGGTCCACACCTTTCAGAACATGTACGGCTGCGAGGTCTCCCCCGAGCTCACCTTCAAGCGCGGGTTTCAACAACACGCCTACGACGGGCGGGACTATATCACCCTGGACTCGGAGACCTCCACGTGAACGGCTGAGGTGCCGCAGGCTCTGAACACCAAACGCAAGTGGGAGGCAGAAAAGAGCTACACGGAGGGACAGAAAGCCTACCTGGAGGAGACGTGCGTGCTGTGGCTGAAGAAGTACCTGGAGATGGGGAAGGAGACGCTGAAGAGGACAGAACCGCCCTCCGCCCGAGTGACCCGCCACACTGGCCCCCACGGGGAGGTGACCCTGCGGTGCCGGGCCCAGGACTTCTACCCCGAGGACGTCTCCCTGACGTGGCTGAGGAATGGGGAGGAGCAGCTCCAGGACGCGGAGTTCATTGAGACCAGGCCGGCGGGGGAGGGGACCTTCCAGAAGTGGGCAGGTGTGGACGTGACCTCGGGCCAGGAAGGGAAATATACCTGCCGAGTTCAGCACGAGGGACTGCCTGAGCCCCTCACCCTGAAGTGGGAGCCAGAGTCCTCATTCCCCTGGTTCATCGTGGGGGGCATTGCTGTCCTCCTCCTCCTCATTGCAGCCATTGCTGGAGTTGTGATCTGGAAGAAGAATACTTCAGGTGGAAAAGGAGGGGACTATGTTCCTGCTGCAGGCAATGACAGTGCACAGGGGTCAGATGTCTCCCTCACAGTCAAAGCTTGA

>MHCI_4_UA_Hap_15

ATGGAGGCTTATCTGCGCGCTCTCTTTTTGCTGGGGACCCTGGCCCTGCCGGAGACCTGGGCAGGCTCTCACTCCCTGAGGTATTTCTACACCGCCGTGGCCTCCCCTGAGCTCGCGGAGCCGCGGTTCCTCACCGTGGGCTACGTGGACGATCAGGAGTTCGTGCGCTTCGACAGTGCCCGCGCGAGTCCGAGTATGGAGCCGCGGGCGGCGTGGATAGAGCGGGTGCAGCAGGAGGAGCCGGGCTACTGGGAGCGGCAAACGCAGAACATGAAGGCGGTCACACAGACTTACCGAGTGAACCTGCAGAACCTCCGCGGCTACTTCAACCAGAGCGAAGGCGGGGTCCACACCTTTCAGAACATGTACGGCTGCGAGGTCTCCCCCGAGCTCACCTTCAAGCGCGGGTTTCAACAACACGCCTACGACGGGCGGGACTATATCACCCTGGACTCGGAGACCTCCACGTGAACGGCTGAGGTGCCGCAGGCTCTGAACACCAAGCGCAAGTGGGAGGCAGAAAAGAGCTACACGGAGGGACAGAAAGCCTACCTGGAGGAGACGTGCGTGCTGTGGCTGAAGAAGTACCTGGAGATGGGGAAGGAGACGCTGAAGAGGACAGAACCGCCCTCTGCCCGAGTGACCCGCCACACTGGCCCCCACGGGGAGGTGACCCTGCGGTGCCGGGCCCAGGACTTCTACCCCGAGGACGTCTCCCTGACGTGGCTGAGGGATGGGGAGGAGCAGCTCCAGGACGCGGAGTTCATTGAGACCAGGCCGGCGGGGGAGGGGACCTTCCAGAAGTGGGCAGGTGTGGACGTGACCTCGGGCCAGGAAGGGAAATATACCTGCCGAGTTCAGCACGAGGGACTGCCTGAGCCCCTCACCCTGAAGTGGGAGCCAGAGTCCTCATTCCCCTGGTTCATCGTGGGGGGCATTGCTGTCCTCCTCCTCCTCATTGCAGCCATTGCTGGAGTTGTGATCTGGAAGAAGAATACTTCAGGTGGAAAAGGAGGGGACTATGTTCCTGCTGCAGGCAATGACAGTGCACAGGGGTCAGATGTCTCCCTCACAGTCAAAGCTTGA

>MHCI_4_UA_Hap_21

ATGGAGGCTTATCTGCGCGCTCTCTTTTTGCTGGGGACCCTGGCCCTGCCGGAGACCTGGGCAGGCTCTCACTCCCTGAGGTATTTCTACACCGCCGTGGCCTCCCCTGAGCTCGCGGAGCCGCGGTTCCTCACCGTGGGCTACGTGGACGATCAGGAGTTCGTGCGCTTCGACAGTGCCCGCGCGAGTCCGAGGGAGGAGCCGCGGGCGGCGTGGATAGAGCGGGTGCAGCAGGAGGAGCCGGGCTACTGGGACCAGGAGACGCGGAACATGAAGGCGGTCACACAGACTTACCGAGTGAGCCTGCAGAACCTCCGCGGCTACTTCAACCAGAGCGAAGGCGGGGTCCACACCTTTCAGAACATGTACGGCTGCGAGGTCTCCCCCGAGCTCACCTTCAAGCGCGGGTTTCACCAATACGCCTACGACGGGCGGGACTATATCGCCCTGGACTCGGAGACCTCCACGTGAACGGCTGAGGTGCCGCAGGCTCTGAACACCAAACGCAAGTGGGAGGCAGAAAAGAGCTACACGGAGGGACAGAAAGCCTACCTGGAGGAGACGTGCGTGCTGTGGCTGAAGAAGTACCTGGAGATGGGGAAGGAGACGCTGAAGAGGACAGAACCGCCCTCCGCCCGAGTGACCCGCCACACTGGCCCCCACGGGGAGGTGACCCTGCGGTGCCGGGCCCAGGACTTCTACCCCGAGGACGTCTCCCTGACGTGGCTGAGGAATGGGGAGGAGCAGCTCCAGGACGCGGAGTTCATTGAGACCAGGCCGGCGGGGGAGGGGACCTTCCAGAAGTGGGCAGGTGTGGACGTGACCTCGGGCCAGGAAGGGAAATATACCTGCCGAGTTCAGCACGAGGGACTGCCTGAGCCCCTCACCCTGAAGTGGGAGCCAGAGTCCTCATTCCCCTGGTTCATCGTGGGGGGCATTGCTGTCCTCCTCCTCCTCATTGCAGCCATTGCTGGAGTTGTGATCTGGAAGAAGAATACTTCAGGTGGAAAAGGAGGGGACTATGTTCCTGCTGCAGGCAATGACAGTGCACAGGGGTCAGATGTCTCCCTCACAGTCAAAGCTTGA

>MHCI_4_UA_Hap_22

ATGGAGGCTTATCTGCGCGCTCTCTTTTTGCTGGGGACCCTGGCCCTGCCGGAGACCTGGGCAGGCTCTCACTCCCTGAGGTATTTCTACACCGCCGTGGCCTCCCCCGAGCTCGCGGAGCCGCGGTTCCTCATCGTGGGCTACGTGGACGATCAGCAGTTCGTGCGCTTCGACAGTGCCCGCGCGAGTCCGAGTATGGAGCCGCGGGCGGCATGGATAGAGCGGGTGCAGCAGGAGGAGCCGGGCTACTGGGACCAGGAGACGCGGAACATGAAGGCGGTCACACAGACTTACCGAGTGAGCCTGCAGAACCTCCGCGGCTACTTCAACCAGAGCGAAGGCGGGGTCCACACCATCCAGCACATGTACGGCTGCGAGGTCTCCCCCGAGCTCACCTTCAAGCGCGGGTTTCTCCAATACGCCTACGACGGGCGGGACTACATCGCCCTGGACTCGGAGACCTCCACGTGGACGGCGGAGGTGCCGCAGGCTCTGAACACCAAGCGCAAGTGGGAGGCGGAAAAGAGCTACACGGAGGGACAGAAAGCCTACCTGGAGGAGACGTGCGTGCTGTGGCTGAAGAAGTACCTGGAGATGGGGAAGGAGACGCTGAAGAGGACAGACCCGCCCTCCGCCCGAGTGACCCGCCACACTGGCCCCCACGGGGAGGTGACCCTGCGGTGCCGGGCCCAGGACTTCTACCCCGAGGACATCTCCCTGACGTGGCTGAGGGATGGGGAGGAGCAGCTCCAGGACGCGGAGTTCATTGAGACCAGGCCGGCGGGGGAGGGGACCTTCCAGAAGTGGGCAGGTGTGGACGTGACCTCGGGCCAGGAAGGGAAATATACCTGCCGAGTTCAGCACGAGGGACTGCCTGAGCCCCTCACCCTGAAGTGGGAGCCAGAGTCCTCATCCCCCTGGTTCATCGTGGGGGGCATTGCTGTCCTCCTCCTCCTCACTGCAGCCATTGCTGGAGTTGTGATCTGGAAGAAGAATACTTCAGGTGGAAAAGGAGGGGACTATGTTCCTGCTGCAGGCAATGACAGTGCACAGGGGTCAGATGTCTCCCTCACAGTCAAAGCTTGA
